# Supplementary material for: Developing a core outcome set for traumatic brachial plexus injuries: a systematic review of outcomes
Source: BMJ Open. 2021 Jul 30;11(7):e044797. doi: 10.1136/bmjopen-2020-044797 (PMC8327802; doi:10.1136/bmjopen-2020-044797)
Supplement: Supplementary data [file bmjopen-2020-044797supp001.pdf]

Developing a core outcome set for traumatic brachial plexus injuries: a systematic review of outcomes

Supplementary file 1. Deviations from study protocol

**Protocol method**	**Deviation from protocol method with justification**
We planned to hand search Journal of Hand Surgery (Eur) and The Journal of Hand Surgery (American).	We did not hand search these Journals as they were all indexed for MEDLINE.
We planned to include studies with participants aged 18 and over within the review.	We reduced the age of include participants to 16 or over as many studies included older teenagers with adults in their studies. On discussion with the research team we concluded that there was no difference between treatment of those aged 16 and over versus aged 18. If we excluded these studies many outcomes used across these age ranges would have been lost.
One search date was originally proposed in the study protocol	We updated the search in May 2021, including prospective and randomized controlled trials to ensure that the outcomes identified and reported in the publication reflected current outcomes in the literature and to ensure that no outcomes were omitted.
No quality assessment was proposed in the original study protocol	Outcome reporting bias was assessed in the included prospective and randomized controlled trials. This was included as it was thought this could improve understanding on what outcomes authors prioritise.
