# Supplementary material for: Developing a core outcome set for traumatic brachial plexus injuries: a systematic review of outcomes
Source: BMJ Open. 2021 Jul 30;11(7):e044797. doi: 10.1136/bmjopen-2020-044797 (PMC8327802; doi:10.1136/bmjopen-2020-044797)
Supplement: Supplementary data [file bmjopen-2020-044797supp002.pdf]

Supplementary file 2. Search strategy systematic review outcome reporting traumatic brachial plexus injuries

Search strategy 10/09/2018 COMBINE systematic review (reran 07 May 2021)

MEDLINE (OVID)

1.(brachial plexus adj3 injur*).mp. [mp=title, abstract, original title, name of substance word, subject heading word, floating sub-heading word, keyword heading word, protocol supplementary concept word, rare disease supplementary concept word, unique identifier, synonyms]

2 (brachial plexus adj3 pals*).mp. [mp=title, abstract, original title, name of substance word, subject heading word, floating sub-heading word, keyword heading word, protocol supplementary concept word, rare disease supplementary concept word, unique identifier, synonyms]

3 (brachial plexus adj3 lesion*).mp. [mp=title, abstract, original title, name of substance word, subject heading word, floating sub-heading word, keyword heading word, protocol supplementary concept word, rare disease supplementary concept word, unique identifier, synonyms]

4 brachial plexopath*.mp. [mp=title, abstract, original title, name of substance word, subject heading word, floating sub-heading word, keyword heading word, protocol supplementary concept word, rare disease supplementary concept word, unique identifier, synonyms]

5 (brachial plexus adj3 traction*).mp. [mp=title, abstract, original title, name of substance word, subject heading word, floating sub-heading word, keyword heading word, protocol supplementary concept word, rare disease supplementary concept word, unique identifier, synonyms]

6 (brachial plexus adj3 avulsion*).mp. [mp=title, abstract, original title, name of substance word, subject heading word, floating sub-heading word, keyword heading word, protocol supplementary concept word, rare disease supplementary concept word, unique identifier, synonyms]

7 Brachial Plexus/in, su, tr [Injuries, Surgery, Transplantation]

8 1 or 2 or 3 or 4 or 5 or 6 or 7

9 limit 8 to (humans and "all adult (19 plus years)")

10. limit 9 to yr="2013 -Current

Supplementary file 2. Search strategy systematic review outcome reporting traumatic brachial plexus injuries
