# Supplementary material for: Developing a core outcome set for traumatic brachial plexus injuries: a systematic review of outcomes
Source: BMJ Open. 2021 Jul 30;11(7):e044797. doi: 10.1136/bmjopen-2020-044797 (PMC8327802; doi:10.1136/bmjopen-2020-044797)
Supplement: Supplementary data [file bmjopen-2020-044797supp003.pdf]

Supplementary file 3. Outcome reporting bias assessment instrument

Outcome Reporting Bias assessment instrument (adapted from Deshmukh et al 2021)

**Study ID**	**Author**	**Registered**	**No clear reporting of outcome through description/table/figure**	**Outcome only by summary comment (e.g. there was no significant difference), no numerical values provided, lack of information so that reporting not meaningful (outcomes but no timepoints)**	**Outcome reported but not at all timepoints; lacks detail to be included in review**	**Outcome reported at all time points (methods, Results)**	**Outcome not specified in registration or prior to results**
			**NOT DONE**	**MIMIMAL**	**PARTIAL**	**COMPLETE**	**UNEXPECTED**

Deshmukh SR, Mousoulis C, Marson BA et al. Developing a core outcome set for hand fractures and joint injuries in adults: a systematic review. Journal of Hand Surgery (Eur) 2021;46(5):488-495
