# Supplementary material for: Developing a core outcome set for traumatic brachial plexus injuries: a systematic review of outcomes
Source: BMJ Open. 2021 Jul 30;11(7):e044797. doi: 10.1136/bmjopen-2020-044797 (PMC8327802; doi:10.1136/bmjopen-2020-044797)
Supplement: Supplementary data [file bmjopen-2020-044797supp004.pdf]

Supplementary File 4 COMET outcome taxonomy

Title: Supplemental File 4 COMET outcome taxonomy - adapted from Dodd et al (2018)

**Core Area**	Outcome Domain
**Death**	1. Mortality/ survival
**Physiological/clinical**	2. Blood and lymphatic system outcomes
	3. Cardiac outcomes
	4. Congenital, familial and genetic outcomes
	5. Endocrine outcomes
	6. Ear and labyrinth outcomes
	7. Eye outcomes
	8. Gastrointestinal outcomes
	9. General outcomes
	10. Hepatobilary outcomes
	11. Immune system outcomes
	12. Infection and infestation outcomes
	13. Injury and poisoning outcomes
	14. Metabolism and nutrition outcomes
	15. Musculoskeletal and connective tissue outcomes
	16. Outcomes, relating to neoplasms: benign, malignant and unspecified (including cysts and polyps)
	17. Nervous system outcomes
	18. Pregnancy, puerperium and perinatal outcomes
	19. Renal and urinary outcomes
	20. Reproductive system and breast outcomes
	21. Psychiatric outcomes
	22. Respiratory, thoracic and mediastinal outcomes
	23. Skin and subcutaneous tissue outcomes
	24. Vascular outcomes
**Life Impact**	**Functioning**
	25. Physical functioning
	26. Social functioning
	27. Role functioning
	28. Emotional functioning/ well being
	29. Cognitive functioning
	30. Global quality of life
	31. Perceived health status
	32. Delivery of care
	33. Personal circumstances
**Resource use**	**Resource Use**
	34. Economic
	35. Hospital
	36. Need for further intervention
	37. Societal/ carer burden
**Adverse Events**	38. Adverse Events / effects

Dodd S, Clarke M, Becker L et al. A taxonomy has been developed for outcomes in medical research to help improve knowledge discovery. *J Clin Epidemiol*. 2018;96:84-92.
