# Supplementary material for: Developing a core outcome set for traumatic brachial plexus injuries: a systematic review of outcomes
Source: BMJ Open. 2021 Jul 30;11(7):e044797. doi: 10.1136/bmjopen-2020-044797 (PMC8327802; doi:10.1136/bmjopen-2020-044797)
Supplement: Supplementary data [file bmjopen-2020-044797supp005.pdf]

Supplementary file 5. Included Studies

	**Study title**	**First author**	**Year of publication**
**1**	Effectiveness and safety of home-based muscle electrical stimulator in brachial plexus Injury patient	Limthongthang	2014
**2**	Elbow proprioception sense in total arm -type brachial plexus injured patients after neurotisation: a preliminary study	Homreprasert	2014
**3**	Comparison between the anterior and posterior approach for transfer of the spinal accessory nerve to the suprascapular nerve in late traumatic brachial plexus injuries	Souza	2014
**4**	Ultrasound-guided peripheral nerve stimulation for neuropathic pain after brachial plexus injury: two case reports	Kim	2017
**5**	Contralateral lower trapezius transfer for restoration of shoulder external rotation in traumatic brachial plexus palsy: preliminary report and literature review	Satbhai	2014
**6**	Restoration of shoulder abduction in brachial plexus avulsion injuries with double neurotization from the spinal accessory nerve: a report of 13 cases	Huan	2017
**7**	Transfer of the musculocutaneous nerve branch to the brachialis muscle to the triceps for elbow extension: anatomical study and report of five cases	Bertelli	2017
**8**	Posterior approach for accessory to suprascapular nerve transfer: an electrophysiological outcomes study	Rui	2013
**9**	Reliability of functioning free muscle transfer and vascularized ulnar nerve grafting for elbow flexion in complete brachial plexus palsy	Potter	2017
**10**	Management of infraclavicular (Chuang Level IV) brachial plexus injuries: A single surgeon experience with 75 cases	Lam	2015
**11**	Functioning free muscle transfer for the restoration of elbow flexion in brachial plexus injury patients	Estrella	2016
**12**	Radial to axillary nerve transfers: A combined case series	Desai	2016
**13**	Thalamic deep brain stimulation for neuropathic pain after amputation or brachial plexus avulsion	Pereira	2013
**14**	Nerve transfers for shoulder function for traumatic brachial plexus injuries	Estrella	2014
**15**	Results of operative treatment of brachial plexus injury resulting from shoulder dislocation: A study with a long-term follow-up	Gutkowska	2017
**16**	Surgical treatment of brachial plexus posterior cord lesion: A combination of nerve and tendon transfers, about nine patients	Oberlin	2013
**17**	The medial cord to musculocutaneous (MCMc) nerve transfer: a new method to reanimate elbow flexion after C5-C6-C7-(C8) avulsive injuries of the brachial plexus—technique and results	Ferraresi	2014
**18**	Transfer of a terminal motor branch nerve to the flexor carpi ulnaris for triceps reinnervation: anatomical study and clinical cases	Bertelli	2015
**19**	Free functioning gracilis muscle transfer with and without simultaneous intercostal nerve transfer to musculocutaneous nerve for restoration of elbow flexion after traumatic adult brachial pan-plexus injury	Maldonado	2017(a)

Article title: Developing a core outcome set for Traumatic Brachial Plexus Injuries: a systematic review of outcomes

Supplementary file 5. Included Studies

**20**	Isolated latissimus dorsi transfer to restore shoulder external rotation in adults with brachial plexus injury	Ghosh	2013
**21**	Functional outcome and quality of life after traumatic total brachial plexus injury treated by nerve transfer or single/double free muscle transfers	Satbhai	2016
**22**	Successful graded mirror therapy in a patient with chronic deafferentation pain in whom traditional mirror therapy was ineffective: A case report	Mibu	2016
**23**	Bipolar Transfer of Latissimus Dorsi Myocutaneous Flap for Restoration of Elbow Flexion in Late Traumatic Brachial Plexus Injury: Evaluation of 13 Cases	Azab	2017
**24**	Comparison of objective muscle strength in C5-C6 and C5-C7 brachial plexus injury patients after double nerve transfer	Tsai	2014
**25**	Phantom remodeling effect of dorsal root entry zone lesioning in phantom limb pain caused by brachial plexus avulsion	Son	2015
**26**	Comparison of surgical strategies between proximal nerve graft and/or nerve transfer and distal nerve transfer based on functional restoration of elbow flexion: A retrospective review of 147 patients	Hu	2018
**27**	Reconstruction of shoulder abduction by multiple nerve fascicle transfer through posterior approach	Ren	2013
**28**	Intercostal nerve transfer to neurotize the musculocutaneous nerve after traumatic brachial plexus avulsion: A comparison of two, three, and four nerve transfers	Xiao	2014
**29**	Use of the DEKA Arm for amputees with brachial plexus injury: A case series	Resnik	2017
**30**	Polyester tape scapulopexy for chronic upper extremity brachial plexus injury	Leechavengvongs	2015
**31**	Contralateral C7 nerve transfer with direct coaptation to restore lower trunk function after traumatic brachial plexus avulsion	Wang	2013
**32**	Outcome of surgical reconstruction after traumatic total brachial plexus palsy	Dodakundi	2013
**33**	Bionic reconstruction to restore hand function after brachial plexus injury: a case series of three patients	Aszmann	2015
**34**	Surgical treatment of the plexus brachialis injury using long-lasting electrostimulation	Tsymbalyuk	2013
**35**	Phrenic nerve transfer for reconstruction of elbow extension in severe brachial plexus injuries	Flores	2016
**36**	Direct coaptation of the phrenic nerve with the posterior division of the lower trunk to restore finger and elbow extension function in patients with total brachial plexus injuries	Wang	2016
**37**	A prospective study comparing single and double fascicular transfer to restore elbow flexion after brachial plexus injury	Martins	2013
**38**	Chronic post-traumatic neuropathic pain of brachial plexus and upper limb: a new technique of peripheral nerve stimulation	Stevanato	2014

Article title: Developing a core outcome set for Traumatic Brachial Plexus Injuries: a systematic review of outcomes

Supplementary file 5. Included Studies

**39**	Effectiveness of contralateral C7 nerve root and multiple nerve transfer for treatment of brachial plexus root avulsion	Wei	2014
**40**	Combined proximal nerve graft and distal nerve transfer for a posterior cord brachial plexus injury	Plate	2013
**41**	The role of elective amputation in patients with traumatic brachial plexus injury	Maldonado	2016
**42**	Early microsurgical management of clavicular fracture combined with brachial plexus injury	Liu	2014(a)
**43**	Contralateral trapezius transfer to restore shoulder external rotation following adult brachial plexus injury	Elhassan	2016
**44**	Comparative study of phrenic nerve transfers with and without nerve graft for elbow flexion after global brachial plexus injury	Liu	2014
**45**	Shoulder and elbow recovery at 2 and 11 years following brachial plexus reconstruction	Wang	2016
**46**	Functional outcomes after treatment of traumatic brachial plexus injuries: clinical study	Aras	2013
**47**	Free gracilis transfer reinnervated by the nerve to the supinator for the reconstruction of finger and thumb extension in longstanding C7-T1 brachial plexus root avulsion	Soldado	2013
**48**	Restoration of hand function in C7–T1 brachial plexus palsies using a staged approach with nerve and tendon transfer	Zhang	2014
**49**	Neurotization to innervate the deltoid and biceps: 3 cases	Dy	2013
**50**	Arthroscopic arthrodesis of the shoulder in brachial plexus palsy	Lenoir	2017
**51**	Outcome of contralateral C7 nerve transferring to median nerve	Gao	2013
**52**	Intercostal nerve transfer to the biceps motor branch in complete traumatic brachial plexus injuries	Cho	2015
**53**	Tactile feedback for relief of deafferentation pain using virtual reality system: a pilot study	Sano	2016
**54**	Functioning free gracilis transfer to reconstruct elbow flexion and quality of life in global brachial plexus injured patients	Yang	2016
**55**	Evaluation of infraspinatus reinnervation and function following spinal accessory nerve to suprascapular nerve transfer in adult traumatic brachial plexus injuries	Baltzer	2017
**56**	Anatomic study of the intercostal nerve transfer to the suprascapular nerve and a case report	Hu	2014
**57**	Shoulder abduction and external rotation restoration with nerve transfer	Kostas-Agnantis	2013
**58**	Contralateral C-7 transfer: is direct repair really superior to grafting?	Bhatia	2017
**59**	Impact of phrenic nerve paralysis on the surgical outcome of intercostal nerve transfer	Kita	2015
**60**	Flow-through anastomosis using a T-shaped vascular pedicle for gracilis functioning free muscle transplantation in brachial plexus injury	Hou	2015
**61**	Free functional muscle transfer tendon insertion secondary advancement procedure to improve elbow flexion	Sechachalam	2017

Article title: Developing a core outcome set for Traumatic Brachial Plexus Injuries: a systematic review of outcomes

Supplementary file 5. Included Studies

**62**	Dual nerve transfers for restoration of shoulder function after brachial plexus avulsion injury	Chu	2016
**63**	Cortical plasticity after brachial plexus injury and repair: a resting-state functional MRI study	Bhat	2017
**64**	Results of spinal accessory to suprascapular nerve transfer in 110 patients with complete palsy of the brachial plexus	Bertelli	2016
**65**	Magnetic resonance neurographic and clinical long-term results after oberlins transfer for adult brachial plexus injuries	Frueh	2017
**66**	Free functioning gracilis muscle transfer versus intercostal nerve transfer to musculocutaneous nerve for restoration of elbow flexion after traumatic adult brachial pan-plexus injury	Maldonado	2016
**67**	Results of wrist extension reconstruction in C5–8 brachial plexus palsy by transferring the pronator quadratus motor branch to the extensor carpi radialis brevis muscle	Bertelli	2016
**68**	Donor nerve sources in free functional gracilis muscle transfer for elbow flexion in adult brachial plexus injury	Nicoson	2017
**69**	Use of contralateral spinal accessory nerve for ipsilateral suprascapular neurotization in global brachial plexus injury: a new technique	Bhandari	2016
**70**	Objective evaluation of elbow flexion strength and fatigability after nerve transfer in adult traumatic brachial plexus injuries	Marciq	2014
**71**	Outcomes of muscle brachialis transfer to restore finger flexion in brachial plexus palsy	DeGeorge	2017
**72**	Functional outcome of nerve transfers for traumatic global brachial plexus avulsion	Liu	2013
**73**	Transfer of a flexor digitorum superficialis motor branch for wrist extension reconstruction in C5-C8 root injuries of the brachial plexus: a case series	Bertelli	2013
**74**	Outcome after transfer of intercostal nerves to the nerve of triceps long head in 25 adult patients with total plexus root avulsion injury	Gao	2013
**75**	Good sensory recovery of the hand in brachial plexus surgery using the intercostobrachial nerve as the donor	Foroni	2017
**76**	The phrenic nerve as a donor for brachial plexus injuries: is it safe and effective? Case series and literature analysis	Socolovsky	2015
**77**	Complete avulsion of brachial plexus with associated vascular trauma: Feasibility of reconstruction using the double free muscle technique	Hattori	2013
**78**	Long-term outcome of brachial plexus re-implantation after complete brachial plexus avulsion injury	Kachramanoglou	2017
**79**	Force recovery assessment of functioning free muscle transfers using ultrasonography	Kodama	2014
**80**	Rhomboid nerve transfer to the suprascapular nerve for shoulder reanimation in brachial plexus palsy: A clinical report	Goubier	2016
**81**	Outcome of contralateral C7 transfer to two recipient nerves in 22 patients with the total brachial plexus avulsion injury	Gao	2013
**82**	Comparative study of phrenic and intercostal nerve transfers for elbow flexion after global brachial plexus injury	Liu	2015

Article title: Developing a core outcome set for Traumatic Brachial Plexus Injuries: a systematic review of outcomes

Supplementary file 5. Included Studies

**83**	Donor-side morbidity after contralateral C-7 nerve transfer: results at a minimum of 6 months after surgery	Li	2016
**84**	Outcome after brachial plexus injury surgery and impact on quality of life	Rasulić	2017
**85**	Pronator teres branch transfer to the anterior interosseous nerve for treating C8T1 brachial plexus avulsion: An anatomic study and case report	Yang	2014
**86**	Operative treatment with nerve repair can restore function in patients with traction injuries in the brachial plexus	Stiasny	2015
**87**	Thoracodorsal nerve transfer for triceps reinnervation in partial brachial plexus injuries	Soldado	2016
**88**	Co-infusion of autologous adipose tissue derived neuronal differentiated mesenchymal stem cells and bone marrow derived hematopoietic stem cells, a viable therapy for post-traumatic brachial plexus injury: a case report	Thakkar	2014
**89**	Long-term clinical outcomes of spinal accessory nerve transfer to the suprascapular nerve in patients with brachial plexus palsy	Emamhadi	2016
**90**	Surgical treatment for total root avulsion type brachial plexus injuries by neurotisation: a prospective comparison study between total and hemicontralateral C7 nerve root transfer	Tu	2014
**91**	Deactivation of distant pain-related regions induced by 20-day rTMS: a case study of one-week pain relief for long-term intractable deafferentation pain	Qiu	2014
**92**	End-to-side neurorrhaphy in brachial plexus reconstruction	Haninec	2013
**93**	Reanimation of elbow extension with medial pectoral nerve transfer in partial injuries to the brachial plexus	Flores	2013
**94**	Early post-operative results after repair of traumatic brachial plexus palsy	Mohammad-Reda	2013
**95**	Satisfied patients after shoulder arthrodesis for brachial plexus lesions even after 20 years of follow-up	van der Lingen	2018
**96**	Posterior branch of the axillary nerve transfer to the lateral triceps branch for restoration of elbow extension: case report	Kilka	2013
**97**	Clinical analysis of repairing the whole brachial plexus nerve root avulsion by transferring C7 nerve root from the uninjured side	Liu	2014
**98**	Bipolar transfer of the pectoralis major muscle for restoration of elbow flexion in 29 cases	Cambon-Binder	2018
**99**	Thoracodorsal nerve transfer for elbow flexion reconstruction in infraclavicular brachial plexus injuries	Soldado	2014
**100**	Median nerve fascicle transfer versus ulnar nerve fascicle transfer to the biceps motor branch in C5-C6 and C5-C7 brachial plexus injuries: nonrandomised prospective study of 23 consecutive patients	Cho	2014
**101**	Free functional muscle transplantation of an anomalous femoral adductor with a very large muscle belly: a case report	Kaizawa	2013
**102**	Selective neurotisation of the radial nerve in the axilla using the intercostal nerve to treat complete brachial plexus palsy	Tuohuti	2016

Article title: Developing a core outcome set for Traumatic Brachial Plexus Injuries: a systematic review of outcomes

Supplementary file 5. Included Studies

**103**	Objective predictors of functional recovery associated with intercostal nerves transfer for triceps reinnervation in global brachial plexus palsy	Flores	2016
**104**	Nerve transfer to relieve pain in upper brachial plexus injuries: does it work?	Emamhadi	2017
**105**	Phrenic nerve transfer versus intercostal nerve transfer for the repair of brachial plexus root avulsion injuries	Abdixbir	2016
**106**	End-to-side neurorrhaphy to restore elbow flexion in brachial plexus injury	Limthongthang	2016
**107**	Chordata method combined with electrotherapy in functional recovery after brachial plexus injury:report of three clinical cases	De Oliveira	2016
**108**	Clinical outcome following transfer of the supinator motor branch to the posterior interosseous nerve in patients with C7-T1 brachial plexus palsy	Xu	2015
**109**	Transposition of branches of radial nerve innervating supinator to posterior interosseous nerve for functional reconstruction of finger and thumb extension in 4 patients with middle and lower trunk root avulsion injuries of brachial plexus	Wu	2017
**110**	Electromyographic findings in gracilis muscle grafts used to augment elbow flexion in traumatic brachial plexopathy	Kazamel	2016
**111**	Double distal intraneural fascicular nerve transfers for lower brachial plexus injuries	Li	2016
**112**	Restoration of elbow and hand function in total brachial plexus palsy with intercostal nerves and C5 root neurotisation. Results in 21 patients	Amal	2016
**113**	The phrenic nerve transfer in the treatment of a septuagenarian with brachial plexus avulsion injury: a case study	Jiang	2018
**114**	Outcomes of transferring a healthy motor fascicle from the radial nerve to a branch for the triceps to recover elbow extension in partial brachial plexus palsy	Flores	2017
**115**	Successful nerve transfers for traumatic brachial plexus palsy in a septuagenarian	Johnsen	2016
**116**	Free functioning gracilis muscle transfer for elbow flexion reconstruction after traumatic brachial pan-plexus injury: Where is the optimal distal tendon attachment for elbow flexion?	Maldonado	2017(b)
**117**	Results of distal nerve transfers in restoration of shoulder function in C5 and C6 root avulsion injury to the brachial plexus	Bhandari	2017
**118**	Bipolar dual-lead spinal cord stimulation between two electrodes on the ventral and dorsal sides of the spinal cord: consideration of putative mechanisms	Watanabe	2018
**119**	Triceps nerve to deltoid nerve transfer after an unsatisfactory intra-plexus neurotisation of the posterior division of the upper trunk	Al-Qattan	2017
**120**	Trapezius muscle transfer for restoration of elbow extension in a traumatic brachial plexus injury	Alrabai	2018
**121**	Transfer of the radial nerve branch to the extensor carpi radialis brevis to the anterior interosseous nerve to reconstruct thumb and finger flexion	Bertelli	2015

Supplementary file 5. Included Studies

**122**	Ultrasound-guided pulse-dose radiofrequency: treatment of neuropathic pain after brachial plexus lesion and arm vascularisation	Magistroni	2014
**123**	Phrenic nerve transfer to the musculocutaneous nerve for the repair of brachial plexus injury: electrophysiological characteristics	Liu	2015
**124**	Postoperative motor deficits following elbow flexion reanimation by nerve transfer	Hanneur	2018
**125**	Comparative study of phrenic and partial ulnar nerve transfers for elbow flexion after upper brachial plexus avulsion-a retrospective clinical analysis	Liu	2018
**126**	Contralateral medial pectoral nerve transfer with free gracilis muscle transfer in old brachial plexus injury	Yavari	2018
**127**	MEG-BMI to control phantom limb pain	Yanagisawa	2018
**128**	Complete brachial plexus injury- an amputation dilemma, A case report	Choong	2015
**129**	Reversal of phantom pain and hand-to-face remapping after brachial plexus avulsion	Tsao	2016
**130**	A newly developed upper limb single-joint HAL in a patient with elbow flexion reconstruction after traumatic brachial plexus injury: A case report	Kubota	2017
**131**	Free reverse gracilis muscle combined with steindler flexorplasty for elbow flexion reconstruction after failed primary repair of extended upper-type paralysis of the brachial plexus	Bertelli	2018
**132**	Multiple nerve and tendon transfers – a new strategy for restoring hand function in a patient with C7-T1 brachial plexus avulsions	Xu	2017
**Studies included following updated review May 2021**			
**133**	Outcomes after occupational therapy intervention for traumatic brachial plexus injury: A prospective longitudinal cohort study	Cole	2020
**134**	Lower trapezius transfer for patients with brachial plexus injury	Crepaldi	2019
**135**	Bionic upper limb reconstruction: A valuable alternative in global brachial plexus avulsion injuries—a case series	Hruby	2019
**136**	Transcranial Direct Current Stimulation and mirror therapy for neuropathic pain after brachial plexus avulsion: A randomised double-blind, controlled pilot study	Ferreira	2020
**137**	A comparative study of two modalities in pain management of patients presenting with chronic brachial neuralgia	Razak	2019
**138**	Do technical components of microanastomoses influence the functional outcome of free gracilis muscle transfer for elbow flexion in traumatic brachial plexus injury	Martins-Filho	2021

Article title: Developing a core outcome set for Traumatic Brachial Plexus Injuries: a systematic review of outcomes

Supplementary file 5. Included Studies

References

[1] Limthongthang R, Muennoi P, Phoojaroenchanachai R et al. Effectiveness and safety of home-based muscle electrical stimulator in brachial plexus injury patients. *J Med Assoc Thail* 2014;97:S56–61.

[2] Homsreprasert T, Limthongthang R, Vathana T et al. Elbow joint proprioceptive sense in total arm-type brachial plexus injured patients after neurotization: a preliminary study. *J Med Assoc Thail* 2014;97:S103–7.

[3] Souza F, Bernardino S, Filho H et al. Comparison between the anterior and posterior approach for transfer of the spinal accessory nerve to the suprascapular nerve in late traumatic brachial plexus injuries. *Acta Neurochir (Wien)* 2014;156: 2345–9.

[4] Kim JH, Shin SH, Lee YR et al. Ultrasound-guided peripheral nerve stimulation for neuropathic pain after brachial plexus injury: two case reports. *J Anesth* 2017; 31:453–7.

[5] Satbhai NG, Doi K, Hattori Y, Sakamoto S et al. Contralateral lower trapezius transfer for restoration of shoulder external rotation in traumatic brachial plexus palsy: a preliminary report and literature review. *J Hand Surg Eur* 2014;39:861–7.

[6] Huan KWSJ, Tan JSW, Tan SH et al. Restoration of shoulder abduction in brachial plexus avulsion injuries with double neurotization from the spinal accessory nerve: a report of 13 cases. *J Hand Surg Eur* 2017;42:700–5.

[7] Bertelli JA, Soldado F, Ghizoni MF et al. Transfer of the musculocutaneous nerve branch to the brachialis muscle to the triceps for elbow extension: anatomical study and report of five cases. *J Hand Surg Eur* 2017;42:710–4.

[8] Rui J, Zhao X, Zhu Y et al. Posterior approach for accessory-suprascapular nerve transfer: an electrophysiological outcomes study. *J Hand Surg Eur* 2013;38:242–7.

[9] Potter SM, Ferris SI. Reliability of functioning free muscle transfer and vascularized ulnar nerve grafting for elbow flexion in complete brachial plexus palsy. *J Hand Surg Eur* 2017; 42: 693–9.

[10] Lam WL, Fufa D, Chang N-J et al. Management of infraclavicular (Chuang Level IV) brachial plexus injuries: A single surgeon experience with 75 cases. *J Hand Surg Eur* 2015; 40:573–82.

[11] Estrella EP, Montales TD. Functioning free muscle transfer for the restoration of elbow flexion in brachial plexus injury patients. *Injury* 2016;47:2525–33.

[12] Desai MJ, Daly CA, Seiler JG et al. Radial to Axillary Nerve Transfers: A Combined Case Series. *J Hand Surg Am* 2016; 41:1128–34.

[13] Pereira EAC, Boccard SG, Linhares P et al. Thalamic deep brain stimulation for neuropathic pain after amputation or brachial plexus avulsion. *Neurosurg Focus* 2013;35:E7.

[14] Estrella EP, Favila AS . Nerve transfers for shoulder function for traumatic brachial plexus injuries. *J Reconstr Microsurg* 2014;30:59-64.

[15] Gutkowska O, Martynkiewicz J, Mizia S et al. Results of Operative Treatment of Brachial Plexus Injury Resulting from Shoulder Dislocation: A Study with A Long-Term Follow-Up. *World Neurosurg* 2017;105:623–31.

[16] Oberlin C, Chino J, Belkheyar Z. Surgical treatment of brachial plexus posterior cord lesion: A combination of nerve and tendon transfers, about nine patients. *Chir Main* 2013;32:141–6.

Supplementary file 5. Included Studies

[17] Ferraresi S, Garozzo D, Basso E et al. The medial cord to musculocutaneous (MCMc) nerve transfer: A new method to reanimate elbow flexion after C5-C6-C7-(C8) avulsive injuries of the brachial plexus - Technique and results. *Neurosurg Rev* 2014;37:321–9.

[18] Bertelli J, Soldado F, Ghizoni MF et al. Transfer of a terminal motor branch nerve to the flexor carpi ulnaris for triceps reinnervation: Anatomical study and clinical cases. *J Hand Surg Am* 2015;40:2229-2235.

[19] Maldonado AA, Kircher MF, Spinner RJ et al. Free Functioning Gracilis Muscle Transfer With and Without Simultaneous Intercostal Nerve Transfer to Musculocutaneous Nerve for Restoration of Elbow Flexion After Traumatic Adult Brachial Pan-Plexus Injury. *J Hand Surg Am* 2017;42:293.

[20] Ghosh S, Singh VK, Jeyaseelan L, et al. Isolated latissimus dorsi transfer to restore shoulder external rotation in adults with brachial plexus injury. *Bone Jt J* 2013;95:660–3.

[21] Satbhai NG, Doi K, Hattori Y et al. Functional outcome and quality of life after traumatic total brachial plexus injury treated by nerve transfer or single/double free muscle transfers: A comparative study. *Bone Jt J* 2016;98:209–17.

[22] Mibu A, Nishigami T, Tanaka K et al. Successful Graded Mirror Therapy in a Patient with Chronic Deafferentation Pain in Whom Traditional Mirror Therapy was Ineffective: A Case Report. *Pain Pract* 2016;16:E62–9.

[23] Azab AA-H, Alsabbahi MS. Bipolar Transfer of Latissimus Dorsi Myocutaneous Flap for Restoration of Elbow Flexion in Late Traumatic Brachial Plexus Injury: Evaluation of 13 Cases. *Ann Plast Surg* 2017;78:98–201.

[24] Tsai YJ, Su FC, Hsiao CK et al. Comparison of objective muscle strength in C5-C6 and C5-C7 brachial plexus injury patients after double nerve transfer. *Microsurgery* 2015;35:107–14.

[25] Son BC, Ha SW. Phantom Remodeling Effect of Dorsal Root Entry Zone Lesioning in Phantom Limb Pain Caused by Brachial Plexus Avulsion. *Stereotact Funct Neurosurg* 2015;93:240–4.

[26] Hu CH, Chang TN, Lu JC et al. Comparison of Surgical Strategies between Proximal Nerve Graft and/or Nerve Transfer and Distal Nerve Transfer Based on Functional Restoration of Elbow Flexion: A Retrospective Review of 147 Patients. *Plast Reconstr Surg* 2018;141:68e-79e.

[27] Ren G, Li R, Xiang D, Yu B. Reconstruction of shoulder abduction by multiple nerve fascicle transfer through posterior approach. *Injury* 2013;44:492–7.

[28] Xiao C, Lao J, Wang T et al. Intercostal nerve transfer to neurotize the musculocutaneous nerve after traumatic brachial plexus avulsion: a comparison of two, three, and four nerve transfers. *J Reconstr Microsurg* 2014;30:297–304.

[29] Resnik L, Fantini C, Latlief G et al. Use of the DEKA Arm for amputees with brachial plexus injury: A case series. *PLoS One* 2017; 12: e0178642.

[30] Leechavengvongs S, Jiamton C, Uerpairojkit C et al. Polyester tape scapulopexy for chronic upper extremity brachial plexus injury. *J Hand Surg Am* 2015;40:1184

[31] Wang S, Li P, Xue Y et al. Contralateral C7 nerve transfer with direct coaptation to restore lower trunk function after traumatic brachial plexus avulsion. *J Bone Joint Surg Am* 2013;95: 821–2.

[32] Dodakundi C, Doi K, Hattori Y et al. Outcome of surgical reconstruction after traumatic total brachial plexus palsy. *J Bone Joint Surg Am* 2013;95:1505–12.

Article title: Developing a core outcome set for Traumatic Brachial Plexus Injuries: a systematic review of outcomes

Supplementary file 5. Included Studies

[33] Aszmann OC, Roche AD, Salminger S et al. Bionic reconstruction to restore hand function after brachial plexus injury: a case series of three patients. *Lancet* 2015; 385:2183–9.

[34] Tsymbaliuk VI, Tretiak IB. [Surgical treatment of the plexus brachialis injury using long-lasting electrostimulation]. *Klin Khirurhiia* 2013;59–61.

[35] Flores LP, Socolovsky M. Phrenic Nerve Transfer for Reconstruction of Elbow Extension in Severe Brachial Plexus Injuries. *J Reconstr Microsurg* 2016;32:546–50.

[36] Wang S, Li P, Xue Y et al. Direct Coaptation of the Phrenic Nerve With the Posterior Division of the Lower Trunk to Restore Finger and Elbow Extension Function in Patients With Total Brachial Plexus Injuries. *Neurosurgery* 2016;78:208–14.

[37] Martins RS, Siqueira MG, Heise CO et al. A prospective study comparing single and double fascicular transfer to restore elbow flexion after brachial plexus injury. *Neurosurgery* 2013; 72:709–15.

[38] Stevanato G, Devigili G, Eleopra R et al. Chronic post-traumatic neuropathic pain of brachial plexus and upper limb: a new technique of peripheral nerve stimulation. *Neurosurg Rev* 2014;37:473–9.

[39] Wei W, Meihua S, Yafei L et al. [EFFECTIVENESS OF CONTRALATERAL C7 NERVE ROOT AND MULTIPLE NERVES TRANSFER FOR TREATMENT OF BRACHIAL PLEXUS ROOT AVULSION]. *Zhongguo Xiu Fu Chong Jian Wai Ke Za Zhi* 2014;28:737–40.

[40] Plate JF, Ely LK, Pulley BR et al. Combined proximal nerve graft and distal nerve transfer for a posterior cord brachial plexus injury. *J Neurosurg* 2013;118: 155–9.

[41] Maldonado AA, Kircher MF, Spinner RJ et al. The role of elective amputation in patients with traumatic brachial plexus injury. *J Plast Reconstr Aesthetic Surg* 2016;69:311–7.

[42] Liu Y, Wang W, Regmi AM et al. Early microsurgical management of clavicular fracture combined with brachial plexus injury. *Zhongguo Xiu Fu Chong Jian Wai Ke Za Zhi* 2014;28:1329–32.

[43] Elhassan BT, Wagner ER, Spinner RJ et al. Contralateral trapezius transfer to restore shoulder external rotation following adult brachial plexus injury. *J Hand Surg Am* 2016;41:e45-51.

[44] Liu Y, Lao J, Gao K et al. Comparative study of phrenic nerve transfers with and without nerve graft for elbow flexion after global brachial plexus injury. *Injury* 2014;45:227–31.

[45] Wang JP, Rancy SK, Lee SK et al. Shoulder and Elbow Recovery at 2 and 11 Years Following Brachial Plexus Reconstruction. *J Hand Surg Am* 2016;41:173–9.

[46] Aras Y, Aydoseli A, Sabanci PA et al. Functional outcomes after treatment of traumatic brachial plexus injuries: clinical study. *Ulus Travma Acil Cerrahi Derg* 2013;19:521–8.

[47] Soldado F, Bertelli J. Free gracilis transfer reinnervated by the nerve to the supinator for the reconstruction of finger and thumb extension in longstanding C7-T1 brachial plexus root avulsion. *J Hand Surg Am* 2013;38: 941–6.

[48] Zhang CG, Dong Z, Gu YD. Restoration of hand function in C7-T1 brachial plexus palsies using a staged approach with nerve and tendon transfer. *J Neurosurg* 2014;121:1264–70.

[49] Dy CJ, Kitay A, Garg R et al. Neurotization to innervate the deltoid and biceps: 3 Cases. *J Hand Surg Am* 2013;38:237–40.

[50] Lenoir H, Williams T, Griffart A et al. Arthroscopic arthrodesis of the shoulder in brachial

Article title: Developing a core outcome set for Traumatic Brachial Plexus Injuries: a systematic review of outcomes

Supplementary file 5. Included Studies

plexus palsy. *J Shoulder Elb Surg* 2017; 26: e115–21.

[51] Gao K, Lao J, Zhao X et al. Outcome of contralateral C7 nerve transferring to median nerve. *Chin Med J (Engl)* 2013;126:3865–8.

[52] Cho AB, Iamaguchi RB, Silva GB et al. Intercostal nerve transfer to the biceps motor branch in complete traumatic brachial plexus injuries. *Microsurgery* 2015;35:428–31.

[53] Sano Y, Wake N, Ichinose A et al. Tactile feedback for relief of deafferentation pain using virtual reality system: a pilot study. *J Neuroeng Rehabil* 2016;13:61.

[54] Yang Y, Yang JT, Fu G et al. Functioning free gracilis transfer to reconstruct elbow flexion and quality of life in global brachial plexus injured patients. *Sci Rep* 2016;6:22479.

[55] Baltzer HL, Wagner ER, Kircher MF, et al. Evaluation of infraspinatus reinnervation and function following spinal accessory nerve to suprascapular nerve transfer in adult traumatic brachial plexus injuries. *Microsurgery* 2017;37:365–70.

[56] Hu S, Chu B, Song J, et al. Anatomic study of the intercostal nerve transfer to the suprascapular nerve and a case report. *J Hand Surg Eur* 2014;39:194–8.

[57] Kostas-Agnantis I, Korompilias A, Vekris M et al. Shoulder abduction and external rotation restoration with nerve transfer. *Injury* 2013;44:299–304.

[58] Bhatia A, Doshi P, Koul A, et al. Contralateral C-7 transfer: is direct repair really superior to grafting? *Neurosurg Focus* 2017;43:E3.

[59] Kita Y, Tajiri Y, Hoshikawa S et al. Impact of phrenic nerve paralysis on the surgical outcome of intercostal nerve transfer. *Hand Surg* 2015; 20:47–52.

[60] Hou Y, Yang J, Yang Y, et al. Flow-through anastomosis using a T-shaped vascular pedicle for gracilis functioning free muscle transplantation in brachial plexus injury. *Clinics (Sao Paulo*) 2015;70:544–9.

[61] Sechachalam S, O'Byrne A, MacQuillan A. Free Functional Muscle Transfer Tendon Insertion Secondary Advancement Procedure to Improve Elbow Flexion. *Tech Hand Up Extrem Surg* 2017;21: 8–12.

[62] Chu B, Wang H, Chen L, et al. Dual Nerve Transfers for Restoration of Shoulder Function After Brachial Plexus Avulsion Injury. *Ann Plast Surg* 2016;76: 668–73.

[63] Bhat DI, Indira Devi B, Bharti K, et al. Cortical plasticity after brachial plexus injury and repair: a resting-state functional MRI study. *Neurosurg Focus* 2017;42: E14.

[64] Bertelli JA, Ghizoni MF. Results of spinal accessory to suprascapular nerve transfer in 110 patients with complete palsy of the brachial plexus. *J Neurosurg Spine* 2016;24:990–5.

[65] Frueh FS, Ho M, Schiller A, et al. Magnetic Resonance Neurographic and Clinical Long-Term Results After Oberlin's Transfer for Adult Brachial Plexus Injuries. *Ann Plast Surg* 2017;78:67–72.

[66] Maldonado AA, Kircher MF, Spinner RJ,et al. Free Functioning Gracilis Muscle Transfer versus Intercostal Nerve Transfer to Musculocutaneous Nerve for Restoration of Elbow Flexion after Traumatic Adult Brachial Pan-Plexus Injury. *Plast Reconstr Surg* 2016;138:483e-488e.

[67] Bertelli JA, Ghizoni MF, Tacca CP. Results of wrist extension reconstruction in C5-8 brachial plexus palsy by transferring the pronator quadratus motor branch to the extensor carpi radialis brevis muscle. *J Neurosurg* 2016;124:1442–9.

Article title: Developing a core outcome set for Traumatic Brachial Plexus Injuries: a systematic review of outcomes

Supplementary file 5. Included Studies

[68] Nicoson MC, Franco MJ, Tung TH. Donor nerve sources in free functional gracilis muscle transfer for elbow flexion in adult brachial plexus injury. *Microsurgery* 2017;37:377–82.

[69] Bhandari PS, Deb P. Use of contralateral spinal accessory nerve for ipsilateral suprascapular neurotization in global brachial plexus injury: A new technique. *J Neurosurg Spine* 2016;24: 186–8.

[70] Maricq C, Jeunehomme M, Mouraux D, et al. Objective evaluation of elbow flexion strength and fatigability after nerve transfer in adult traumatic upper brachial plexus injuries. *Hand Surg* 2014;19:335–41.

[71] DeGeorge B, Becker H, Faryna J,et al. Outcomes of brachialis muscle transfer to restore finger flexion in traumatic lower trunk brachial plexus palsy. *J Hand Surg Am* 2017;42:S33–4.

[72] Liu Y, Lao J, Gao K, et al. Functional outcome of nerve transfers for traumatic global brachial plexus avulsion. *Injury* 2013;44:655–60.

[73] Bertelli JA, Ghizoni MF. Transfer of a flexor digitorum superficialis motor branch for wrist extension reconstruction in C5-C8 root injuries of the brachial plexus: a case series. *Microsurgery* 2013;33:39–42.

[74] Gao K, Lao J, Zhao X,et al. Outcome after transfer of intercostal nerves to the nerve of triceps long head in 25 adult patients with total brachial plexus root avulsion injury. *J Neurosurg* 2013;118:606–10.

[75] Foroni L, Siqueira MG, Martins RS, et al. Good sensory recovery of the hand in brachial plexus surgery using the intercostobrachial nerve as the donor. *Arq Neuropsiquiatr* 2017;75:796–800.

[76] Socolovsky M, di Masi G, Bonilla G, et al. The phrenic nerve as a donor for brachial plexus injuries: is it safe and effective? Case series and literature analysis. *Acta Neurochir (Wien)* 2015;157:1077–86.

[77] Hattori Y, Doi K, Sakamoto S,et al. Complete avulsion of brachial plexus with associated vascular trauma: feasibility of reconstruction using the double free muscle technique. *Plast Reconstr Surg* 2013;132:1504–12.

[78] Kachramanoglou C, Carlstedt T, Koltzenburg M, et al. Long-Term Outcome of Brachial Plexus Reimplantation After Complete Brachial Plexus Avulsion Injury. *World Neurosurg* 2017;103:28–36.

[79] Kodama N, Doi K, Hattori Y. Force recovery assessment of functioning free muscle transfers using ultrasonography. *J Hand Surg Am* 2014;39:2269–76.

[80] Goubier J, Teboul F. Rhomboid nerve transfer to the suprascapular nerve for shoulder reanimation in brachial plexus palsy: A clinical report. *Hand Surg Rehabil* 2016;35:363–6.

[81] Gao K, Lao J, Zhao X,et al. Outcome of contralateral C7 transfer to two recipient nerves in 22 patients with the total brachial plexus avulsion injury. *Microsurgery* 2013;33:605–11.

[82] Liu Y, Lao J, Zhao X. Comparative study of phrenic and intercostal nerve transfers for elbow flexion after global brachial plexus injury. *Injury* 2015;46:671–5.

[83] Li XM, Yang JT, Hou Y, et al. Donor-side morbidity after contralateral C-7 nerve transfer: results at a minimum of 6 months after surgery. *J Neurosurg* 2016;124:1434–41

[84] Rasulic L, Savic A, Zivkovic B, et al. Outcome after brachial plexus injury surgery and impact on

Article title: Developing a core outcome set for Traumatic Brachial Plexus Injuries: a systematic review of outcomes

Supplementary file 5. Included Studies

quality of life. *Acta Neurochir (Wien)* 2017;159:1257–64

[85] Yang J, Jia X, Yu C, et al. Pronator teres branch transfer to the anterior interosseous nerve for treating C8T1 brachial plexus avulsion: An anatomic study and case report. *Neurosurgery* 2014;75:375–9.

[86] Stiasny J, Birkeland P, J. S. Operative treatment with nerve repair can restore function in patients with traction injuries in the brachial plexus. *Dan Med J* 2015;62.

[87] Soldado F, Ghizoni MF, Bertelli J, et al. Thoracodorsal nerve transfer for triceps reinnervation in partial brachial plexus injuries. *Microsurgery* 2016;36:191–7.

[88] Thakkar UG, Vanikar A V, Trivedi HL. Co-infusion of autologous adipose tissue derived neuronal differentiated mesenchymal stem cells and bone marrow derived hematopoietic stem cells, a viable therapy for post-traumatic brachial plexus injury: a case report. *Biomed J* 2014;37:237–40.

[89] Emamhadi M, Alijani B, Andalib S,et al. Long-term clinical outcomes of spinal accessory nerve transfer to the suprascapular nerve in patients with brachial plexus palsy. *Acta Neurochir (Wien)* 2016;158:1801–6.

[90] Tu YK, Tsai YJ, Chang CH et al. Surgical treatment for total root avulsion type brachial plexus injuries by neurotization: a prospective comparison study between total and hemicontralateral C7 nerve root transfer. *Microsurgery* 2014;34:91–101.

[91] Qiu YQ, Hua XY, Zuo CT et al. Deactivation of distant pain-related regions induced by 20-day rTMS: a case study of one-week pain relief for long-term intractable deafferentation pain. *Pain Physician* 2014;17:E99–105.

[92] Haninec P, Mencl L, Kaiser R. End-to-side neurorrhaphy in brachial plexus reconstruction. *J Neurosurg* 2013;119:689–94.

[93] Flores LP. Reanimation of elbow extension with medial pectoral nerve transfer in partial injuries to the brachial plexus. *J Neurosurg* 2013;118:588–93.

[94] Mohammad-Reda A. Early post-operative results after repair of traumatic brachial plexus palsy. *Turk Neurosurg* 2013;23:1–9.

[95] van der Lingen MAJ, de Joode SGCJ, Schotanus MGM, et al. Satisfied patients after shoulder arthrodesis for brachial plexus lesions even after 20 years of follow-up. *Eur J Orthop Surg Traumatol* 2018;28:1089–94

[96] Klika BJ, Spinner RJ, Bishop AT et al. Posterior branch of the axillary nerve transfer to the lateral triceps branch for restoration of elbow extension: case report. *J Hand Surg Am* 2013;38:1145–9.

[97] Liu J, Wang X, Zhang S, et al. A clinical analysis of repairing the whole brachial plexus nerve root avulsion by transferring C7 nerve root from the uninjured side. *J Neurol Sci* 2014;31:521–31.

[98] Cambon-Binder A, Walch A, Marchei P et al. Bipolar transfer of the pectoralis major muscle for restoration of elbow flexion in 29 cases. *J Shoulder Elb Surg* 2018;27:e330-e336

[99] Soldado F, Ghizoni MF, Bertelli J. Thoracodorsal nerve transfer for elbow flexion reconstruction in infraclavicular brachial plexus injuries. *J Hand Surg Am* 2014;39:1766–70.

[100] Cho A, Paulos R, De Resende M, et al. Median nerve fascicle transfer versus ulnar nerve

Article title: Developing a core outcome set for Traumatic Brachial Plexus Injuries: a systematic review of outcomes

Supplementary file 5. Included Studies

fascicle transfer to the biceps motor branch in C5-C6 and C5-C7 brachial plexus injuries: nonrandomized prospective study of 23 consecutive patients. *Microsurgery* 2014;34:511–5.

[101] Kaizawa Y, Kakinoki R, Ohta S,et al. Free functional muscle transplantation of an anomalous femoral adductor with a very large muscle belly: A case report. *J Brachial Plex Peripher Nerve* Inj 2013;8:11.

[102] Tuohuti T, Yu Q, Yang J,et al. Selective neurotization of the radial nerve in the axilla using intercostal nerve to treat complete brachial plexus palsy. *Int J Clin Exp Med* 2016;9:22880–5.

[103] Flores LP. Objective Predictors of Functional Recovery Associated with Intercostal Nerves Transfer for Triceps Reinnervation in Global Brachial Plexus Palsy. *Brazilian Neurosurg* 2016;35:271–8.

[104] Emamhadi M. Nerve transfer to relieve pain in upper brachial plexus injuries: Does it work? *Clin Neurol Neurosurg* 2017;163:67–70.

[105] Abdixbir A, Li P, Ilhamjan U, et al. Phrenic nerve transfer versus intercostal nerve transfer for the repair of brachial plexus root avulsion injuries. *Chinese J Tissue Eng Res* 2016;20:7660–5.

[106] Limthongthang R, Vathana T, Wongtrakul S,et al. End-to-Side Neurorrhaphy to Restore Elbow Flexion in Brachial Plexus Injury. *J Med Assoc Thai* 2016;99:1203–8.

[107] De Oliveira C, Da Silva Melo D, et al. Chordata method combined with electrotherapy in functional recovery after brachial plexus injury: Report of three clinical cases. *Sci Med (Porto Alegre)* 2016;26:22425.

[108] Xu B, Dong Z, Zhang CG,et al. Clinical outcome following transfer of the supinator motor branch to the posterior interosseous nerve in patients with C7-T1 brachial plexus palsy. *J Reconstr Microsurg* 2015;31:102–6.

[109] Wu X, Cong XB, Huang QS, et al. Transposition of branches of radial nerve innervating supinator to posterior interosseous nerve for functional reconstruction of finger and thumb extension in 4 patients with middle and lower trunk root avulsion injuries of brachial plexus. *J Huazhong Univ Sci Technolog Med Sci* 2017;37:933–7.

[110] Kazamel M, Sorenson EJ. Electromyographic Findings in Gracilis Muscle Grafts Used to Augment Elbow Flexion in Traumatic Brachial Plexopathy. *J Clin Neurophysiol* 2016;33:549–53.

[111] Li Z, Reynolds M, Satteson E,et al. Double Distal Intraneural Fascicular Nerve Transfers for Lower Brachial Plexus Injuries. *J Hand Surg Am* 2016;41:e15-9.

[112] Arnal M, Cambon A, Marcheix P. Restoration of elbow and hand function in total brachial plexus palsy with intercostal nerves and C5 root neurotization. Results in 21 patients. *Hand Surg Rehabil* 2016;35:283–7.

[113] Jiang Y, Lao J. The phrenic nerve transfer in the treatment of a septuagenarian with brachial plexus avulsion injury: a case report. *Int J Neurosci* 2018;128:467–71.

[114] Flores LP. Outcomes of Transferring a Healthy Motor Fascicle From the Radial Nerve to a Branch for the Triceps to Recover Elbow Extension in Partial Brachial Plexus Palsy. *Neurosurgery* 2017;80:448–53.

[115] Johnsen PH, Wolfe SW. Successful Nerve Transfers for Traumatic Brachial Plexus Palsy in a Septuagenarian: A Case Report. *Hand (N Y)* 2016;11:NP30–3.

Article title: Developing a core outcome set for Traumatic Brachial Plexus Injuries: a systematic review of outcomes

Supplementary file 5. Included Studies

[116] Maldonado AA, Romero-Brufau S, Kircher MF, et al. Free Functioning Gracilis Muscle Transfer for Elbow Flexion Reconstruction after Traumatic Adult Brachial Pan-Plexus Injury: Where Is the Optimal Distal Tendon Attachment for Elbow Flexion? *Plast Reconstr Surg* 2017;139: 128–36.

[117] Bhandari PS. Results of Distal Nerve Transfers in Restoration of Shoulder Function in C5 and C6 Root Avulsion Injury to the Brachial Plexus. *Indian J Neurotrauma* 2017;14:21–5.

[118] Watanabe M, Yamamoto T, Fukaya C, et al. Bipolar dual-lead spinal cord stimulation between two electrodes on the ventral and dorsal sides of the spinal cord: consideration of putative mechanisms. *Acta Neurochir (Wien)* 2018;160:639–43.

[119] Al-Qattan M, Kattan A, Al-Qahtany B et al. Triceps nerve to deltoid nerve transfer after an unsatisfactory intra-plexus neurotization of the posterior division of the upper trunk. *Int J Surg Case Rep* 2017;37:124–6.

[120] Alrabai H, Gesheff G, Hammouda A,et al. Trapezius Muscle Transfer for Restoration of Elbow Extension in a Traumatic Brachial Plexus Injury. *J Hand Surg Am* 2018;43:872.

[121] Bertelli JA. Transfer of the radial nerve branch to the extensor carpi radialis brevis to the anterior interosseous nerve to reconstruct thumb and finger flexion. *J Hand Surg Am* 2015;40:323-328.

[122] Magistroni E, Ciclamini D, Panero B. Ultrasound-guided pulse-dose radiofrequency: Treatment of neuropathic pain after brachial plexus lesion and arm revascularization. *Case Rep Med* 2014;201:429618.

[123] Liu Y, Xu X, Zou Y, et al. Phrenic nerve transfer to the musculocutaneous nerve for the repair of brachial plexus injury: Electrophysiological characteristics. *Neural Regen Res* 2015;10:328–33.

[124] Le Hanneur M, Walch A, Gerosa T et al. Postoperative motor deficits following elbow flexion reanimation by nerve transfer. *Hand Surg Rehabil* 2018;37:289-294

[125] Liu Y, Zhuang Y, Yu H,et al. Comparative study of phrenic and partial ulnar nerve transfers for elbow flexion after upper brachial plexus avulsion: A retrospective clinical analysis. *J Plast Reconstr Aesthetic Surg* 2018;71:1245–51.

[126] Yavari M, Mahmoudvand H, Nadri S. Contralateral medial pectoral nerve transfer with free gracilis muscle transfer in old brachial plexus palsy. *J Surg Res* 2018;231:94–8.

[127] Yanagisawa T, Fukuma R, Seymour B, et al. MEG-BMI to control phantom limb pain. *Neurol Med Chir (Tokyo)* 2018;58:327–33.

[128] Choong C, Shalimar A. Complete brachial plexus injury - An amputation dilemma. A case report. *Malaysian Orthop J* 2015;9:52–4.

[129] Tsao J, Finn S. Reversal of phantom pain and hand-to-face remapping after brachial plexus avulsion. *Ann Clin Transl Neurol* 2016;3:463–4..

[130] Kubota S, Hara Y, Shimizu Y, et al. A newly developed upper limb single-joint HAL in a patient with elbow flexion reconstruction after traumatic brachial plexus injury: A case report. *Interdiscip Neurosurg Adv Tech Case Manag* 2017;10:66–8.

[131] Bertelli JA. Free Reverse Gracilis Muscle Combined With Steindler Flexorplasty for Elbow Flexion Reconstruction After Failed Primary Repair of Extended Upper-Type Paralysis of the Brachial Plexus. *J Hand Surg Am* 2019;44:112-120

Article title: Developing a core outcome set for Traumatic Brachial Plexus Injuries: a systematic review of outcomes

Supplementary file 5. Included Studies

[132] Xu B, Dong Z, Zhang C. Multiple nerve and tendon transfers: A new strategy for restoring hand function in a patient with C7-T1 brachial plexus avulsions. *J Neurosurg* 2017;127:837–42.

[133] Cole T, Nicks R, Ferris S et al. Outcomes after occupational therapy intervention for traumatic brachial plexus injury: A prospective longitudinal cohort study. *Journal of Hand Therapy*. 2020;33:528-539

[134] Crepaldi BE, Neto JQL, Rezende MR et al. *Hand*.2019;14(2):179-186

[135] Hruby LA, Gstoettner C, Sturma A et al. *Journal of Clinical Medicine*. 2020;9(23):1-14

[136] Ferreira CM, de Carvalho CD, Gomes R et al. Transcranial direct current stimulation and mirror therapy for neuropathic pain after brachial plexus avulsion: A randomised double-blind, controlled pilot study. Frontiers in *Neurology*. 2020;11:1-10

[137] Razak I, Chung TY, Ahmad S et al. A comparative study of two modalities in pain managementof patients presenting with chronic brachial neuralgia*. Journal of Alternative and Complementary Medicine.* 2019;1-7

[138] Martins-Filho FVF, de Carmo Iwase F, Silva GB et al. Do technical components of microanastomosis influence the functional outcome of free gracilis muscle transfer for elbow flexion in traumatic brachial plexus injury? *Orthopaedics & Traumatology: Surgery & Research.*2021;107:1-5

Article title: Developing a core outcome set for Traumatic Brachial Plexus Injuries: a systematic review of outcomes
