# Supplementary material for: Developing a core outcome set for traumatic brachial plexus injuries: a systematic review of outcomes
Source: BMJ Open. 2021 Jul 30;11(7):e044797. doi: 10.1136/bmjopen-2020-044797 (PMC8327802; doi:10.1136/bmjopen-2020-044797)
Supplement: Supplementary data [file bmjopen-2020-044797supp006.pdf]

Supplementary file 6: Unique outcomes mapped to potential domains and core areas according to COMET

Online Supplementary file 4. Table: Unique outcomes mapped to potential domains and core areas according to COMET(Dodd et al., 2018)

Outcomes (n=157)	Subdomains	Domains	Core Areas
Isometric muscle strength	Muscle strength/ function	Musculoskeletal and connective tissue domain	Physiological/Clinical
Concentric strength			
Eccentric strength			
Muscle flicker/contraction			
Anti-gravity muscle activity			
Muscle endurance			
Muscle fatigue			
Muscle torque			
Active range of movement	Active movement		
Perception of movement			
Antigravity movement			
Independent movement without donor			
Passive range of movement	Passive movement		
Movement control/stability	Control of movement/stability		
Muscle mass	Muscle mass		
Bony union	Bone structure/position		
Joint position			
Joint stability			
General sensory recovery	General sensory recovery	Nervous system	
Feeling of numbness			
Proprioception			
Light touch	Discriminative touch		
2 PD			

Article title: Developing a core outcome set for Traumatic Brachial Plexus Injuries: a systematic review of outcomes

Supplementary file 6: Unique outcomes mapped to potential domains and core areas according to COMET

Vibration			
Object recognition			
Pain	Protective touch		
Temperature			
Deep pressure			
Brachial plexus structure	Peripheral nervous system structure		
Level of reinnervation	Reinnervation		
Time to reinnervation			
Progression of regeneration	Progression of regeneration		
Speed of motor sensory conduction	Speed of motor and sensory conduction		
Pain intensity	Pain intensity/relief	General outcomes/symptoms	
Pain relief / reduction			
Pain duration	Pain duration/frequency		
Pain frequency			
Pain quality	Pain quality and interference with life		
Pain interference with walking			
Pain interference in mood			
Pain interference with work			
Pain interference in activities of daily living			
Pain interference with relationships			
Pain interference with enjoyment of life			
Pain interference with sleep			
Sensitivity to cold	Pain when arm exposed to cold		
Paraesthesia	Paraesthesia and itchiness		
Itchiness			

Article title: Developing a core outcome set for Traumatic Brachial Plexus Injuries: a systematic review of outcomes

Supplementary file 6: Unique outcomes mapped to potential domains and core areas according to COMET

Sensitivity to pressure	Sensitivity to touch, pressure etc		
Sensitivity to touch			
Pain location	Location of pain		
Pain relief from medication	Pain medication use		
Stiffness	Stiffness		
Impact on general sleep	Impact on sleep		
Impact on sleep on affected side			
Frequency sleep disturbed by injury			
General physical function	Physical function non-specific	Physical functioning	Life Impact
Patient led functional outcome			
Walking short distance	Lower limb and non -upper limb function		
Balance			
Running			
Climbing stairs			
Bending			
Kneeling			
Reaching	Reaching, pulling, pushing, carrying etc		
Pulling			
Pushing			
Carrying			
Throwing			
Lifting			
General function of arm			
Turning and twisting arm	Turning twisting, gripping and releasing with the arm		
Grip and release			
Pinching	Fine hand movement including writing		
Fine hand movement (writing/buttons)			
Returning to work	Impact on paid or unpaid work or role in education	Role functioning	
Ability to do work			
Usual time at work			
Type of work			

Article title: Developing a core outcome set for Traumatic Brachial Plexus Injuries: a systematic review of outcomes

Miller C, *et al. BMJ Open* 2021; 11:e044797. doi: 10.1136/bmjopen-2020-044797

Supplementary file 6: Unique outcomes mapped to potential domains and core areas according to COMET

Usual school activities			
General rating to perform a patient specific activity	Role function - patient specific		
Impact on ADL (general)	Carrying out daily routine, (including food preparation, housework, garden, plants)		
Return to ADL (general)			
Impact on food preparation and feeding			
Housework (washing, cleaning, ironing, folding, vacuuming)			
Gardening (Includes indoor plants)			
Using a phone			
Maintaining personal hygiene			
Maintaining personal appearance (grooming hair)	Maintaining personal hygiene		
Dressing	Maintaining personal appearance		
Transport needs (e.g driving)	Dressing		
Impact on normal hobbies	Transport needs		
Time doing normal hobbies	Impact on recreational activities and sport		
Playing instrument in usual way			
Ability to play instrument			
Impact on time spent playing instrument			
Impact on time spent doing sport			
Impact on participation in sport			
Social activities with friends	Effect on relationship with	Social functioning	

Article title: Developing a core outcome set for Traumatic Brachial Plexus Injuries: a systematic review of outcomes

Supplementary file 6: Unique outcomes mapped to potential domains and core areas according to COMET

Social activities with neighbours	family, friends, neighbours and groups		
Social activities with family			
Social activities with groups			
Dependence on family and friends			
Appearance interferes with social activities			
Intimate relationships	Effect on intimate relationships		
Emotional impact on work	Emotional distress/mood	Emotional functioning	
Energy levels			
Emotional impact on ADL			
Happiness			
Impact on life enjoyment / satisfaction			
Emotional impact on relationships			
Anxiety			
Depression			
Acceptance/ Adjustment	Thoughts and beliefs (acceptance, coping)		
Coping with trauma			
Confidence	Self esteem and confidence		
Self esteem			
Body image	Body image		
Quality of life	Quality of Life	Global Quality of Life	Quality of Life
Rating of health	Perceived Health status	Health status	Health status
General patient satisfaction	Patient satisfaction	Delivery of Care	Delivery of Care
Satisfaction with appearance of arm			
Satisfaction with function			
Satisfaction with movement			
Satisfaction with strength			

Article title: Developing a core outcome set for Traumatic Brachial Plexus Injuries: a systematic review of outcomes

Miller C, *et al. BMJ Open* 2021; 11:e044797. doi: 10.1136/bmjopen-2020-044797

Supplementary file 6: Unique outcomes mapped to potential domains and core areas according to COMET

Satisfaction with pain			
Satisfaction with colour			
Satisfaction with shape			
Satisfaction with feeling			
Satisfaction with procedure			
Patient preference	Patient preference		
Quality of intervention	Accessibility, quality and adequacy of intervention		
Time to surgery	Time to surgery		
Operation time	Operation time	Resource Use	Resource Use
Motor morbidity	Donor site morbidity	Adverse Events	Adverse Events
Sensory morbidity			
Pain			
General complications	General complications		
Pneumothorax	Respiratory complications		
Respiratory function			
Respiratory symptoms			
Pneumonia			
Arterial thrombosis	Vascular complications		
Venous thrombosis			
Haematoma			
Venous spasm			
Iatrogenic vascular injury			
Vascularity of flap			
Swelling			
Fracture	Musculoskeletal complications		
Passive range of motion loss			
Co-contraction			
Bowstringing			
Failure of tendon attachment			
Joint Instability			
Scapula crepitus			

Supplementary file 6: Unique outcomes mapped to potential domains and core areas according to COMET

Infection complications	Infection complications		

Dodd, S. et al. A taxonomy has been developed for outcomes in medical research to help improve knowledge discovery. Journal of clinical epidemiology. 2018, 96: 84–92.
