# Supplementary material for: Developing a core outcome set for traumatic brachial plexus injuries: a systematic review of outcomes
Source: BMJ Open. 2021 Jul 30;11(7):e044797. doi: 10.1136/bmjopen-2020-044797 (PMC8327802; doi:10.1136/bmjopen-2020-044797)
Supplement: Supplementary data [file bmjopen-2020-044797supp007.pdf]

Supplementary file 7. Measurement instruments mapped to domains

Supplementary file 7. Measurement instruments mapped to domains

56 outcome subdomains in 4 core areas (Physiological/clinical, Life Impact, Resource Use and Adverse events) and within the following COMET domains

Musculoskeletal/connective tissue, Nervous system outcome domain, General outcome and symptom domain, Physical functioning, Role functioning, Emotional functioning, Global quality of life, Perceived health status, Delivery of care, Hospital resources and Adverse Events

**Core Area**	Outcome subdomains	**Measurement type used (N)**				**Measurement instruments used (number of studies)**
**PHYSIOLOGICAL /CLINICAL**		**Patient reported Outcome**	**Clinician reported Outcome**	**Performance Outcome**	**Not Clear**	
	**Musculoskeletal/connective tissue**					
	Muscle strength	31	131	20	3	DASH (n= 28), UEFI (n=2), MHQ (n=1)<br><br>**Manual Muscle Testing**<br>**Manual muscle testing undefined (n=5)**<br>**MRC muscle grading (n=62, including UCLA)**<br>**MRC muscle grading modified (n= 24)**<br>*MRC modified*, unclear how (n= 6)<br>*MRC modified,* grade 3 active must equal passive (n=2)<br>*MRC modified ,* grade 2 active must equal passive movement (n=2)<br>*MRC modified,* M3+ contraction with resistance against a finger for less than 30 seconds, M4 contraction of resistance against a finger against a finger for more than 30 seconds (n=1)<br>*MRC modified*: M0, M1+, M1, M1+, M2-, M2, M2+,M3-, M3, M3+, M4-, M4, M4+, M5-, M5 (n=6)<br>*MRC modified,* Finger flexion tested with wrist extended 20-30 degrees (n=1)

Supplementary file 7. Measurement instruments mapped to domains

						MRC modified, Addition of M4.5 (n=1)<br>*MRC modified,* graded two muscles together (n=1)<br>*MRC modified,* finger extension tested with wrist extension at 20-30 degrees (n=1)<br>*MRC modified,* summated muscle score (n=1)<br>*MRC modified,* FDS tested by stabilising LF and IF to table and testing MF and RF IP flexion (n=1)<br>Lovett & Sunderlands standardisation (n=1)<br>**Other manual muscle tests (n=3)**<br>Kendall and McCreary testing procedure (n=1)<br>Oxford muscle testing (n=1)<br>Modification of the Louisiana State University Medical Centre grading system (n=1)<br>**Time to (n= 12)**<br>contraction (n=7); M2 (n=1); strength greater than or equal to M3 (n=1); M 3 (n=1); greater than or equal to modified M3 (n=1); Time to improvement in MRC scale (n= 1)<br>**Dynanometry (n=23)**<br>Dynanometry – isokinetic machine, undefined method (n =1)<br>Grip strength JAMAR , undefined method (n=4); Hook grip – isokinetic machine, undefined method (n=1); Grip strength JAMAR, mean of 3 trials n=2); Grip strength , PABLO system, undefined (n=1); Pinch grip, JAMAR, undefined (n= 3), Pinch grip JAMAR, mean 3 trials (n= 1); Peak isometric, hand held dynamometer (n=2); Isometric strength , hand held dynamometer, best of 3 trials (n=1); Isometric strength , Kendall & Kendall positions, 3 trials mean value (n=1); Measurement on digital scales after 5 seconds (n=1)<br>Concentric strength through range, Isokinetics (n=1)<br>Eccentric strength through range, isokinetics (n=1)<br>Combined action of using elbow and hand on digital hanging scale (n=1)

Supplementary file 7. Measurement instruments mapped to domains

						Constant-Murley score: dynanometry 90 degrees abduction(n=2)<br><br>**Narakas score modified (one study)**<br>Thoaraco brachial grasp (n=1)<br>Elbow flexion with weight (n=1)<br>Elbow extension with weight (n=1)<br>Wrist flexion with weight (n=1)<br>Wrist extension with weight(n=1)<br>Fist power with weight (n=1)<br>Pinch power (n=1)<br><br>**ULM (one study)**<br>Shoulder flexion to shoulder height with 500g (n=1)<br>Shoulder flexion above shoulder height with 500g (n=1)<br>Shoulder flexion above shoulder with 1kg (n=1)<br>Move weight on table (100g) (n=1)<br>Move weight on table (500g) (n=1)<br>Move weight on table (1KG) (n=1)<br><br>**SHAP (two studies)**<br>Grip strength (n=2)<br>Pinch strength (n=2)<br>Pinch grip (lateral) (n=2)<br>Pinch grip (tip) (n=2)<br>Grip strength (power) (n=2)<br>Heavy extension (n=2)<br><br>Ability to lift weight, undefined (n=1)<br>Number of repetitions movement can be performed in 10 seconds (n=1)<br>Maximum weight sustained when flexing elbow (n=1)

Supplementary file 7. Measurement instruments mapped to domains

						**Unclear (n= 3)**<br><br>Force recovery: Cross sectional area of the muscle under isometric contraction divided by cross sectional area at rest (n=1)
	Active movement	5	105	4	63	SST(n=1), MHQ (n=1), UCLA shoulder rating scale (n=1), MPI(n=2), CONSTANT- MURLEY(n=2) (2xPRO, 8x ClinRO), ARAT (PerfO, n=2), ULM (PerfO, n=2), Goniometry(n=50), MALLET (n=1), Visual assessment (n=32), First web space in cm (n=3), Total active movement(n=2), Pulp to palm distance (n=2)<br>Months to full active movement (n=1)<br>Months to antigravity movement (n=3)<br>Months to initial movement (n= 1)<br>Months to independent movement without donor (n=1)<br>Not clear (n=63)
	Passive range of movement		6		7	Not defined (n=7), Goniometry(n=6)
	Movement control and stability		1	1	2	MPI (ClinRo, n=1), ULM (PerfO, n=1), Not clear (n=2)
	Bone structure/position/healing				4	Not clear (n=4)
	Muscle mass				4	Not clear(n=4)
	**Nervous system outcome subdomains**					
	General sensory recovery including proprioception		**9**		**8**	Sensory BMRC (n=5), Modified Sensory BMRC (n= 2), Highet classification(n=2), Not clear (n=8)
	Discriminative touch (light touch, two point discrimination, vibration, object recognition)	**1**	**14**			MHQ (n=1), Cotton wool (n=3), Semmes Weinstein Monofilaments (n=4), Two point discrimination(n=2), Tuning fork (n=4), Not defined (ClinRo, n=1)
	Protective touch (pain, temperature, deep pressure)		**3**		**7**	Blunt pin (n=3), Not clear (n=7)
	Structure of peripheral nervous system		1			MRI (n=1)
	Reinnervation (level of reinnervation, time to innervation)		54			Two point scale on EMG(n=1) Four point scale on EMG (n=4), Not clear EMG (n= 49)
	Progression of regeneration		5			Tinel sign (n=5)

Supplementary file 7. Measurement instruments mapped to domains

	Speed of motor and sensory conduction		9			EMG (n=9)
	**General outcomes / symptoms**					
	Pain intensity/ relief	81			3	DASH (n=27), ASES (n=1), TAPES (n=1), VAS(n=20), NRS(n=12),MHQ (n=1) WBFRS(n=1), BPI (n= 4), UNWNS (n=1), McGill Pain Questionnaire SF (n=2), McGill pain questionnaire (n= 2), MPI (n=1), CONSTANT-MURLEY (n=2), 4 point scale (n=3) Author developed questionnaire(n=1), Not Clear (n=3), QuickDash (n=1), EQ5D 3L (n=1)
	Pain duration or frequency	18	0	0	0	SST (n=1), SF36 (n=8), MHQ (n=1),TAPES(n=1), NPSI (n=1), BPI (n= 4), UCLA shoulder rating score (n=1), Not described PRO (n=1)
	Pain quality	8				TAPES (n= 1), NPSI(n=1), UWNS(n= 1), McGill SF(n=2), McGill (n=2), Non described PRO (n =1)
	Pain when arm exposed to cold	1				NPSI (n=1)
	Paraesthesia	28				DASH (n=27), QuickDash(n=1)
	Sensitivity to touch, pressure, vibration etc	3				NPSI (n=1) UWNS (n= 1), NRS (n=1)
	Location of pain	4				BPI (n=4)
	Pain medication use	4				BPI (n=4)
	Stiffness	27				DASH (n=27)
**LIFE IMPACT**	**Physical functioning**					
	Physical function non-specific	3				PSFS (n=2), TAPES (n=1)
	Lower limb and non-upper limb function (walking, running, climbing stairs etc)	14			1	SF36 (n=8), TAPES (n= 1), BPI (n=4) Non described PRO (n=1), EQ5D-3L (n=1)
	Reaching, pulling, pushing, carrying, throwing , lifting	41		4		DASH (n=28), UEFI (n=2), MHQ(n=1), ASES(n=1), SST (n=1), SF36(n=8), ARAT(n=2), AMULA (n=1) UNBtP (n=1)
	Turning twisting, gripping and releasing with the arm	33		6	1	DASH (n=28), UEFI (n=2), MHQ (n=1),ARAT(n=2),SHAP(n= 2), JHFT (n=1), AMULA (n=1), UNBtP (n=1), Not clear (n=1), QuickDash (n=1)
	Fine hand movement include writing	32		7		DASH (n=28), UEFI (n=2), MHQ (n=1),ARAT(n=2), SHAP(n=2), JHFT (n=1) Purdue Peg test (n=1),AMULA (n=1), UNBtP (n=1)
	**Role Functioning**					

Supplementary file 7. Measurement instruments mapped to domains

	Impact on return to work	46				DASH (n =28), UEFI (n=2),MHQ (n=1), ASES (n=1), SST (n=1), SF36 (n=8), TAPES (n=1), MPI (n=1) No description PRO (n=1), Questionnaire no data (n=1), QuickDash(n=1)
	Role function patient specific	2				PSFS(n=2)
	Carrying out daily routine, (including food preparation, housework, garden, plants)	43	1	5		DASH (n=28), UEFI (n=2), MHQ (n=1),TAPES(n=1) , BPI (n=4), UCLA (n=1), SHAP (n=2), Jebsen (n=1), ULM (n=1) Questionnaire not defined (n=2),No description PRO (n=1) Unclear CLinRO(n=1), AMULA (n=1), UNBtP (n=1), QuickDash(n=1), EQ5D 3L (n=1)
	Maintaining personal hygiene	41		2		DASH (n=28), ASES (n=1), SST(n=1), SF36(n=8), MHQ(n=1) AMULA (n=1), UNBtP (n=1), QuickDash(n=1), EQ5D 3L (n=1)
	Maintaining personal appearance	3		1		UEFI (n=2), ASES (n= 1), AMULA (n=1)
	Dressing	33		2		DASH (n =28), UEFI (n=2), MHQ (n=1),ASES (n= 1), SST (n=1), AMULA (n=1) SHAP(n=2)
	Transport needs	29				DASH (n =27), UEFI (n=2),
	Impact on recreational activities and sport	36				DASH (n =28), UEFI (n=2), ASES (n= 1), TAPES(n=1), CONSTANT-MURLEY (n=2),Not described PRO (n=1), QuickDash(n=1)
	**Social functioning**					
	Effect on relationship with family, friends, neighbours and groups	43				DASH (n =28), SF36 (n=8), TAPES (n=1), MHQ (n=1), QuickDash(n=1), BPI (n=4)
	Effect on intimate relationships	28				DASH (n =28)
	**Emotional Functioning**					
	Emotional distress/ mood	18				SF36 (n=8), TAPES (n= 1),BPI(n=4), UWNS(n=1), Self-rated anxiety scale (n=1), Self-rated depression scale (n=1), MHQ (n=1), EQ5D 3L (n=1)
	Thoughts and beliefs (acceptance and adjustment)	1				TAPES (n=1)
	Self-esteem and self confidence	29				DASH (n=28), TAPES(n= 1)
	Body image	3				MHQ (n= 2), Not described (n=1)
	**Sleep and overall health**					

Supplementary file 7. Measurement instruments mapped to domains

	Impact on sleep	41				DASH (n=28), UEFI (n=3), ASES(n= 1), MHQ (n=1), SST (n=1), BPI(n=4), CONSTANT- MURLEY(n=2),Not described PRO (n=1)
	**General Quality of life**	1				Not described PRO (n=1)
	**Perceived Health Status**	10				SF36 (n=8), TAPES (n=1), Eq5D 3L (n=1)
	**Delivery of Care**					
	Patient satisfaction	10				TAPES (n=1), UCLA (n=1), MHQ (n=1),10-point scale (n=1) 4 point scale (n=2), 3 point likert scale (n=1), Questionnaire not described (n=1),Not defined PRO(n=2)
	Patient preference for treatment	1				Not described (n=1)
	Accessibility, quality and adequacy of intervention				1	4 point scale (n=1)
**RESOURCE**	**Hospital**					
	Operation time				1	Not described (n=1)
	**Adverse Events**					
**ADVERSE EVENTS**	Donor site motor morbidity to include weakness		18		19	BMRC (n=7), BMRC modified(n=2), Dynanometry (n=8), EMG(n=1),Not clear (n=19)
	Donor site sensory morbidity	1	3		4	10-point scale PRO (n=1),Not defined (n=4),2PD (n=2), Monofilaments (n=1)
	Donor site morbidity -pain	3				Not defined PRO (n=3)
	General complications				3	Unclear (n=3)
	Respiratory complications	1	5		4	4 point scale PRO (n=1), x-ray (n=2), FEV (n=1), TLC(n=1), MVV (n-1), Not defined (n=4),
	Vascular complications		2		13	Not defined (n=13), Visual assessment (n=1), USS (n=1)
	Musculoskeletal complications		2		19	Not defined CLinRO(n=2), Unclear (n=19)
	Infection complications		1		2	Not defined ClinRo(n=1), Unclear (n=2)
		757	370	52	169	

Supplementary file 7. Measurement instruments mapped to domains

DASH Disabilities of the arm shoulder and hand, *UEFI* Upper Extremity Functional Index, *MHQ* Michigan Hand Questionnaire, *BMRC* British Medical Research Council, *ULM* Upper Limb Module, *SHAP* Southampton Hand Assessment Procedure, *SST* Simple Shoulder Test, *MPI* Mayo clinic Performance Index for the elbow, *ARAT* Action Research Arm Test, *ClinRO* Clinician Reported Outcome, *PerfO* Performance Outcome, *PRO* Patient Reported Outcome, *ASES* American Shoulder and Elbow Surgeons Index, *TAPES* The Trinity Amputation and Prosthesis Experience Scales, *VAS* Visual Analogue Scale, *NRS* Numerical Rating Scale, *WBFRS* Wong Baker Faces Rating Scale, *UNWNS* University of Washington Neuropathic pain Score, *SF36* Short Form 36 health survey, *NPSI* Neuropathic Pain Symptom Inventory, *BPI* Brief Pain Inventory, *PSFS* Pain Specific Functional Scale, *AMULA* American Measures for Upper Limb Amputees, *UNBPT* University of New Brunswick test of Prosthetics function, *JHFT* Jebsen Hand Function Test, *FEV* Forced Expiratory Volume, *TLC* Tidal Lung Capacity, *MVV* maximal voluntary ventilation, *USS* Ultrasound Scan.
