# Supplementary material for: Developing a core outcome set for traumatic brachial plexus injuries: a systematic review of outcomes
Source: BMJ Open. 2021 Jul 30;11(7):e044797. doi: 10.1136/bmjopen-2020-044797 (PMC8327802; doi:10.1136/bmjopen-2020-044797)
Supplement: Author's manuscript [file bmjopen-2020-044797.draft_revisions.pdf]

BMJ Open is committed to open peer review. As part of this commitment we make the peer review history of every article we publish publicly available.

When an article is published we post the peer reviewers' comments and the authors' responses online. We also post the versions of the paper that were used during peer review. These are the versions that the peer review comments apply to.

The versions of the paper that follow are the versions that were submitted during the peer review process. They are not the versions of record or the final published versions. They should not be cited or distributed as the published version of this manuscript.

BMJ Open is an open access journal and the full, final, typeset and author-corrected version of record of the manuscript is available on our site with no access controls, subscription charges or pay-per-view fees (http://bmjopen.bmj.com).

If you have any questions on BMJ Open's open peer review process please email info.bmjopen@bmj.com

BMJ Open

Developing a core outcome set for traumatic brachial plexus injuries: a systematic review of outcomes.

Journal:	*BMJ Open*
Manuscript ID	bmjopen-2020-044797
Article Type:	Original research
Date Submitted by the Author:	13-Sep-2020

Complete List of Authors:	Miller, Caroline; University of East Anglia, School of Health Sciences, The Queens Building ; Queen Elizabeth Hospital Birmingham, Physiotherapy Department<br>cross, jane; University of East anglia, school AHP<br>O'Sullivan, Joel; Queen Elizabeth Hospital Birmingham, Physiotherapy<br>Power, Dominic; Queen Elizabeth Hospital Birmingham, The Birmingham Peripheral Nerve Injury Service<br>Kyte, Derek; University of Birmingham, Institute of Applied Health Research<br>Jerosch-Herold, Christina; University of East Anglia, Health Sciences

Keywords:	Neurological injury < NEUROLOGY, Limb reconstruction < ORTHOPAEDIC & TRAUMA SURGERY, Plastic & reconstructive surgery < SURGERY

SCHOLARONE™ Manuscripts

I, the Submitting Author has the right to grant and does grant on behalf of all authors of the Work (as defined in the below author licence), an exclusive licence and/or a non-exclusive licence for contributions from authors who are: i) UK Crown employees; ii) where BMJ has agreed a CC-BY licence shall apply, and/or iii) in accordance with the terms applicable for US Federal Government officers or employees acting as part of their official duties; on a worldwide, perpetual, irrevocable, royalty-free basis to BMJ Publishing Group Ltd ("BMJ") its licensees and where the relevant Journal is co-owned by BMJ to the co-owners of the Journal, to publish the Work in this journal and any other BMJ products and to exploit all rights, as set out in our licence.

The Submitting Author accepts and understands that any supply made under these terms is made by BMJ to the Submitting Author unless you are acting as an employee on behalf of your employer or a postgraduate student of an affiliated institution which is paying any applicable article publishing charge ("APC") for Open Access articles. Where the Submitting Author wishes to make the Work available on an Open Access basis (and intends to pay the relevant APC), the terms of reuse of such Open Access shall be governed by a Creative Commons licence – details of these licences and which Creative Commons licence will apply to this Work are set out in our licence referred to above.

Other than as permitted in any relevant BMJ Author's Self Archiving Policies, I confirm this Work has not been accepted for publication elsewhere, is not being considered for publication elsewhere and does not duplicate material already published. I confirm all authors consent to publication of this Work and authorise the granting of this licence.

For peer review only

Title Page

Title of article

Developing a core outcome set for traumatic brachial plexus injuries: a systematic review of outcomes.

For peer review only

Corresponding author

Caroline Miller (corresponding author)

School of Health Sciences,

University of East Anglia,

Norwich,

UK,

NR4 7TJ

Tel no: 00447743501508

Email: caroline.miller@uea.ac.uk

Twitter handle: @c_ahern26

Co-authors

Jane Cross

School of Health Sciences,

University of East Anglia,

Norwich,

UK,

Twitter handle: @JaneCross12

For peer review only - http://bmjopen.bmj.com/site/about/guidelines.xhtml

Joel O Sullivan,

Physiotherapy Department,

Queen Elizabeth Hospital Birmingham,

Birmingham

UK,

Twitter handle: @jos2305

Dominic M Power,

The Birmingham Peripheral Nerve Injury Service,

Queen Elizabeth Hospital Birmingham,

Birmingham,

UK,

@dominicpower1

Derek G Kyte,

Centre for Patient-Reported Outcomes Research,

University of Birmingham,

Birmingham,

UK

Christina Jerosch-Herold,

School of Health Sciences,

University of East Anglia,

Norwich,

UK,

Twitter handle: @tinajerosch

Word count, 3,300

For peer review only

ABSTRACT

Objective This review aimed to summarize outcome reporting in traumatic brachial plexus injury research.

Method Medline (OVID), EMBASE, CINAHL, and AMED were systematically searched for studies evaluating the clinical effectiveness of interventions in traumatic brachial plexus injuries. Two authors independently screened papers. All outcomes were extracted verbatim from studies. If a patient reported or performance outcome measure was used then outcomes were extracted directly from the instrument. Variation in outcome reporting was determined by assessing the number of unique outcomes reported across all included studies. Outcomes were categorized into domains using a prespecified taxonomy.

Results Verbatim outcomes (n= 1460) were extracted from 132 studies including 30 questionnaires. Unique outcomes (n= 157) were structured into four core areas and 11 domains. Outcomes within the musculoskeletal domain were measured in 87% of studies, physical functioning in 23%, emotional functioning in 22% and adverse events in 33%. One study measured quality of life. We identified 62 different methods for measuring muscle strength, 16 for range of movement and 63 studies did not define how they measured movement.

Conclusion This review of outcome reporting in traumatic brachial plexus injury research demonstrated an impairment focus and heterogeneity. A core outcome set would ensure standardized and relevant outcomes are reported to facilitate future systematic review and meta-analysis.

For peer review only - http://bmjopen.bmj.com/site/about/guidelines.xhtml

Prospero registration number: CRD42018109843

Strengths and limitations of this study

- This study is a comprehensive and systematic review of all reported clinical outcomes reported in traumatic brachial plexus studies from 2013- 2018 inclusive.
- Unique outcomes were systematically categorized into a clear taxonomy to inform the development of a core outcome set.
- Definition of unique outcomes and categorisation was conducted by researchers and clinicians to account for multidisciplinary perspectives.
- Quality assessment was not undertaken as the aim of the study was to review outcome reporting and not to synthesize data about effectiveness of interventions.

For peer review only

INTRODUCTION

A traumatic brachial plexus injury (TBPI) is a major injury to the brachial plexus. It can result in significant functional, social, psychological and economic effects,[1, 2] with most occurring in young men as a result of motorbike accidents,[3]. Survival from major trauma is increasing,[4] and with this an increase in the incidence of TBPI,[5] which accounts for 1.2% of polytrauma,[6].The complex and chronic nature of the injury is associated with significant healthcare costs,[7] in addition to indirect costs estimated at $2.34 million (in 2017 dollars) over the lifetime of an individual with a TBPI,[8]. There are multiple strategies for managing a patient with a TBPI with recent advancements in nerve microsurgery,[9] and robotics,[10] resulting in increased treatment options. The choice of treatment should be made using up-to-date, high quality scientific evidence,[11, 12].

Ideally, a meta-analysis would identify the most effective treatment for an individual with a TBPI, however, such analysis requires homogenous outcome measurement and reporting across studies to enable optimum synthesis. Indeed, despite increasing numbers of TBPI studies, outcome heterogeneity and poorly defined outcomes has been highlighted as a significant challenge to evidence synthesis in two recent systematic reviews,[13,14]. There is now international agreement that the definition of a core outcome set (COS) for TBPI is a priority,[15, 16]. A COS is a minimum agreed set of outcomes to be reported and measured in all studies and collected through routine clinical care,[17, 18]. Development of a COS has been shown to reduce heterogeneity of outcome reporting in other health conditions, with 81% of trialists in rheumatoid arthritis (RA) now measuring the COS for RA,[19].

For peer review only - http://bmjopen.bmj.com/site/about/guidelines.xhtml

To date a minimum set of outcomes, important to patients and professionals for reporting in TBPI studies, has not been agreed. The choice of what are important outcomes to measure in TBPI is complex due to patient heterogeneity with different mechanisms, locations and severity of injury. As a first step in the development of an international COS for TBPI we conducted a systematic review to identify outcomes reported in the literature.

The aim of this review was to:

1. Identify what outcome domains are assessed in studies evaluating surgical and non-surgical treatment for TBPI.
2. Compare the definitions of outcomes and time points of outcomes assessed.
3. Identify measurement instruments used to assess outcome domains.

For peer review only

For peer review only - http://bmjopen.bmj.com/site/about/guidelines.xhtml

METHODS

We followed the methods described in the Cochrane Handbook for Systematic Reviews of Interventions,[20] and report in accordance with the Preferred Reporting Items for Systematic Reviews and Meta-Analysis (PRISMA) guidelines,[21] and Core Outcomes Sets Standards for Reporting (COS-STAR) guidelines,[22]. The systematic review protocol was prospectively registered with PROSPERO (PROSPERO registration number: CRD42018109843).

Identification of studies

We conducted an electronic search of Medline (OVID), EMBASE (OVID), CINAHL and AMED on the 18th September 2018. Studies published between 01 Jan 2013 and 18 September 2018 were included to reflect outcomes employed in current TBPI care. An example of the search strategy for Ovid MEDLINE is presented in supplementary file 1. The thesaurus vocabulary of each database was used to adapt search terms. Boolean operators (AND, OR) were used to narrow or widen the search and no language restrictions were applied.

Study eligibility

Studies were included if they met the following criteria:

Study type: Any controlled and uncontrolled experimental and observational studies evaluating interventions in traumatic brachial plexus injury including case reports, case series, case studies, prospective and retrospective cohort studies, randomized and non-randomized clinical trials. We excluded conference proceedings, abstract only publications and those not involving human subjects.

For peer review only - http://bmjopen.bmj.com/site/about/guidelines.xhtml

Participants: Studies reporting outcomes in individuals with traumatic brachial plexus injury aged 16 years or over. Studies of patients with obstetric brachial plexus injuries were excluded.

Interventions: Any surgical or non-surgical intervention for TBPI.

Outcomes: All outcomes reported in the published abstract, methods or results. These included physiological and functional outcomes, adverse events and patient reported outcomes (PROs) either reported in the study or subsequently extrapolated from the PRO instruments.

Language: Non-English language publications were included

Study selection process

The reference management software Mendeley was used to compile the literature, with duplicates removed. Authors (X and X) independently screened the titles and then the abstracts against the eligibility criteria. Disagreements were discussed and a third reviewer (x) was involved where required. Studies appearing to meet the inclusion criteria based on title and abstract were retrieved as full text articles, and were read to assess for eligibility with decisions on inclusion and exclusion recorded (Figure 1. PRISMA flow diagram). Disagreements in study selection were resolved by discussion within the research team (x, x, x).

For peer review only

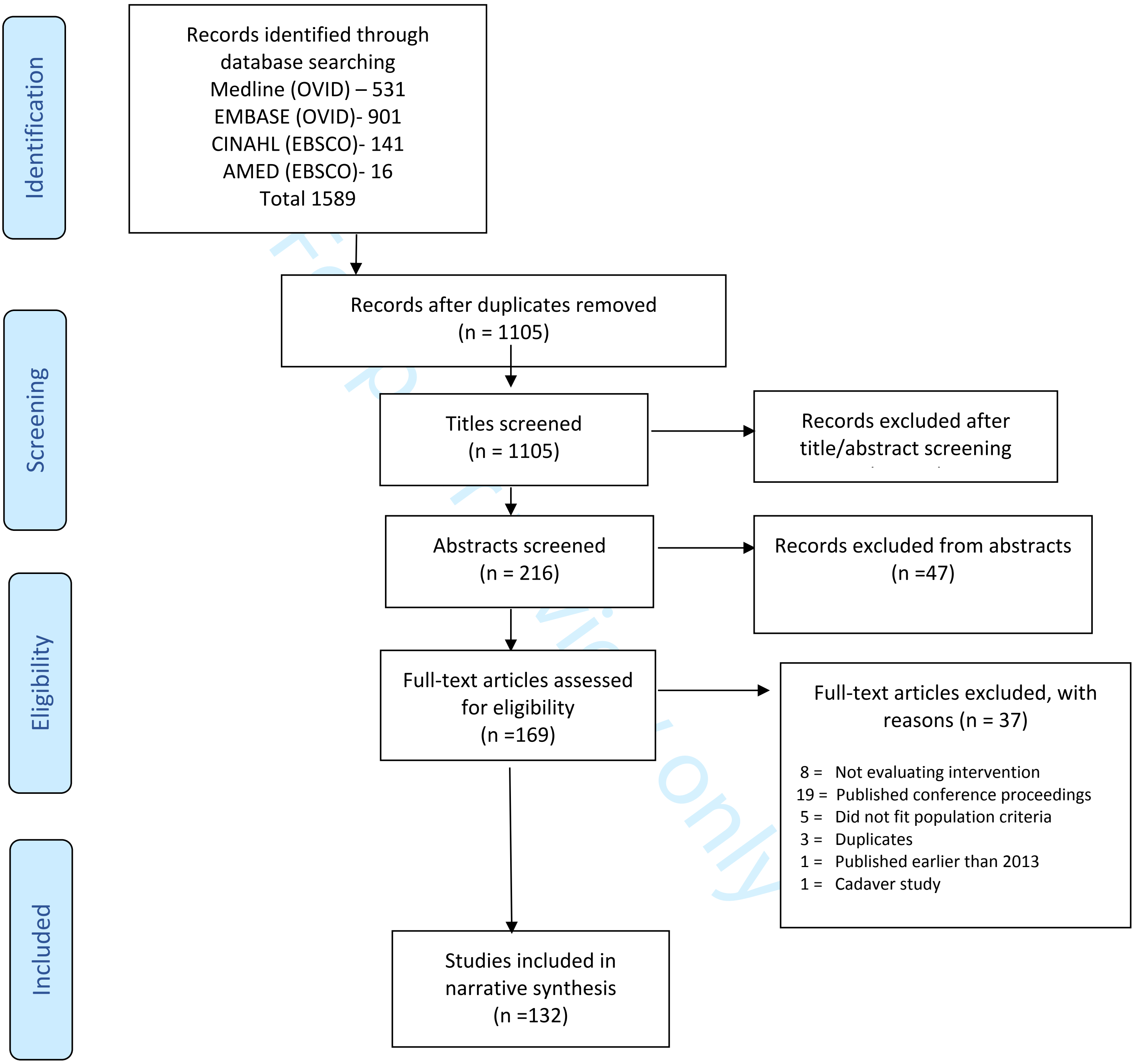

Figure 1. Preferred Reporting Items for Systematic Reviews and meta-analysis flow diagram.

For peer review only - http://bmjopen.bmj.com/site/about/guidelines.xhtml

Quality assessment

Quality assessment of studies was not relevant as the objective was to systematically document all outcomes reported in TBPI studies rather than synthesize the data about intervention effectiveness.

Data Extraction

Data were extracted into a piloted data extraction sheet (Microsoft Excel). General data extracted from each study included author, study design, recruiting country, publication year, number of participants, gender, mean age, level of TBPI and intervention tested. The following information was extracted regarding outcomes: each outcome reported (verbatim), area of body assessed if relevant (shoulder, elbow, wrist or hand), method of administration, name of measure, timepoints of measure and reported complications. The number of outcomes per study was also documented.

Data extraction was performed independently by X and X for the first 20% of included studies. These were compared, and disagreements discussed and resolved through debate or discussion with a third reviewer (X). Following this a further ten percent of studies had data extracted by both X and X. Due to the high level of agreement between reviewers (91% agreement) on outcomes extracted, at this stage, the remaining studies underwent extraction by a single reviewer (X).

For peer review only

For peer review only - http://bmjopen.bmj.com/site/about/guidelines.xhtml

If an instrument was used and was composed of multiple items, including patient-reported questionnaires, the following data was extracted by the first author; verbatim name of the instrument, verbatim name for each item. The frequency of use of instruments was noted and compared between studies. The instruments were categorized as: (i) General Health (generic - for use with any patient); (ii) Upper limb physical function (region-specific); (iii) Symptom or domain specific (to assess a single symptom e.g. pain) and (iv) Condition Specific. Timepoints of measurement of all outcomes were noted. If the outcome was assessed at different timepoints then all timings were recorded.

Classification of outcomes into domains and defining unique outcomes

Identically worded and spelled verbatim outcomes were removed at this stage. Identical outcomes measured over different time points were noted as one outcome. Where outcomes were assessed using an instrument containing several items, each individual item was assigned an outcome name using the International Classification of Functioning and following standard linking rules,[23].

X categorized all outcomes into an outcome taxonomy developed by COMET for categorizing outcomes for core outcome set development,[24]. These included 5 core areas and 38 outcome domains. This is presented in supplementary file 2. A long list of all categorized outcomes was presented to researchers (X and X) at a face to face meeting where the categorization of all outcomes was reviewed using the recommended taxonomy. Subdomains were created within the larger taxonomy to manage the large variation in TBPI clinical outcomes extracted. Disagreements not resolved at this stage were discussed

For peer review only - http://bmjopen.bmj.com/site/about/guidelines.xhtml

further with subject experts (for example, the Adverse Event domain was discussed with a surgeon).

Due to the diversity in terminology used to report outcomes, we grouped similar outcomes within each subdomain. It is recommended that outcomes with different words, phrasing, or spelling addressing the same concept should be categorized as a unique outcome,[25]. For example, active range of motion of shoulder abduction and active goniometry of shoulder abduction were named as active shoulder abduction range and grasp strength and grip strength were named as grip strength. Independent meetings were held with four subject experts to ratify and define unique outcome names within each domain.

Patient and public involvement

The need for a COS in TBPI care was conceived following discussions with patients and health professionals. Patients highlighted the diverse effect the injury has on their life and that often these outcomes were overlooked by professionals, such as body image. There is a patient advisory group for the COS and the systematic review was discussed at these meetings. Patients were not actively involved in data collection or analysis of this review. Dissemination will occur at the annual traumatic brachial plexus charity UK meeting where updates from the project are presented yearly and through a six monthly newsletter.

For peer review only

For peer review only - http://bmjopen.bmj.com/site/about/guidelines.xhtml

Results

Included studies

The search identified 1159 studies, after removing duplicates 1105 studies remained. Titles and abstract review identified 169 potentially relevant articles. Of these, 37 studies did not meet the inclusion criteria and were excluded (PRISMA flow diagram; figure 1) thus, 132 studies formed the basis of this review. All included articles are presented in supplementary file 3.

Study characteristics

Thirty-two countries from six continents recruited 3201 participants into the 132 studies (Table 1). Of the 132 studies, 87 (66%) were retrospective case series with most studies published from Asia (n=61, 46%). The most frequently studied surgical intervention was nerve transfers (n=66, 57%).

For peer review only

For peer review only - http://bmjopen.bmj.com/site/about/guidelines.xhtml

Table 1. Characteristics and demographics of included studies

	Study number (%)
Number of retrospective studies	87/132(66)
Number of prospective studies	21/132 (16)
Number of case studies	23/132(17)
Randomized controlled trial	1/132 (0.8)
**World region recruitment**	
Asia	61/132(46)
North America	20/132(15)
South America	20/132(15)
Europe	27/132(20)
Africa	3/132(2.2)
Australasia	1/132(0.8)
**Year published**	
2013	25/132 (19)
2014	24/132(18)
2015	15/132(11)
2016	30/132(23)
2017	27/132(20)
2018	11/132(8.3)
**Gender (total 3201)**	
Male	2622/3201(82)
Female	323/3201(10)
Not stated	256/3201(7.9)

For peer review only

**Site of plexus injury per study (n=132)**	
Upper trunk	26/132(20)
Lower trunk	10/132(7.6)
Pan plexus (all avulsed)	50/132(38)
Infraclavicular	7/132(5.3)
Mixture	32/132(24)
Unclear	7/132(5.3)
**Interventions (n=132)**	
Surgical	115/132(87)
Electrotherapy	2/132(1.5)
Pain treatments	11/132 (8.3)
Rehabilitation	2/132(1.5)
Orthotic	1/132(0.7)
Stem cell	1/132(0.7)
**Types of surgical intervention (n=115)**	
Neurotisation	66/115(57)
Tendon transfer	7/115(6.1)
Free flap	16/115(14)
Multiple surgeries	12/115(10)
Contralateral C7	8/115(6.9)
Other	6/115(5.2)

For peer review only

Outcomes

A total of 1460 verbatim outcomes were reported, after removing duplicates 157 different unique outcomes remained. No single outcome was reported across all 132 studies.

Outcome definition variation. Many outcomes were not clearly defined and different terms were frequently found for the same concept. For example, shoulder abduction strength was described in eleven different ways including 'deltoid strength', 'motor function of axillary nerve', 'motor recovery of shoulder abductors', 'muscle power supraspinatus', 'motor function of Deltoid', 'motor function of Supraspinatus'.

Outcome timing variation: Of the 1460 verbatim outcomes, 46% (672) were measured between one and three years following intervention. For 83 outcomes the timing of the measurement was not stated. See Figure 2.

Place Figure 2 here

Figure 2. Timepoints of reported outcomes

For peer review only

Outcome domains: The 157 different types of outcomes were categorized into four core areas (Physiological and Clinical, Life Impact, Resource Use, Adverse Events/Complications) and 11 domains according to the COMET recommendations,[24]. See supplementary file 4. The core area Physiological/Clinical included three domains: musculoskeletal and connective tissue outcomes, nervous system outcomes and general/symptom outcomes. The core area Life Impact included seven domains: physical functioning, social functioning, role functioning, emotional functioning, global quality of life, perceived health status and delivery of care. The core area Resource Use included one domain: hospital resources. The core area Adverse Events included one domain: adverse events. No outcome could be placed into the core area Death.

Tables 2 to 4 summarise the number of unique outcomes within each domain and the number of studies reporting these outcomes in each core area. The most frequently reported domains were all in the Physiological/ Clinical core area and included musculoskeletal and connective tissue (87%), nervous system (35%) and symptoms (36%). Forty-four studies (33%) reported complications/ adverse events.

For peer review only - http://bmjopen.bmj.com/site/about/guidelines.xhtml

Table 2. Physiological /Clinical Core Area

Outcome Domains	Number of unique outcomes reported within domain	Examples of unique outcomes	Number of studies reporting outcomes in domain (%)
Musculoskeletal and connective tissue	18	Active range of movement, muscle strength, muscle fatigue	115/132 (87%)
Nervous system	15	Progression of nerve regeneration,<br>ability to feel light touch, ability to feel pain	46/132 (35%)
General/ symptoms	23	Pain intensity/relief, pain duration, pain quality, pain when arm exposed to cold, stiffness, sleep, paresthesia	47/132 (36%)

For peer review only

Table 3. Life Impact Core Area

Outcome Domains	Number of unique outcomes reported within domain	Examples of unique outcomes	Number of studies reporting outcomes within domain (%)
Physical functioning	19	Reaching, fine hand movement	30/132 (23%)
Role functioning	23	Return to work, Impact on normal hobbies	33/132 (25%)
Social functioning	7	Social activities with family	30/132 (23%)
Emotional functioning	13	Body image, acceptance	29/132 (22%)
Global quality of life	1	Quality of life	1/132 (0.8%)
Perceived health Status	1	Health status rating	6/132 (4.5%)
Delivery of care	13	Patient satisfaction, quality of care, patient preference, time to surgery	11/132(8.3%)

For peer review only

Table 4. Adverse Events and Resource Use Core Areas

Outcome Domains	Number of unique outcomes reported within domain	Examples of unique outcomes	Number of studies reporting outcomes within domain
**Adverse Events Core Area**			
Donor site morbidity	3	Motor weakness, sensory loss	24/132(18%)
Musculoskeletal	7	Co -contraction, Passive movement	12/132 (9%)
Respiratory	4	Pneumothorax	6/132 (4.5%)
Vascular	7	Hematoma	7/132 (5.3%)
Infection	1	Infection	3/132 (2.3%)
General non specified complications	1	General complications	2/132 (1.5%)
**Resource Use Core Area**			
Hospital resource use	1	Operation time	1/132 (.75%)

For peer review only

For peer review only - http://bmjopen.bmj.com/site/about/guidelines.xhtml

Outcome Measurement

Outcomes were extracted from 30 different instruments; PRO measures (n= 20), combined clinician-reported and patient-reported measures (n= 3) and performance measures (n= 7). See table 5. These measures were reported 83 times in the included publications. Most outcome measures were used once (n= 25, 30%). The most frequently reported measures were the Disabilities of the Arm Shoulder and Hand (DASH,[26]) questionnaire (n=27 studies, 32%) and the Visual Analogue Scale (n=18, 22%). The median number of items per instrument was 15 ranging from one (Visual Analogue Scale, Numerical Rating Scale and Wong Baker Faces rating scale),[27] to 54,[28]. These items mapped to 34 different outcome domains.

There was wide variation in the methods used to measure outcomes. This is presented in supplementary file 5 (Measurement instruments mapped to domains). For example; 62 different measurements were used to evaluate muscle function, including the British Medical Research Council,[29] eleven different modifications of the British Medical Council, Isokinetics, Dynanometry and Constant - Murley score,[30]. In addition, it was often not clear which instrument was used for measurement of the outcomes. For example, the instrument used to measure active range of movement was not reported in 36% of total times (63/ 174) the outcome was assessed. Finally with regards to method of measurement 55 studies employed a PRO instrument to evaluate the intervention.

For peer review only

Table 5: Outcome measures used in included studies

		Number of items	Number of scales	Frequency (n=83)
Patient Reported Outcome Measures	**Upper limb physical function measures (n= 16)**			
	Disabilities of Arm Shoulder and Hand	38	3	27
	Upper Extremity Functional Index	20	0	2
	American Shoulder and Elbow Score	15	0	1
	Modified American Shoulder and Elbow Score	13	0	1
	Simple Shoulder test	12	0	1
	Michigan Hand Questionnaire	37	0	1
PRO & ClinRO Measure	University of California Los Angelus shoulder score	5	0	1
	Constant- Murley	5	0	1
	MAYO Performance Index	4	0	1
Performance Measures	Jebsen Taylor	7	0	1
	University of New Brunswick Test of Prosthetic Function for Unilateral Amputees (UNB)	30	3	1
	Upper Limb Module Questionnaire	22	3	1
	Action Reach Arm Test	19	4	1
	Southampton Hand Assessment Procedure	26	0	1
	Purdue Peg test	3	0	1
	Activities Measure for Upper Limb Amputees	24	0	1
Patient Reported Outcome Measures	**Generic questionnaires (n=2)**			
	36 item short form survey (SF36)	36	8	5
	Patient Specific Functional Score	4	0	1
	**Condition specific questionnaires (n=1)**			

For peer review only

Trinity Amputation and Prosthesis scale	54	5	1
**Symptom specific questionnaires (n=10)**			
Visual Analogue Scale	1	0	18
Numerical Rating Scale	1	0	6
Wong Baker Faces rating scale	1	0	1
Brief pain inventory	15	6	1
Neuropathic pain symptom inventory	10	5	1
University of Washington Neuropathic score	10	3	1
McGill Pain Questionnaire	28	3	1
McGill Pain Questionnaire SF	17	3	1
McGill Pain Questionnaire (Japanese version)	17	3	1
Self- rating anxiety scale	20	0	1
Zung Self rating Depression scale	20	0	1

For peer review only

DISCUSSION

This systematic review aimed to identify what outcome domains have been reported in studies evaluating interventions for TBPI, examine outcome definitions and timepoints and identify the instruments used to assess outcomes. We found a wide variation in reported outcomes, timing of outcomes and outcome instruments used. Furthermore, a lack of standardized definition for commonly reported outcomes was observed. This heterogeneity in outcome reporting across studies hinders evidence synthesis and results in research waste,[31].

The most commonly reported core area was Physiological/ Clinical including musculoskeletal, nervous system and symptom domains. Eighty-seven percent of studies reported musculoskeletal outcomes. However, there were 21 different outcomes reported in this category making comparison between studies difficult. Furthermore, the diversity of measures used to assess the outcomes increases the difficulty with synthesis. For example, muscle function/ strength was assessed using 59 different measures, whilst 10 studies did not report what measure they used. To compound this muscle strength was assessed by both physical examination by a clinician (86%) and also by asking the patient(10%).

For peer review only - http://bmjopen.bmj.com/site/about/guidelines.xhtml

Only 42% of studies (55/132) evaluated PROs and within these studies there was significant heterogeneity in the measurement instrument used. Twenty-three different instruments were used with 18 only ever used once. The DASH was the most common instrument employed, in just over half the studies evaluating a PRO. The PRO instruments also varied greatly in terms of content with some as simple as a single item whilst others included up to 54 items. Over 273 individual questionnaire items were evident from the 23 PRO instruments mapping to 34 different outcomes domains. This highlighted a lack of consistency with no domain being measured by all PRO instruments. None of the included PRO assessments were designed specifically for individuals with a TBPI. Although this may be beneficial in terms of comparison with other conditions, such instruments may not be sensitive to issues of importance to patients with TBPI. These issues combined pose major questions regarding the clinical interpretation of results from TBPI studies.

It is clear that that individuals with a TBPI suffer significant emotional and psychoscocial issues,[1, 32]. However such issues were infrequently and inconsistently measured within this review. Only one study considered Quality of Life (QoL) as an outcome,[33] using a single item PRO. Similarly, physical, role and social functioning outcomes were reported in 23%, 25% and 23% of studies respectively. This relates strongly to the use of the DASH within the studies. Indeed, emotional functioning was reported in 29 studies, 27 of these studies used the DASH which has one item on confidence and capability mapping to this

For peer review only - http://bmjopen.bmj.com/site/about/guidelines.xhtml

domain. If the DASH was excluded, only seven studies would assess outcomes within the emotional functioning domain. This is surprising considering the existing literature which evidences the complex emotional and psychological factors, individuals face when adjusting to their injury,[1, 34].

Complications/adverse events were reported in 33% of studies. Documentation of complications is crucial to improve patient care and gather data for benchmarking. In 1992, the Clavien-Dindo classification,[35] was introduced to assist with classification of complications to enable comparison between studies,[36]. However, within the adverse events outcomes identified in this review there was heterogeneity. Of the 37 verbatim outcomes reported within the donor morbidity (motor) outcome 19 did not define how this was assessed.

There are some limitations. We excluded outcomes from older studies to ensure we identified outcomes relevant to contemporary TBPI care. Formal quality assessment of studies was not undertaken, however the review was designed to identify the breadth of reporting in the literature and not to examine the effectiveness of interventions. The strengths of this review are that the protocol and the data extraction form were prespecified, prospectively registered and the literature search systematic. To account for multidisciplinary perspectives, researchers and clinicians where involved in categorizing outcomes into domains. It is the first review to detail the scale of outcome heterogeneity in TBPI research using a systematic method. International and non-English publications were included to reduce the risk of selection bias.

For peer review only

For peer review only - http://bmjopen.bmj.com/site/about/guidelines.xhtml

Variation in definitions and measurement of outcomes has been found within other areas of healthcare. Outcome heterogeneity is found in the reporting of outcomes relating to burn care,[37] breast reconstruction,[38] and spinal cord injury,[39] amongst others. A recent review of outcome reporting within burns illustrated wound healing was defined in 166 different ways across 147 studies,[37]. A solution to the variation in outcome reporting across studies in TBPI is the development of a COS,[40]. This has been shown to improve consistency of outcome reporting,[41, 19]. Development of a COS in TBPI would not restrict the range of outcomes that can be measured. Researchers and clinicians would still be free to select additional outcomes but the inclusion of such a COS would facilitate synthesis of evidence,[42, 43]. Whilst work has begun in obstetric brachial plexus injuries to develop a minimum data set,[44] there is no COS for TBPI.

Considerable work has been done by the Core Outcome Measures in Effectiveness Trials (COMET) initiative through dissemination of resources for COS development and support for methodological development. COMET recommends a five step process to develop a COS: define the scope, assess the need, develop the protocol, determine what to measure and determine how to measure,[45]. This systematic review addresses these first two steps for the development of the COS in TBPI care. This review has shown the majority of TBPI studies use only clinician reported outcomes to evaluate interventions. However they do not adequately capture patients' health related quality of life,[46] and may underestimate the impact of a condition,[47]. Concurrent qualitative work to identify outcomes which are important to individuals with a TBPI has been completed by this group. The next stage

For peer review only - http://bmjopen.bmj.com/site/about/guidelines.xhtml

involves integration of all potential outcomes from this review and the qualitative work into a long list of domains. Healthcare professionals and patients will then prioritize these using a consensus process,[45]. This will strengthen the case for uptake of a COS for TBPI as it represents patients' and clinicians' perspectives on what outcomes are important.

For peer review only

For peer review only - http://bmjopen.bmj.com/site/about/guidelines.xhtml

CONCLUSION

This systematic review has shown that outcome reporting in TBPI care is heterogenous and impairment focused with a lack of standardized definitions for commonly reported outcomes. This makes it difficult to compare and combine data from studies to inform decision making in clinical practice. We have identified a list of potentially relevant outcomes and categorized these into a clear taxonomy. This will inform the next stage of developing a COS for TBPI where patients, surgeons and therapists will be involved in a consensus process to decide the final outcomes included in a COS for TBPI.

Acknowledgements

We would like to thank Colin Shirley for his assistance and guidance categorising neurophysiological outcomes

Competing Interests

Conflicting interests: CM, CJH, JC, DMP and JOS declare no potential conflicts of interest with respect to the research, authorship and publication of this article. DGK reports grants from NIHR, grants from Innovate UK, grants from NIHR Birmingham Biomedical Research Centre, grants from NIHR SRMRC at the University of Birmingham and University Hospitals Birmingham NHS Foundation Trust, personal fees from Merck, personal fees from GSK,

For peer review only

grants from Macmillan Cancer Support, grants from Kidney research UK, outside the submitted work; .

Funding

The work was supported by the National Institute of Health Research [grant number ICA-CDRF-2017-03-039]

Ethical approval

Ethical approval was not sought for the present study because it was a systematic review and did not involve human participation

Informed consent

Informed consent was not sought for the present study because it was a systematic review and did not involve human participation

For peer review only

Contributorship

CM, CJH and JC conceived and designed the review. CM and JOS reviewed the titles, abstracts and full text papers for eligibility. Authors resolved disagreements by discussion or where necessary CJH and DMP offered their view. CM and JOS were responsible for extracting data and data extraction was verified by CJH. CM, CJH and JC categorised outcomes. Categorisation was reviewed and edited by DMP and DK. CM prepared the manuscript. CJH,JC, DMP, DK and JOS reviewed and edited the manuscript.

For peer review only

REFERENCES

1 Brito S, White J, Thomacos N, et al. The lived experience following free functioning muscle transfer for management of pan-brachial plexus injury: reflections from a long-term follow-up study. *Disabil Rehabil* 2019;1:1-9.

2 Morris MT, Daluiski A, Dy CJ. A Thematic Analysis of Online Discussion Boards for Brachial Plexus Injury. *J Hand Surg Am* 2016;41:813-818.

3 Kaiser R, Waldauf P, Ullas G, et al. Epidemiology, etiology, and types of severe adult brachial plexus injuries requiring surgical repair: systematic review and meta-analysis. *Neurosurg Rev* 2020;43:443-452.

4 Moran CG, Lecky F, Bouamra O, et al. Changing the System - Major Trauma Patients and Their Outcomes in the NHS (England) 2008-17. *EClinicalMedicine* 2018;2-3:13-21.

5 Dy, C.J, Peacock, K, Olsen, MA, et al. Incidence of Surgically Treated Brachial Plexus Injury in Privately Insured Adults Under 65 Years of Age in the USA. *HSS Jrnl* 2020. Jan. https://doi.org/10.1007/s11420-019-09741-8 (accessed 12 September 2020).

6 Midha R. Epidemiology of brachial plexus injuries in a multitrauma population. *Neurosurgery*. 1997;40:1182-1189.

7 Felici N, Zaami S, Ciancolini G, et al. Cost analysis of brachial plexus injuries: variability of compensation by insurance companies before and after surgery. *Handchir Mikrochir Plast Chir*. 2014;46:85-89.

8 Hong TS, Tian A, Sachar R, et al. Indirect Cost of Traumatic Brachial Plexus Injuries in

For peer review only - http://bmjopen.bmj.com/site/about/guidelines.xhtml

the United States. *J Bone Joint Surg Am*. 2019;101:e80.

9 Bhandari PS, Maurya S. Recent advances in the management of brachial plexus injuries. Indian *J Plast Surg*. 2014;47:191-198.

10 Kubota, S, Hara Y, Shimizu Y, et al. (2017) A newly developed upper limb single-joint HAL in a patient with elbow flexion reconstruction after traumatic brachial plexus injury: A case report. *Interdisciplinary Neurosurgery.* 2017;10:66-68.

11 Dickinson HD. Evidence-based decision-making: an argumentative approach. *Int J Med Inform*. 1998;51:71-81.

12 Sackett DL, Rosenberg WM, Gray JA, et al. Evidence based medicine: what it is and what it isn't. *BMJ*. 1996;312:71-72.

13 Ayhan E, Soldado F, Fontecha CG, et al. Elbow flexion reconstruction with nerve transfer or grafting in patients with brachial plexus injuries: A systematic review and comparison study. *Microsurgery*. 2020;40:79-86.

14 Donnelly MR, Rezzadeh KT, Vieira D, et al. Is one nerve transfer enough? A systematic review and pooled analysis comparing ulnar fascicular nerve transfer and double ulnar and median fascicular nerve transfer for restoration of elbow flexion after traumatic brachial plexus injury. *Microsurgery*. 2020;40:361-369.

15 Dy CJ, Garg R, Lee SK, et al. A systematic review of outcomes reporting for brachial plexus reconstruction. *J Hand Surg Am*. 2015;40:308-313.

16 Hill B, Williams G, Olver J, et al. Letter Regarding Outcome Reporting for Brachial Plexus Reconstruction. *J Hand Surg Am*. 2015;40:1504.

17 Gargon E, Gurung B, Medley N, et al. Choosing Important Health Outcomes For Comparative Effectiveness Research: A Systematic Review. *Value Health*. 2014;17:A435.

For peer review only - http://bmjopen.bmj.com/site/about/guidelines.xhtml

18 Williamson P, Altman D, Blazeby J, Clarke M, Gargon E. Driving up the quality and relevance of research through the use of agreed core outcomes. *J Health Serv Res Policy*. 2012;17:1-2.

19

20 Kirkham JJ, Bracken M, Hind L, et al. Industry funding was associated with increased use of core outcome sets. *J Clin Epidemiol*. 2019;115:90-97.

21 Higgins JPT, Green S. *Cochrane Handbook for Systematic Reviews of Interventions*. 5th Edn. The Cochrane Collaboration 2011. Available from www.handbook.cochrane.org.

22 Shamseer L, Moher D, Clarke M, et al. Preferred reporting items for systematic review and meta-analysis protocols (PRISMA-P) 2015: elaboration and explanation. *BMJ*. 2015;350:7647.

23 Kirkham JJ, Gorst S, Altman DG, et al. Core Outcome Set-STAndards for Reporting: The COS-STAR Statement. *PLoS Med*. 2016;13: e1002148.

24 Cieza A, Geyh S, Chatterji S, et al. ICF linking rules: an update based on lessons learned. *J Rehabil Med.* 2005;37:212-218.

25 Dodd S, Clarke M, Becker L et al. A taxonomy has been developed for outcomes in medical research to help improve knowledge discovery. *J Clin Epidemiol*. 2018,96: 84-92.

26 Young AE, Brookes ST, Avery KNL et al. A systematic review of core outcome set development studies demonstrates difficulties in defining unique outcomes. *J Clin Epidemiol*. 2019a;115:14-24.

27 Hudak PL, Amadio PC, Bombardier C. Development of an upper extremity outcome measure: the DASH (disabilities of the arm, shoulder and hand). *Am J Ind Med*. 1996; 29:602-608.

For peer review only - http://bmjopen.bmj.com/site/about/guidelines.xhtml

28 Baker CM, Wong DL. Q.U.E.S.T.: a process of pain assessment in children (continuing education credit). Orthop Nurs. 1987; 6:11-21.

29 Gallagher P, MacLachlan M. Development and psychometric evaluation of the trinity amputation and prosthesis experience scales (TAPES). *Rehab Psychology*. 2000;45:130–154.

30 Committee Medical. Medical Research Council [Great Britain]. Nerve Injuries Committee. Aids to the investigation of the peripheral nervous system. HM Stationary Office, 1942.

31 Conboy VB, Morris RW, Kiss J et al. An evaluation of the Constant-Murley shoulder assessment. *J Bone Joint Surg Br*. 1996;78:229-232.

32 Chalmers I, Glasziou P. Avoidable waste in the production and reporting of research evidence. *Lancet*. 2009;374:86-89.

33 McDonald J, Pettigrew J. Traumatic brachial plexus injury: the lived experience. *The British Journal of Occupational Therapy*. 2014;3:147-154.

34 Kim JH, Shin SH, Lee YR, et al. Ultrasound-guided peripheral nerve stimulation for neuropathic pain after brachial plexus injury: two case reports. *J Anesth.* 2017;31:453-457.

35 Miller C, Power D, Peek L et al. Psychological consequences of traumatic upper limb peripheral nerve injury: A systematic review. *Hand Therapy*. 2017;22:35–45.

36 Clavien PA, Sanabria JR, Strasberg SM. Proposed classification of complications of surgery with examples of utility in cholecystectomy. *Surgery*. 1992; 111:518-526.

37 Martins RS, Siqueira MG, Heise CO. A prospective study comparing single and double fascicular transfer to restore elbow flexion after brachial plexus injury. *Neurosurgery*. 2013; 72:709-715.

For peer review only - http://bmjopen.bmj.com/site/about/guidelines.xhtml

38 Young AE, Davies A, Bland S, et al. Systematic review of clinical outcome reporting in randomized controlled trials of burn care. *BMJ Open;* 2019b, 9: e025135.

39 Potter S, Brigic A, Whiting PF, et al. Reporting clinical outcomes of breast reconstruction: a systematic review. *J Natl Cancer Inst.* 2011;103:31-46.

40 Watzlawick R, Antonic A, Sena ES, et al. Outcome heterogeneity and bias in acute experimental spinal cord injury: A meta-analysis. *Neurology*. 2019;93:e40-e51.

41 Williamson PR, Altman DG, Blazeby JM et al. The COMET (Core Outcome Measures in Effectiveness Trials) Initiative. *Trials.* 2011;12:A70.

42 Bautista-Molano W, Navarro-Compán V, Landewé RB, et al. How well are the ASAS/OMERACT Core Outcome Sets for Ankylosing Spondylitis implemented in randomized clinical trials? A systematic literature review. *Clin Rheumatol*. 2014;33: 1313-1322.

43 Clarke M. Standardizing outcomes for clinical trials and systematic reviews. *Trials.* 2007;8:39.

44 Clarke M, Williamson PR. Core outcome sets and systematic reviews. *Syst Rev*. 2016;5:11.

45 Pondaag W, Malessy MJA. Outcome assessment for Brachial Plexus birth injury. Results from the iPluto world-wide consensus survey. *J Orthop Res*. 2018;36:2533-2541.

46 Williamson PR, Altman DG, Bagley H, et al. The COMET Handbook: version 1.0. Trials. 2017;18(Suppl 3):280.

47 Pakhomov SV, Jacobsen SJ, Chute CG, et al. Agreement between patient-reported symptoms and their documentation in the medical record. *Am J Manag Care*. 2008;14:530-539.

For peer review only

For peer review only - http://bmjopen.bmj.com/site/about/guidelines.xhtml

48 Turner GM, Slade A, Retzer A, et al. An introduction to patient-reported outcome measures (PROMs) in trauma. *J Trauma Acute Care Surg*. 2019;86:314-320.

Figure 2 Legends

mths, months; NS, not stated; yrs, years.

For peer review only

Title: Supplementary File 1 MEDLINE (OVID) search strategy

Article title: Developing a core outcome set for Traumatic Brachial Plexus Injuries: a systematic review of outcomes
Author: Miller et al (2020)

Search strategy 18/09/2018 COMBINE systematic review

MEDLINE (OVID)

1.(brachial plexus adj3 injur*).mp. [mp=title, abstract, original title, name of substance word, subject heading word, floating sub-heading word, keyword heading word, protocol supplementary concept word, rare disease supplementary concept word, unique identifier, synonyms]

2 (brachial plexus adj3 pals*).mp. [mp=title, abstract, original title, name of substance word, subject heading word, floating sub-heading word, keyword heading word, protocol supplementary concept word, rare disease supplementary concept word, unique identifier, synonyms]

3 (brachial plexus adj3 lesion*).mp. [mp=title, abstract, original title, name of substance word, subject heading word, floating sub-heading word, keyword heading word, protocol supplementary concept word, rare disease supplementary concept word, unique identifier, synonyms]

4 brachial plexopath*.mp. [mp=title, abstract, original title, name of substance word, subject heading word, floating sub-heading word, keyword heading word, protocol supplementary concept word, rare disease supplementary concept word, unique identifier, synonyms]

5 (brachial plexus adj3 traction*).mp. [mp=title, abstract, original title, name of substance word, subject heading word, floating sub-heading word, keyword heading word, protocol supplementary concept word, rare disease supplementary concept word, unique identifier, synonyms]

6 (brachial plexus adj3 avulsion*).mp. [mp=title, abstract, original title, name of substance word, subject heading word, floating sub-heading word, keyword heading word, protocol supplementary concept word, rare disease supplementary concept word, unique identifier, synonyms]

7 Brachial Plexus/in, su, tr [Injuries, Surgery, Transplantation]

8 1 or 2 or 3 or 4 or 5 or 6 or 7

9 limit 8 to (humans and "all adult (19 plus years)")

10. limit 9 to yr= “2013- current”

For peer review only

Supplementary file 2: COMET outcome taxonomy
Article title: Developing a core outcome set for Traumatic Brachial Plexus Injuries: a systematic review of outcomes

Title: Supplementary file 2: COMET outcome taxonomy - adapted from Dodd et al (2018)

**Core Area**	Outcome Domain
**Death**	1. Mortality/ survival
**Physiological/clinical**	2. Blood and lymphatic system outcomes
	3. Cardiac outcomes
	4. Congenital, familial and genetic outcomes
	5. Endocrine outcomes
	6. Ear and labyrinth outcomes
	7. Eye outcomes
	8. Gastrointestinal outcomes
	9. General outcomes
	10. Hepatobilary outcomes
	11. Immune system outcomes
	12. Infection and infestation outcomes
	13. Injury and poisoning outcomes
	14. Metabolism and nutrition outcomes
	15. Musculoskeletal and connective tissue outcomes
	16. Outcomes, relating to neoplasms: benign, malignant and unspecified (including cysts and polyps)
	17. Nervous system outcomes
	18. Pregnancy, puerperium and perinatal outcomes
	19. Renal and urinary outcomes
	20. Reproductive system and breast outcomes
	21. Psychiatric outcomes
	22. Respiratory, thoracic and mediastinal outcomes
	23. Skin and subcutaneous tissue outcomes
	24. Vascular outcomes
**Life Impact**	**Functioning**
	25. Physical functioning
	26. Social functioning
	27. Role functioning
	28. Emotional functioning/ well being
	29. Cognitive functioning
	30. Global quality of life
	31. Perceived health status
	32. Delivery of care
	33. Personal circumstances
**Resource use**	**Resource Use**
	34. Economic
	35. Hospital
	36. Need for further intervention
	37. Societal/ carer burden
**Adverse Events**	38. Adverse Events / effects

Dodd S, Clarke M, Becker L et al. A taxonomy has been developed for outcomes in medical research to help improve knowledge discovery. *J Clin Epidemiol*. 2018;96:84-92.

For peer review only

Supplementary file 3. Included Studies

	Study title	First author	Year of publication
**1**	Effectiveness and safety of home-based muscle electrical stimulator in brachial plexus Injury patient(Limthongthang et al., 2014)	Limthongthang	2014
**2**	Elbow proprioception sense in total arm -type brachial plexus injured patients after neurotisation: a preliminary study(Homsreprasert et al., 2014)	Homreprasert	2014
**3**	Comparison between the anterior and posterior approach for transfer of the spinal accessory nerve to the suprascapular nerve in late traumatic brachial plexus injuries (Souza et al., 2014)	Souza	2014
**4**	Ultrasound-guided peripheral nerve stimulation for neuropathic pain after brachial plexus injury: two case reports(Kim et al., 2017)	Kim	2017
**5**	Contralateral lower trapezius transfer for restoration of shoulder external rotation in traumatic brachial plexus palsy: preliminary report and literature review(Satbhai et al., 2014)	Satbhai	2014
**6**	Restoration of shoulder abduction in brachial plexus avulsion injuries with double neurotization from the spinal accessory nerve: a report of 13 cases(Huan et al., 2017)	Huan	2017
**7**	Transfer of the musculocutaneous nerve branch to the brachialis muscle to the triceps for elbow extension: anatomical study and report of five cases(Bertelli et al., 2017)	Bertelli	2017
**8**	Posterior approach for accessory to suprascapular nerve transfer: an electrophysiological outcomes study(Rui et al., 2013)	Rui	2013
**9**	Reliability of functioning free muscle transfer and vascularized ulnar nerve grafting for elbow flexion in complete brachial plexus palsy (Potter and Ferris, 2017)	Potter	2017
**10**	Management of infraclavicular (Chuang Level IV) brachial plexus injuries: A single surgeon experience with 75 cases (Lam et al., 2015)	Lam	2015
**11**	Functioning free muscle transfer for the restoration of elbow flexion in brachial plexus injury patients (Estrella and Montales 2016)	Estrella	2016
**12**	Radial to axillary nerve transfers: A combined case series(Desai et al., 2016)	Desai	2016
**13**	Thalamic deep brain stimulation for neuropathic pain after amputation or brachial plexus avulsion(Pereira et al., 2013)	Pereira	2013
**14**	Nerve transfers for shoulder function for traumatic brachial plexus injuries(Estrella et al., 2014)	Estrella	2014
**15**	Results of operative treatment of brachial plexus injury resulting from shoulder dislocation: A study with a long-term follow-up(Gutkowska et al., 2017)	Gutkowska	2017
**16**	Surgical treatment of brachial plexus posterior cord lesion: A combination of nerve and tendon transfers, about nine patients(Oberlin., 2013)	Oberlin	2013
**17**	The medial cord to musculocutaneous (MCMc) nerve transfer: a new method to reanimate elbow flexion after C5-C6-C7-(C8) avulsive injuries of the brachial plexus—technique and results(Ferraresi et al., 2014)	Ferraresi	2014

Article title: Developing a core outcome set for Traumatic Brachial Plexus Injuries: a systematic review of outcomes

For peer review only - http://bmjopen.bmj.com/site/about/guidelines.xhtml

Supplementary file 3. Included Studies

**18**	Transfer of a terminal motor branch nerve to the flexor carpi ulnaris for triceps reinnervation: anatomical study and clinical cases(Bertelli et al., 2015)	Bertelli	2015
**19**	Free functioning gracilis muscle transfer with and without simultaneous intercostal nerve transfer to musculocutaneous nerve for restoration of elbow flexion after traumatic adult brachial pan-plexus injury(Maldonado et al., 2017a)	Maldonado	2017(a)
**20**	Isolated latissimus dorsi transfer to restore shoulder external rotation in adults with brachial plexus injury(Ghosh et al., 2013)	Ghosh	2013
**21**	Functional outcome and quality of life after traumatic total brachial plexus injury treated by nerve transfer or single/double free muscle transfers(Satbhai et al., 2016)	Satbhai	2016
**22**	Successful graded mirror therapy in a patient with chronic deafferentation pain in whom traditional mirror therapy was ineffective: A case report(Mibu et al., 2016)	Mibu	2016
**23**	Bipolar Transfer of Latissimus Dorsi Myocutaneous Flap for Restoration of Elbow Flexion in Late Traumatic Brachial Plexus Injury: Evaluation of 13 Cases(Azab et al., 2017)	Azab	2017
**24**	Comparison of objective muscle strength in C5-C6 and C5-C7 brachial plexus injury patients after double nerve transfer (Tsai et al. 2015)	Tsai	2014
**25**	Phantom remodeling effect of dorsal root entry zone lesioning in phantom limb pain caused by brachial plexus avulsion(Son et al., 2015)	Son	2015
**26**	Comparison of surgical strategies between proximal nerve graft and/or nerve transfer and distal nerve transfer based on functional restoration of elbow flexion: A retrospective review of 147 patients(Hu et al., 2018)	Hu	2018
**27**	Reconstruction of shoulder abduction by multiple nerve fascicle transfer through posterior approach(Ren et al., 2013)	Ren	2013
**28**	Intercostal nerve transfer to neurotize the musculocutaneous nerve after traumatic brachial plexus avulsion: A comparison of two, three, and four nerve transfers(Xiao et al., 2014)	Xiao	2014
**29**	Use of the DEKA Arm for amputees with brachial plexus injury: A case series(Resnik et al., 2017)	Resnik	2017
**30**	Polyester tape scapulopexy for chronic upper extremity brachial plexus injury(Leechavengvongs et al., 2015)	Leechavengvongs	2015
**31**	Contralateral C7 nerve transfer with direct coaptation to restore lower trunk function after traumatic brachial plexus avulsion(Wang et al., 2013)	Wang	2013
**32**	Outcome of surgical reconstruction after traumatic total brachial plexus palsy(Dodakundi et al., 2013)	Dodakundi	2013
**33**	Bionic reconstruction to restore hand function after brachial plexus injury: a case series of three patients(Aszmann et al., 2015)	Aszmann	2015
**34**	Surgical treatment of the plexus brachialis injury using long-lasting electrostimulation (Tsymbaliuk and Tretiak, 2013)	Tsymbalyuk	2013
**35**	Phrenic nerve transfer for reconstruction of elbow extension in severe brachial plexus injuries(Flores and Socolovsky, 2016)	Flores	2016

Article title: Developing a core outcome set for Traumatic Brachial Plexus Injuries: a systematic review of outcomes

For peer review only - http://bmjopen.bmj.com/site/about/guidelines.xhtml

Supplementary file 3. Included Studies

**36**	Direct coaptation of the phrenic nerve with the posterior division of the lower trunk to restore finger and elbow extension function in patients with total brachial plexus injuries(Wang et al., 2016)	Wang	2016
**37**	A prospective study comparing single and double fascicular transfer to restore elbow flexion after brachial plexus injury(Martins et al., 2013)	Martins	2013
**38**	Chronic post-traumatic neuropathic pain of brachial plexus and upper limb: a new technique of peripheral nerve stimulation(Stevanato et al., 2014)	Stevanato	2014
**39**	Effectiveness of contralateral C7 nerve root and multiple nerve transfer for treatment of brachial plexus root avulsion(Wei et al., 2014)	Wei	2014
**40**	Combined proximal nerve graft and distal nerve transfer for a posterior cord brachial plexus injury(Plate et al., 2013)	Plate	2013
**41**	The role of elective amputation in patients with traumatic brachial plexus injury(Maldonado et al., 2016b)	Maldonado	2016
**42**	Early microsurgical management of clavicular fracture combined with brachial plexus injury(Liu et al., 2014)	Liu	2014(a)
**43**	Contralateral trapezius transfer to restore shoulder external rotation following adult brachial plexus injury (Elhassan et al., 2016)	Elhassan	2016
**44**	Comparative study of phrenic nerve transfers with and without nerve graft for elbow flexion after global brachial plexus injury(Liu et al., 2014)	Liu	2014
**45**	Shoulder and elbow recovery at 2 and 11 years following brachial plexus reconstruction(Wang et al., 2016)	Wang	2016
**46**	Functional outcomes after treatment of traumatic brachial plexus injuries: clinical study(Aras et al., 2013)	Aras	2013
**47**	Free gracilis transfer reinnervated by the nerve to the supinator for the reconstruction of finger and thumb extension in longstanding C7-T1 brachial plexus root avulsion(Soldado et al., 2013)	Soldado	2013
**48**	Restoration of hand function in C7–T1 brachial plexus palsies using a staged approach with nerve and tendon transfer(Zhang et al., 2014)	Zhang	2014
**49**	Neurotization to innervate the deltoid and biceps: 3 cases(Dy et al., 2013)	Dy	2013
**50**	Arthroscopic arthrodesis of the shoulder in brachial plexus palsy(Lenoir et al., 2017)	Lenoir	2017
**51**	Outcome of contralateral C7 nerve transferring to median nerve(Kai-ming Gao et al., 2013)	Gao	2013
**52**	Intercostal nerve transfer to the biceps motor branch in complete traumatic brachial plexus injuries (Cho et al., 2015)	Cho	2015
**53**	Tactile feedback for relief of deafferentation pain using virtual reality system: a pilot study(Sano et al., 2016)	Sano	2016
**54**	Functioning free gracilis transfer to reconstruct elbow flexion and quality of life in global brachial plexus injured patients(Yang et al., 2016)	Yang	2016

Article title: Developing a core outcome set for Traumatic Brachial Plexus Injuries: a systematic review of outcomes

For peer review only - http://bmjopen.bmj.com/site/about/guidelines.xhtml

Supplementary file 3. Included Studies

**55**	Evaluation of infraspinatus reinnervation and function following spinal accessory nerve to suprascapular nerve transfer in adult traumatic brachial plexus injuries(Baltzer et al., 2017)	Baltzer	2017
**56**	Anatomic study of the intercostal nerve transfer to the suprascapular nerve and a case report(Hu et al., 2014)	Hu	2014
**57**	Shoulder abduction and external rotation restoration with nerve transfer(Kostas-Agnantis et al., 2013)	Kostas-Agnantis	2013
**58**	Contralateral C-7 transfer: is direct repair really superior to grafting?(Bhatia et al., 2017)	Bhatia	2017
**59**	Impact of phrenic nerve paralysis on the surgical outcome of intercostal nerve transfer(Kita et al., 2015)	Kita	2015
**60**	Flow-through anastomosis using a T-shaped vascular pedicle for gracilis functioning free muscle transplantation in brachial plexus injury(Hou et al., 2015)	Hou	2015
**61**	Free functional muscle transfer tendon insertion secondary advancement procedure to improve elbow flexion(Sechachalam et al., 2017)	Sechachalam	2017
**62**	Dual nerve transfers for restoration of shoulder function after brachial plexus avulsion injury(Chu et al., 2016)	Chu	2016
**63**	Cortical plasticity after brachial plexus injury and repair: a resting-state functional MRI study(Bhat et al., 2017)	Bhat	2017
**64**	Results of spinal accessory to suprascapular nerve transfer in 110 patients with complete palsy of the brachial plexus(Bertelli et al., 2016)	Bertelli	2016
**65**	Magnetic resonance neurographic and clinical long-term results after oberlins transfer for adult brachial plexus injuries(Frueh et al., 2017)	Frueh	2017
**66**	Free functioning gracilis muscle transfer versus intercostal nerve transfer to musculocutaneous nerve for restoration of elbow flexion after traumatic adult brachial pan-plexus injury(Maldonado et al., 2016a)	Maldonado	2016
**67**	Results of wrist extension reconstruction in C5–8 brachial plexus palsy by transferring the pronator quadratus motor branch to the extensor carpi radialis brevis muscle(Bertelli et al., 2016)	Bertelli	2016
**68**	Donor nerve sources in free functional gracilis muscle transfer for elbow flexion in adult brachial plexus injury(Nicoson et al., 2017)	Nicoson	2017
**69**	Use of contralateral spinal accessory nerve for ipsilateral suprascapular neurotization in global brachial plexus injury: a new technique(Bhandari and Deb, 2016)	Bhandari	2016
**70**	Objective evaluation of elbow flexion strength and fatigability after nerve transfer in adult traumatic brachial plexus injuries (Maricq et al., 2014)	Marciq	2014
**71**	Outcomes of muscle brachialis transfer to restore finger flexion in brachial plexus palsy(DeGeorge et al., 2017)	DeGeorge	2017
**72**	Functional outcome of nerve transfers for traumatic global brachial plexus avulsion(Liu et al., 2013)	Liu	2013

Article title: Developing a core outcome set for Traumatic Brachial Plexus Injuries: a systematic review of outcomes

For peer review only - http://bmjopen.bmj.com/site/about/guidelines.xhtml

Supplementary file 3. Included Studies

**73**	Transfer of a flexor digitorum superficialis motor branch for wrist extension reconstruction in C5-C8 root injuries of the brachial plexus: a case series(Bertelli and Ghizoni, 2013)	Bertelli	2013
**74**	Outcome after transfer of intercostal nerves to the nerve of triceps long head in 25 adult patients with total plexus root avulsion injury(KaiMing Gao et al., 2013)	Gao	2013
**75**	Good sensory recovery of the hand in brachial plexus surgery using the intercostobrachial nerve as the donor(Foroni et al., 2017)	Foroni	2017
**76**	The phrenic nerve as a donor for brachial plexus injuries: is it safe and effective? Case series and literature analysis(Socolovsky et al., 2015)	Socolovsky	2015
**77**	Complete avulsion of brachial plexus with associated vascular trauma: Feasibility of reconstruction using the double free muscle technique(Hattori et al., 2013)	Hattori	2013
**78**	Long-term outcome of brachial plexus re-implantation after complete brachial plexus avulsion injury(Kachramanoglou et al., 2017)	Kachramanoglo u	2017
**79**	Force recovery assessment of functioning free muscle transfers using ultrasonography(Kodama et al., 2014)	Kodama	2014
**80**	Rhomboid nerve transfer to the suprascapular nerve for shoulder reanimation in brachial plexus palsy: A clinical report(Goubier and Teboul, 2016)	Goubier	2016
**81**	Outcome of contralateral C7 transfer to two recipient nerves in 22 patients with the total brachial plexus avulsion injury(Kaiming et al., 2013)	Gao	2013
**82**	Comparative study of phrenic and intercostal nerve transfers for elbow flexion after global brachial plexus injury(Yuzhou et al., 2015)	Liu	2015
**83**	Donor-side morbidity after contralateral C-7 nerve transfer: results at a minimum of 6 months after surgery(Li et al., 2016)	Li	2016
**84**	Outcome after brachial plexus injury surgery and impact on quality of life(Rasulic et al., 2017)	Rasulić	2017
**85**	Pronator teres branch transfer to the anterior interosseous nerve for treating C8T1 brachial plexus avulsion: An anatomic study and case report(Yang et al., 2014)	Yang	2014
**86**	Operative treatment with nerve repair can restore function in patients with traction injuries in the brachial plexus(Stiasny et al., 2015)	Stiasny	2015
**87**	Thoracodorsal nerve transfer for triceps reinnervation in partial brachial plexus injuries(Soldado et al., 2016)	Soldado	2016
**88**	Co-infusion of autologous adipose tissue derived neuronal differentiated mesenchymal stem cells and bone marrow derived hematopoietic stem cells, a viable therapy for post-traumatic brachial plexus injury: a case report (Thakkar et al., 2014)	Thakkar	2014
**89**	Long-term clinical outcomes of spinal accessory nerve transfer to the suprascapular nerve in patients with brachial plexus palsy(Emamhadi et al., 2016)	Emamhadi	2016
**90**	Surgical treatment for total root avulsion type brachial plexus injuries by neurotisation: a prospective comparison study between total and hemicontralateral C7 nerve root transfer(Tu et al., 2014)	Tu	2014

Article title: Developing a core outcome set for Traumatic Brachial Plexus Injuries: a systematic review of outcomes

For peer review only - http://bmjopen.bmj.com/site/about/guidelines.xhtml

Supplementary file 3. Included Studies

**91**	Deactivation of distant pain-related regions induced by 20-day rTMS: a case study of one-week pain relief for long-term intractable deafferentation pain (Qiu et al., 2014)	Qiu	2014
**92**	End-to-side neurorrhaphy in brachial plexus reconstruction(Haninec et al., 2013)	Haninec	2013
**93**	Reanimation of elbow extension with medial pectoral nerve transfer in partial injuries to the brachial plexus (Flores., 2013)	Flores	2013
**94**	Early post-operative results after repair of traumatic brachial plexus palsy(Mohammad-Reda., 2013)	Mohammad-Reda	2013
**95**	Satisfied patients after shoulder arthrodesis for brachial plexus lesions even after 20 years of follow-up(van der Lingen et al., 2018)	van der Lingen	2018
**96**	Posterior branch of the axillary nerve transfer to the lateral triceps branch for restoration of elbow extension: case report(Klika et al., 2013)	Kilka	2013
**97**	Clinical analysis of repairing the whole brachial plexus nerve root avulsion by transferring C7 nerve root from the uninjured side(Liu et al., 2014)	Liu	2014
**98**	Bipolar transfer of the pectoralis major muscle for restoration of elbow flexion in 29 cases(Cambon-Binder et al., 2018)	Cambon-Binder	2018
**99**	Thoracodorsal nerve transfer for elbow flexion reconstruction in infraclavicular brachial plexus injuries(Soldado et al., 2014)	Soldado	2014
**100**	Median nerve fascicle transfer versus ulnar nerve fascicle transfer to the biceps motor branch in C5-C6 and C5-C7 brachial plexus injuries: nonrandomised prospective study of 23 consecutive patients(Cho et al., 2014)	Cho	2014
**101**	Free functional muscle transplantation of an anomalous femoral adductor with a very large muscle belly: a case report(Kaizawa et al., 2013)	Kaizawa	2013
**102**	Selective neurotisation of the radial nerve in the axilla using the intercostal nerve to treat complete brachial plexus palsy(Tuohuti et al., 2016)	Tuohuti	2016
**103**	Objective predictors of functional recovery associated with intercostal nerves transfer for triceps reinnervation in global brachial plexus palsy(Flores., 2016)	Flores	2016
**104**	Nerve transfer to relieve pain in upper brachial plexus injuries: does it work? (Emamhadi., 2017)	Emamhadi	2017
**105**	Phrenic nerve transfer versus intercostal nerve transfer for the repair of brachial plexus root avulsion injuries(Abdixbir et al., 2016)	Abdixbir	2016
**106**	End-to-side neurorrhaphy to restore elbow flexion in brachial plexus injury(Limthongthang et al., 2016)	Limthongthang	2016
**107**	Chordata method combined with electrotherapy in functional recovery after brachial plexus injury:report of three clinical cases(De Oliveira et al., 2016)	De Oliveira	2016
**108**	Clinical outcome following transfer of the supinator motor branch to the posterior interosseous nerve in patients with C7-T1 brachial plexus palsy(Xu et al., 2015)	Xu	2015

Article title: Developing a core outcome set for Traumatic Brachial Plexus Injuries: a systematic review of outcomes

For peer review only - http://bmjopen.bmj.com/site/about/guidelines.xhtml

Supplementary file 3. Included Studies

**109**	Transposition of branches of radial nerve innervating supinator to posterior interosseous nerve for functional reconstruction of finger and thumb extension in 4 patients with middle and lower trunk root avulsion injuries of brachial plexus(Wu et al., 2017)	Wu	2017
**110**	Electromyographic findings in gracilis muscle grafts used to augment elbow flexion in traumatic brachial plexopathy(Kazamel and Sorenson, 2016)	Kazamel	2016
**111**	Double distal intraneural fascicular nerve transfers for lower brachial plexus injuries(Li et al., 2016)	Li	2016
**112**	Restoration of elbow and hand function in total brachial plexus palsy with intercostal nerves and C5 root neurotisation. Results in 21 patients(Arnal et al., 2016)	Amal	2016
**113**	The phrenic nerve transfer in the treatment of a septuagenarian with brachial plexus avulsion injury: a case study(Jiang and Lao, 2018)	Jiang	2018
**114**	Outcomes of transferring a healthy motor fascicle from the radial nerve to a branch for the triceps to recover elbow extension in partial brachial plexus palsy(Flores., 2017)	Flores	2017
**115**	Successful nerve transfers for traumatic brachial plexus palsy in a septuagenarian(Johnsen and Wolfe, 2016)	Johnsen	2016
**116**	Free functioning gracilis muscle transfer for elbow flexion reconstruction after traumatic brachial pan-plexus injury: Where is the optimal distal tendon attachment for elbow flexion?(Maldonado et al., 2017b)	Maldonado	2017(b)
**117**	Results of distal nerve transfers in restoration of shoulder function in C5 and C6 root avulsion injury to the brachial plexus (Bhandari., 2017)	Bhandari	2017
**118**	Bipolar dual-lead spinal cord stimulation between two electrodes on the ventral and dorsal sides of the spinal cord: consideration of putative mechanisms(Watanabe et al., 2018)	Watanabe	2018
**119**	Triceps nerve to deltoid nerve transfer after an unsatisfactory intra-plexus neurotisation of the posterior division of the upper trunk(Al-Qattan et al., 2017)	Al-Qattan	2017
**120**	Trapezius muscle transfer for restoration of elbow extension in a traumatic brachial plexus injury(Alrabai et al., 2018)	Alrabai	2018
**121**	Transfer of the radial nerve branch to the extensor carpi radialis brevis to the anterior interosseous nerve to reconstruct thumb and finger flexion(Bertelli., 2015)	Bertelli	2015
**122**	Ultrasound-guided pulse-dose radiofrequency: treatment of neuropathic pain after brachial plexus lesion and arm vascularisation(Magistroni et al., 2014)	Magistroni	2014
**123**	Phrenic nerve transfer to the musculocutaneous nerve for the repair of brachial plexus injury: electrophysiological characteristics(Liu et al., 2015)	Liu	2015
**124**	Postoperative motor deficits following elbow flexion reanimation by nerve transfer(Hanneur et al., 2018)	Hanneur	2018

Article title: Developing a core outcome set for Traumatic Brachial Plexus Injuries: a systematic review of outcomes

For peer review only - http://bmjopen.bmj.com/site/about/guidelines.xhtml

Supplementary file 3. Included Studies

**125**	Comparative study of phrenic and partial ulnar nerve transfers for elbow flexion after upper brachial plexus avulsion-a retrospective clinical analysis(Liu et al., 2018)	Liu	2018
**126**	Contralateral medial pectoral nerve transfer with free gracilis muscle transfer in old brachial plexus injury(Yavari et al., 2018)	Yavari	2018
**127**	MEG-BMI to control phantom limb pain(Yanagisawa et al., 2018)	Yanagisawa	2018
**128**	Complete brachial plexus injury- an amputation dilemma, A case report(Choong and Shalimar, 2015)	Choong	2015
**129**	Reversal of phantom pain and hand-to-face remapping after brachial plexus avulsion(Tsao and Finn, 2016)	Tsao	2016
**130**	A newly developed upper limb single-joint HAL in a patient with elbow flexion reconstruction after traumatic brachial plexus injury: A case report(Kubota et al., 2017)	Kubota	2017
**131**	Free reverse gracilis muscle combined with steindler flexorplasty for elbow flexion reconstruction after failed primary repair of extended upper-type paralysis of the brachial plexus(Bertelli., 2018)	Bertelli	2018
**132**	Multiple nerve and tendon transfers – a new strategy for restoring hand function in a patient with C7-T1 brachial plexus avulsions(Xu et al ., 2017)	Xu	2017

For peer review only

Article title: Developing a core outcome set for Traumatic Brachial Plexus Injuries: a systematic review of outcomes

For peer review only - http://bmjopen.bmj.com/site/about/guidelines.xhtml

Supplementary file 3. Included Studies

References

[1] Limthongthang R, Muennoi P, Phoojaroenchanachai R, Vathana T, Wongtrakul S. Effectiveness and safety of home-based muscle electrical stimulator in brachial plexus injury patients. J Med Assoc Thail. 2014, 97: S56–61.

[2] Homsreprasert T, Limthongthang R, Vathana T, Wongtrakul S. Elbow joint proprioceptive sense in total arm-type brachial plexus injured patients after neurotization: a preliminary study. J Med Assoc Thail. 2014, 97: S103–7.

[3] Souza F, Bernardino S, Filho H, Gobbato PL, Martins HAL, Martins RS, et al. Comparison between the anterior and posterior approach for transfer of the spinal accessory nerve to the suprascapular nerve in late traumatic brachial plexus injuries. Acta Neurochir (Wien) 2014, 156: 2345–9.

[4] Kim JH, Shin SH, Lee YR, Lee HS, Chon JY, Sung CH, et al. Ultrasound-guided peripheral nerve stimulation for neuropathic pain after brachial plexus injury: two case reports. J Anesth. 2017, 31: 453–7.

[5] Satbhai NG, Doi K, Hattori Y, Sakamoto S. Contralateral lower trapezius transfer for restoration of shoulder external rotation in traumatic brachial plexus palsy: a preliminary report and literature review. J Hand Surg Eur. 2014, 39: 861–7.

[6] Huan KWSJ, Tan JSW, Tan SH, Teoh LC, Yong FC. Restoration of shoulder abduction in brachial plexus avulsion injuries with double neurotization from the spinal accessory nerve: a report of 13 cases. J Hand Surg Eur. 2017, 42: 700–5.

[7] Bertelli JA, Soldado F, Ghizoni MF, Rodriguez-Baeza A, J.A. B, F. S, et al. Transfer of the musculocutaneous nerve branch to the brachialis muscle to the triceps for elbow extension: anatomical study and report of five cases. J Hand Surg Eur. 2017, 42: 710–4.

[8] Rui J, Zhao X, Zhu Y, Gu Y, Lao J, J. R, et al. Posterior approach for accessory-suprascapular nerve transfer: an electrophysiological outcomes study. J Hand Surg Eur, 2013, 38: 242–7.

[9] Potter SM, Ferris SI. Reliability of functioning free muscle transfer and vascularized ulnar nerve grafting for elbow flexion in complete brachial plexus palsy. J Hand Surg Eur Vol 2017, 42: 693–9.

[10] Lam WL, Fufa D, Chang N-J, Chuang DC-C. Management of infraclavicular (Chuang Level IV) brachial plexus injuries: A single surgeon experience with 75 cases. J Hand Surg Eur. 2015, 40: 573–82.

[11] Estrella EP, Montales TD. Functioning free muscle transfer for the restoration of elbow flexion in brachial plexus injury patients. Injury. 2016, 47: 2525–33.

[12] Desai MJ, Daly CA, Seiler JG 3rd, Wray WH 3rd, Ruch DS, Leversedge FJ, et al. Radial to Axillary Nerve Transfers: A Combined Case Series. J Hand Surg Am. 2016, 41: 1128–34.

[13] Pereira EAC, Boccard SG, Linhares P, Chamadoira C, Rosas MJ, Abreu P, et al. Thalamic deep brain stimulation for neuropathic pain after amputation or brachial plexus avulsion. Neurosurg Focus. 2013, 35:E7.

[14] Estrella EP, Favila AS Jr. Nerve transfers for shoulder function for traumatic brachial plexus injuries. J Reconstr Microsurg. 2014, 30: 59-64.

For peer review only - http://bmjopen.bmj.com/site/about/guidelines.xhtml

Supplementary file 3. Included Studies

[15] Gutkowska O, Martynkiewicz J, Mizia S, Bak M, Gosk J. Results of Operative Treatment of Brachial Plexus Injury Resulting from Shoulder Dislocation: A Study with A Long-Term Follow-Up. World Neurosurg. 2017,105:623–31.

[16] Oberlin C, Chino J, Belkheyar Z. Surgical treatment of brachial plexus posterior cord lesion: A combination of nerve and tendon transfers, about nine patients. Chir Main. 2013, 32:141–6.

[17] Ferraresi S, Garozzo D, Basso E, Maistrello L, Lucchin F, Di Pasquale P. The medial cord to musculocutaneous (MCMc) nerve transfer: A new method to reanimate elbow flexion after C5-C6-C7-(C8) avulsive injuries of the brachial plexus - Technique and results. Neurosurg Rev. 2014, 37:321–9.

[18] Bertelli J, Soldado F, Ghizoni MF, Rodriguez-Baeza A, J. B, F. S, et al. Transfer of a terminal motor branch nerve to the flexor carpi ulnaris for triceps reinnervation: Anatomical study and clinical cases. J Hand Surg Am. 2015, 40:2229-2235.

[19] Maldonado AA, Kircher MF, Spinner RJ, Bishop AT, Shin AY. Free Functioning Gracilis Muscle Transfer With and Without Simultaneous Intercostal Nerve Transfer to Musculocutaneous Nerve for Restoration of Elbow Flexion After Traumatic Adult Brachial Pan-Plexus Injury. J Hand Surg Am 2017, 42: 293.

[20] Ghosh S, Singh VK, Jeyaseelan L, Sinisi M, Fox M, S. G, et al. Isolated latissimus dorsi transfer to restore shoulder external rotation in adults with brachial plexus injury. Bone Jt J. 2013, 95: 660–3.

[21] Satbhai NG, Doi K, Hattori Y, Sakamoto S, N.G. S, K. D, et al. Functional outcome and quality of life after traumatic total brachial plexus injury treated by nerve transfer or single/double free muscle transfers: A comparative study. Bone Jt J. 2016, 98: 209–17.

[22] Mibu A, Nishigami T, Tanaka K, Osumi M, Tanabe A. Successful Graded Mirror Therapy in a Patient with Chronic Deafferentation Pain in Whom Traditional Mirror Therapy was Ineffective: A Case Report. Pain Pract. 2016, 16: E62–9.

[23] Azab AA-H, Alsabbahi MS, A.A.-H. A, Azab AA-H, Alsabbahi MS. Bipolar Transfer of Latissimus Dorsi Myocutaneous Flap for Restoration of Elbow Flexion in Late Traumatic Brachial Plexus Injury: Evaluation of 13 Cases. Ann Plast Surg. 2017, 78: 198–201.

[24] Tsai Y-J, Su F-C, Hsiao C-K, Tu Y-K. Comparison of objective muscle strength in C5-C6 and C5-C7 brachial plexus injury patients after double nerve transfer. Microsurgery. 2015, 35: 107–14.

[25] Son BC, Ha SW. Phantom Remodeling Effect of Dorsal Root Entry Zone Lesioning in Phantom Limb Pain Caused by Brachial Plexus Avulsion. Stereotact Funct Neurosurg 2015, 93: 240–4.

[26] Hu CH, Chang TN, Lu JC, Laurence VG, Chuang DC. Comparison of Surgical Strategies between Proximal Nerve Graft and/or Nerve Transfer and Distal Nerve Transfer Based on Functional Restoration of Elbow Flexion: A Retrospective Review of 147 Patients. Plast Reconstr Surg. 2018, 141: 68e-79e.

[27] Ren G, Li R, Xiang D, Yu B. Reconstruction of shoulder abduction by multiple nerve fascicle transfer through posterior approach. Injury. 2013, 44: 492–7.

[28] Xiao C, Lao J, Wang T, Zhao X, Liu J, Gu Y. Intercostal nerve transfer to neurotize the musculocutaneous nerve after traumatic brachial plexus avulsion: a comparison of two, three, and four nerve transfers. J Reconstr Microsurg 2014, 30: 297–304.

[29] Resnik L, Fantini C, Latlief G, Phillips S, Sasson N, Sepulveda E, et al. Use of the DEKA Arm for

For peer review only - http://bmjopen.bmj.com/site/about/guidelines.xhtml

Supplementary file 3. Included Studies

amputees with brachial plexus injury: A case series. PLoS One. 2017, 12: e0178642.

[30] Leechavengvongs S, Jiamton C, Uerpairojkit C, Malungpaishorpe K, Witoonchart K, Poonotoke P, et al. Polyester tape scapulopexy for chronic upper extremity brachial plexus injury. J Hand Surg Am. 2015, 40:1184

[31] Wang S, Li P, Xue Y, Yiu H, Li YC, Wang H. Contralateral C7 nerve transfer with direct coaptation to restore lower trunk function after traumatic brachial plexus avulsion. J Bone Joint Surg Am. 2013, 95: 821–2.

[32] Dodakundi C, Doi K, Hattori Y, Sakamoto S, Fujihara Y, Takagi T, et al. Outcome of surgical reconstruction after traumatic total brachial plexus palsy. J Bone Joint Surg Am. 2013, 95:1505–12.

[33] Aszmann OC, Roche AD, Salminger S, Paternostro-Sluga T, Herceg M, Sturma A, et al. Bionic reconstruction to restore hand function after brachial plexus injury: a case series of three patients. Lancet. 2015, 385: 2183–9.

[34] Tsymbaliuk VI, Tretiak IB. [Surgical treatment of the plexus brachialis injury using long-lasting electrostimulation]. Klin Khirurhiia. 2013, 59–61.

[35] Flores LP, Socolovsky M. Phrenic Nerve Transfer for Reconstruction of Elbow Extension in Severe Brachial Plexus Injuries. J Reconstr Microsurg. 2016, 32: 546–50.

[36] Wang S, Li P, Xue Y, Zou J, Li W, Li Y. Direct Coaptation of the Phrenic Nerve With the Posterior Division of the Lower Trunk to Restore Finger and Elbow Extension Function in Patients With Total Brachial Plexus Injuries. Neurosurgery. 2016, 78: 208–14.

[37] Martins RS, Siqueira MG, Heise CO, Foroni L, Teixeira MJ. A prospective study comparing single and double fascicular transfer to restore elbow flexion after brachial plexus injury. Neurosurgery. 2013, 72: 709–15.

[38] Stevanato G, Devigili G, Eleopra R, Fontana P, Lettieri C, Baracco C, et al. Chronic post-traumatic neuropathic pain of brachial plexus and upper limb: a new technique of peripheral nerve stimulation. Neurosurg Rev. 2014, 37: 473–9.

[39] Wei W, Alimujiang-Abulaiti, Tuerxunjiang-Dadihan, Meihua S, Yafei L, Chunxiao Y, et al. [EFFECTIVENESS OF CONTRALATERAL C7 NERVE ROOT AND MULTIPLE NERVES TRANSFER FOR TREATMENT OF BRACHIAL PLEXUS ROOT AVULSION]. Zhongguo Xiu Fu Chong Jian Wai Ke Za Zhi. 2014, 28: 737–40.

[40] Plate JF, Ely LK, Pulley BR, Smith BP, Li Z. Combined proximal nerve graft and distal nerve transfer for a posterior cord brachial plexus injury. J Neurosurg. 2013, 118: 155–9.

[41] Maldonado AA, Kircher MF, Spinner RJ, Bishop AT, Shin AY. The role of elective amputation in patients with traumatic brachial plexus injury. J Plast Reconstr Aesthetic Surg. 2016, 69:311–7.

[42] Liu Y, Wang W, Regmi AM, Ahemaitijiang-Yusufu. Early microsurgical management of clavicular fracture combined with brachial plexus injury. Zhongguo Xiu Fu Chong Jian Wai Ke Za Zhi. 2014a,28: 1329–32.

[43] Elhassan BT, Wagner ER, Spinner RJ, Bishop AT, Shin AY. Contralateral Trapezius Transfer to Restore Shoulder External Rotation Following Adult Brachial Plexus Injury. J Hand Surg Am. 2016, 41: e45-51.

[44] Liu Y, Lao J, Gao K, Gu Y, Zhao X. Comparative study of phrenic nerve transfers with and

Article title: Developing a core outcome set for Traumatic Brachial Plexus Injuries: a systematic review of outcomes

For peer review only - http://bmjopen.bmj.com/site/about/guidelines.xhtml

Supplementary file 3. Included Studies

without nerve graft for elbow flexion after global brachial plexus injury. Injury. 2014b, 45: 227–31.

[45] Wang J-P, Rancy SK, Lee SK, Feinberg JH, Wolfe SW. Shoulder and Elbow Recovery at 2 and 11 Years Following Brachial Plexus Reconstruction. J Hand Surg Am. 2016, 41:173–9.

[46] Aras Y, Aydoseli A, Sabanci PA, Akcakaya MO, Alkir G, Imer M. Functional outcomes after treatment of traumatic brachial plexus injuries: clinical study. Ulus Travma Acil Cerrahi Derg. 2013, 19: 521–8.

[47] Soldado F, Bertelli J, F. S. Free gracilis transfer reinnervated by the nerve to the supinator for the reconstruction of finger and thumb extension in longstanding C7-T1 brachial plexus root avulsion. J Hand Surg Am. 2013, 38: 941–6.

[48] Zhang C-G, Dong Z, Gu Y-D. Restoration of hand function in C7-T1 brachial plexus palsies using a staged approach with nerve and tendon transfer. J Neurosurg. 2014, 121: 1264–70.

[49] Dy CJ, Kitay A, Garg R, Kang L, Feinberg JH, Wolfe SW, et al. Neurotization to innervate the deltoid and biceps: 3 Cases. J Hand Surg Am. 2013, 38:237–40.

[50] Lenoir H, Williams T, Griffart A, Lazerges C, Chammas M, Coulet B, et al. Arthroscopic arthrodesis of the shoulder in brachial plexus palsy. J Shoulder Elb Surg. 2017, 26: e115–21.

[51] Gao K, Lao J, Zhao X, Gu Y. Outcome of contralateral C7 nerve transferring to median nerve. Chin Med J (Engl). 2013, 126: 3865–8.

[52] Cho AB, Iamaguchi RB, Silva GB, Paulos RG, Kiyohara LY, Sorrenti L, et al. Intercostal nerve transfer to the biceps motor branch in complete traumatic brachial plexus injuries. Microsurgery. 2015, 35: 428–31.

[53] Sano Y, Wake N, Ichinose A, Osumi M, Oya R, Sumitani M, et al. Tactile feedback for relief of deafferentation pain using virtual reality system: a pilot study. J Neuroeng Rehabil. 2016, 13:61.

[54] Yang Y, Yang J-T, Fu G, Li X-M, Qin B-G, Hou Y, et al. Functioning free gracilis transfer to reconstruct elbow flexion and quality of life in global brachial plexus injured patients. Sci Rep. 2016, 6: 22479.

[55] Baltzer HL, Wagner ER, Kircher MF, Spinner RJ, Bishop AT, Shin AY, et al. Evaluation of infraspinatus reinnervation and function following spinal accessory nerve to suprascapular nerve transfer in adult traumatic brachial plexus injuries. Microsurgery. 2017, 37: 365–70.

[56] Hu S, Chu B, Song J, Chen L. Anatomic study of the intercostal nerve transfer to the suprascapular nerve and a case report. J Hand Surg Eur. 2014, 39: 194–8.

[57] Kostas-Agnantis I, Korompilias A, Vekris M, Lykissas M, Gkiatas I, Mitsionis G, et al. Shoulder abduction and external rotation restoration with nerve transfer. Injury. 2013, 44: 299–304.

[58] Bhatia A, Doshi P, Koul A, Shah V, Brown JM, Salama M, et al. Contralateral C-7 transfer: is direct repair really superior to grafting? Neurosurg Focus. 2017, 43: E3.

[59] Kita Y, Tajiri Y, Hoshikawa S, Hara Y, Iijima J. Impact of phrenic nerve paralysis on the surgical outcome of intercostal nerve transfer. Hand Surg. 2015, 20: 47–52.

[60] Hou Y, Yang J, Yang Y, Qin B, Fu G, Li X, et al. Flow-through anastomosis using a T-shaped vascular pedicle for gracilis functioning free muscle transplantation in brachial plexus injury. Clinics (Sao Paulo). 2015, 70: 544–9.

Article title: Developing a core outcome set for Traumatic Brachial Plexus Injuries: a systematic review of outcomes

For peer review only - http://bmjopen.bmj.com/site/about/guidelines.xhtml

Supplementary file 3. Included Studies

[61] Sechachalam S, O'Byrne A, MacQuillan A. Free Functional Muscle Transfer Tendon Insertion Secondary Advancement Procedure to Improve Elbow Flexion. Tech Hand Up Extrem Surg. 2017, 21: 8–12.

[62] Chu B, Wang H, Chen L, Gu Y, Hu S. Dual Nerve Transfers for Restoration of Shoulder Function After Brachial Plexus Avulsion Injury. Ann Plast Surg. 2016, 76: 668–73.

[63] Bhat DI, Indira Devi B, Bharti K, Panda R. Cortical plasticity after brachial plexus injury and repair: a resting-state functional MRI study. Neurosurg Focus. 2017, 42: E14.

[64] Bertelli JA, Ghizoni MF, J.A. B. Results of spinal accessory to suprascapular nerve transfer in 110 patients with complete palsy of the brachial plexus. J Neurosurg Spine. 2016, 24: 990–5.

[65] Frueh FS, Ho M, Schiller A, Ducommun P, Manoliu A, Andreisek G, et al. Magnetic Resonance Neurographic and Clinical Long-Term Results After Oberlin's Transfer for Adult Brachial Plexus Injuries. Ann Plast Surg. 2017, 78: 67–72.

[66] Maldonado AA, Kircher MF, Spinner RJ, Bishop AT, Shin AY. Free Functioning Gracilis Muscle Transfer versus Intercostal Nerve Transfer to Musculocutaneous Nerve for Restoration of Elbow Flexion after Traumatic Adult Brachial Pan-Plexus Injury. Plast Reconstr Surg. 2016, 138: 483e-488e.

[67] Bertelli JA, Ghizoni MF, Tacca CP. Results of wrist extension reconstruction in C5-8 brachial plexus palsy by transferring the pronator quadratus motor branch to the extensor carpi radialis brevis muscle. J Neurosurg. 2016, 124: 1442–9.

[68] Nicoson MC, Franco MJ, Tung TH. Donor nerve sources in free functional gracilis muscle transfer for elbow flexion in adult brachial plexus injury. Microsurgery. 2017, 37: 377–82.

[69] Bhandari PS, Deb P. Use of contralateral spinal accessory nerve for ipsilateral suprascapular neurotization in global brachial plexus injury: A new technique. J Neurosurg Spine. 2016, 24: 186–8.

[70] Maricq C, Jeunehomme M, Mouraux D, Remy P, Brassinne E, Bahm J, et al. Objective evaluation of elbow flexion strength and fatigability after nerve transfer in adult traumatic upper brachial plexus injuries. Hand Surg. 2014, 19: 335–41.

[71] DeGeorge B, Becker H, Faryna J, Spinner R, Bishop A. Outcomes of brachialis muscle transfer to restore finger flexion in traumatic lower trunk brachial plexus palsy. J Hand Surg Am. 2017, 42: S33–4.

[72] Liu Y, Lao J, Gao K, Gu Y, Zhao X. Functional outcome of nerve transfers for traumatic global brachial plexus avulsion. Injury. 2013, 44:655–60.

[73] Bertelli JA, Ghizoni MF. Transfer of a flexor digitorum superficialis motor branch for wrist extension reconstruction in C5-C8 root injuries of the brachial plexus: a case series. Microsurgery. 2013, 33:39–42.

[74] Gao K, Lao J, Zhao X, Gu Y. Outcome after transfer of intercostal nerves to the nerve of triceps long head in 25 adult patients with total brachial plexus root avulsion injury. J Neurosurg 2013, 118: 606–10.

[75] Foroni L, Siqueira MG, Martins RS, Heise CO, Sterman HN, Imamura AY. Good sensory recovery of the hand in brachial plexus surgery using the intercostobrachial nerve as the donor. Arq Neuropsiquiatr. 2017, 75: 796–800.

[76] Socolovsky M, di Masi G, Bonilla G, Dominguez Paez M, Robla J, Calvache Cabrera C, et al. The

For peer review only - http://bmjopen.bmj.com/site/about/guidelines.xhtml

Supplementary file 3. Included Studies

phrenic nerve as a donor for brachial plexus injuries: is it safe and effective? Case series and literature analysis. Acta Neurochir (Wien). 2015, 157: 1077–86.

[77] Hattori Y, Doi K, Sakamoto S, Satbhai NG. Complete avulsion of brachial plexus with associated vascular trauma: feasibility of reconstruction using the double free muscle technique. Plast Reconstr Surg. 2013, 132: 1504–12.

[78] Kachramanoglou C, Carlstedt T, Koltzenburg M, Choi D, C. K, T. C, et al. Long-Term Outcome of Brachial Plexus Reimplantation After Complete Brachial Plexus Avulsion Injury. World Neurosurg. 2017, 103: 28–36.

[79] Kodama N, Doi K, Hattori Y. Force recovery assessment of functioning free muscle transfers using ultrasonography. J Hand Surg Am. 2014, 39: 2269–76.

[80] Goubier J, Teboul F. Rhomboid nerve transfer to the suprascapular nerve for shoulder reanimation in brachial plexus palsy: A clinical report. Hand Surg Rehabil. 2016, 35: 363–6.

[81] Gao K, Lao J, Zhao X, Gu Y. Outcome of contralateral C7 transfer to two recipient nerves in 22 patients with the total brachial plexus avulsion injury. Microsurgery. 2013, 33: 605–11.

[82] Liu Y, Lao J, Zhao X. Comparative study of phrenic and intercostal nerve transfers for elbow flexion after global brachial plexus injury. Injury. 2015, 46: 671–5.

[83] Li XM, Yang JT, Hou Y, Yang Y, Qin BG, Fu G, et al. Donor-side morbidity after contralateral C-7 nerve transfer: results at a minimum of 6 months after surgery. J Neurosurg. 2016, 124: 1434–41

[84] Rasulic L, Savic A, Zivkovic B, Vitosevic F, Micovic M, Bascarevic V, et al. Outcome after brachial plexus injury surgery and impact on quality of life. Acta Neurochir (Wien) 2017, 159: 1257–64

[85] Yang J, Jia X, Yu C, Gu Y. Pronator teres branch transfer to the anterior interosseous nerve for treating C8T1 brachial plexus avulsion: An anatomic study and case report. Neurosurgery. 2014, 75: 375–9.

[86] Stiasny J, Birkeland P, J. S. Operative treatment with nerve repair can restore function in patients with traction injuries in the brachial plexus. Dan Med J. 2015, 62.

[87] Soldado F, Ghizoni MF, Bertelli J, F. S, M.F. G. Thoracodorsal nerve transfer for triceps reinnervation in partial brachial plexus injuries. Microsurgery. 2016, 36: 191–7.

[88] Thakkar UG, Vanikar A V, Trivedi HL. Co-infusion of autologous adipose tissue derived neuronal differentiated mesenchymal stem cells and bone marrow derived hematopoietic stem cells, a viable therapy for post-traumatic brachial plexus injury: a case report. Biomed J. 2014, 37: 237–40.

[89] Emamhadi M, Alijani B, Andalib S, M. E, B. A. Long-term clinical outcomes of spinal accessory nerve transfer to the suprascapular nerve in patients with brachial plexus palsy. Acta Neurochir (Wien). 2016, 158: 1801–6.

[90] Tu YK, Tsai YJ, Chang CH, Su FC, Hsiao CK, Tan JS. Surgical treatment for total root avulsion type brachial plexus injuries by neurotization: a prospective comparison study between total and hemicontralateral C7 nerve root transfer. Microsurgery. 2014, 34: 91–101.

[91] Qiu YQ, Hua XY, Zuo CT, Li T, Zheng MX, Shen YD, et al. Deactivation of distant pain-related regions induced by 20-day rTMS: a case study of one-week pain relief for long-term intractable deafferentation pain. Pain Physician. 2014, 17: E99–105.

Article title: Developing a core outcome set for Traumatic Brachial Plexus Injuries: a systematic review of outcomes

For peer review only - http://bmjopen.bmj.com/site/about/guidelines.xhtml

Supplementary file 3. Included Studies

[92] Haninec P, Mencl L, Kaiser R. End-to-side neurorrhaphy in brachial plexus reconstruction. J Neurosurg. 2013, 119: 689–94.

[93] Flores LP. Reanimation of elbow extension with medial pectoral nerve transfer in partial injuries to the brachial plexus. J Neurosurg. 2013, 118: 588–93.

[94] Mohammad-Reda A. Early post-operative results after repair of traumatic brachial plexus palsy. Turk Neurosurg. 2013, 23: 1–9.

[95] van der Lingen MAJ, de Joode SGCJ, Schotanus MGM, Grimm B, van Nie FA, Speth LAWM, et al. Satisfied patients after shoulder arthrodesis for brachial plexus lesions even after 20 years of follow-up. Eur J Orthop Surg Traumatol. 2018, 28: 1089–94

[96] Klika BJ, Spinner RJ, Bishop AT, Kircher MF, Shin AY. Posterior branch of the axillary nerve transfer to the lateral triceps branch for restoration of elbow extension: case report. J Hand Surg Am. 2013, 38: 1145–9.

[97] Liu J, Wang X, Zhang S, Wang L, Xia D, Gao Q, et al. A clinical analysis of repairing the whole brachial plexus nerve root avulsion by transferring C7 nerve root from the uninjured side. J Neurol Sci. 2014, 31: 521–31.

[98] Cambon-Binder A, Walch A, Marchei P, Belkheyar Z. Bipolar transfer of the pectoralis major muscle for restoration of elbow flexion in 29 cases. J Shoulder Elb Surg. 2018, 27: e330-e336

[99] Soldado F, Ghizoni MF, Bertelli J. Thoracodorsal nerve transfer for elbow flexion reconstruction in infraclavicular brachial plexus injuries. J Hand Surg Am. 2014, 39: 1766–70.

[100] Cho A, Paulos R, De Resende M, Kiyohara L, Sorrenti L, Wei TH, et al. Median nerve fascicle transfer versus ulnar nerve fascicle transfer to the biceps motor branch in C5-C6 and C5-C7 brachial plexus injuries: Nonrandomized prospective study of 23 consecutive patients. Microsurgery. 2014, 34: 511–5.

[101] Kaizawa Y, Kakinoki R, Ohta S, Noguchi T. Free functional muscle transplantation of an anomalous femoral adductor with a very large muscle belly: A case report. J Brachial Plex Peripher Nerve Inj. 2013, 8: 11.

[102] Tuohuti T, Yu Q, Yang J, Wang T. Selective neurotization of the radial nerve in the axilla using intercostal nerve to treat complete brachial plexus palsy. Int J Clin Exp Med. 2016, 9: 22880–5.

[103] Flores LP. Objective Predictors of Functional Recovery Associated with Intercostal Nerves Transfer for Triceps Reinnervation in Global Brachial Plexus Palsy. Brazilian Neurosurg 2016, 35: 271–8.

[104] Emamhadi M. Nerve transfer to relieve pain in upper brachial plexus injuries: Does it work? Clin Neurol Neurosurg. 2017,163: 67–70.

[105] Abdixbir A, Li P, Ilhamjan U, Exmetjan Y. Phrenic nerve transfer versus intercostal nerve transfer for the repair of brachial plexus root avulsion injuries. Chinese J Tissue Eng Res. 2016, 20: 7660–5.

[106] Limthongthang R, Vathana T, Wongtrakul S, Songcharoen P. End-to-Side Neurorrhaphy to Restore Elbow Flexion in Brachial Plexus Injury. J Med Assoc Thai. 2016, 99: 1203–8.

[107] De Oliveira C, Da Silva Melo D, Mestriner R, De Menezes M, Da Silva Filho I, Da Silva J. Chordata method combined with electrotherapy in functional recovery after brachial plexus injury: Report of three clinical cases. Sci Med (Porto Alegre). 2016, 26: 22425.

Article title: Developing a core outcome set for Traumatic Brachial Plexus Injuries: a systematic review of outcomes

For peer review only - http://bmjopen.bmj.com/site/about/guidelines.xhtml

Supplementary file 3. Included Studies

[108] Xu B, Dong Z, Zhang C-G, Gu Y-D. Clinical outcome following transfer of the supinator motor branch to the posterior interosseous nerve in patients with C7-T1 brachial plexus palsy. J Reconstr Microsurg. 2015, 31: 102–6.

[109] Wu X, Cong XB, Huang QS, Ai FX, Liu YT, Lu XC, et al. Transposition of branches of radial nerve innervating supinator to posterior interosseous nerve for functional reconstruction of finger and thumb extension in 4 patients with middle and lower trunk root avulsion injuries of brachial plexus. J Huazhong Univ Sci Technolog Med Sci. 2017, 37: 933–7.

[110] Kazamel M, Sorenson EJ. Electromyographic Findings in Gracilis Muscle Grafts Used to Augment Elbow Flexion in Traumatic Brachial Plexopathy. J Clin Neurophysiol. 2016. 33:549–53.

[111] Li Z, Reynolds M, Satteson E, Nazir O, Petit J, Smith BP. Double Distal Intraneural Fascicular Nerve Transfers for Lower Brachial Plexus Injuries. J Hand Surg Am. 2016, 41: e15-9.

[112] Arnal M, Cambon A, Marcheix P. Restoration of elbow and hand function in total brachial plexus palsy with intercostal nerves and C5 root neurotization. Results in 21 patients. Hand Surg Rehabil. 2016, 35: 283–7.

[113] Jiang Y, Lao J. The phrenic nerve transfer in the treatment of a septuagenarian with brachial plexus avulsion injury: a case report. Int J Neurosci. 2018, 128: 467–71.

[114] Flores LP. Outcomes of Transferring a Healthy Motor Fascicle From the Radial Nerve to a Branch for the Triceps to Recover Elbow Extension in Partial Brachial Plexus Palsy. Neurosurgery. 2017, 80: 448–53.

[115] Johnsen PH, Wolfe SW. Successful Nerve Transfers for Traumatic Brachial Plexus Palsy in a Septuagenarian: A Case Report. Hand (N Y). 2016, 11: NP30–3.

[116] Maldonado AA, Romero-Brufau S, Kircher MF, Spinner RJ, Bishop AT, Shin AY. Free Functioning Gracilis Muscle Transfer for Elbow Flexion Reconstruction after Traumatic Adult Brachial Pan-Plexus Injury: Where Is the Optimal Distal Tendon Attachment for Elbow Flexion?. Plast Reconstr Surg. 2017, 139: 128–36.

[117] Bhandari PS. Results of Distal Nerve Transfers in Restoration of Shoulder Function in C5 and C6 Root Avulsion Injury to the Brachial Plexus. Indian J Neurotrauma 2017, 14: 21–5.

[118] Watanabe M, Yamamoto T, Fukaya C, Obuchi T, Kano T, Kobayashi K, et al. Bipolar dual-lead spinal cord stimulation between two electrodes on the ventral and dorsal sides of the spinal cord: consideration of putative mechanisms. Acta Neurochir (Wien). 2018, 160: 639–43.

[119] Al-Qattan M, Kattan A, Al-Qahtany B, Al-Qattan O. Triceps nerve to deltoid nerve transfer after an unsatisfactory intra-plexus neurotization of the posterior division of the upper trunk. Int J Surg Case Rep. 2017, 37: 124–6.

[120] Alrabai H, Gesheff G, Hammouda A, Conway J. Trapezius Muscle Transfer for Restoration of Elbow Extension in a Traumatic Brachial Plexus Injury. J Hand Surg Am. 2018, 43: 872.

[121] Bertelli JA. Transfer of the radial nerve branch to the extensor carpi radialis brevis to the anterior interosseous nerve to reconstruct thumb and finger flexion. J Hand Surg Am. 2015, 40: 323-328.

[122] Magistroni E, Ciclamini D, Panero B. Ultrasound-guided pulse-dose radiofrequency: Treatment of neuropathic pain after brachial plexus lesion and arm revascularization. Case Rep Med. 2014, 201:429618.

Article title: Developing a core outcome set for Traumatic Brachial Plexus Injuries: a systematic review of outcomes

For peer review only - http://bmjopen.bmj.com/site/about/guidelines.xhtml

Supplementary file 3. Included Studies

[123] Liu Y, Xu X, Zou Y, Li S, Zhang B. Phrenic nerve transfer to the musculocutaneous nerve for the repair of brachial plexus injury: Electrophysiological characteristics. Neural Regen Res. 2015, 10: 328–33.

[124] Le Hanneur M, Walch A, Gerosa T, Grandjean A, Masmejean E. Postoperative motor deficits following elbow flexion reanimation by nerve transfer. Hand Surg Rehabil 2018, 37: 289-294

[125] Liu Y, Zhuang Y, Yu H, Xiong H. Comparative study of phrenic and partial ulnar nerve transfers for elbow flexion after upper brachial plexus avulsion: A retrospective clinical analysis. J Plast Reconstr Aesthetic Surg. 2018, 71: 1245–51.

[126] Yavari M, Mahmoudvand H, Nadri S. Contralateral medial pectoral nerve transfer with free gracilis muscle transfer in old brachial plexus palsy. J Surg Res. 2018, 231: 94–8.

[127] Yanagisawa T, Fukuma R, Seymour B, Hosomi K, Kishima H, Shimizu T, et al. MEG-BMI to control phantom limb pain. Neurol Med Chir (Tokyo). 2018, 58: 327–33.

[128] Choong C, Shalimar A. Complete brachial plexus injury - An amputation dilemma. A case report. Malaysian Orthop J. 2015, 9: 52–4.

[129] Tsao J, Finn S. Reversal of phantom pain and hand-to-face remapping after brachial plexus avulsion. Ann Clin Transl Neurol. 2016, 3: 463–4..

[130] Kubota S, Hara Y, Shimizu Y, Kadone H, Kubo T, Marushima A, et al. A newly developed upper limb single-joint HAL in a patient with elbow flexion reconstruction after traumatic brachial plexus injury: A case report. Interdiscip Neurosurg Adv Tech Case Manag. 2017, 10: 66–8.

[131] Bertelli JA. Free Reverse Gracilis Muscle Combined With Steindler Flexorplasty for Elbow Flexion Reconstruction After Failed Primary Repair of Extended Upper-Type Paralysis of the Brachial Plexus. J Hand Surg Am. 2019, 44: 112-120

[132] Xu B, Dong Z, Zhang C. Multiple nerve and tendon transfers: A new strategy for restoring hand function in a patient with C7-T1 brachial plexus avulsions. J Neurosurg. 2017, 127: 837–42.

For peer review only

Article title: Developing a core outcome set for Traumatic Brachial Plexus Injuries: a systematic review of outcomes

For peer review only - http://bmjopen.bmj.com/site/about/guidelines.xhtml

Supplementary file 4: Unique outcomes mapped to potential domains and core areas according to COMET

Online Supplementary file 4. Table: Unique outcomes mapped to potential domains and core areas according to COMET(Dodd et al., 2018)

Outcomes (n=157)	Subdomains	Domains	Core Areas
Isometric muscle strength	Muscle strength/ function	Musculoskeletal and connective tissue domain	Physiological/Clinical
Concentric strength			
Eccentric strength			
Muscle flicker/contraction			
Anti-gravity muscle activity			
Muscle endurance			
Muscle fatigue			
Muscle torque			
Active range of movement	Active movement		
Perception of movement			
Antigravity movement			
Independent movement without donor			
Passive range of movement	Passive movement		
Movement control/stability	Control of movement/stability		
Muscle mass	Muscle mass		
Bony union	Bone structure/position		
Joint position			
Joint stability			
General sensory recovery	General sensory recovery	Nervous system	
Feeling of numbness			
Proprioception			
Light touch	Discriminative touch		
2 PD			

Article title: Developing a core outcome set for Traumatic Brachial Plexus Injuries: a systematic review of outcomes

For peer review only - http://bmjopen.bmj.com/site/about/guidelines.xhtml

Supplementary file 4: Unique outcomes mapped to potential domains and core areas according to COMET

Vibration			
Object recognition			
Pain	Protective touch		
Temperature			
Deep pressure			
Brachial plexus structure	Peripheral nervous system structure		
Level of reinnervation	Reinnervation		
Time to reinnervation			
Progression of regeneration	Progression of regeneration		
Speed of motor sensory conduction	Speed of motor and sensory conduction		
Pain intensity	Pain intensity/relief	General outcomes/symptoms	
Pain relief / reduction			
Pain duration	Pain duration/frequency		
Pain frequency			
Pain quality	Pain quality and interference with life		
Pain interference with walking			
Pain interference in mood			
Pain interference with work			
Pain interference in activities of daily living			
Pain interference with relationships			
Pain interference with enjoyment of life			
Pain interference with sleep			
Sensitivity to cold	Pain when arm exposed to cold		
Paraesthesia	Paraesthesia and itchiness		
Itchiness			

Article title: Developing a core outcome set for Traumatic Brachial Plexus Injuries: a systematic review of outcomes

For peer review only - http://bmjopen.bmj.com/site/about/guidelines.xhtml

For peer review only

Supplementary file 4: Unique outcomes mapped to potential domains and core areas according to COMET

Sensitivity to pressure	Sensitivity to touch, pressure etc		
Sensitivity to touch			
Pain location	Location of pain		
Pain relief from medication	Pain medication use		
Stiffness	Stiffness		
Impact on general sleep	Impact on sleep		
Impact on sleep on affected side			
Frequency sleep disturbed by injury			
General physical function	Physical function non-specific	Physical functioning	Life Impact
Patient led functional outcome			
Walking short distance	Lower limb and non -upper limb function		
Balance			
Running			
Climbing stairs			
Bending			
Kneeling			
Reaching	Reaching, pulling, pushing, carrying etc		
Pulling			
Pushing			
Carrying			
Throwing			
Lifting			
General function of arm			
Turning and twisting arm	Turning twisting, gripping and releasing with the arm		
Grip and release			
Pinching	Fine hand movement including writing		
Fine hand movement (writing/buttons)			
Returning to work	Impact on paid or unpaid work or role in education	Role functioning	
Ability to do work			
Usual time at work			
Type of work			

Article title: Developing a core outcome set for Traumatic Brachial Plexus Injuries: a systematic review of outcomes

For peer review only - http://bmjopen.bmj.com/site/about/guidelines.xhtml

For peer review only

Supplementary file 4: Unique outcomes mapped to potential domains and core areas according to COMET

Usual school activities			
General rating to perform a patient specific activity	Role function - patient specific		
Impact on ADL (general)	Carrying out daily routine, (including food preparation, housework, garden, plants)		
Return to ADL (general)			
Impact on food preparation and feeding			
Housework (washing, cleaning, ironing, folding, vacuuming)			
Gardening (Includes indoor plants)			
Using a phone			
Maintaining personal hygiene			
Maintaining personal appearance (grooming hair)	Maintaining personal hygiene		
Dressing	Maintaining personal appearance		
Transport needs (e.g driving)	Dressing		
Impact on normal hobbies	Transport needs		
Time doing normal hobbies	Impact on recreational activities and sport		
Playing instrument in usual way			
Ability to play instrument			
Impact on time spent playing instrument			
Impact on time spent doing sport			
Impact on participation in sport			
Social activities with friends	Effect on relationship with	Social functioning	

For peer review only - http://bmjopen.bmj.com/site/about/guidelines.xhtml

For peer review only

Supplementary file 4: Unique outcomes mapped to potential domains and core areas according to COMET

Social activities with neighbours	family, friends, neighbours and groups		
Social activities with family			
Social activities with groups			
Dependence on family and friends			
Appearance interferes with social activities			
Intimate relationships	Effect on intimate relationships		
Emotional impact on work	Emotional distress/mood	Emotional functioning	
Energy levels			
Emotional impact on ADL			
Happiness			
Impact on life enjoyment / satisfaction			
Emotional impact on relationships			
Anxiety			
Depression			
Acceptance/ Adjustment	Thoughts and beliefs (acceptance, coping)		
Coping with trauma			
Confidence	Self esteem and confidence		
Self esteem			
Body image	Body image		
Quality of life	Quality of Life	Global Quality of Life	Quality of Life
Rating of health	Perceived Health status	Health status	Health status
General patient satisfaction	Patient satisfaction	Delivery of Care	Delivery of Care
Satisfaction with appearance of arm			
Satisfaction with function			
Satisfaction with movement			
Satisfaction with strength			

For peer review only

For peer review only - http://bmjopen.bmj.com/site/about/guidelines.xhtml

Supplementary file 4: Unique outcomes mapped to potential domains and core areas according to COMET

Satisfaction with pain			
Satisfaction with colour			
Satisfaction with shape			
Satisfaction with feeling			
Satisfaction with procedure			
Patient preference	Patient preference		
Quality of intervention	Accessibility, quality and adequacy of intervention		
Time to surgery	Time to surgery		
Operation time	Operation time	Resource Use	Resource Use
Motor morbidity	Donor site morbidity	Adverse Events	Adverse Events
Sensory morbidity			
Pain			
General complications	General complications		
Pneumothorax	Respiratory complications		
Respiratory function			
Respiratory symptoms			
Pneumonia			
Arterial thrombosis	Vascular complications		
Venous thrombosis			
Haematoma			
Venous spasm			
Iatrogenic vascular injury			
Vascularity of flap			
Swelling			
Fracture	Musculoskeletal complications		
Passive range of motion loss			
Co-contraction			
Bowstringing			
Failure of tendon attachment			
Joint Instability			
Scapula crepitus			

Article title: Developing a core outcome set for Traumatic Brachial Plexus Injuries: a systematic review of outcomes

For peer review only

For peer review only - http://bmjopen.bmj.com/site/about/guidelines.xhtml

Supplementary file 4: Unique outcomes mapped to potential domains and core areas according to COMET

Infection complications	Infection complications		

Dodd, S. et al. A taxonomy has been developed for outcomes in medical research to help improve knowledge discovery. Journal of clinical epidemiology. 2018, 96: 84–92.

For peer review only

Supplementary file 5. Measurement of outcomes and measurement tools used

Supplementary file 5. Measurement of outcomes and measurement tools used

56 outcome subdomains in 4 core areas (Physiological/clinical, Life Impact, Resource Use and Adverse events) and within the following COMET domains

Musculoskeletal/connective tissue, Nervous system outcome domain, General outcome and symptom domain, Physical functioning, Role functioning, Emotional functioning, Global quality of life, Perceived health status, Delivery of care, Hospital resources and Adverse Events

**Core Area**	Outcome subdomains	**Measurement type used (N)**				**Measurement instruments used (number of studies)**
**PHYSIOLOGICAL /CLINICAL**		**Patient reported Outcome**	**Clinician reported Outcome**	**Perfomance Outcome**	**Not Clear**	
	**Musculoskeletal/connective tissue**					
	Muscle strength	30	129	19	3	DASH (n= 27), UEFI (n=2), MHQ (n=1),<br><br>**Manual Muscle Testing**<br>**Manual muscle testing undefined (n=5)**<br>**MRC muscle grading (n=61 , including UCLA)**<br>**MRC muscle grading modified (n= 22),**<br>*MRC modified*, unclear how (n= 5)<br>*MRC modified,* grade 3 active must equal passive (n=2)<br>*MRC modified* , grade 2 active must equal passive movement (n=2)<br>*MRC modified,* M3+ contraction with resistance against a finger for less than 30 seconds, M4 contraction of resistance against a finger against a finger for more than 30 seconds (n=1)<br>*MRC modified*: M0, M1+, M1, M1+, M2-, M2, M2+,M3-, M3, M3+, M4-, M4, M4+, M5-, M5 (n=6)<br>*MRC modified,* Finger flexion tested with wrist extended 20-30 degrees (n=1)

For peer review only

Supplementary file 5. Measurement of outcomes and measurement tools used

						MRC modified, Addition of M4.5 (n=1)<br>*MRC modified,* graded two muscles together (n=1)<br>*MRC modified,* finger extension tested with wrist extension at 20-30 degrees (n=1)<br>*MRC modified,* summated muscle score (n=1)<br>*MRC modified,* FDS tested by stabilising LF and IF to table and testing MF and RF IP flexion (n=1)<br>**Other manual muscle tests (n=3)**<br>Kendall and McCreary testing procedure (n=1)<br>Oxford muscle testing (n=1)<br>Modification of the Louisiana State University Medical Centre grading system (n=1)<br>**Time to (n= 12)**<br>contraction (n=7); M2 (n=1); strength greater than or equal to M3 (n=1); M 3 (n=1); greater than or equal to modified M3 (n=1); Time to improvement in MRC scale (n= 1)<br>**Dynanometry (n=23)**<br>Dynanometry – isokinetic machine, undefined method (n =1)<br>Grip strength JAMAR , undefined method (n=4); Hook grip – isokinetic machine, undefined method (n=1); Grip strength JAMAR, mean of 3 trials n=2); Grip strength , PABLO system, undefined (n=1); Pinch grip, JAMAR, undefined (n= 3), Pinch grip JAMAR, mean 3 trials (n= 1); Peak isometric, hand held dynamometer (n=2); Isometric strength , hand held dynamometer, best of 3 trials (n=1); Isometric strength , Kendall & Kendall positions, 3 trials mean value (n=1); Measurement on digital scales after 5 seconds (n=1)<br>Concentric strength through range, Isokinetics (n=1)<br>Eccentric strength through range, isokinetics (n=1)<br>Combined action of using elbow and hand on digital hanging scale (n=1)<br>Constant-Murley score: dynanometry 90 degrees abduction(n=2)

For peer review only

Supplementary file 5. Measurement of outcomes and measurement tools used

						**Narakas score modified (one study)**<br>Thoaraco brachial grasp (n=1)<br>Elbow flexion with weight (n=1)<br>Elbow extension with weight (n=1)<br>Wrist flexion with weight (n=1)<br>Wrist extension with weight(n=1)<br>Fist power with weight (n=1)<br>Pinch power (n=1)<br><br>**ULM (one study)**<br>Shoulder flexion to shoulder height with 500g (n=1)<br>Shoulder flexion above shoulder height with 500g (n=1)<br>Shoulder flexion above shoulder with 1kg (n=1)<br>Move weight on table (100g) (n=1)<br>Move weight on table (500g) (n=1)<br>Move weight on table (1KG) (n=1)<br><br>**SHAP (one study)**<br>Grip strength (n=1)<br>Pinch strength (n=1)<br>Pinch grip (lateral) (n=1)<br>Pinch grip (tip) (n=1)<br>Grip strength (power) (n=1)<br>Heavy extension (n=1)<br><br>Ability to lift weight, undefined (n=1)<br>Number of repetitions movement can be performed in 10 seconds (n=1)<br>Maximum weight sustained when flexing elbow (n=1)<br>**Unclear (n= 3)**

For peer review only

Supplementary file 5. Measurement of outcomes and measurement tools used

						Force recovery: Cross sectional area of the muscle under isometric contraction divided by cross sectional area at rest (n=1)
	Active movement	5	103	3	63	SST(n=1), MHQ (n=1), UCLA shoulder rating scale (n=1), MPI(n=2), CONSTANT- MURLEY(n=2) (2xPRO, 8x ClinRO), ARAT (PerfO, n=1), ULM (PerfO, n=2), Goniometry(n=48), Visual assessment (n=32), First web space in cm (n=3), Total active movement(n=2), Pulp to palm distance (n=2)<br>Months to full active movement (n=1)<br>Months to antigravity movement (n=3)<br>Months to initial movement (n= 1)<br>Months to independent movement without donor (n=1)<br>Not clear (n=63)
	Passive range of movement		6		7	Not defined (n=7), Goniometry(n=6)
	Movement control and stability		1	1	2	MPI (ClinRo, n=1), ULM (PerfO, n=1), Not clear (n=2)
	Bone structure/position/healing				4	Not clear (n=4)
	Muscle mass				4	Not clear(n=4)
	**Nervous system outcome subdomains**					
	General sensory recovery including proprioception		**9**		**8**	Sensory BMRC (n=5), Modified Sensory BMRC (n= 2), Highet classification(n=2), Not clear (n=8)
	Discriminative touch (light touch, two point discrimination, vibration, object recognition)	**1**	**14**			MHQ (n=1), Cotton wool (n=3), Semmes Weinstein Monofilaments (n=4), Two point discrimination(n=2), Tuning fork (n=4), Not defined (ClinRo, n=1)
	Protective touch (pain, temperature, deep pressure)		**3**		**7**	Blunt pin (n=3), Not clear (n=7)
	Structure of peripheral nervous system		1			MRI (n=1)
	Reinnervation (level of reinnervation, time to innervation)		54			Two point scale on EMG(n=1) Four point scale on EMG (n=4), Not clear EMG (n= 49)
	Progression of regeneration		5			Tinel sign (n=5)
	Speed of motor and sensory conduction		9			EMG (n=9)
	**General outcomes / symptoms**					

For peer review only

Supplementary file 5. Measurement of outcomes and measurement tools used

	Pain intensity/ relief	73			3	DASH (n=27), ASES (n=1), TAPES (n=1), VAS(n=18), NRS(n=12),MHQ (n=1) WBFRS(n=1), BPI (n= 1), UNWNS (n=1), McGill Pain Questionnaire SF (n=2), McGill pain questionnaire (n= 1), MPI (n=1), CONSTANT-MURLEY (n=2), 4 point scale (n=3) Author developed questionnaire(n=1), Not Clear (n=3)
	Pain duration or frequency	12	0	0	0	SST (n=1), SF36 (n=5), MHQ (n=1),TAPES(n=1), NPSI (n=1), BPI (n= 1), UCLA shoulder rating score (n=1), Not described PRO (n=1)
	Pain quality	7				TAPES (n= 1), NPSI(n=1), UWNS(n= 1), McGill SF(n=2), McGill (n=1), Non described PRO (n =1)
	Pain when arm exposed to cold	1				NPSI (n=1)
	Paraesthesia	27				DASH (n=27)
	Sensitivity to touch, pressure, vibration etc	3				NPSI (n=1) UWNS (n= 1), NRS (n=1)
	Location of pain	1				BPI (n=1)
	Pain medication use	1				BPI(n=1)
	Stiffness	27				DASH (n=27)
	**Physical functioning**					
	Physical function non-specific	2				PSFS (n=1), TAPES (n=1)
	Lower limb and non-upper limb function (walking, running, climbing stairs etc)	7			1	SF36 (n=5), TAPES (n= 1), BPI (n=1) Non described PRO (n=1)
	Reaching, pulling, pushing, carrying, throwing , lifting	37		3		DASH (n=27), UEFI (n=2), MHQ(n=1), ASES(n=1), SST (n=1), SF36(n=5), ARAT(n=1), AMULA (n=1) UNBtP (n=1)
**LIFE IMPACT**	Turning twisting, gripping and releasing with the arm	30		5	1	DASH (n=27), UEFI (n=2), MHQ (n=1),ARAT(n=1),SHAP(n= 1), JHFT (n=1), AMULA (n=1), UNBtP (n=1), Not clear (n=1)
	Fine hand movement include writing	30		6		DASH (n=27), UEFI (n=2), MHQ (n=1),ARAT(n=1), SHAP(n=1), JHFT (n=1) Purdue Peg test (n=1),AMULA (n=1), UNBtP (n=1)
	**Role Functioning**					
	Impact on return to work	41				DASH (n =27), UEFI (n=2),MHQ (n=1), ASES (n=1), SST (n=1), SF36 (n=5), TAPES (n=1), MPI (n=1) No description PRO (n=1), Questionnaire no data (n=1)
	Role function patient specific	1				PSFS(n=1)

For peer review only

Supplementary file 5. Measurement of outcomes and measurement tools used

	Carrying out daily routine, (including food preparation, housework, garden, plants)	36	1	5		DASH (n=27), UEFI (n=2), MHQ (n=1),TAPES(n=1) , BPI (n=1), UCLA (n=1), SHAP (n=1), Jebsen (n=1), ULM (n=1) Questionnaire not defined (n=2),No description PRO (n=1) Unclear CLinRO(n=1), AMULA (n=1), UNBtP (n=1)
	Maintaining personal hygiene	35		2		DASH (n=27), ASES (n=1), SST(n=1), SF36(n=5), MHQ(n=1) AMULA (n=1), UNBtP (n=1)
	Maintaining personal appearance	3		1		UEFI (n=2), ASES (n= 1), AMULA (n=1)
	Dressing	32		2		DASH (n =27), UEFI (n=2), MHQ (n=1),ASES (n= 1), SST (n=1), AMULA (n=1) SHAP(n=1)
	Transport needs	29				DASH (n =27), UEFI (n=2),
	Impact on recreational activities and sport	34				DASH (n =27), UEFI (n=2), ASES (n= 1), TAPES(n=1), CONSTANT-MURLEY (n=2),Not described PRO (n=1)
	**Social functioning**					
	Effect on relationship with family, friends, neighbours and groups	34				DASH (n =27), SF36 (n=5), TAPES (n=1), MHQ (n=1)
	Effect on intimate relationships	27				DASH (n =27)
	**Emotional Functioning**					
	Emotional distress/ mood	11				SF36 (n=5), TAPES (n= 1),BPI(n=1), UWNS(n=1), Self-rated anxiety scale (n=1), Self-rated depression scale (n=1), MHQ (n=1)
	Thoughts and beliefs (acceptance and adjustment)	1				TAPES (n=1)
	Self-esteem and self confidence	28				DASH (n=27), TAPES(n= 1)
	Body image	3				MHQ (n= 2), Not described (n=1)
	**Sleep and overall health**					
	Impact on sleep	37				DASH (n=27), UEFI (n=3), ASES(n= 1), MHQ (n=1), SST (n=1), BPI(n=1), CONSTANT- MURLEY(n=2),Not described PRO (n=1)
	**General Quality of life**	1				Not described PRO (n=1)
	**Perceived Health Status**	6				SF36 (n=5), TAPES (n=1)
	**Delivery of Care**					
	Patient satisfaction	10				TAPES (n=1), UCLA (n=1), MHQ (n=1),10-point scale (n=1)

For peer review only

Supplementary file 5. Measurement of outcomes and measurement tools used

						4 point scale (n=2), 3 point likert scale (n=1), Questionnaire not described (n=1),Not defined PRO(n=2)
	Patient preference for treatment	1				Not described (n=1)
	Accessibility, quality and adequacy of intervention				1	4 point scale (n=1)
RESOURCE	**Hospital**					
	Operation time				1	Not described (n=1)
	**Adverse Events**					
ADVERSE	Donor site motor morbidity to include weakness		18		19	BMRC (n=7), BMRC modified(n=2), Dynanometry (n=8), EMG(n=1)<br>Not clear (n=19)
	Donor site sensory morbidity	1	3		4	10-point scale PRO (n=1)<br>Not defined (n=4),2PD (n=2), Monofilaments (n=1)
	Donor site morbidity -pain	3				Not defined PRO (n=3)

For peer review only

Supplementary file 5. Measurement of outcomes and measurement tools used

	General complications				2	Unclear (n=2)
	Respiratory complications	1	5		4	4 point scale PRO (n=1), x-ray (n=2), FEV (n=1), TLC(n=1), MVV (n-1), Not defined (n=4),
	Vascular complications		2		13	Not defined (n=13), Visual assessment (n=1), USS (n=1)
	Musculoskeletal complications		2		19	Not defined CLinRO(n=2), Unclear (n=19)
	Infection complications		1		2	Not defined ClinRo(n=1), Unclear (n=2)
		669	366	46	168	

DASH Disabilities of the arm shoulder and hand, *UEFI* Upper Extremity Functional Index, *MHQ* Michigan Hand Questionnaire, *BMRC* British Medical Research Council, *ULM* Upper Limb Module, *SHAP* Southampton Hand Assessment Procedure, *SST* Simple Shoulder Test, *MPI* Mayo clinic Performance Index for the elbow, *ARAT* Action Research Arm Test, *ClinRO* Clinician Reported Outcome, *PerfO* Performance Outcome, *PRO* Patient Reported Outcome, *ASES* American Shoulder and Elbow Surgeons Index, *TAPES* The Trinity Amputation and Prosthesis Experience Scales, *VAS* Visual Analogue Scale, *NRS* Numerical Rating Scale, *WBFRS* Wong Baker Faces Rating Scale, *UNWNS* University of Washington Neuropathic pain Score, *SF36* Short Form 36 health survey, *NPSI* Neuropathic Pain Symptom Inventory, *BPI* Brief Pain Inventory, *PSFS* Pain Specific Functional Scale, *AMULA* American Measures for Upper Limb Amputees, *UNBPT* University of New Brunswick test of Prosthetics function, *JHFT* Jebsen Hand Function Test, *FEV* Forced Expiratory Volume, *TLC* Tidal Lung Capacity, *MVV* maximal voluntary ventilation, *USS* Ultrasound Scan.

For peer review only

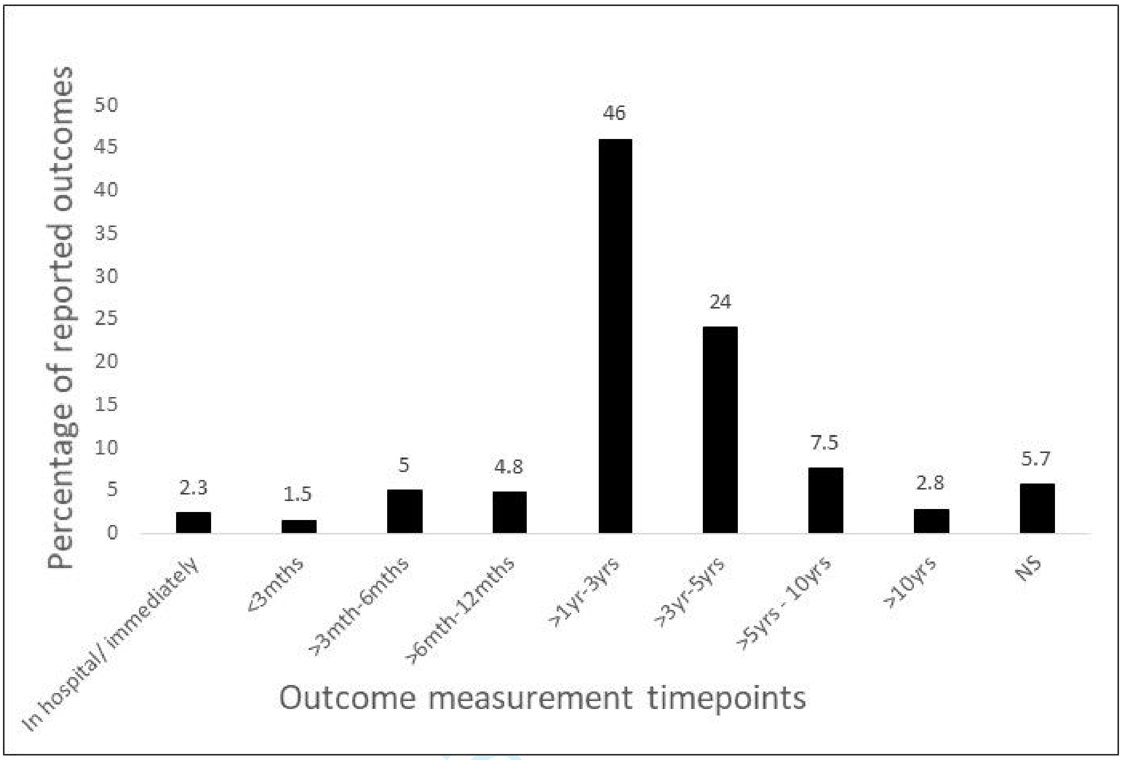
Percentage of reported outcomes
50
45
40
35
30
25
20
15
10
5
0
2.3
1.5
5
4.8
46
24
7.5
2.8
5.7
In hospital/ immediately
<3mths
>3mth-6mths
>6mth-12mths
>1yr-3yrs
>3yr-5yrs
>5yrs - 10yrs
>10yrs
NS
Outcome measurement timepoints

PRISMA 2009 Checklist

Section/topic	#	Checklist item	Reported on page #
**TITLE**			
Title	1	Identify the report as a systematic review, meta-analysis, or both.	1
**ABSTRACT**			
Structured summary	2	Provide a structured summary including, as applicable: background; objectives; data sources; study eligibility criteria, participants, and interventions; study appraisal and synthesis methods; results; limitations; conclusions and implications of key findings; systematic review registration number.	2
**INTRODUCTION**			
Rationale	3	Describe the rationale for the review in the context of what is already known.	3
Objectives	4	Provide an explicit statement of questions being addressed with reference to participants, interventions, comparisons, outcomes, and study design (PICOS).	4
**METHODS**			
Protocol and registration	5	Indicate if a review protocol exists, if and where it can be accessed (e.g., Web address), and, if available, provide registration information including registration number.	5
Eligibility criteria	6	Specify study characteristics (e.g., PICOS, length of follow-up) and report characteristics (e.g., years considered, language, publication status) used as criteria for eligibility, giving rationale.	7
Information sources	7	Describe all information sources (e.g., databases with dates of coverage, contact with study authors to identify additional studies) in the search and date last searched.	5
Search	8	Present full electronic search strategy for at least one database, including any limits used, such that it could be repeated.	S1
Study selection	9	State the process for selecting studies (i.e., screening, eligibility, included in systematic review, and, if applicable, included in the meta-analysis).	6
Data collection process	10	Describe method of data extraction from reports (e.g., piloted forms, independently, in duplicate) and any processes for obtaining and confirming data from investigators.	8
Data items	11	List and define all variables for which data were sought (e.g., PICOS, funding sources) and any assumptions and simplifications made.	8
Risk of bias in individual studies	12	Describe methods used for assessing risk of bias of individual studies (including specification of whether this was done at the study or outcome level), and how this information is to be used in any data synthesis.	8
Summary measures	13	State the principal summary measures (e.g., risk ratio, difference in means).	n/a
Synthesis of results	14	Describe the methods of handling data and combining results of studies, if done, including measures of consistency (e.g., I^2) for each meta-analysis.	n/a

For peer review only - http://bmjopen.bmj.com/site/about/guidelines.xhtml

PRISMA 2009 Checklist

Section/topic	#	Checklist item	Reported on page #
Risk of bias across studies	15	Specify any assessment of risk of bias that may affect the cumulative evidence (e.g., publication bias, selective reporting within studies).	n/a
Additional analyses	16	Describe methods of additional analyses (e.g., sensitivity or subgroup analyses, meta-regression), if done, indicating which were pre-specified.	n/a
**RESULTS**			
Study selection	17	Give numbers of studies screened, assessed for eligibility, and included in the review, with reasons for exclusions at each stage, ideally with a flow diagram.	7
Study characteristics	18	For each study, present characteristics for which data were extracted (e.g., study size, PICOS, follow-up period) and provide the citations.	Table S2
Risk of bias within studies	19	Present data on risk of bias of each study and, if available, any outcome level assessment (see item 12).	n/a
Results of individual studies	20	For all outcomes considered (benefits or harms), present, for each study: (a) simple summary data for each intervention group (b) effect estimates and confidence intervals, ideally with a forest plot.	n/a
Synthesis of results	21	Present results of each meta-analysis done, including confidence intervals and measures of consistency.	n/a
Risk of bias across studies	22	Present results of any assessment of risk of bias across studies (see Item 15).	n/a
Additional analysis	23	Give results of additional analyses, if done (e.g., sensitivity or subgroup analyses, meta-regression [see Item 16]).	n/a
**DISCUSSION**			
Summary of evidence	24	Summarize the main findings including the strength of evidence for each main outcome; consider their relevance to key groups (e.g., healthcare providers, users, and policy makers).	25-26
Limitations	25	Discuss limitations at study and outcome level (e.g., risk of bias), and at review-level (e.g., incomplete retrieval of identified research, reporting bias).	27
Conclusions	26	Provide a general interpretation of the results in the context of other evidence, and implications for future research.	30
**FUNDING**			
Funding	27	Describe sources of funding for the systematic review and other support (e.g., supply of data); role of funders for the systematic review.	Title page

From: Moher D, Liberati A, Tetzlaff J, Altman DG, The PRISMA Group (2009). Preferred Reporting Items for Systematic Reviews and Meta-Analyses: The PRISMA Statement. PLoS Med 6(7): e1000097. doi:10.1371/journal.pmed1000097

For more information, visit: **www.prisma-statement.org**.

For peer review only - http://bmjopen.bmj.com/site/about/guidelines.xhtml

BMJ Open

Developing a core outcome set for traumatic brachial plexus injuries: a systematic review of outcomes.

Journal:	*BMJ Open*
Manuscript ID	bmjopen-2020-044797.R1
Article Type:	Original research
Date Submitted by the Author:	07-Dec-2020
Complete List of Authors:	Miller, Caroline; University of East Anglia, School of Health Sciences, The Queens Building ; Queen Elizabeth Hospital Birmingham, Physiotherapy Department<br>cross, jane; University of East anglia, school AHP<br>O'Sullivan, Joel; Queen Elizabeth Hospital Birmingham, Physiotherapy<br>Power, Dominic; Queen Elizabeth Hospital Birmingham, The Birmingham Peripheral Nerve Injury Service<br>Kyte, Derek; University of Birmingham, Institute of Applied Health Research<br>Jerosch-Herold, Christina; University of East Anglia, Health Sciences
**Primary Subject Heading**:	Neurology
Secondary Subject Heading:	Emergency medicine, Rehabilitation medicine, Surgery
Keywords:	Neurological injury < NEUROLOGY, Limb reconstruction < ORTHOPAEDIC & TRAUMA SURGERY, Plastic & reconstructive surgery < SURGERY, Neurological pain < NEUROLOGY, Elbow & shoulder < ORTHOPAEDIC & TRAUMA SURGERY, Trauma management < ORTHOPAEDIC & TRAUMA SURGERY

SCHOLARONE™
Manuscripts

I, the Submitting Author has the right to grant and does grant on behalf of all authors of the Work (as defined in the below author licence), an exclusive licence and/or a non-exclusive licence for contributions from authors who are: i) UK Crown employees; ii) where BMJ has agreed a CC-BY licence shall apply, and/or iii) in accordance with the terms applicable for US Federal Government officers or employees acting as part of their official duties; on a worldwide, perpetual, irrevocable, royalty-free basis to BMJ Publishing Group Ltd ("BMJ") its licensees and where the relevant Journal is co-owned by BMJ to the co-owners of the Journal, to publish the Work in this journal and any other BMJ products and to exploit all rights, as set out in our licence.

The Submitting Author accepts and understands that any supply made under these terms is made by BMJ to the Submitting Author unless you are acting as an employee on behalf of your employer or a postgraduate student of an affiliated institution which is paying any applicable article publishing charge ("APC") for Open Access articles. Where the Submitting Author wishes to make the Work available on an Open Access basis (and intends to pay the relevant APC), the terms of reuse of such Open Access shall be governed by a Creative Commons licence – details of these licences and which Creative Commons licence will apply to this Work are set out in our licence referred to above.

Other than as permitted in any relevant BMJ Author's Self Archiving Policies, I confirm this Work has not been accepted for publication elsewhere, is not being considered for publication elsewhere and does not duplicate material already published. I confirm all authors consent to publication of this Work and authorise the granting of this licence.

For peer review only

Title Page

Title of article

Developing a core outcome set for traumatic brachial plexus injuries: a systematic review of outcomes.

For peer review only

Corresponding author

Caroline Miller (corresponding author)

School of Health Sciences,

University of East Anglia,

Norwich,

UK,

NR4 7TJ

Tel no: 00447743501508

Email: caroline.miller@uea.ac.uk

Twitter handle: @c_ahern26

Co-authors

Jane Cross

School of Health Sciences,

University of East Anglia,

Norwich,

UK,

Twitter handle: @JaneCross12

Joel O Sullivan,
Physiotherapy Department,
Queen Elizabeth Hospital Birmingham,
Birmingham
UK,
Twitter handle: @jos2305

Dominic M Power,
The Birmingham Peripheral Nerve Injury Service,
Queen Elizabeth Hospital Birmingham,
Birmingham,
UK,
@dominicpower1

Derek G Kyte,
Centre for Patient-Reported Outcomes Research,
University of Birmingham,
Birmingham,
UK

Christina Jerosch-Herold,
School of Health Sciences,
University of East Anglia,
Norwich,
UK,
Twitter handle: @tinajerosch

Word count, 3,300

For peer review only

ABSTRACT

Background Clinical decisions on treatment for traumatic brachial plexus injuries (TBPI) should be based in best evidence from systematic reviews. However a lack of consistency in outcome reporting has hampered combining study findings. As a first step to developing a Core Outcome Set for TBPI, a systematic review is needed to identify what outcomes have been outcome assessed in traumatic brachial plexus injury research.

Method Medline (OVID), EMBASE, CINAHL, and AMED were systematically searched for studies evaluating the clinical effectiveness of interventions in adult traumatic brachial plexus injuries from January 2013 to September 2018. Two authors independently screened papers. All outcomes were extracted verbatim from studies. If a patient reported or performance outcome measure was used then outcomes were extracted directly from the instrument. Variation in outcome reporting was determined by assessing the number of unique outcomes reported across all included studies. Outcomes were categorized into domains using a prespecified taxonomy.

Results Verbatim outcomes (n= 1460) were extracted from 132 studies including 30 questionnaires. Unique outcomes (n= 157) were structured into four core areas and 11 domains. Outcomes within the musculoskeletal domain were measured in 87% of studies, physical functioning in 23%, emotional functioning in 22% and adverse events in 33%. One study measured quality of life. We identified 62 different methods for measuring muscle strength, 16 for range of movement and 63 studies did not define how they measured movement.

For peer review only - http://bmjopen.bmj.com/site/about/guidelines.xhtml

Conclusion This review of outcome reporting in traumatic brachial plexus injury research demonstrated an impairment focus and heterogeneity. A core outcome set would ensure standardized and relevant outcomes are reported to facilitate future systematic review and meta-analysis.

Prospero registration number: CRD42018109843

Strengths and limitations of this study

- This study is a comprehensive and systematic review of all reported clinical outcomes reported in traumatic brachial plexus studies from 2013- 2018 inclusive.
- Unique outcomes were systematically categorized into a clear taxonomy to inform the development of a core outcome set.
- Definition of unique outcomes and categorisation was conducted by researchers and clinicians to account for multidisciplinary perspectives.
- Quality assessment was not undertaken as the aim of the study was to review outcome reporting and not to synthesize data about effectiveness of interventions.

For peer review only

INTRODUCTION

A traumatic brachial plexus injury (TBPI) is a major injury to the brachial plexus. It can result in significant functional, social, psychological and economic effects, [1,2] with most occurring in young men as a result of motorbike accidents,[3]. Survival from major trauma is increasing,[4] and with this an increase in the incidence of TBPI,[5] which accounts for 1.2% of polytrauma,[6].The complex and chronic nature of the injury is associated with significant healthcare costs,[7] in addition to indirect costs estimated at up to $2.34 million (in 2017 dollars) over the lifetime of an manual labourer in the USA with a TBPI,[8]. There are multiple strategies for managing a patient with a TBPI with recent advancements in nerve microsurgery,[9] and robotics,[10] resulting in increased treatment options. The choice of treatment should be made using up-to-date, high quality scientific evidence,[11,12].

Ideally, a meta-analysis would identify the most effective treatment for an individual with a TBPI, however, such analysis requires homogenous outcome measurement and reporting across studies to enable optimum synthesis. Indeed, despite increasing numbers of TBPI studies, outcome heterogeneity and poorly defined outcomes has been highlighted as a significant challenge to evidence synthesis in two recent systematic reviews,[13,14]. There is now international agreement that the definition of a core outcome set (COS) for TBPI is a priority,[15,16]. A COS is a minimum agreed set of outcomes to be reported and measured in all studies,[17,18]. Development of a COS has been shown to reduce heterogeneity of outcome reporting in other health conditions, with 81% of trialists in rheumatoid arthritis (RA) now measuring the COS for RA,[19].

For peer review only

To date a minimum set of outcomes, important to patients and professionals for reporting in TBPI studies, has not been agreed. The choice of what are important outcomes to measure in TBPI is complex due to patient heterogeneity with different mechanisms, locations and severity of injury. COS methodology is continuously being refined and promoted by the Core Outcome Measures in Effectiveness Trials (COMET) initiative [20]. Development of a COS usually begins with identification of a long list of outcomes which is then prioritised through a consensus process. This systematic review sits within the larger global COMBINE project to identify a COS for TBPI. A Delphi study and consensus meeting, informed by data from this systematic review and interviews with people with the injury, will prioritise the final COS for TBPI.

As a first step in the development of an international COS for TBPI we conducted a systematic review to identify outcomes reported and measurement instruments used and their timing in the literature. The final step of the global project will match the COS to existing validated measurement instruments and make recommendations on when they should be collected, therefore it was necessary to identify currently used instruments and their timepoints also.

For peer review only - http://bmjopen.bmj.com/site/about/guidelines.xhtml

The aim of this review was to:

1. Identify what outcome domains are assessed in studies evaluating surgical and non-surgical treatment for TBPI.
2. Compare the definitions of outcomes and time points of outcomes assessed.
3. Identify how the outcomes were measured, that is what validated or non-validated instruments are used.

METHODS

We followed the methods described in the Cochrane Handbook for Systematic Reviews of Interventions,[21] and report in accordance with the Preferred Reporting Items for Systematic Reviews and Meta-Analysis (PRISMA) guidelines,[22]. The systematic review protocol was prospectively registered with PROSPERO (PROSPERO registration number: CRD42018109843). Deviations from the protocol are reported in supplementary file 1.

Identification of studies

We conducted an electronic search of Medline (OVID), EMBASE (OVID), CINAHL and AMED on the 18th September 2018. Studies published between 01 Jan 2013 and 18 September 2018 were included to reflect outcomes employed in current TBPI care. An example of the search strategy for Ovid MEDLINE is presented in supplementary file 2. The thesaurus vocabulary of each database was used to adapt search terms. Boolean operators (AND, OR) were used to narrow or widen the search and no language restrictions were applied.

For peer review only - http://bmjopen.bmj.com/site/about/guidelines.xhtml

Study eligibility

Studies were included if they met the following criteria:

Study type: Any controlled and uncontrolled experimental and observational studies evaluating interventions in traumatic brachial plexus injury including case reports, case series, case studies, prospective and retrospective cohort studies, randomized and non-randomized clinical trials. We excluded conference proceedings, abstract only publications and those not involving human subjects.

Participants: Studies reporting outcomes in individuals with traumatic brachial plexus injury aged 16 years or over. Studies of patients with obstetric brachial plexus injuries were excluded.

Interventions: Any surgical or non-surgical intervention for TBPI.

Outcomes: All outcomes reported in the published abstract, methods or results. These included physiological and functional outcomes, adverse events and patient reported outcomes (PROs) either reported in the study or subsequently extrapolated from the PRO instruments.

Language: Non-English language publications were included

Study selection process

The reference management software Mendeley was used to compile the literature, with duplicates removed. Authors (X and X) independently screened the titles and then the abstracts against the eligibility criteria. Disagreements were discussed and a third reviewer (x) was involved where required. Studies appearing to meet the inclusion criteria based on title and abstract were retrieved as full text articles, and were read to assess for eligibility with decisions on inclusion and exclusion recorded (Figure 1. PRISMA flow diagram).

For peer review only - http://bmjopen.bmj.com/site/about/guidelines.xhtml

Disagreements in study selection were resolved by discussion within the research team (x, x, x).

Quality assessment

Quality assessment of studies was not relevant as the objective was to systematically document all outcomes reported in TBPI studies rather than synthesize the data about intervention effectiveness.

Data Extraction

Data were extracted into a piloted data extraction sheet (Microsoft Excel). General data extracted from each study included author, study design, recruiting country, publication year, number of participants, gender, mean age, level of TBPI and intervention tested. The following information was extracted regarding outcomes: each outcome reported (verbatim), area of body assessed if relevant (shoulder, elbow, wrist or hand), method of administration, name of measure, timepoints of measure and reported complications. The number of outcomes per study was also documented.

Data extraction was performed independently by X and X for the first 20% of included studies. These were compared, and disagreements discussed and resolved through debate or discussion with a third reviewer (X). Following this a further ten percent of studies had

For peer review only

data extracted by both X and X. Due to the high level of agreement between reviewers (91% agreement) on outcomes extracted, at this stage, the remaining studies underwent extraction by a single reviewer (X).

Where a validated PRO or performance outcome measurement was used and composed of multiple items, the following data was extracted by the first author: verbatim name of the instrument, verbatim wording for each individual item. A performance outcome measurement was defined as "A measurement based on a standardized task performed by a patient that is administered and evaluated by an appropriately trained individual or is independently completed" [23]. The frequency of use of instruments was noted and compared between studies. The instruments were categorized as: (i) General Health (generic - for use with any patient); (ii) Upper limb physical function (region-specific); (iii) Symptom or domain specific (to assess a single symptom e.g. pain) and (iv) Condition Specific. Timepoints of measurement of all outcomes were noted. If the outcome was assessed at different timepoints then all timings were recorded.

Classification of outcomes into domains and defining unique outcomes

Identically worded and spelled verbatim outcomes were removed at this stage. Identical outcomes measured over different time points were noted as one outcome. Where outcomes were assessed using an instrument containing several items, each individual item was assigned an outcome name using the International Classification of Functioning and following standard linking rules,[24].

For peer review only

X categorized all outcomes into an outcome taxonomy developed by COMET for categorizing outcomes for core outcome set development,[25]. These included 5 core areas and 38 outcome domains. This is presented in supplementary file 3. A long list of all categorized outcomes was presented to researchers (X and X) at a face to face meeting where the categorization of all outcomes was reviewed using the recommended taxonomy. Subdomains were created within the larger taxonomy to manage the large variation in TBPI clinical outcomes extracted. Disagreements not resolved at this stage were discussed further with subject experts (for example, the Adverse Event domain was discussed with a surgeon).

Due to the diversity in terminology used to report outcomes, we grouped similar outcomes within each subdomain. It is recommended that outcomes with different words, phrasing, or spelling addressing the same concept should be categorized as a unique outcome,[26]. For example, active range of motion of shoulder abduction and active goniometry of shoulder abduction were named as active shoulder abduction range and grasp strength and grip strength were named as grip strength. Independent meetings were held with four subject experts to ratify and define unique outcome names within each domain.

Patient and Public Involvement

The need for a COS in TBPI care was conceived following discussions with patients and health professionals. Patients highlighted the diverse effect the injury has on their life and that often these outcomes were overlooked by professionals, such as body image. There is a patient advisory group for the COS and the systematic review was discussed at these meetings. Patients were not actively involved in data collection or analysis of this review.

For peer review only - http://bmjopen.bmj.com/site/about/guidelines.xhtml

Dissemination will occur at the annual traumatic brachial plexus charity UK meeting where updates from the project are presented yearly and through a six monthly newsletter.

Results

Included studies

The search identified 1159 studies, after removing duplicates 1105 studies remained. Titles and abstract review identified 169 potentially relevant articles. Of these, 37 studies did not meet the inclusion criteria and were excluded (PRISMA flow diagram; figure 1) thus, 132 studies formed the basis of this review. All included studies are presented in supplementary file 4.

Place figure 1 here

Figure 1. Preferred Reporting Items for Systematic Reviews and meta-analysis flow diagram.

Study characteristics

Thirty-two countries from six continents recruited 3201 participants into the 132 studies (Table 1). Of the 132 studies, 87 (66%) were retrospective case series with most studies published from Asia (n=61, 46%). The most frequently studied surgical intervention was nerve transfers (n=66, 57%).

For peer review only

Table 1. Characteristics and demographics of included studies

	Study number (%)
Number of retrospective studies	87/132(66)
Number of prospective studies	21/132 (16)
Number of case studies	23/132(17)
Randomized controlled trial	1/132 (0.8)
**World region recruitment**	
Asia	61/132(46)
North America	20/132(15)
South America	20/132(15)
Europe	27/132(20)
Africa	3/132(2.2)
Australasia	1/132(0.8)
**Year published**	
2013	25/132 (19)
2014	24/132(18)
2015	15/132(11)
2016	30/132(23)
2017	27/132(20)
2018	11/132(8.3)
**Gender (total 3201)**	
Male	2622/3201(82)
Female	323/3201(10)
Not stated	256/3201(7.9)
**Site of plexus injury per study (n=132)**	
Upper trunk	26/132(20)
Lower trunk	10/132(7.6)

For peer review only

For peer review only - http://bmjopen.bmj.com/site/about/guidelines.xhtml

Pan plexus (all avulsed)	50/132(38)
Infraclavicular	7/132(5.3)
Mixture	32/132(24)
Unclear	7/132(5.3)
**Interventions (n=132)**	
Surgical	115/132(87)
Electrotherapy	2/132(1.5)
Pain treatments	11/132 (8.3)
Rehabilitation	2/132(1.5)
Orthotic	1/132(0.7)
Stem cell	1/132(0.7)
**Types of surgical intervention (n=115)**	
Neurotisation	66/115(57)
Tendon transfer	7/115(6.1)
Free flap	16/115(14)
Multiple surgeries	12/115(10)
Contralateral C7	8/115(6.9)
Other	6/115(5.2)

For peer review only

Outcomes

Extraction of each verbatim outcome domain from each study (e.g range of movement and muscle strength) and those extracted from measures composed of several items identified a total of 1460 verbatim outcomes. After removing duplicates 157 different unique outcomes remained. No single outcome was reported across all 132 studies.

Outcome definition variation. Many outcomes were not clearly defined and different terms were frequently found for the same concept. For example, shoulder abduction strength was described in eleven different ways including 'deltoid strength', 'motor function of axillary nerve', 'motor recovery of shoulder abductors', 'muscle power supraspinatus', 'motor function of Deltoid', 'motor function of Supraspinatus'.

Outcome timing variation: Of the 1460 verbatim outcomes, 46% (672) were measured between one and three years following intervention. For 83 outcomes the timing of the measurement was not stated. See Figure 2.

Place Figure 2 here

Figure 2. Timepoints of reported outcomes

For peer review only

Outcome domains: The 157 different types of outcomes were categorized into four core areas (Physiological and Clinical, Life Impact, Resource Use, Adverse Events/Complications) and 11 domains according to the COMET recommendations,[24]. See supplementary file 5. The core area Physiological/Clinical included three domains: musculoskeletal and connective tissue outcomes, nervous system outcomes and general/symptom outcomes. The core area Life Impact included seven domains: physical functioning, social functioning, role functioning, emotional functioning, global quality of life, perceived health status and delivery of care. The core area Resource Use included one domain: hospital resources. The core area Adverse Events included one domain: adverse events. No outcome could be placed into the core area Death.

Tables 2 to 4 summarise the number of unique outcomes within each domain and the number of studies reporting these outcomes in each core area. The most frequently reported domains were all in the Physiological/ Clinical core area and included musculoskeletal and connective tissue (87%), nervous system (35%) and symptoms (36%). Forty-four studies (33%) reported complications/ adverse events.

For peer review only - http://bmjopen.bmj.com/site/about/guidelines.xhtml

Table 2. Physiological /Clinical Core Area

Outcome Domains	Number of unique outcomes reported within domain	Examples of unique outcomes	Number of studies reporting outcomes in domain (%)
Musculoskeletal and connective tissue	18	Active range of movement, muscle strength, muscle fatigue	115/132 (87%)
Nervous system	15	Progression of nerve regeneration, ability to feel light touch, ability to feel pain	46/132 (35%)
General/ symptoms	23	Pain intensity/relief, pain duration, pain quality, pain when arm exposed to cold, stiffness, sleep, paresthesia	47/132 (36%)

For peer review only

Table 3. Life Impact Core Area

**Outcome Domains**	**Number of unique outcomes reported within domain**	**Examples of unique outcomes**	**Number of studies reporting outcomes within domain (%)**
Physical functioning	19	Reaching, fine hand movement	30/132 (23%)
Role functioning	23	Return to work, Impact on normal hobbies	33/132 (25%)
Social functioning	7	Social activities with family	30/132 (23%)
Emotional functioning	13	Body image, acceptance	29/132 (22%)
Global quality of life	1	Quality of life	1/132 (0.8%)
Perceived health Status	1	Health status rating	6/132 (4.5%)
Delivery of care	13	Patient satisfaction, quality of care, patient preference, time to surgery	11/132(8.3%)

For peer review only

Table 4. Adverse Events and Resource Use Core Areas

Outcome Domains	Number of unique outcomes reported within domain	Examples of unique outcomes	Number of studies reporting outcomes within domain
**Adverse Events Core Area**			
Donor site morbidity	3	Motor weakness, sensory loss	24/132(18%)
Musculoskeletal	7	Co -contraction, Passive movement	12/132 (9%)
Respiratory	4	Pneumothorax	6/132 (4.5%)
Vascular	7	Hematoma	7/132 (5.3%)
Infection	1	Infection	3/132 (2.3%)
General non specified complications	1	General complications	2/132 (1.5%)
**Resource Use Core Area**			
Hospital resource use	1	Operation time	1/132 (.75%)

For peer review only

Outcome Measurement

In addition to extraction of standalone clinician reported and patient reported outcomes such as muscle power, range or movement or return to work, outcomes were also extracted from individual items contained in a total of 30 different instruments; PRO measures (n= 20), combined clinician-reported and patient-reported measures (n= 3) and performance measures (n= 7). See table 5. These measures were reported 83 times in the included publications. Most outcome measures were used once (n= 25, 30%). The most frequently reported measures were the Disabilities of the Arm Shoulder and Hand (DASH,[27]) questionnaire (n=27 studies, 32%) and the Visual Analogue Scale (n=18, 22%). The median number of items per instrument was 15 ranging from one (Visual Analogue Scale, Numerical Rating Scale and Wong Baker Faces rating scale),[28] to 54,[29]. These items mapped to 34 different outcome domains.

There was wide variation in the methods used to measure outcomes. This is presented in supplementary file 6 (Measurement instruments mapped to domains). For example; 62 different measurements were used to evaluate muscle function, including the British Medical Research Council,[30] eleven different modifications of the British Medical Council, Isokinetics, Dynanometry and Constant - Murley score,[31]. In addition, it was often not clear which instrument was used for measurement of the outcomes. For example, the instrument used to measure active range of movement was not reported in 36% of total times (63/ 174) the outcome was assessed. Finally with regards to method of measurement 55 studies employed a PRO instrument to evaluate the intervention.

For peer review only

For peer review only - http://bmjopen.bmj.com/site/about/guidelines.xhtml

Table 5: Outcome measures used in included studies

		**Number of items**	**Number of scales**	**Frequency (n=83)**
PRO Measures	**Upper limb physical function measures (n= 16)**			
	Disabilities of Arm Shoulder and Hand	38	3	27
	Upper Extremity Functional Index	20	0	2
	American Shoulder and Elbow Score	15	0	1
	Modified American Shoulder and Elbow Score	13	0	1
	Simple Shoulder test	12	0	1
	Michigan Hand Questionnaire	37	0	1
PRO & ClinRO Measure	University of California Los Angelus shoulder score	5	0	1
	Constant- Murley	5	0	1
	MAYO Performance Index	4	0	1
Performance Measures	Jebsen Taylor	7	0	1
	University of New Brunswick Test of Prosthetic Function for Unilateral Amputees (UNB)	30	3	1
	Upper Limb Module Questionnaire	22	3	1
	Action Reach Arm Test	19	4	1
	Southampton Hand Assessment Procedure	26	0	1
	Purdue Peg test	3	0	1
	Activities Measure for Upper Limb Amputees	24	0	1
PRO Measures	**Generic questionnaires (n=2)**			
	36 item short form survey (SF36)	36	8	5
	Patient Specific Functional Score	4	0	1
	**Condition specific questionnaires (n=1)**			

For peer review only

Trinity Amputation and Prosthesis scale	54	5	1
**Symptom specific questionnaires (n=10)**			
Visual Analogue Scale	1	0	18
Numerical Rating Scale	1	0	6
Wong Baker Faces rating scale	1	0	1
Brief pain inventory	15	6	1
Neuropathic pain symptom inventory	10	5	1
University of Washington Neuropathic score	10	3	1
McGill Pain Questionnaire	28	3	1
McGill Pain Questionnaire SF	17	3	1
McGill Pain Questionnaire (Japanese version)	17	3	1
Self- rating anxiety scale	20	0	1
Zung Self rating Depression scale	20	0	1

For peer review only

DISCUSSION

This systematic review aimed to identify what outcome domains have been reported in studies evaluating interventions for TBPI, examine outcome definitions and timepoints and identify the instruments used to assess outcomes. We found a wide variation in reported outcomes, timing of outcomes and outcome instruments used. Furthermore, a lack of standardized definition for commonly reported outcomes was observed. This heterogeneity in outcome reporting across studies hinders evidence synthesis and results in research waste,[32].

The most commonly reported core area was Physiological/ Clinical including musculoskeletal, nervous system and symptom domains. Eighty-seven percent of studies reported musculoskeletal outcomes. However, there were 21 different outcomes reported in this category making comparison between studies difficult. Furthermore, the diversity of measures used to assess the outcomes increases the difficulty with synthesis. For example, muscle function/ strength was assessed using 59 different measures, whilst 10 studies did not report what measure they used. To compound this muscle strength was assessed by both physical examination by a clinician (86%) and also by asking the patient(10%).

Only 42% of studies (55/132) evaluated PROs and within these studies there was significant heterogeneity in the measurement instrument used. Twenty-three different instruments were used with 18 only ever used once. The DASH was the most common instrument employed, in just over half the studies evaluating a PRO. The PRO instruments also varied greatly in terms of content with some as simple as a single item whilst others included up to 54 items. Over 273 individual questionnaire items were evident from the 23 PRO instruments mapping to 34 different outcomes domains. This highlighted a lack of consistency with no domain being measured by all PRO instruments. None of the included PRO assessments were designed specifically for individuals with a TBPI. Although this may be beneficial in terms of comparison with other conditions, such instruments may not be sensitive to issues of importance to patients with TBPI. These issues combined pose major questions regarding the clinical interpretation of results from TBPI studies.

It is clear that that individuals with a TBPI suffer significant emotional and psychoscocial issues,[1,33]. However such issues were infrequently and inconsistently measured within this review. Only one study considered Quality of Life (QoL) as an outcome,[34] using a single item PRO. Similarly, physical, role and social functioning outcomes were reported in 23%, 25% and 23% of studies respectively. This relates strongly to the use of the DASH within the studies. Indeed, emotional functioning was reported in 29 studies, 27 of these studies used the DASH which has one item on confidence and capability mapping to this domain. If the DASH was excluded, only seven studies would assess outcomes within the emotional functioning domain. This is surprising considering the existing literature which

For peer review only - http://bmjopen.bmj.com/site/about/guidelines.xhtml

evidences the complex emotional and psychological factors, individuals face when adjusting to their injury,[1,35].

Complications/adverse events were reported in 33% of studies. Documentation of complications is crucial to improve patient care and gather data for benchmarking. In 1992, the Clavien-Dindo classification,[36] was introduced to assist with classification of complications to enable comparison between studies,[36]. However, within the adverse events outcomes identified in this review there was heterogeneity. Of the 37 verbatim outcomes reported within the donor morbidity (motor) outcome 19 did not define how this was assessed.

There are some limitations. We excluded outcomes from older studies to ensure we identified outcomes relevant to contemporary TBPI care. Formal quality assessment of studies was not undertaken, however the review was designed to identify the breadth of reporting in the literature and not to examine the effectiveness of interventions. The strengths of this review are that the protocol and the data extraction form were prespecified, prospectively registered and the literature search systematic. To account for multidisciplinary perspectives, researchers and clinicians where involved in categorizing outcomes into domains. It is the first review to detail the scale of outcome heterogeneity in TBPI research using a systematic method. International and non-English publications were included to reduce the risk of selection bias.

For peer review only

Variation in definitions and measurement of outcomes has been found within other areas of healthcare. Outcome heterogeneity is found in the reporting of outcomes relating to burn care,[37] breast reconstruction,[38] and spinal cord injury,[39] amongst others. A recent review of outcome reporting within burns illustrated wound healing was defined in 166 different ways across 147 studies,[37]. A solution to the variation in outcome reporting across studies in TBPI is the development of a COS,[20]. This has been shown to improve consistency of outcome reporting,[19,40]. Development of a COS in TBPI would not restrict the range of outcomes that can be measured. Researchers and clinicians would still be free to select additional outcomes but the inclusion of such a COS would facilitate synthesis of evidence,[41,42]. Whilst work has begun in obstetric brachial plexus injuries to develop a minimum data set[43], there is no COS for TBPI.

Considerable work has been done by the Core Outcome Measures in Effectiveness Trials (COMET) initiative through dissemination of resources for COS development and support for methodological development. COMET recommends a five step process to develop a COS: define the scope, assess the need, develop the protocol, determine what to measure and determine how to measure,[44]. This systematic review addresses these first two steps for the development of the COS in TBPI care. This review has shown the majority of TBPI studies use only clinician reported outcomes to evaluate interventions. However they do not adequately capture patients' health related quality of life,[45] and may underestimate the impact of a condition,[46]. Concurrent qualitative work to identify outcomes which are important to individuals with a TBPI has been completed by this group. The next stage involves integration of all potential outcomes from this review and the qualitative work into a long list of domains. Healthcare professionals and patients will be invited to prioritize

For peer review only - http://bmjopen.bmj.com/site/about/guidelines.xhtml

these outcomes during a three round international online Delphi process and consensus meeting. This will strengthen the case for uptake of a COS for TBPI as it represents patients' and clinicians' perspectives on what outcomes are important. The final stage will map existing validated measures to the outcome domains in the final COS. A future study will evaluate the psychometric properties of those mapped measurement instruments and identify if new measures need to be developed.

For peer review only

For peer review only - http://bmjopen.bmj.com/site/about/guidelines.xhtml

CONCLUSION

This systematic review has shown that outcome reporting in TBPI care is heterogenous and impairment focused with a lack of standardized definitions for commonly reported outcomes. This makes it difficult to compare and combine data from studies to inform decision making in clinical practice. The measurement instruments used in the studies were also often not clear, particularly when range of movement was assessed. In future studies, authors need to be clearer with descriptions of outcomes assessed and how they were measured. Less than half the studies in this review evaluated outcomes using PRO measures. Given that TBPI has a significant impact on health-related quality of life, it is recommended that authors of future studies include PROs in future studies. We have identified a list of potentially relevant outcomes and categorized these into a clear taxonomy. This will inform the next stage of developing a COS for TBPI where patients, surgeons and therapists will be involved in a consensus process to decide the final outcomes included in a COS for TBPI.

Acknowledgements

We would like to thank Colin Shirley for his assistance and guidance categorising neurophysiological outcomes

For peer review only - http://bmjopen.bmj.com/site/about/guidelines.xhtml

Competing Interests

Conflicting interests: CM, CJH, JC, DMP and JOS declare no potential conflicts of interest with respect to the research, authorship and publication of this article. DGK reports grants from NIHR, grants from Innovate UK, grants from NIHR Birmingham Biomedical Research Centre, grants from NIHR SRMRC at the University of Birmingham and University Hospitals Birmingham NHS Foundation Trust, personal fees from Merck, personal fees from GSK, grants from Macmillan Cancer Support, grants from Kidney research UK, outside the submitted work; .

Funding

The work was supported by the National Institute of Health Research [grant number ICA-CDRF-2017-03-039]

Data availability

All data relevant to the study are included in the article or uploaded as supplementary information.

Ethical approval

Ethical approval was not sought for the present study because it was a systematic review and did not involve human participation

For peer review only

For peer review only - http://bmjopen.bmj.com/site/about/guidelines.xhtml

Informed consent

Informed consent was not sought for the present study because it was a systematic review and did not involve human participation

Contributorship

CM, CJH and JC conceived and designed the review. CM and JOS reviewed the titles, abstracts and full text papers for eligibility. Authors resolved disagreements by discussion or where necessary CJH and DMP offered their view. CM and JOS were responsible for extracting data and data extraction was verified by CJH. CM, CJH and JC categorised outcomes. Categorisation was reviewed and edited by DMP and DK. CM prepared the manuscript. CJH,JC, DMP, DK and JOS reviewed and edited the manuscript.

For peer review only

For peer review only - http://bmjopen.bmj.com/site/about/guidelines.xhtml

References

[1] Brito S, White J, Thomacos N, et al. The lived experience following free functioning muscle transfer for management of pan-brachial plexus injury: reflections from a long-term follow-up study. *Disabil Rehabil* 2019;1:1–9.

[2] Morris M, Daluiski A, Dy C. A thematic analysis of online discussion boards for brachial plexus injury. *J Hand Surg AM* 2016;41(8):813-8.

[3] Kaiser R, Waldauf P, Ullas G, et al. Epidemiology, etiology, and types of severe adult brachial plexus injuries requiring surgical repair: systematic review and meta-analysis. Neurosurg Rev 2020;43(2):443-452.

[4] Moran CG, Lecky F, Bouamra O, et al. Changing the system - major trauma patients and their outcomes in the NHS (England) 2008-17. *EClinicalMedicine* 2018;13–21.

[5] Dy CJ, Peacock K, Olsen MA, et al. Incidence of surgically treated brachial plexus injury in privately insured adults under 65 years of age in the USA. *Hosp Spec Surg J* 2020;online.

[6] Midha R. Epidemiology of brachial plexus injuries in a multitrauma population. *Neurosurgery* 1997;40:1182–8

[7] Felici N, Zaami S, Ciancolini G, et al. Cost analysis of brachial plexus injuries: Variability of compensation by insurance companies before and after surgery. *Handchir Mikrochir Plast Chir* 2014;46:85–9.

[8] Hong TS, Tian A, Sachar R, et al. Indirect cost of traumatic brachial plexus injuries in the United States. *J Bone Joint Surg Am* 2019;101:e80.

[9] Bhandari PS, Maurya S. Recent advances in the management of brachial plexus

For peer review only - http://bmjopen.bmj.com/site/about/guidelines.xhtml

injuries. *Indian J Plast Surg* 2014;47:191–8.

[10] Kubota S, Hara Y, Shimizu Y, et al. A newly developed upper limb single-joint HAL in a patient with elbow flexion reconstruction after traumatic brachial plexus injury: A case report. *Interdiscip Neurosurg Adv Tech Case Manag* 2017;10:66–8.

[11] Dickinson HD. Evidence-based decision-making: an argumentative approach. *Int J Med Inform* 1998;51:71–81.

[12] Sackett DL, Rosenberg WM, Gray JA, et al . Evidence based medicine: what it is and what it isn't. *BMJ* 1996;312:71–2.

[13] Ayhan E, Soldado F, Fontecha CG, et al. Elbow flexion reconstruction with nerve transfer or grafting in patients with brachial plexus injuries: A systematic review and comparison study. *Microsurgery* 2020;40:79–86.

[14] Donnelly MR, Rezzadeh KT, Vieira D et al. Is one nerve transfer enough? A systematic review and pooled analysis comparing ulnar fascicular nerve transfer and double ulnar and median fascicular nerve transfer for restoration of elbow flexion after traumatic brachial plexus injury. *Microsurgery* 2020;40:361–9.

[15] Dy CJ, Garg R, Lee SK, et al. A systematic review of outcomes reporting for brachial plexus reconstruction. *J Hand Surg Am* 2015;40:308–13.

[16] Hill B, Williams G, Olver J, et al. Letter regarding outcome reporting for brachial plexus reconstruction. *J Hand Surg Am* 2015;40:1504.

[17] Gargon E, Gurung B, Medley N, et al. Choosing important health outcomes for comparative effectiveness research: a systematic review. *PLoS One* 2014;17(7):A435

[18] Williamson P, Altman D, Blazeby J et al. Driving up the quality and relevance of research through the use of agreed core outcomes. *J Health Serv Res Policy* 2012;17:1–2.

For peer review only

For peer review only - http://bmjopen.bmj.com/site/about/guidelines.xhtml

[19] Kirkham JJ, Bracken M, Hind L, et al. Industry funding was associated with increased use of core outcome sets. *J Clin Epidemiol* 2019;115:90–7.

[20] Williamson PR, Altman DG, Blazeby JM et al. The COMET (Core Outcome Measures in Effectiveness Trials) Initiative. *Trials* 2011;12:A70.

[21] Higgins J, Green S. Cochrane Handbook for Systematic Reviews of Interventions Version (updated March 2011). version 5. 2011.

[22] Shamseer L, Moher D, Clarke M, et al. Preferred reporting items for systematic review and meta-analysis protocols (PRISMA-P) 2015: elaboration and explanation. *BMJ* 2015;349:g7647.

[23] Richardson E, Burnell J, Adams HR, et al. Developing and Implementing Performance Outcome Assessments: Evidentiary, Methodologic, and Operational Considerations. *Ther Innov Regul Sci.*2019;53(1):146-153.

[24] Cieza A, Geyh S, Chatterji S, et al. ICF linking rules: an update based on lessons learned. *J Rehabil Med* 2005;37:212–8.

[25] Dodd S, Clarke M, Becker L, et al. A taxonomy has been developed for outcomes in medical research to help improve knowledge discovery. *J Clin Epidemiol* 2018;96:84–92.

[26] Young AE, Brookes ST, Avery KNL, et al. A systematic review of core outcome set development studies demonstrates difficulties in defining unique outcomes. *J Clin Epidemiol* 2019;115:14–24.

[27] Hudak PL, Amadio PC, Bombardier C, et al. Development of an upper extremity outcome measure: The DASH (disabilities of the arm, shoulder, and head). *Am J Ind Med* 1996;29:602–8.

[28] Baker C, Wong D. Q.U.E.S.T: a process of pain assessment in children (continuing

For peer review only

eductation credit). *Orthop Nurs* 1987;6:11–21.

[29] Gallagher P, MacLachlan M. Development and psychometric evaluation of the trinity amputation and prosthesis experience scales (TAPES). *Rehabil Psychol* 2000;45:130–54.

[30] Medical Research Council. Aids to the investigation of the peripheral nervous system. London: Her Majesty's stationary office; 1943.

[31] Constant CR, Murley AH (1987) A clinical method of functional assessment of the shoulder. *Clinical Orthopaedics & Related Research* 1987;214:160-164

[32] Chalmers I, Glasziou P. Avoidable waste in the production and reporting of research evidence. *Lancet* 2009;374:86–9.

[33] McDonald J, Pettigrew J. Traumatic brachial plexus injury: the lived experience. *British Journal of Occupational Therapy* 2014;77(3):147-154.

[34] Kim JH, Shin SH, Lee YR, et al. Ultrasound-guided peripheral nerve stimulation for neuropathic pain after brachial plexus injury: two case reports. *J Anesth* 2017;31:453–7.

[35] Miller C, Peek AL, Power D, et al. Psychological consequences of traumatic upper limb peripheral nerve injury: A systematic review. *Hand Ther* 2017;22:35–45.

[36] Clavien P, Sanabria J, Strasberg S. Proposed classification of complications of surgery with examples of utility in cholecystectomy. *Surgery* 1992;111(5):518-26

[37] Young AE, Davies A, Bland S et al. Systematic review of clinical outcome reporting in randomised controlled trials of burn care. *BMJ Open* 2019;15;9(2):e025135

[38] Potter S, Brigic A, Whiting PF, et al. Reporting clinical outcomes of breast reconstruction: a systematic review. *J Natl Cancer Inst* 2011;103:31–46.

[39] Watzlawick R, Antonic A, Sena ES, et al. Outcome heterogeneity and bias in acute

For peer review only

For peer review only - http://bmjopen.bmj.com/site/about/guidelines.xhtml

experimental spinal cord injury A meta-analysis. *Neurology* 2019;2;93(1):e40-e51

[40] Bautista-Molano W, Navarro-Compán V, Landewé RBM, et al. How well are the ASAS/OMERACT core outcome sets for ankylosing spondylitis implemented in randomized clinical trials? A systematic literature review. *Clin Rheumatol* 2014;33:1313–22.

[41] Clarke M, Williamson PR. Core outcome sets and systematic reviews. *Syst Rev* 2016;5:11.

[42] Clarke M. Standardising outcomes for clinical trials and systematic reviews. *Trials* 2007;8:39.

[43] Pondaag W, Malessy MJA. Outcome assessment for Brachial Plexus birth injury. Results from the iPluto world-wide consensus survey. *J Orthop Res* 2018;36:2533–41.

[44] Williamson PR, Altman DG, Bagley H, et al. The COMET Handbook: version 1.0. *Trials* 2017;18:280.

[45] Pakhomov S, Jacobsen SJ, Chute CG, et al. Agreement between patient-reported symptoms and their documentation in the medical record. *Am J Manag Care* 2008;14:530–9.

[46] Turner GM, Slade A, Retzer A, et al. An Introduction to Patient Reported Outcome Measures (PROMs) in Trauma. *J Trauma Acute Care Surg* 2019;86(2):314-320

Figure 2 Legends

mths, months; NS, not stated; yrs, years.

For peer review only - http://bmjopen.bmj.com/site/about/guidelines.xhtml

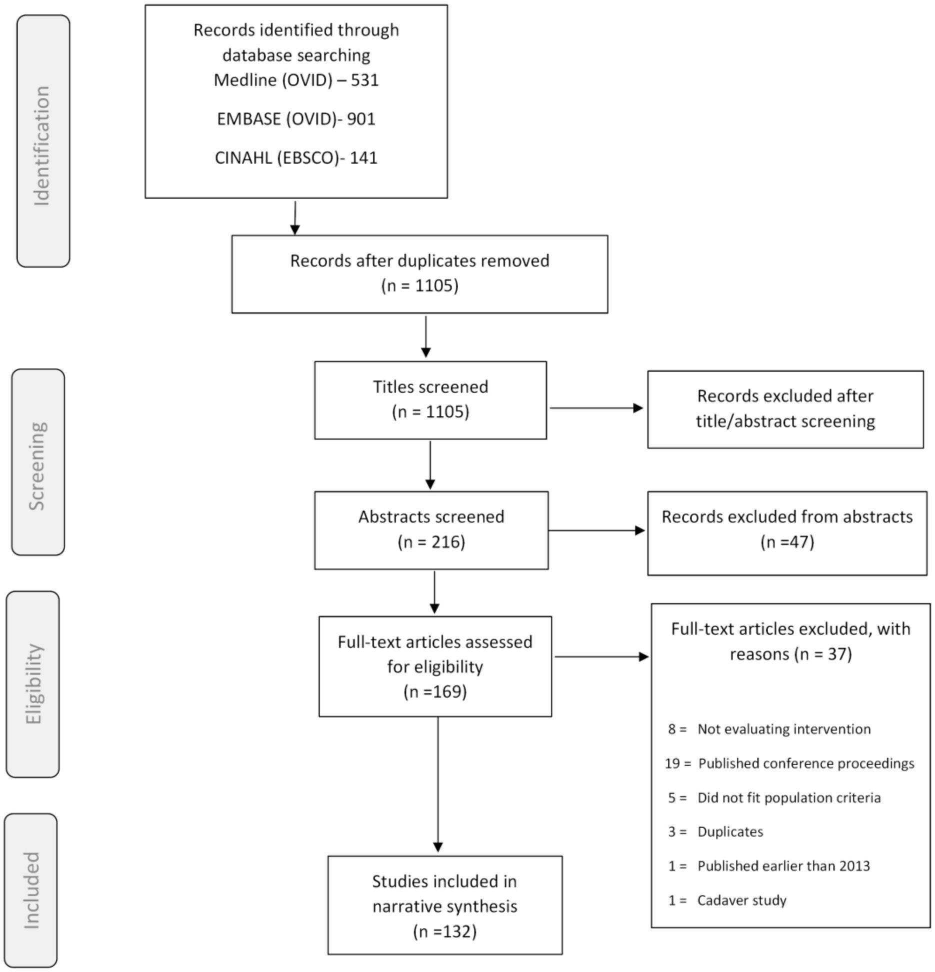

Figure 1. Preferred Reporting Items for Systematic Reviews and meta-analysis flow diagram.

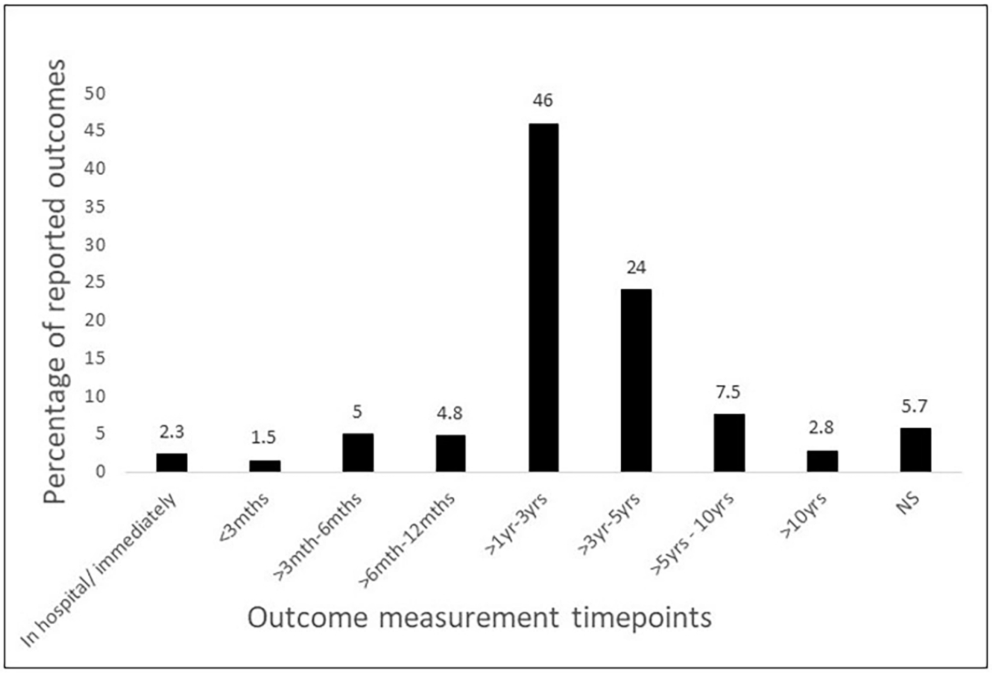

Figure 2. Timepoints of reported outcomes

Developing a core outcome set for traumatic brachial plexus injuries: a systematic review of outcomes

Appendix S1. Deviations from study protocol

Protocol method	Deviation from protocol method with justification
We planned to hand search Journal of Hand Surgery (Eur) and The Journal of Hand Surgery (American).	We did not hand search these Journals as they were all indexed for MEDLINE.
We planned to include studies with participants aged 18 and over within the review.	We reduced the age of include participants to 16 or over as many studies included older teenagers with adults in their studies. On discussion with the research team we concluded that there was no difference between treatment of those aged 16 and over versus aged 18. If we excluded these studies many outcomes used across these age ranges would have been lost.

For peer review only

Title: Supplementary File 2 MEDLINE (OVID) search strategy

Article title: Developing a core outcome set for Traumatic Brachial Plexus Injuries: a systematic review of outcomes
Author: Miller et al (2020)

Search strategy 18/09/2018 COMBINE systematic review

MEDLINE (OVID)

1.(brachial plexus adj3 injur*).mp. [mp=title, abstract, original title, name of substance word, subject heading word, floating sub-heading word, keyword heading word, protocol supplementary concept word, rare disease supplementary concept word, unique identifier, synonyms]

2 (brachial plexus adj3 pals*).mp. [mp=title, abstract, original title, name of substance word, subject heading word, floating sub-heading word, keyword heading word, protocol supplementary concept word, rare disease supplementary concept word, unique identifier, synonyms]

3 (brachial plexus adj3 lesion*).mp. [mp=title, abstract, original title, name of substance word, subject heading word, floating sub-heading word, keyword heading word, protocol supplementary concept word, rare disease supplementary concept word, unique identifier, synonyms]

4 brachial plexopath*.mp. [mp=title, abstract, original title, name of substance word, subject heading word, floating sub-heading word, keyword heading word, protocol supplementary concept word, rare disease supplementary concept word, unique identifier, synonyms]

5 (brachial plexus adj3 traction*).mp. [mp=title, abstract, original title, name of substance word, subject heading word, floating sub-heading word, keyword heading word, protocol supplementary concept word, rare disease supplementary concept word, unique identifier, synonyms]

6 (brachial plexus adj3 avulsion*).mp. [mp=title, abstract, original title, name of substance word, subject heading word, floating sub-heading word, keyword heading word, protocol supplementary concept word, rare disease supplementary concept word, unique identifier, synonyms]

7 Brachial Plexus/in, su, tr [Injuries, Surgery, Transplantation]

8 1 or 2 or 3 or 4 or 5 or 6 or 7

9 limit 8 to (humans and "all adult (19 plus years)")

10. limit 9 to yr= “2013- current”

For peer review only

Supplementary file 3: COMET outcome taxonomy
Article title: Developing a core outcome set for Traumatic Brachial Plexus Injuries: a systematic review of outcomes

Title: Supplementary file 3: COMET outcome taxonomy - adapted from Dodd et al (2018)

**Core Area**	Outcome Domain
**Death**	1. Mortality/ survival
**Physiological/clinical**	2. Blood and lymphatic system outcomes
	3. Cardiac outcomes
	4. Congenital, familial and genetic outcomes
	5. Endocrine outcomes
	6. Ear and labyrinth outcomes
	7. Eye outcomes
	8. Gastrointestinal outcomes
	9. General outcomes
	10. Hepatobilary outcomes
	11. Immune system outcomes
	12. Infection and infestation outcomes
	13. Injury and poisoning outcomes
	14. Metabolism and nutrition outcomes
	15. Musculoskeletal and connective tissue outcomes
	16. Outcomes, relating to neoplasms: benign, malignant and unspecified (including cysts and polyps)
	17. Nervous system outcomes
	18. Pregnancy, puerperium and perinatal outcomes
	19. Renal and urinary outcomes
	20. Reproductive system and breast outcomes
	21. Psychiatric outcomes
	22. Respiratory, thoracic and mediastinal outcomes
	23. Skin and subcutaneous tissue outcomes
	24. Vascular outcomes
**Life Impact**	**Functioning**
	25. Physical functioning
	26. Social functioning
	27. Role functioning
	28. Emotional functioning/ well being
	29. Cognitive functioning
	30. Global quality of life
	31. Perceived health status
	32. Delivery of care
	33. Personal circumstances
**Resource use**	**Resource Use**
	34. Economic
	35. Hospital
	36. Need for further intervention
	37. Societal/ carer burden
**Adverse Events**	38. Adverse Events / effects

Dodd S, Clarke M, Becker L et al. A taxonomy has been developed for outcomes in medical research to help improve knowledge discovery. *J Clin Epidemiol*. 2018;96:84-92.

For peer review only

Supplementary file 4. Included Studies

	Study title	First author	Year of publication
1	Effectiveness and safety of home-based muscle electrical stimulator in brachial plexus Injury patient(Limthongthang et al., 2014)	Limthongthang	2014
2	Elbow proprioception sense in total arm -type brachial plexus injured patients after neurotisation: a preliminary study(Homsreprasert et al., 2014)	Homreprasert	2014
3	Comparison between the anterior and posterior approach for transfer of the spinal accessory nerve to the suprascapular nerve in late traumatic brachial plexus injuries (Souza et al., 2014)	Souza	2014
4	Ultrasound-guided peripheral nerve stimulation for neuropathic pain after brachial plexus injury: two case reports(Kim et al., 2017)	Kim	2017
5	Contralateral lower trapezius transfer for restoration of shoulder external rotation in traumatic brachial plexus palsy: preliminary report and literature review(Satbhai et al., 2014)	Satbhai	2014
6	Restoration of shoulder abduction in brachial plexus avulsion injuries with double neurotization from the spinal accessory nerve: a report of 13 cases(Huan et al., 2017)	Huan	2017
7	Transfer of the musculocutaneous nerve branch to the brachialis muscle to the triceps for elbow extension: anatomical study and report of five cases(Bertelli et al., 2017)	Bertelli	2017
8	Posterior approach for accessory to suprascapular nerve transfer: an electrophysiological outcomes study(Rui et al., 2013)	Rui	2013
9	Reliability of functioning free muscle transfer and vascularized ulnar nerve grafting for elbow flexion in complete brachial plexus palsy (Potter and Ferris, 2017)	Potter	2017
10	Management of infraclavicular (Chuang Level IV) brachial plexus injuries: A single surgeon experience with 75 cases (Lam et al., 2015)	Lam	2015
11	Functioning free muscle transfer for the restoration of elbow flexion in brachial plexus injury patients (Estrella and Montales 2016)	Estrella	2016
12	Radial to axillary nerve transfers: A combined case series(Desai et al., 2016)	Desai	2016
13	Thalamic deep brain stimulation for neuropathic pain after amputation or brachial plexus avulsion(Pereira et al., 2013)	Pereira	2013
14	Nerve transfers for shoulder function for traumatic brachial plexus injuries(Estrella et al., 2014)	Estrella	2014
15	Results of operative treatment of brachial plexus injury resulting from shoulder dislocation: A study with a long-term follow-up(Gutkowska et al., 2017)	Gutkowska	2017
16	Surgical treatment of brachial plexus posterior cord lesion: A combination of nerve and tendon transfers, about nine patients(Oberlin., 2013)	Oberlin	2013
17	The medial cord to musculocutaneous (MCMc) nerve transfer: a new method to reanimate elbow flexion after C5-C6-C7-(C8) avulsive injuries of the brachial plexus—technique and results(Ferraresi et al., 2014)	Ferraresi	2014

Article title: Developing a core outcome set for Traumatic Brachial Plexus Injuries: a systematic review of outcomes

For peer review only - http://bmjopen.bmj.com/site/about/guidelines.xhtml

Supplementary file 4. Included Studies

**18**	Transfer of a terminal motor branch nerve to the flexor carpi ulnaris for triceps reinnervation: anatomical study and clinical cases(Bertelli et al., 2015)	Bertelli	2015
**19**	Free functioning gracilis muscle transfer with and without simultaneous intercostal nerve transfer to musculocutaneous nerve for restoration of elbow flexion after traumatic adult brachial pan-plexus injury(Maldonado et al., 2017a)	Maldonado	2017(a)
**20**	Isolated latissimus dorsi transfer to restore shoulder external rotation in adults with brachial plexus injury(Ghosh et al., 2013)	Ghosh	2013
**21**	Functional outcome and quality of life after traumatic total brachial plexus injury treated by nerve transfer or single/double free muscle transfers(Satbhai et al., 2016)	Satbhai	2016
**22**	Successful graded mirror therapy in a patient with chronic deafferentation pain in whom traditional mirror therapy was ineffective: A case report(Mibu et al., 2016)	Mibu	2016
**23**	Bipolar Transfer of Latissimus Dorsi Myocutaneous Flap for Restoration of Elbow Flexion in Late Traumatic Brachial Plexus Injury: Evaluation of 13 Cases(Azab et al., 2017)	Azab	2017
**24**	Comparison of objective muscle strength in C5-C6 and C5-C7 brachial plexus injury patients after double nerve transfer (Tsai et al. 2015)	Tsai	2014
**25**	Phantom remodeling effect of dorsal root entry zone lesioning in phantom limb pain caused by brachial plexus avulsion(Son et al., 2015)	Son	2015
**26**	Comparison of surgical strategies between proximal nerve graft and/or nerve transfer and distal nerve transfer based on functional restoration of elbow flexion: A retrospective review of 147 patients(Hu et al., 2018)	Hu	2018
**27**	Reconstruction of shoulder abduction by multiple nerve fascicle transfer through posterior approach(Ren et al., 2013)	Ren	2013
**28**	Intercostal nerve transfer to neurotize the musculocutaneous nerve after traumatic brachial plexus avulsion: A comparison of two, three, and four nerve transfers(Xiao et al., 2014)	Xiao	2014
**29**	Use of the DEKA Arm for amputees with brachial plexus injury: A case series(Resnik et al., 2017)	Resnik	2017
**30**	Polyester tape scapulopexy for chronic upper extremity brachial plexus injury(Leechavengvongs et al., 2015)	Leechavengvongs	2015
**31**	Contralateral C7 nerve transfer with direct coaptation to restore lower trunk function after traumatic brachial plexus avulsion(Wang et al., 2013)	Wang	2013
**32**	Outcome of surgical reconstruction after traumatic total brachial plexus palsy(Dodakundi et al., 2013)	Dodakundi	2013
**33**	Bionic reconstruction to restore hand function after brachial plexus injury: a case series of three patients(Aszmann et al., 2015)	Aszmann	2015
**34**	Surgical treatment of the plexus brachialis injury using long-lasting electrostimulation (Tsymbaliuk and Tretiak, 2013)	Tsymbalyuk	2013
**35**	Phrenic nerve transfer for reconstruction of elbow extension in severe brachial plexus injuries(Flores and Socolovsky, 2016)	Flores	2016

Article title: Developing a core outcome set for Traumatic Brachial Plexus Injuries: a systematic review of outcomes

For peer review only - http://bmjopen.bmj.com/site/about/guidelines.xhtml

For peer review only

Supplementary file 4. Included Studies

**36**	Direct coaptation of the phrenic nerve with the posterior division of the lower trunk to restore finger and elbow extension function in patients with total brachial plexus injuries(Wang et al., 2016)	Wang	2016
**37**	A prospective study comparing single and double fascicular transfer to restore elbow flexion after brachial plexus injury(Martins et al., 2013)	Martins	2013
**38**	Chronic post-traumatic neuropathic pain of brachial plexus and upper limb: a new technique of peripheral nerve stimulation(Stevanato et al., 2014)	Stevanato	2014
**39**	Effectiveness of contralateral C7 nerve root and multiple nerve transfer for treatment of brachial plexus root avulsion(Wei et al., 2014)	Wei	2014
**40**	Combined proximal nerve graft and distal nerve transfer for a posterior cord brachial plexus injury(Plate et al., 2013)	Plate	2013
**41**	The role of elective amputation in patients with traumatic brachial plexus injury(Maldonado et al., 2016b)	Maldonado	2016
**42**	Early microsurgical management of clavicular fracture combined with brachial plexus injury(Liu et al., 2014)	Liu	2014(a)
**43**	Contralateral trapezius transfer to restore shoulder external rotation following adult brachial plexus injury (Elhassan et al., 2016)	Elhassan	2016
**44**	Comparative study of phrenic nerve transfers with and without nerve graft for elbow flexion after global brachial plexus injury(Liu et al., 2014)	Liu	2014
**45**	Shoulder and elbow recovery at 2 and 11 years following brachial plexus reconstruction(Wang et al., 2016)	Wang	2016
**46**	Functional outcomes after treatment of traumatic brachial plexus injuries: clinical study(Aras et al., 2013)	Aras	2013
**47**	Free gracilis transfer reinnervated by the nerve to the supinator for the reconstruction of finger and thumb extension in longstanding C7-T1 brachial plexus root avulsion(Soldado et al., 2013)	Soldado	2013
**48**	Restoration of hand function in C7–T1 brachial plexus palsies using a staged approach with nerve and tendon transfer(Zhang et al., 2014)	Zhang	2014
**49**	Neurotization to innervate the deltoid and biceps: 3 cases(Dy et al., 2013)	Dy	2013
**50**	Arthroscopic arthrodesis of the shoulder in brachial plexus palsy(Lenoir et al., 2017)	Lenoir	2017
**51**	Outcome of contralateral C7 nerve transferring to median nerve(Kai-ming Gao et al., 2013)	Gao	2013
**52**	Intercostal nerve transfer to the biceps motor branch in complete traumatic brachial plexus injuries (Cho et al., 2015)	Cho	2015
**53**	Tactile feedback for relief of deafferentation pain using virtual reality system: a pilot study(Sano et al., 2016)	Sano	2016
**54**	Functioning free gracilis transfer to reconstruct elbow flexion and quality of life in global brachial plexus injured patients(Yang et al., 2016)	Yang	2016

Article title: Developing a core outcome set for Traumatic Brachial Plexus Injuries: a systematic review of outcomes

For peer review only - http://bmjopen.bmj.com/site/about/guidelines.xhtml

Supplementary file 4. Included Studies

**55**	Evaluation of infraspinatus reinnervation and function following spinal accessory nerve to suprascapular nerve transfer in adult traumatic brachial plexus injuries(Baltzer et al., 2017)	Baltzer	2017
**56**	Anatomic study of the intercostal nerve transfer to the suprascapular nerve and a case report(Hu et al., 2014)	Hu	2014
**57**	Shoulder abduction and external rotation restoration with nerve transfer(Kostas-Agnantis et al., 2013)	Kostas-Agnantis	2013
**58**	Contralateral C-7 transfer: is direct repair really superior to grafting?(Bhatia et al., 2017)	Bhatia	2017
**59**	Impact of phrenic nerve paralysis on the surgical outcome of intercostal nerve transfer(Kita et al., 2015)	Kita	2015
**60**	Flow-through anastomosis using a T-shaped vascular pedicle for gracilis functioning free muscle transplantation in brachial plexus injury(Hou et al., 2015)	Hou	2015
**61**	Free functional muscle transfer tendon insertion secondary advancement procedure to improve elbow flexion(Sechachalam et al., 2017)	Sechachalam	2017
**62**	Dual nerve transfers for restoration of shoulder function after brachial plexus avulsion injury(Chu et al., 2016)	Chu	2016
**63**	Cortical plasticity after brachial plexus injury and repair: a resting-state functional MRI study(Bhat et al., 2017)	Bhat	2017
**64**	Results of spinal accessory to suprascapular nerve transfer in 110 patients with complete palsy of the brachial plexus(Bertelli et al., 2016)	Bertelli	2016
**65**	Magnetic resonance neurographic and clinical long-term results after oberlins transfer for adult brachial plexus injuries(Frueh et al., 2017)	Frueh	2017
**66**	Free functioning gracilis muscle transfer versus intercostal nerve transfer to musculocutaneous nerve for restoration of elbow flexion after traumatic adult brachial pan-plexus injury(Maldonado et al., 2016a)	Maldonado	2016
**67**	Results of wrist extension reconstruction in C5–8 brachial plexus palsy by transferring the pronator quadratus motor branch to the extensor carpi radialis brevis muscle(Bertelli et al., 2016)	Bertelli	2016
**68**	Donor nerve sources in free functional gracilis muscle transfer for elbow flexion in adult brachial plexus injury(Nicoson et al., 2017)	Nicoson	2017
**69**	Use of contralateral spinal accessory nerve for ipsilateral suprascapular neurotization in global brachial plexus injury: a new technique(Bhandari and Deb, 2016)	Bhandari	2016
**70**	Objective evaluation of elbow flexion strength and fatigability after nerve transfer in adult traumatic brachial plexus injuries (Maricq et al., 2014)	Marciq	2014
**71**	Outcomes of muscle brachialis transfer to restore finger flexion in brachial plexus palsy(DeGeorge et al., 2017)	DeGeorge	2017
**72**	Functional outcome of nerve transfers for traumatic global brachial plexus avulsion(Liu et al., 2013)	Liu	2013

Article title: Developing a core outcome set for Traumatic Brachial Plexus Injuries: a systematic review of outcomes

For peer review only - http://bmjopen.bmj.com/site/about/guidelines.xhtml

Supplementary file 4. Included Studies

**73**	Transfer of a flexor digitorum superficialis motor branch for wrist extension reconstruction in C5-C8 root injuries of the brachial plexus: a case series(Bertelli and Ghizoni, 2013)	Bertelli	2013
**74**	Outcome after transfer of intercostal nerves to the nerve of triceps long head in 25 adult patients with total plexus root avulsion injury(KaiMing Gao et al., 2013)	Gao	2013
**75**	Good sensory recovery of the hand in brachial plexus surgery using the intercostobrachial nerve as the donor(Foroni et al., 2017)	Foroni	2017
**76**	The phrenic nerve as a donor for brachial plexus injuries: is it safe and effective? Case series and literature analysis(Socolovsky et al., 2015)	Socolovsky	2015
**77**	Complete avulsion of brachial plexus with associated vascular trauma: Feasibility of reconstruction using the double free muscle technique(Hattori et al., 2013)	Hattori	2013
**78**	Long-term outcome of brachial plexus re-implantation after complete brachial plexus avulsion injury(Kachramanoglou et al., 2017)	Kachramanoglo u	2017
**79**	Force recovery assessment of functioning free muscle transfers using ultrasonography(Kodama et al., 2014)	Kodama	2014
**80**	Rhomboid nerve transfer to the suprascapular nerve for shoulder reanimation in brachial plexus palsy: A clinical report(Goubier and Teboul, 2016)	Goubier	2016
**81**	Outcome of contralateral C7 transfer to two recipient nerves in 22 patients with the total brachial plexus avulsion injury(Kaiming et al., 2013)	Gao	2013
**82**	Comparative study of phrenic and intercostal nerve transfers for elbow flexion after global brachial plexus injury(Yuzhou et al., 2015)	Liu	2015
**83**	Donor-side morbidity after contralateral C-7 nerve transfer: results at a minimum of 6 months after surgery(Li et al., 2016)	Li	2016
**84**	Outcome after brachial plexus injury surgery and impact on quality of life(Rasulic et al., 2017)	Rasulić	2017
**85**	Pronator teres branch transfer to the anterior interosseous nerve for treating C8T1 brachial plexus avulsion: An anatomic study and case report(Yang et al., 2014)	Yang	2014
**86**	Operative treatment with nerve repair can restore function in patients with traction injuries in the brachial plexus(Stiasny et al., 2015)	Stiasny	2015
**87**	Thoracodorsal nerve transfer for triceps reinnervation in partial brachial plexus injuries(Soldado et al., 2016)	Soldado	2016
**88**	Co-infusion of autologous adipose tissue derived neuronal differentiated mesenchymal stem cells and bone marrow derived hematopoietic stem cells, a viable therapy for post-traumatic brachial plexus injury: a case report (Thakkar et al., 2014)	Thakkar	2014
**89**	Long-term clinical outcomes of spinal accessory nerve transfer to the suprascapular nerve in patients with brachial plexus palsy(Emamhadi et al., 2016)	Emamhadi	2016
**90**	Surgical treatment for total root avulsion type brachial plexus injuries by neurotisation: a prospective comparison study between total and hemicontralateral C7 nerve root transfer(Tu et al., 2014)	Tu	2014

Article title: Developing a core outcome set for Traumatic Brachial Plexus Injuries: a systematic review of outcomes

For peer review only - http://bmjopen.bmj.com/site/about/guidelines.xhtml

Supplementary file 4. Included Studies

**91**	Deactivation of distant pain-related regions induced by 20-day rTMS: a case study of one-week pain relief for long-term intractable deafferentation pain (Qiu et al., 2014)	Qiu	2014
**92**	End-to-side neurorrhaphy in brachial plexus reconstruction(Haninec et al., 2013)	Haninec	2013
**93**	Reanimation of elbow extension with medial pectoral nerve transfer in partial injuries to the brachial plexus (Flores., 2013)	Flores	2013
**94**	Early post-operative results after repair of traumatic brachial plexus palsy(Mohammad-Reda., 2013)	Mohammad-Reda	2013
**95**	Satisfied patients after shoulder arthrodesis for brachial plexus lesions even after 20 years of follow-up(van der Lingen et al., 2018)	van der Lingen	2018
**96**	Posterior branch of the axillary nerve transfer to the lateral triceps branch for restoration of elbow extension: case report(Klika et al., 2013)	Kilka	2013
**97**	Clinical analysis of repairing the whole brachial plexus nerve root avulsion by transferring C7 nerve root from the uninjured side(Liu et al., 2014)	Liu	2014
**98**	Bipolar transfer of the pectoralis major muscle for restoration of elbow flexion in 29 cases(Cambon-Binder et al., 2018)	Cambon-Binder	2018
**99**	Thoracodorsal nerve transfer for elbow flexion reconstruction in infraclavicular brachial plexus injuries(Soldado et al., 2014)	Soldado	2014
**100**	Median nerve fascicle transfer versus ulnar nerve fascicle transfer to the biceps motor branch in C5-C6 and C5-C7 brachial plexus injuries: nonrandomised prospective study of 23 consecutive patients(Cho et al., 2014)	Cho	2014
**101**	Free functional muscle transplantation of an anomalous femoral adductor with a very large muscle belly: a case report(Kaizawa et al., 2013)	Kaizawa	2013
**102**	Selective neurotisation of the radial nerve in the axilla using the intercostal nerve to treat complete brachial plexus palsy(Tuohuti et al., 2016)	Tuohuti	2016
**103**	Objective predictors of functional recovery associated with intercostal nerves transfer for triceps reinnervation in global brachial plexus palsy(Flores., 2016)	Flores	2016
**104**	Nerve transfer to relieve pain in upper brachial plexus injuries: does it work? (Emamhadi., 2017)	Emamhadi	2017
**105**	Phrenic nerve transfer versus intercostal nerve transfer for the repair of brachial plexus root avulsion injuries(Abdixbir et al., 2016)	Abdixbir	2016
**106**	End-to-side neurorrhaphy to restore elbow flexion in brachial plexus injury(Limthongthang et al., 2016)	Limthongthang	2016
**107**	Chordata method combined with electrotherapy in functional recovery after brachial plexus injury:report of three clinical cases(De Oliveira et al., 2016)	De Oliveira	2016
**108**	Clinical outcome following transfer of the supinator motor branch to the posterior interosseous nerve in patients with C7-T1 brachial plexus palsy(Xu et al., 2015)	Xu	2015

Article title: Developing a core outcome set for Traumatic Brachial Plexus Injuries: a systematic review of outcomes

For peer review only - http://bmjopen.bmj.com/site/about/guidelines.xhtml

Supplementary file 4. Included Studies

**109**	Transposition of branches of radial nerve innervating supinator to posterior interosseous nerve for functional reconstruction of finger and thumb extension in 4 patients with middle and lower trunk root avulsion injuries of brachial plexus(Wu et al., 2017)	Wu	2017
**110**	Electromyographic findings in gracilis muscle grafts used to augment elbow flexion in traumatic brachial plexopathy(Kazamel and Sorenson, 2016)	Kazamel	2016
**111**	Double distal intraneural fascicular nerve transfers for lower brachial plexus injuries(Li et al., 2016)	Li	2016
**112**	Restoration of elbow and hand function in total brachial plexus palsy with intercostal nerves and C5 root neurotisation. Results in 21 patients(Arnal et al., 2016)	Amal	2016
**113**	The phrenic nerve transfer in the treatment of a septuagenarian with brachial plexus avulsion injury: a case study(Jiang and Lao, 2018)	Jiang	2018
**114**	Outcomes of transferring a healthy motor fascicle from the radial nerve to a branch for the triceps to recover elbow extension in partial brachial plexus palsy(Flores., 2017)	Flores	2017
**115**	Successful nerve transfers for traumatic brachial plexus palsy in a septuagenarian(Johnsen and Wolfe, 2016)	Johnsen	2016
**116**	Free functioning gracilis muscle transfer for elbow flexion reconstruction after traumatic brachial pan-plexus injury: Where is the optimal distal tendon attachment for elbow flexion?(Maldonado et al., 2017b)	Maldonado	2017(b)
**117**	Results of distal nerve transfers in restoration of shoulder function in C5 and C6 root avulsion injury to the brachial plexus (Bhandari., 2017)	Bhandari	2017
**118**	Bipolar dual-lead spinal cord stimulation between two electrodes on the ventral and dorsal sides of the spinal cord: consideration of putative mechanisms(Watanabe et al., 2018)	Watanabe	2018
**119**	Triceps nerve to deltoid nerve transfer after an unsatisfactory intra-plexus neurotisation of the posterior division of the upper trunk(Al-Qattan et al., 2017)	Al-Qattan	2017
**120**	Trapezius muscle transfer for restoration of elbow extension in a traumatic brachial plexus injury(Alrabai et al., 2018)	Alrabai	2018
**121**	Transfer of the radial nerve branch to the extensor carpi radialis brevis to the anterior interosseous nerve to reconstruct thumb and finger flexion(Bertelli., 2015)	Bertelli	2015
**122**	Ultrasound-guided pulse-dose radiofrequency: treatment of neuropathic pain after brachial plexus lesion and arm vascularisation(Magistroni et al., 2014)	Magistroni	2014
**123**	Phrenic nerve transfer to the musculocutaneous nerve for the repair of brachial plexus injury: electrophysiological characteristics(Liu et al., 2015)	Liu	2015
**124**	Postoperative motor deficits following elbow flexion reanimation by nerve transfer(Hanneur et al., 2018)	Hanneur	2018

Article title: Developing a core outcome set for Traumatic Brachial Plexus Injuries: a systematic review of outcomes

For peer review only

For peer review only - http://bmjopen.bmj.com/site/about/guidelines.xhtml

Supplementary file 4. Included Studies

**125**	Comparative study of phrenic and partial ulnar nerve transfers for elbow flexion after upper brachial plexus avulsion-a retrospective clinical analysis(Liu et al., 2018)	Liu	2018
**126**	Contralateral medial pectoral nerve transfer with free gracilis muscle transfer in old brachial plexus injury(Yavari et al., 2018)	Yavari	2018
**127**	MEG-BMI to control phantom limb pain(Yanagisawa et al., 2018)	Yanagisawa	2018
**128**	Complete brachial plexus injury- an amputation dilemma, A case report(Choong and Shalimar, 2015)	Choong	2015
**129**	Reversal of phantom pain and hand-to-face remapping after brachial plexus avulsion(Tsao and Finn, 2016)	Tsao	2016
**130**	A newly developed upper limb single-joint HAL in a patient with elbow flexion reconstruction after traumatic brachial plexus injury: A case report(Kubota et al., 2017)	Kubota	2017
**131**	Free reverse gracilis muscle combined with steindler flexorplasty for elbow flexion reconstruction after failed primary repair of extended upper-type paralysis of the brachial plexus(Bertelli., 2018)	Bertelli	2018
**132**	Multiple nerve and tendon transfers – a new strategy for restoring hand function in a patient with C7-T1 brachial plexus avulsions(Xu et al ., 2017)	Xu	2017

Article title: Developing a core outcome set for Traumatic Brachial Plexus Injuries: a systematic review of outcomes

For peer review only - http://bmjopen.bmj.com/site/about/guidelines.xhtml

Supplementary file 4. Included Studies

References

[1] Limthongthang R, Muennoi P, Phoojaroenchanachai R, Vathana T, Wongtrakul S. Effectiveness and safety of home-based muscle electrical stimulator in brachial plexus injury patients. J Med Assoc Thail. 2014, 97: S56–61.

[2] Homsreprasert T, Limthongthang R, Vathana T, Wongtrakul S. Elbow joint proprioceptive sense in total arm-type brachial plexus injured patients after neurotization: a preliminary study. J Med Assoc Thail. 2014, 97: S103–7.

[3] Souza F, Bernardino S, Filho H, Gobbato PL, Martins HAL, Martins RS, et al. Comparison between the anterior and posterior approach for transfer of the spinal accessory nerve to the suprascapular nerve in late traumatic brachial plexus injuries. Acta Neurochir (Wien) 2014, 156: 2345–9.

[4] Kim JH, Shin SH, Lee YR, Lee HS, Chon JY, Sung CH, et al. Ultrasound-guided peripheral nerve stimulation for neuropathic pain after brachial plexus injury: two case reports. J Anesth. 2017, 31: 453–7.

[5] Satbhai NG, Doi K, Hattori Y, Sakamoto S. Contralateral lower trapezius transfer for restoration of shoulder external rotation in traumatic brachial plexus palsy: a preliminary report and literature review. J Hand Surg Eur. 2014, 39: 861–7.

[6] Huan KWSJ, Tan JSW, Tan SH, Teoh LC, Yong FC. Restoration of shoulder abduction in brachial plexus avulsion injuries with double neurotization from the spinal accessory nerve: a report of 13 cases. J Hand Surg Eur. 2017, 42: 700–5.

[7] Bertelli JA, Soldado F, Ghizoni MF, Rodriguez-Baeza A, J.A. B, F. S, et al. Transfer of the musculocutaneous nerve branch to the brachialis muscle to the triceps for elbow extension: anatomical study and report of five cases. J Hand Surg Eur. 2017, 42: 710–4.

[8] Rui J, Zhao X, Zhu Y, Gu Y, Lao J, J. R, et al. Posterior approach for accessory-suprascapular nerve transfer: an electrophysiological outcomes study. J Hand Surg Eur, 2013, 38: 242–7.

[9] Potter SM, Ferris SI. Reliability of functioning free muscle transfer and vascularized ulnar nerve grafting for elbow flexion in complete brachial plexus palsy. J Hand Surg Eur Vol 2017, 42: 693–9.

[10] Lam WL, Fufa D, Chang N-J, Chuang DC-C. Management of infraclavicular (Chuang Level IV) brachial plexus injuries: A single surgeon experience with 75 cases. J Hand Surg Eur. 2015, 40: 573–82.

[11] Estrella EP, Montales TD. Functioning free muscle transfer for the restoration of elbow flexion in brachial plexus injury patients. Injury. 2016, 47: 2525–33.

[12] Desai MJ, Daly CA, Seiler JG 3rd, Wray WH 3rd, Ruch DS, Leversedge FJ, et al. Radial to Axillary Nerve Transfers: A Combined Case Series. J Hand Surg Am. 2016, 41: 1128–34.

[13] Pereira EAC, Boccard SG, Linhares P, Chamadoira C, Rosas MJ, Abreu P, et al. Thalamic deep brain stimulation for neuropathic pain after amputation or brachial plexus avulsion. Neurosurg Focus. 2013, 35:E7.

[14] Estrella EP, Favila AS Jr. Nerve transfers for shoulder function for traumatic brachial plexus injuries. J Reconstr Microsurg. 2014, 30: 59-64.

Article title: Developing a core outcome set for Traumatic Brachial Plexus Injuries: a systematic review of outcomes

For peer review only - http://bmjopen.bmj.com/site/about/guidelines.xhtml

Supplementary file 4. Included Studies

[15] Gutkowska O, Martynkiewicz J, Mizia S, Bak M, Gosk J. Results of Operative Treatment of Brachial Plexus Injury Resulting from Shoulder Dislocation: A Study with A Long-Term Follow-Up. World Neurosurg. 2017,105:623–31.

[16] Oberlin C, Chino J, Belkheyar Z. Surgical treatment of brachial plexus posterior cord lesion: A combination of nerve and tendon transfers, about nine patients. Chir Main. 2013, 32:141–6.

[17] Ferraresi S, Garozzo D, Basso E, Maistrello L, Lucchin F, Di Pasquale P. The medial cord to musculocutaneous (MCMc) nerve transfer: A new method to reanimate elbow flexion after C5-C6-C7-(C8) avulsive injuries of the brachial plexus - Technique and results. Neurosurg Rev. 2014, 37:321–9.

[18] Bertelli J, Soldado F, Ghizoni MF, Rodriguez-Baeza A, J. B, F. S, et al. Transfer of a terminal motor branch nerve to the flexor carpi ulnaris for triceps reinnervation: Anatomical study and clinical cases. J Hand Surg Am. 2015, 40:2229-2235.

[19] Maldonado AA, Kircher MF, Spinner RJ, Bishop AT, Shin AY. Free Functioning Gracilis Muscle Transfer With and Without Simultaneous Intercostal Nerve Transfer to Musculocutaneous Nerve for Restoration of Elbow Flexion After Traumatic Adult Brachial Pan-Plexus Injury. J Hand Surg Am 2017, 42: 293.

[20] Ghosh S, Singh VK, Jeyaseelan L, Sinisi M, Fox M, S. G, et al. Isolated latissimus dorsi transfer to restore shoulder external rotation in adults with brachial plexus injury. Bone Jt J. 2013, 95: 660–3.

[21] Satbhai NG, Doi K, Hattori Y, Sakamoto S, N.G. S, K. D, et al. Functional outcome and quality of life after traumatic total brachial plexus injury treated by nerve transfer or single/double free muscle transfers: A comparative study. Bone Jt J. 2016, 98: 209–17.

[22] Mibu A, Nishigami T, Tanaka K, Osumi M, Tanabe A. Successful Graded Mirror Therapy in a Patient with Chronic Deafferentation Pain in Whom Traditional Mirror Therapy was Ineffective: A Case Report. Pain Pract. 2016, 16: E62–9.

[23] Azab AA-H, Alsabbahi MS, A.A.-H. A, Azab AA-H, Alsabbahi MS. Bipolar Transfer of Latissimus Dorsi Myocutaneous Flap for Restoration of Elbow Flexion in Late Traumatic Brachial Plexus Injury: Evaluation of 13 Cases. Ann Plast Surg. 2017, 78: 198–201.

[24] Tsai Y-J, Su F-C, Hsiao C-K, Tu Y-K. Comparison of objective muscle strength in C5-C6 and C5-C7 brachial plexus injury patients after double nerve transfer. Microsurgery. 2015, 35: 107–14.

[25] Son BC, Ha SW. Phantom Remodeling Effect of Dorsal Root Entry Zone Lesioning in Phantom Limb Pain Caused by Brachial Plexus Avulsion. Stereotact Funct Neurosurg 2015, 93: 240–4.

[26] Hu CH, Chang TN, Lu JC, Laurence VG, Chuang DC. Comparison of Surgical Strategies between Proximal Nerve Graft and/or Nerve Transfer and Distal Nerve Transfer Based on Functional Restoration of Elbow Flexion: A Retrospective Review of 147 Patients. Plast Reconstr Surg. 2018, 141: 68e-79e.

[27] Ren G, Li R, Xiang D, Yu B. Reconstruction of shoulder abduction by multiple nerve fascicle transfer through posterior approach. Injury. 2013, 44: 492–7.

[28] Xiao C, Lao J, Wang T, Zhao X, Liu J, Gu Y. Intercostal nerve transfer to neurotize the musculocutaneous nerve after traumatic brachial plexus avulsion: a comparison of two, three, and four nerve transfers. J Reconstr Microsurg 2014, 30: 297–304.

[29] Resnik L, Fantini C, Latlief G, Phillips S, Sasson N, Sepulveda E, et al. Use of the DEKA Arm for

For peer review only - http://bmjopen.bmj.com/site/about/guidelines.xhtml

Supplementary file 4. Included Studies

amputees with brachial plexus injury: A case series. PLoS One. 2017, 12: e0178642.

[30] Leechavengvongs S, Jiamton C, Uerpairojkit C, Malungpaishorpe K, Witoonchart K, Poonotoke P, et al. Polyester tape scapulopexy for chronic upper extremity brachial plexus injury. J Hand Surg Am. 2015, 40:1184

[31] Wang S, Li P, Xue Y, Yiu H, Li YC, Wang H. Contralateral C7 nerve transfer with direct coaptation to restore lower trunk function after traumatic brachial plexus avulsion. J Bone Joint Surg Am. 2013, 95: 821–2.

[32] Dodakundi C, Doi K, Hattori Y, Sakamoto S, Fujihara Y, Takagi T, et al. Outcome of surgical reconstruction after traumatic total brachial plexus palsy. J Bone Joint Surg Am. 2013, 95:1505–12.

[33] Aszmann OC, Roche AD, Salminger S, Paternostro-Sluga T, Herceg M, Sturma A, et al. Bionic reconstruction to restore hand function after brachial plexus injury: a case series of three patients. Lancet. 2015, 385: 2183–9.

[34] Tsymbaliuk VI, Tretiak IB. [Surgical treatment of the plexus brachialis injury using long-lasting electrostimulation]. Klin Khirurhiia. 2013, 59–61.

[35] Flores LP, Socolovsky M. Phrenic Nerve Transfer for Reconstruction of Elbow Extension in Severe Brachial Plexus Injuries. J Reconstr Microsurg. 2016, 32: 546–50.

[36] Wang S, Li P, Xue Y, Zou J, Li W, Li Y. Direct Coaptation of the Phrenic Nerve With the Posterior Division of the Lower Trunk to Restore Finger and Elbow Extension Function in Patients With Total Brachial Plexus Injuries. Neurosurgery. 2016, 78: 208–14.

[37] Martins RS, Siqueira MG, Heise CO, Foroni L, Teixeira MJ. A prospective study comparing single and double fascicular transfer to restore elbow flexion after brachial plexus injury. Neurosurgery. 2013, 72: 709–15.

[38] Stevanato G, Devigili G, Eleopra R, Fontana P, Lettieri C, Baracco C, et al. Chronic post-traumatic neuropathic pain of brachial plexus and upper limb: a new technique of peripheral nerve stimulation. Neurosurg Rev. 2014, 37: 473–9.

[39] Wei W, Alimujiang-Abulaiti, Tuerxunjiang-Dadihan, Meihua S, Yafei L, Chunxiao Y, et al. [EFFECTIVENESS OF CONTRALATERAL C7 NERVE ROOT AND MULTIPLE NERVES TRANSFER FOR TREATMENT OF BRACHIAL PLEXUS ROOT AVULSION]. Zhongguo Xiu Fu Chong Jian Wai Ke Za Zhi. 2014, 28: 737–40.

[40] Plate JF, Ely LK, Pulley BR, Smith BP, Li Z. Combined proximal nerve graft and distal nerve transfer for a posterior cord brachial plexus injury. J Neurosurg. 2013, 118: 155–9.

[41] Maldonado AA, Kircher MF, Spinner RJ, Bishop AT, Shin AY. The role of elective amputation in patients with traumatic brachial plexus injury. J Plast Reconstr Aesthetic Surg. 2016, 69:311–7.

[42] Liu Y, Wang W, Regmi AM, Ahemaitijiang-Yusufu. Early microsurgical management of clavicular fracture combined with brachial plexus injury. Zhongguo Xiu Fu Chong Jian Wai Ke Za Zhi. 2014a,28: 1329–32.

[43] Elhassan BT, Wagner ER, Spinner RJ, Bishop AT, Shin AY. Contralateral Trapezius Transfer to Restore Shoulder External Rotation Following Adult Brachial Plexus Injury. J Hand Surg Am. 2016, 41: e45-51.

[44] Liu Y, Lao J, Gao K, Gu Y, Zhao X. Comparative study of phrenic nerve transfers with and

Article title: Developing a core outcome set for Traumatic Brachial Plexus Injuries: a systematic review of outcomes

For peer review only - http://bmjopen.bmj.com/site/about/guidelines.xhtml

Supplementary file 4. Included Studies

without nerve graft for elbow flexion after global brachial plexus injury. Injury. 2014b, 45: 227–31.

[45] Wang J-P, Rancy SK, Lee SK, Feinberg JH, Wolfe SW. Shoulder and Elbow Recovery at 2 and 11 Years Following Brachial Plexus Reconstruction. J Hand Surg Am. 2016, 41:173–9.

[46] Aras Y, Aydoseli A, Sabanci PA, Akcakaya MO, Alkir G, Imer M. Functional outcomes after treatment of traumatic brachial plexus injuries: clinical study. Ulus Travma Acil Cerrahi Derg. 2013, 19: 521–8.

[47] Soldado F, Bertelli J, F. S. Free gracilis transfer reinnervated by the nerve to the supinator for the reconstruction of finger and thumb extension in longstanding C7-T1 brachial plexus root avulsion. J Hand Surg Am. 2013, 38: 941–6.

[48] Zhang C-G, Dong Z, Gu Y-D. Restoration of hand function in C7-T1 brachial plexus palsies using a staged approach with nerve and tendon transfer. J Neurosurg. 2014, 121: 1264–70.

[49] Dy CJ, Kitay A, Garg R, Kang L, Feinberg JH, Wolfe SW, et al. Neurotization to innervate the deltoid and biceps: 3 Cases. J Hand Surg Am. 2013, 38:237–40.

[50] Lenoir H, Williams T, Griffart A, Lazerges C, Chammas M, Coulet B, et al. Arthroscopic arthrodesis of the shoulder in brachial plexus palsy. J Shoulder Elb Surg. 2017, 26: e115–21.

[51] Gao K, Lao J, Zhao X, Gu Y. Outcome of contralateral C7 nerve transferring to median nerve. Chin Med J (Engl). 2013, 126: 3865–8.

[52] Cho AB, Iamaguchi RB, Silva GB, Paulos RG, Kiyohara LY, Sorrenti L, et al. Intercostal nerve transfer to the biceps motor branch in complete traumatic brachial plexus injuries. Microsurgery. 2015, 35: 428–31.

[53] Sano Y, Wake N, Ichinose A, Osumi M, Oya R, Sumitani M, et al. Tactile feedback for relief of deafferentation pain using virtual reality system: a pilot study. J Neuroeng Rehabil. 2016, 13:61.

[54] Yang Y, Yang J-T, Fu G, Li X-M, Qin B-G, Hou Y, et al. Functioning free gracilis transfer to reconstruct elbow flexion and quality of life in global brachial plexus injured patients. Sci Rep. 2016, 6: 22479.

[55] Baltzer HL, Wagner ER, Kircher MF, Spinner RJ, Bishop AT, Shin AY, et al. Evaluation of infraspinatus reinnervation and function following spinal accessory nerve to suprascapular nerve transfer in adult traumatic brachial plexus injuries. Microsurgery. 2017, 37: 365–70.

[56] Hu S, Chu B, Song J, Chen L. Anatomic study of the intercostal nerve transfer to the suprascapular nerve and a case report. J Hand Surg Eur. 2014, 39: 194–8.

[57] Kostas-Agnantis I, Korompilias A, Vekris M, Lykissas M, Gkiatas I, Mitsionis G, et al. Shoulder abduction and external rotation restoration with nerve transfer. Injury. 2013, 44: 299–304.

[58] Bhatia A, Doshi P, Koul A, Shah V, Brown JM, Salama M, et al. Contralateral C-7 transfer: is direct repair really superior to grafting? Neurosurg Focus. 2017, 43: E3.

[59] Kita Y, Tajiri Y, Hoshikawa S, Hara Y, Iijima J. Impact of phrenic nerve paralysis on the surgical outcome of intercostal nerve transfer. Hand Surg. 2015, 20: 47–52.

[60] Hou Y, Yang J, Yang Y, Qin B, Fu G, Li X, et al. Flow-through anastomosis using a T-shaped vascular pedicle for gracilis functioning free muscle transplantation in brachial plexus injury. Clinics (Sao Paulo). 2015, 70: 544–9.

Article title: Developing a core outcome set for Traumatic Brachial Plexus Injuries: a systematic review of outcomes

For peer review only - http://bmjopen.bmj.com/site/about/guidelines.xhtml

Supplementary file 4. Included Studies

[61] Sechachalam S, O'Byrne A, MacQuillan A. Free Functional Muscle Transfer Tendon Insertion Secondary Advancement Procedure to Improve Elbow Flexion. Tech Hand Up Extrem Surg. 2017, 21: 8–12.

[62] Chu B, Wang H, Chen L, Gu Y, Hu S. Dual Nerve Transfers for Restoration of Shoulder Function After Brachial Plexus Avulsion Injury. Ann Plast Surg. 2016, 76: 668–73.

[63] Bhat DI, Indira Devi B, Bharti K, Panda R. Cortical plasticity after brachial plexus injury and repair: a resting-state functional MRI study. Neurosurg Focus. 2017, 42: E14.

[64] Bertelli JA, Ghizoni MF, J.A. B. Results of spinal accessory to suprascapular nerve transfer in 110 patients with complete palsy of the brachial plexus. J Neurosurg Spine. 2016, 24: 990–5.

[65] Frueh FS, Ho M, Schiller A, Ducommun P, Manoliu A, Andreisek G, et al. Magnetic Resonance Neurographic and Clinical Long-Term Results After Oberlin's Transfer for Adult Brachial Plexus Injuries. Ann Plast Surg. 2017, 78: 67–72.

[66] Maldonado AA, Kircher MF, Spinner RJ, Bishop AT, Shin AY. Free Functioning Gracilis Muscle Transfer versus Intercostal Nerve Transfer to Musculocutaneous Nerve for Restoration of Elbow Flexion after Traumatic Adult Brachial Pan-Plexus Injury. Plast Reconstr Surg. 2016, 138: 483e-488e.

[67] Bertelli JA, Ghizoni MF, Tacca CP. Results of wrist extension reconstruction in C5-8 brachial plexus palsy by transferring the pronator quadratus motor branch to the extensor carpi radialis brevis muscle. J Neurosurg. 2016, 124: 1442–9.

[68] Nicoson MC, Franco MJ, Tung TH. Donor nerve sources in free functional gracilis muscle transfer for elbow flexion in adult brachial plexus injury. Microsurgery. 2017, 37: 377–82.

[69] Bhandari PS, Deb P. Use of contralateral spinal accessory nerve for ipsilateral suprascapular neurotization in global brachial plexus injury: A new technique. J Neurosurg Spine. 2016, 24: 186–8.

[70] Maricq C, Jeunehomme M, Mouraux D, Remy P, Brassinne E, Bahm J, et al. Objective evaluation of elbow flexion strength and fatigability after nerve transfer in adult traumatic upper brachial plexus injuries. Hand Surg. 2014, 19: 335–41.

[71] DeGeorge B, Becker H, Faryna J, Spinner R, Bishop A. Outcomes of brachialis muscle transfer to restore finger flexion in traumatic lower trunk brachial plexus palsy. J Hand Surg Am. 2017, 42: S33–4.

[72] Liu Y, Lao J, Gao K, Gu Y, Zhao X. Functional outcome of nerve transfers for traumatic global brachial plexus avulsion. Injury. 2013, 44:655–60.

[73] Bertelli JA, Ghizoni MF. Transfer of a flexor digitorum superficialis motor branch for wrist extension reconstruction in C5-C8 root injuries of the brachial plexus: a case series. Microsurgery. 2013, 33:39–42.

[74] Gao K, Lao J, Zhao X, Gu Y. Outcome after transfer of intercostal nerves to the nerve of triceps long head in 25 adult patients with total brachial plexus root avulsion injury. J Neurosurg 2013, 118: 606–10.

[75] Foroni L, Siqueira MG, Martins RS, Heise CO, Sterman HN, Imamura AY. Good sensory recovery of the hand in brachial plexus surgery using the intercostobrachial nerve as the donor. Arq Neuropsiquiatr. 2017, 75: 796–800.

[76] Socolovsky M, di Masi G, Bonilla G, Dominguez Paez M, Robla J, Calvache Cabrera C, et al. The

Article title: Developing a core outcome set for Traumatic Brachial Plexus Injuries: a systematic review of outcomes

For peer review only - http://bmjopen.bmj.com/site/about/guidelines.xhtml

Supplementary file 4. Included Studies

phrenic nerve as a donor for brachial plexus injuries: is it safe and effective? Case series and literature analysis. Acta Neurochir (Wien). 2015, 157: 1077–86.

[77] Hattori Y, Doi K, Sakamoto S, Satbhai NG. Complete avulsion of brachial plexus with associated vascular trauma: feasibility of reconstruction using the double free muscle technique. Plast Reconstr Surg. 2013, 132: 1504–12.

[78] Kachramanoglou C, Carlstedt T, Koltzenburg M, Choi D, C. K, T. C, et al. Long-Term Outcome of Brachial Plexus Reimplantation After Complete Brachial Plexus Avulsion Injury. World Neurosurg. 2017, 103: 28–36.

[79] Kodama N, Doi K, Hattori Y. Force recovery assessment of functioning free muscle transfers using ultrasonography. J Hand Surg Am. 2014, 39: 2269–76.

[80] Goubier J, Teboul F. Rhomboid nerve transfer to the suprascapular nerve for shoulder reanimation in brachial plexus palsy: A clinical report. Hand Surg Rehabil. 2016, 35: 363–6.

[81] Gao K, Lao J, Zhao X, Gu Y. Outcome of contralateral C7 transfer to two recipient nerves in 22 patients with the total brachial plexus avulsion injury. Microsurgery. 2013, 33: 605–11.

[82] Liu Y, Lao J, Zhao X. Comparative study of phrenic and intercostal nerve transfers for elbow flexion after global brachial plexus injury. Injury. 2015, 46: 671–5.

[83] Li XM, Yang JT, Hou Y, Yang Y, Qin BG, Fu G, et al. Donor-side morbidity after contralateral C-7 nerve transfer: results at a minimum of 6 months after surgery. J Neurosurg. 2016, 124: 1434–41

[84] Rasulic L, Savic A, Zivkovic B, Vitosevic F, Micovic M, Bascarevic V, et al. Outcome after brachial plexus injury surgery and impact on quality of life. Acta Neurochir (Wien) 2017, 159: 1257–64

[85] Yang J, Jia X, Yu C, Gu Y. Pronator teres branch transfer to the anterior interosseous nerve for treating C8T1 brachial plexus avulsion: An anatomic study and case report. Neurosurgery. 2014, 75: 375–9.

[86] Stiasny J, Birkeland P, J. S. Operative treatment with nerve repair can restore function in patients with traction injuries in the brachial plexus. Dan Med J. 2015, 62.

[87] Soldado F, Ghizoni MF, Bertelli J, F. S, M.F. G. Thoracodorsal nerve transfer for triceps reinnervation in partial brachial plexus injuries. Microsurgery. 2016, 36: 191–7.

[88] Thakkar UG, Vanikar A V, Trivedi HL. Co-infusion of autologous adipose tissue derived neuronal differentiated mesenchymal stem cells and bone marrow derived hematopoietic stem cells, a viable therapy for post-traumatic brachial plexus injury: a case report. Biomed J. 2014, 37: 237–40.

[89] Emamhadi M, Alijani B, Andalib S, M. E, B. A. Long-term clinical outcomes of spinal accessory nerve transfer to the suprascapular nerve in patients with brachial plexus palsy. Acta Neurochir (Wien). 2016, 158: 1801–6.

[90] Tu YK, Tsai YJ, Chang CH, Su FC, Hsiao CK, Tan JS. Surgical treatment for total root avulsion type brachial plexus injuries by neurotization: a prospective comparison study between total and hemicontralateral C7 nerve root transfer. Microsurgery. 2014, 34: 91–101.

[91] Qiu YQ, Hua XY, Zuo CT, Li T, Zheng MX, Shen YD, et al. Deactivation of distant pain-related regions induced by 20-day rTMS: a case study of one-week pain relief for long-term intractable deafferentation pain. Pain Physician. 2014, 17: E99–105.

Article title: Developing a core outcome set for Traumatic Brachial Plexus Injuries: a systematic review of outcomes

For peer review only - http://bmjopen.bmj.com/site/about/guidelines.xhtml

Supplementary file 4. Included Studies

[92] Haninec P, Mencl L, Kaiser R. End-to-side neurorrhaphy in brachial plexus reconstruction. J Neurosurg. 2013, 119: 689–94.

[93] Flores LP. Reanimation of elbow extension with medial pectoral nerve transfer in partial injuries to the brachial plexus. J Neurosurg. 2013, 118: 588–93.

[94] Mohammad-Reda A. Early post-operative results after repair of traumatic brachial plexus palsy. Turk Neurosurg. 2013, 23: 1–9.

[95] van der Lingen MAJ, de Joode SGCJ, Schotanus MGM, Grimm B, van Nie FA, Speth LAWM, et al. Satisfied patients after shoulder arthrodesis for brachial plexus lesions even after 20 years of follow-up. Eur J Orthop Surg Traumatol. 2018, 28: 1089–94

[96] Klika BJ, Spinner RJ, Bishop AT, Kircher MF, Shin AY. Posterior branch of the axillary nerve transfer to the lateral triceps branch for restoration of elbow extension: case report. J Hand Surg Am. 2013, 38: 1145–9.

[97] Liu J, Wang X, Zhang S, Wang L, Xia D, Gao Q, et al. A clinical analysis of repairing the whole brachial plexus nerve root avulsion by transferring C7 nerve root from the uninjured side. J Neurol Sci. 2014, 31: 521–31.

[98] Cambon-Binder A, Walch A, Marchei P, Belkheyar Z. Bipolar transfer of the pectoralis major muscle for restoration of elbow flexion in 29 cases. J Shoulder Elb Surg. 2018, 27: e330-e336

[99] Soldado F, Ghizoni MF, Bertelli J. Thoracodorsal nerve transfer for elbow flexion reconstruction in infraclavicular brachial plexus injuries. J Hand Surg Am. 2014, 39: 1766–70.

[100] Cho A, Paulos R, De Resende M, Kiyohara L, Sorrenti L, Wei TH, et al. Median nerve fascicle transfer versus ulnar nerve fascicle transfer to the biceps motor branch in C5-C6 and C5-C7 brachial plexus injuries: Nonrandomized prospective study of 23 consecutive patients. Microsurgery. 2014, 34: 511–5.

[101] Kaizawa Y, Kakinoki R, Ohta S, Noguchi T. Free functional muscle transplantation of an anomalous femoral adductor with a very large muscle belly: A case report. J Brachial Plex Peripher Nerve Inj. 2013, 8: 11.

[102] Tuohuti T, Yu Q, Yang J, Wang T. Selective neurotization of the radial nerve in the axilla using intercostal nerve to treat complete brachial plexus palsy. Int J Clin Exp Med. 2016, 9: 22880–5.

[103] Flores LP. Objective Predictors of Functional Recovery Associated with Intercostal Nerves Transfer for Triceps Reinnervation in Global Brachial Plexus Palsy. Brazilian Neurosurg 2016, 35: 271–8.

[104] Emamhadi M. Nerve transfer to relieve pain in upper brachial plexus injuries: Does it work? Clin Neurol Neurosurg. 2017,163: 67–70.

[105] Abdixbir A, Li P, Ilhamjan U, Exmetjan Y. Phrenic nerve transfer versus intercostal nerve transfer for the repair of brachial plexus root avulsion injuries. Chinese J Tissue Eng Res. 2016, 20: 7660–5.

[106] Limthongthang R, Vathana T, Wongtrakul S, Songcharoen P. End-to-Side Neurorrhaphy to Restore Elbow Flexion in Brachial Plexus Injury. J Med Assoc Thai. 2016, 99: 1203–8.

[107] De Oliveira C, Da Silva Melo D, Mestriner R, De Menezes M, Da Silva Filho I, Da Silva J. Chordata method combined with electrotherapy in functional recovery after brachial plexus injury: Report of three clinical cases. Sci Med (Porto Alegre). 2016, 26: 22425.

Article title: Developing a core outcome set for Traumatic Brachial Plexus Injuries: a systematic review of outcomes

For peer review only - http://bmjopen.bmj.com/site/about/guidelines.xhtml

Supplementary file 4. Included Studies

[108] Xu B, Dong Z, Zhang C-G, Gu Y-D. Clinical outcome following transfer of the supinator motor branch to the posterior interosseous nerve in patients with C7-T1 brachial plexus palsy. J Reconstr Microsurg. 2015, 31: 102–6.

[109] Wu X, Cong XB, Huang QS, Ai FX, Liu YT, Lu XC, et al. Transposition of branches of radial nerve innervating supinator to posterior interosseous nerve for functional reconstruction of finger and thumb extension in 4 patients with middle and lower trunk root avulsion injuries of brachial plexus. J Huazhong Univ Sci Technolog Med Sci. 2017, 37: 933–7.

[110] Kazamel M, Sorenson EJ. Electromyographic Findings in Gracilis Muscle Grafts Used to Augment Elbow Flexion in Traumatic Brachial Plexopathy. J Clin Neurophysiol. 2016. 33:549–53.

[111] Li Z, Reynolds M, Satteson E, Nazir O, Petit J, Smith BP. Double Distal Intraneural Fascicular Nerve Transfers for Lower Brachial Plexus Injuries. J Hand Surg Am. 2016, 41: e15-9.

[112] Arnal M, Cambon A, Marcheix P. Restoration of elbow and hand function in total brachial plexus palsy with intercostal nerves and C5 root neurotization. Results in 21 patients. Hand Surg Rehabil. 2016, 35: 283–7.

[113] Jiang Y, Lao J. The phrenic nerve transfer in the treatment of a septuagenarian with brachial plexus avulsion injury: a case report. Int J Neurosci. 2018, 128: 467–71.

[114] Flores LP. Outcomes of Transferring a Healthy Motor Fascicle From the Radial Nerve to a Branch for the Triceps to Recover Elbow Extension in Partial Brachial Plexus Palsy. Neurosurgery. 2017, 80: 448–53.

[115] Johnsen PH, Wolfe SW. Successful Nerve Transfers for Traumatic Brachial Plexus Palsy in a Septuagenarian: A Case Report. Hand (N Y). 2016, 11: NP30–3.

[116] Maldonado AA, Romero-Brufau S, Kircher MF, Spinner RJ, Bishop AT, Shin AY. Free Functioning Gracilis Muscle Transfer for Elbow Flexion Reconstruction after Traumatic Adult Brachial Pan-Plexus Injury: Where Is the Optimal Distal Tendon Attachment for Elbow Flexion?. Plast Reconstr Surg. 2017, 139: 128–36.

[117] Bhandari PS. Results of Distal Nerve Transfers in Restoration of Shoulder Function in C5 and C6 Root Avulsion Injury to the Brachial Plexus. Indian J Neurotrauma 2017, 14: 21–5.

[118] Watanabe M, Yamamoto T, Fukaya C, Obuchi T, Kano T, Kobayashi K, et al. Bipolar dual-lead spinal cord stimulation between two electrodes on the ventral and dorsal sides of the spinal cord: consideration of putative mechanisms. Acta Neurochir (Wien). 2018, 160: 639–43.

[119] Al-Qattan M, Kattan A, Al-Qahtany B, Al-Qattan O. Triceps nerve to deltoid nerve transfer after an unsatisfactory intra-plexus neurotization of the posterior division of the upper trunk. Int J Surg Case Rep. 2017, 37: 124–6.

[120] Alrabai H, Gesheff G, Hammouda A, Conway J. Trapezius Muscle Transfer for Restoration of Elbow Extension in a Traumatic Brachial Plexus Injury. J Hand Surg Am. 2018, 43: 872.

[121] Bertelli JA. Transfer of the radial nerve branch to the extensor carpi radialis brevis to the anterior interosseous nerve to reconstruct thumb and finger flexion. J Hand Surg Am. 2015, 40: 323-328.

[122] Magistroni E, Ciclamini D, Panero B. Ultrasound-guided pulse-dose radiofrequency: Treatment of neuropathic pain after brachial plexus lesion and arm revascularization. Case Rep Med. 2014, 201:429618.

Article title: Developing a core outcome set for Traumatic Brachial Plexus Injuries: a systematic review of outcomes

For peer review only - http://bmjopen.bmj.com/site/about/guidelines.xhtml

Supplementary file 4. Included Studies

[123] Liu Y, Xu X, Zou Y, Li S, Zhang B. Phrenic nerve transfer to the musculocutaneous nerve for the repair of brachial plexus injury: Electrophysiological characteristics. Neural Regen Res. 2015, 10: 328–33.

[124] Le Hanneur M, Walch A, Gerosa T, Grandjean A, Masmejean E. Postoperative motor deficits following elbow flexion reanimation by nerve transfer. Hand Surg Rehabil 2018, 37: 289-294

[125] Liu Y, Zhuang Y, Yu H, Xiong H. Comparative study of phrenic and partial ulnar nerve transfers for elbow flexion after upper brachial plexus avulsion: A retrospective clinical analysis. J Plast Reconstr Aesthetic Surg. 2018, 71: 1245–51.

[126] Yavari M, Mahmoudvand H, Nadri S. Contralateral medial pectoral nerve transfer with free gracilis muscle transfer in old brachial plexus palsy. J Surg Res. 2018, 231: 94–8.

[127] Yanagisawa T, Fukuma R, Seymour B, Hosomi K, Kishima H, Shimizu T, et al. MEG-BMI to control phantom limb pain. Neurol Med Chir (Tokyo). 2018, 58: 327–33.

[128] Choong C, Shalimar A. Complete brachial plexus injury - An amputation dilemma. A case report. Malaysian Orthop J. 2015, 9: 52–4.

[129] Tsao J, Finn S. Reversal of phantom pain and hand-to-face remapping after brachial plexus avulsion. Ann Clin Transl Neurol. 2016, 3: 463–4..

[130] Kubota S, Hara Y, Shimizu Y, Kadone H, Kubo T, Marushima A, et al. A newly developed upper limb single-joint HAL in a patient with elbow flexion reconstruction after traumatic brachial plexus injury: A case report. Interdiscip Neurosurg Adv Tech Case Manag. 2017, 10: 66–8.

[131] Bertelli JA. Free Reverse Gracilis Muscle Combined With Steindler Flexorplasty for Elbow Flexion Reconstruction After Failed Primary Repair of Extended Upper-Type Paralysis of the Brachial Plexus. J Hand Surg Am. 2019, 44: 112-120

[132] Xu B, Dong Z, Zhang C. Multiple nerve and tendon transfers: A new strategy for restoring hand function in a patient with C7-T1 brachial plexus avulsions. J Neurosurg. 2017, 127: 837–42.

For peer review only

For peer review only - http://bmjopen.bmj.com/site/about/guidelines.xhtml

Supplementary file 5: Unique outcomes mapped to potential domains and core areas according to COMET

Online Supplementary file 5. Table: Unique outcomes mapped to potential domains and core areas according to COMET(Dodd et al., 2018)

Outcomes (n=157)	Subdomains	Domains	Core Areas
Isometric muscle strength	Muscle strength/ function	Musculoskeletal and connective tissue domain	Physiological/Clinical
Concentric strength			
Eccentric strength			
Muscle flicker/contraction			
Anti-gravity muscle activity			
Muscle endurance			
Muscle fatigue			
Muscle torque			
Active range of movement	Active movement		
Perception of movement			
Antigravity movement			
Independent movement without donor			
Passive range of movement	Passive movement		
Movement control/stability	Control of movement/stability		
Muscle mass	Muscle mass		
Bony union	Bone structure/position		
Joint position			
Joint stability			
General sensory recovery	General sensory recovery	Nervous system	
Feeling of numbness			
Proprioception			
Light touch	Discriminative touch		
2 PD			

Article title: Developing a core outcome set for Traumatic Brachial Plexus Injuries: a systematic review of outcomes

For peer review only

For peer review only - http://bmjopen.bmj.com/site/about/guidelines.xhtml

Supplementary file 5: Unique outcomes mapped to potential domains and core areas according to COMET

Vibration			
Object recognition			
Pain	Protective touch		
Temperature			
Deep pressure			
Brachial plexus structure	Peripheral nervous system structure		
Level of reinnervation	Reinnervation		
Time to reinnervation			
Progression of regeneration	Progression of regeneration		
Speed of motor sensory conduction	Speed of motor and sensory conduction		
Pain intensity	Pain intensity/relief	General outcomes/symptoms	
Pain relief / reduction			
Pain duration	Pain duration/frequency		
Pain frequency			
Pain quality	Pain quality and interference with life		
Pain interference with walking			
Pain interference in mood			
Pain interference with work			
Pain interference in activities of daily living			
Pain interference with relationships			
Pain interference with enjoyment of life			
Pain interference with sleep			
Sensitivity to cold	Pain when arm exposed to cold		
Paraesthesia	Paraesthesia and itchiness		
Itchiness			

Article title: Developing a core outcome set for Traumatic Brachial Plexus Injuries: a systematic review of outcomes

For peer review only - http://bmjopen.bmj.com/site/about/guidelines.xhtml

For peer review only

Supplementary file 5: Unique outcomes mapped to potential domains and core areas according to COMET

Sensitivity to pressure	Sensitivity to touch, pressure etc		
Sensitivity to touch			
Pain location	Location of pain		
Pain relief from medication	Pain medication use		
Stiffness	Stiffness		
Impact on general sleep	Impact on sleep		
Impact on sleep on affected side			
Frequency sleep disturbed by injury			
General physical function	Physical function non-specific	Physical functioning	Life Impact
Patient led functional outcome			
Walking short distance	Lower limb and non -upper limb function		
Balance			
Running			
Climbing stairs			
Bending			
Kneeling			
Reaching	Reaching, pulling, pushing, carrying etc		
Pulling			
Pushing			
Carrying			
Throwing			
Lifting			
General function of arm			
Turning and twisting arm	Turning twisting, gripping and releasing with the arm		
Grip and release			
Pinching	Fine hand movement including writing		
Fine hand movement (writing/buttons)			
Returning to work	Impact on paid or unpaid work or role in education	Role functioning	
Ability to do work			
Usual time at work			
Type of work			

Article title: Developing a core outcome set for Traumatic Brachial Plexus Injuries: a systematic review of outcomes

For peer review only - http://bmjopen.bmj.com/site/about/guidelines.xhtml

For peer review only

Supplementary file 5: Unique outcomes mapped to potential domains and core areas according to COMET

Usual school activities			
General rating to perform a patient specific activity	Role function - patient specific		
Impact on ADL (general)	Carrying out daily routine, (including food preparation, housework, garden, plants)		
Return to ADL (general)			
Impact on food preparation and feeding			
Housework (washing, cleaning, ironing, folding, vacuuming)			
Gardening (Includes indoor plants)			
Using a phone			
Maintaining personal hygiene			
Maintaining personal appearance (grooming hair)	Maintaining personal hygiene		
Dressing	Maintaining personal appearance		
Transport needs (e.g driving)	Dressing		
Impact on normal hobbies	Transport needs		
Time doing normal hobbies	Impact on recreational activities and sport		
Playing instrument in usual way			
Ability to play instrument			
Impact on time spent playing instrument			
Impact on time spent doing sport			
Impact on participation in sport			
Social activities with friends	Effect on relationship with	Social functioning	

For peer review only

For peer review only - http://bmjopen.bmj.com/site/about/guidelines.xhtml

Supplementary file 5: Unique outcomes mapped to potential domains and core areas according to COMET

Social activities with neighbours	family, friends, neighbours and groups		
Social activities with family			
Social activities with groups			
Dependence on family and friends			
Appearance interferes with social activities			
Intimate relationships	Effect on intimate relationships		
Emotional impact on work	Emotional distress/mood	Emotional functioning	
Energy levels			
Emotional impact on ADL			
Happiness			
Impact on life enjoyment / satisfaction			
Emotional impact on relationships			
Anxiety			
Depression			
Acceptance/ Adjustment	Thoughts and beliefs (acceptance, coping)		
Coping with trauma			
Confidence	Self esteem and confidence		
Self esteem			
Body image	Body image		
Quality of life	Quality of Life	Global Quality of Life	Quality of Life
Rating of health	Perceived Health status	Health status	Health status
General patient satisfaction	Patient satisfaction	Delivery of Care	Delivery of Care
Satisfaction with appearance of arm			
Satisfaction with function			
Satisfaction with movement			
Satisfaction with strength			

For peer review only

For peer review only - http://bmjopen.bmj.com/site/about/guidelines.xhtml

Supplementary file 5: Unique outcomes mapped to potential domains and core areas according to COMET

Satisfaction with pain			
Satisfaction with colour			
Satisfaction with shape			
Satisfaction with feeling			
Satisfaction with procedure			
Patient preference	Patient preference		
Quality of intervention	Accessibility, quality and adequacy of intervention		
Time to surgery	Time to surgery		
Operation time	Operation time	Resource Use	Resource Use
Motor morbidity	Donor site morbidity	Adverse Events	Adverse Events
Sensory morbidity			
Pain			
General complications	General complications		
Pneumothorax	Respiratory complications		
Respiratory function			
Respiratory symptoms			
Pneumonia			
Arterial thrombosis	Vascular complications		
Venous thrombosis			
Haematoma			
Venous spasm			
Iatrogenic vascular injury			
Vascularity of flap			
Swelling			
Fracture	Musculoskeletal complications		
Passive range of motion loss			
Co-contraction			
Bowstringing			
Failure of tendon attachment			
Joint Instability			
Scapula crepitus			

Article title: Developing a core outcome set for Traumatic Brachial Plexus Injuries: a systematic review of outcomes

For peer review only - http://bmjopen.bmj.com/site/about/guidelines.xhtml

For peer review only

Supplementary file 5: Unique outcomes mapped to potential domains and core areas according to COMET

Infection complications	Infection complications		

Dodd, S. et al. A taxonomy has been developed for outcomes in medical research to help improve knowledge discovery. Journal of clinical epidemiology. 2018, 96: 84–92.

For peer review only

For peer review only - http://bmjopen.bmj.com/site/about/guidelines.xhtml

Supplementary file 6. Measurement of outcomes and measurement tools used

Supplementary file 6. Measurement of outcomes and measurement tools used

56 outcome subdomains in 4 core areas (Physiological/clinical, Life Impact, Resource Use and Adverse events) and within the following COMET domains

Musculoskeletal/connective tissue, Nervous system outcome domain, General outcome and symptom domain, Physical functioning, Role functioning, Emotional functioning, Global quality of life, Perceived health status, Delivery of care, Hospital resources and Adverse Events

**Core Area**	Outcome subdomains	**Measurement type used (N)**				**Measurement instruments used (number of studies)**
**PHYSIOLOGICAL /CLINICAL**		**Patient reported Outcome**	**Clinician reported Outcome**	**Perfomance Outcome**	**Not Clear**	
	**Musculoskeletal/connective tissue**					
	Muscle strength	30	129	19	3	DASH (n= 27), UEFI (n=2), MHQ (n=1),<br><br>**Manual Muscle Testing**<br>**Manual muscle testing undefined (n=5)**<br>**MRC muscle grading (n=61 , including UCLA)**<br>**MRC muscle grading modified (n= 22),**<br>*MRC modified*, unclear how (n= 5)<br>*MRC modified,* grade 3 active must equal passive (n=2)<br>*MRC modified* , grade 2 active must equal passive movement (n=2)<br>*MRC modified,* M3+ contraction with resistance against a finger for less than 30 seconds, M4 contraction of resistance against a finger against a finger for more than 30 seconds (n=1)<br>*MRC modified*: M0, M1+, M1, M1+, M2-, M2, M2+,M3-, M3, M3+, M4-, M4, M4+, M5-, M5 (n=6)<br>*MRC modified,* Finger flexion tested with wrist extended 20-30 degrees (n=1)

For peer review

Supplementary file 6. Measurement of outcomes and measurement tools used

						MRC modified, Addition of M4.5 (n=1)<br>*MRC modified,* graded two muscles together (n=1)<br>*MRC modified,* finger extension tested with wrist extension at 20-30 degrees (n=1)<br>*MRC modified,* summated muscle score (n=1)<br>*MRC modified,* FDS tested by stabilising LF and IF to table and testing MF and RF IP flexion (n=1)<br>**Other manual muscle tests (n=3)**<br>Kendall and McCreary testing procedure (n=1)<br>Oxford muscle testing (n=1)<br>Modification of the Louisiana State University Medical Centre grading system (n=1)<br>**Time to (n= 12)**<br>contraction (n=7); M2 (n=1); strength greater than or equal to M3 (n=1); M 3 (n=1); greater than or equal to modified M3 (n=1); Time to improvement in MRC scale (n= 1)<br>**Dynanometry (n=23)**<br>Dynanometry – isokinetic machine, undefined method (n =1)<br>Grip strength JAMAR , undefined method (n=4); Hook grip – isokinetic machine, undefined method (n=1); Grip strength JAMAR, mean of 3 trials n=2); Grip strength , PABLO system, undefined (n=1); Pinch grip, JAMAR, undefined (n= 3), Pinch grip JAMAR, mean 3 trials (n= 1); Peak isometric, hand held dynamometer (n=2); Isometric strength , hand held dynamometer, best of 3 trials (n=1); Isometric strength , Kendall & Kendall positions, 3 trials mean value (n=1); Measurement on digital scales after 5 seconds (n=1)<br>Concentric strength through range, Isokinetics (n=1)<br>Eccentric strength through range, isokinetics (n=1)<br>Combined action of using elbow and hand on digital hanging scale (n=1)<br>Constant-Murley score: dynanometry 90 degrees abduction(n=2)

For peer review only

Supplementary file 6. Measurement of outcomes and measurement tools used

						**Narakas score modified (one study)**<br>Thoaraco brachial grasp (n=1)<br>Elbow flexion with weight (n=1)<br>Elbow extension with weight (n=1)<br>Wrist flexion with weight (n=1)<br>Wrist extension with weight(n=1)<br>Fist power with weight (n=1)<br>Pinch power (n=1)<br><br>**ULM (one study)**<br>Shoulder flexion to shoulder height with 500g (n=1)<br>Shoulder flexion above shoulder height with 500g (n=1)<br>Shoulder flexion above shoulder with 1kg (n=1)<br>Move weight on table (100g) (n=1)<br>Move weight on table (500g) (n=1)<br>Move weight on table (1KG) (n=1)<br><br>**SHAP (one study)**<br>Grip strength (n=1)<br>Pinch strength (n=1)<br>Pinch grip (lateral) (n=1)<br>Pinch grip (tip) (n=1)<br>Grip strength (power) (n=1)<br>Heavy extension (n=1)<br><br>Ability to lift weight, undefined (n=1)<br>Number of repetitions movement can be performed in 10 seconds (n=1)<br>Maximum weight sustained when flexing elbow (n=1)<br>**Unclear (n= 3)**

For peer review only

Supplementary file 6. Measurement of outcomes and measurement tools used

						Force recovery: Cross sectional area of the muscle under isometric contraction divided by cross sectional area at rest (n=1)
	Active movement	5	103	3	63	SST(n=1), MHQ (n=1), UCLA shoulder rating scale (n=1), MPI(n=2), CONSTANT- MURLEY(n=2) (2xPRO, 8x ClinRO), ARAT (PerfO, n=1), ULM (PerfO, n=2), Goniometry(n=48), Visual assessment (n=32), First web space in cm (n=3), Total active movement(n=2), Pulp to palm distance (n=2)<br>Months to full active movement (n=1)<br>Months to antigravity movement (n=3)<br>Months to initial movement (n= 1)<br>Months to independent movement without donor (n=1)<br>Not clear (n=63)
	Passive range of movement		6		7	Not defined (n=7), Goniometry(n=6)
	Movement control and stability		1	1	2	MPI (ClinRo, n=1), ULM (PerfO, n=1), Not clear (n=2)
	Bone structure/position/healing				4	Not clear (n=4)
	Muscle mass				4	Not clear(n=4)
	**Nervous system outcome subdomains**					
	General sensory recovery including proprioception		**9**		**8**	Sensory BMRC (n=5), Modified Sensory BMRC (n= 2), Highet classification(n=2), Not clear (n=8)
	Discriminative touch (light touch, two point discrimination, vibration, object recognition)	**1**	**14**			MHQ (n=1), Cotton wool (n=3), Semmes Weinstein Monofilaments (n=4), Two point discrimination(n=2), Tuning fork (n=4), Not defined (ClinRo, n=1)
	Protective touch (pain, temperature, deep pressure)		**3**		**7**	Blunt pin (n=3), Not clear (n=7)
	Structure of peripheral nervous system		1			MRI (n=1)
	Reinnervation (level of reinnervation, time to innervation)		54			Two point scale on EMG(n=1) Four point scale on EMG (n=4), Not clear EMG (n= 49)
	Progression of regeneration		5			Tinel sign (n=5)
	Speed of motor and sensory conduction		9			EMG (n=9)
	**General outcomes / symptoms**					

Supplementary file 6. Measurement of outcomes and measurement tools used

	Pain intensity/ relief	73			3	DASH (n=27), ASES (n=1), TAPES (n=1), VAS(n=18), NRS(n=12),MHQ (n=1) WBFRS(n=1), BPI (n= 1), UNWNS (n=1), McGill Pain Questionnaire SF (n=2), McGill pain questionnaire (n= 1), MPI (n=1), CONSTANT-MURLEY (n=2), 4 point scale (n=3) Author developed questionnaire(n=1), Not Clear (n=3)
	Pain duration or frequency	12	0	0	0	SST (n=1), SF36 (n=5), MHQ (n=1),TAPES(n=1), NPSI (n=1), BPI (n= 1), UCLA shoulder rating score (n=1), Not described PRO (n=1)
	Pain quality	7				TAPES (n= 1), NPSI(n=1), UWNS(n= 1), McGill SF(n=2), McGill (n=1), Non described PRO (n =1)
	Pain when arm exposed to cold	1				NPSI (n=1)
	Paraesthesia	27				DASH (n=27)
	Sensitivity to touch, pressure, vibration etc	3				NPSI (n=1) UWNS (n= 1), NRS (n=1)
	Location of pain	1				BPI (n=1)
	Pain medication use	1				BPI(n=1)
	Stiffness	27				DASH (n=27)
LIFE IMPACT	**Physical functioning**					
	Physical function non-specific	2				PSFS (n=1), TAPES (n=1)
	Lower limb and non-upper limb function (walking, running, climbing stairs etc)	7			1	SF36 (n=5), TAPES (n= 1), BPI (n=1) Non described PRO (n=1)
	Reaching, pulling, pushing, carrying, throwing , lifting	37		3		DASH (n=27), UEFI (n=2), MHQ(n=1), ASES(n=1), SST (n=1), SF36(n=5), ARAT(n=1), AMULA (n=1) UNBtP (n=1)
	Turning twisting, gripping and releasing with the arm	30		5	1	DASH (n=27), UEFI (n=2), MHQ (n=1),ARAT(n=1),SHAP(n= 1), JHFT (n=1), AMULA (n=1), UNBtP (n=1), Not clear (n=1)
	Fine hand movement include writing	30		6		DASH (n=27), UEFI (n=2), MHQ (n=1),ARAT(n=1), SHAP(n=1), JHFT (n=1) Purdue Peg test (n=1),AMULA (n=1), UNBtP (n=1)
	**Role Functioning**					
	Impact on return to work	41				DASH (n =27), UEFI (n=2),MHQ (n=1), ASES (n=1), SST (n=1), SF36 (n=5), TAPES (n=1), MPI (n=1) No description PRO (n=1), Questionnaire no data (n=1)
	Role function patient specific	1				PSFS(n=1)

For peer review only

Supplementary file 6. Measurement of outcomes and measurement tools used

	Carrying out daily routine, (including food preparation, housework, garden, plants)	36	1	5		DASH (n=27), UEFI (n=2), MHQ (n=1),TAPES(n=1) , BPI (n=1), UCLA (n=1), SHAP (n=1), Jebsen (n=1), ULM (n=1) Questionnaire not defined (n=2),No description PRO (n=1) Unclear CLinRO(n=1), AMULA (n=1), UNBtP (n=1)
	Maintaining personal hygiene	35		2		DASH (n=27), ASES (n=1), SST(n=1), SF36(n=5), MHQ(n=1) AMULA (n=1), UNBtP (n=1)
	Maintaining personal appearance	3		1		UEFI (n=2), ASES (n= 1), AMULA (n=1)
	Dressing	32		2		DASH (n =27), UEFI (n=2), MHQ (n=1),ASES (n= 1), SST (n=1), AMULA (n=1) SHAP(n=1)
	Transport needs	29				DASH (n =27), UEFI (n=2),
	Impact on recreational activities and sport	34				DASH (n =27), UEFI (n=2), ASES (n= 1), TAPES(n=1), CONSTANT-MURLEY (n=2),Not described PRO (n=1)
	**Social functioning**					
	Effect on relationship with family, friends, neighbours and groups	34				DASH (n =27), SF36 (n=5), TAPES (n=1), MHQ (n=1)
	Effect on intimate relationships	27				DASH (n =27)
	**Emotional Functioning**					
	Emotional distress/ mood	11				SF36 (n=5), TAPES (n= 1),BPI(n=1), UWNS(n=1), Self-rated anxiety scale (n=1), Self-rated depression scale (n=1), MHQ (n=1)
	Thoughts and beliefs (acceptance and adjustment)	1				TAPES (n=1)
	Self-esteem and self confidence	28				DASH (n=27), TAPES(n= 1)
	Body image	3				MHQ (n= 2), Not described (n=1)
	**Sleep and overall health**					
	Impact on sleep	37				DASH (n=27), UEFI (n=3), ASES(n= 1), MHQ (n=1), SST (n=1), BPI(n=1), CONSTANT- MURLEY(n=2),Not described PRO (n=1)
	**General Quality of life**	1				Not described PRO (n=1)
	**Perceived Health Status**	6				SF36 (n=5), TAPES (n=1)
	**Delivery of Care**					
	Patient satisfaction	10				TAPES (n=1), UCLA (n=1), MHQ (n=1),10-point scale (n=1)

For peer review only

Supplementary file 6. Measurement of outcomes and measurement tools used

						4 point scale (n=2), 3 point likert scale (n=1), Questionnaire not described (n=1),Not defined PRO(n=2)
	Patient preference for treatment	1				Not described (n=1)
	Accessibility, quality and adequacy of intervention				1	4 point scale (n=1)
RESOURCE	**Hospital**					
	Operation time				1	Not described (n=1)
	**Adverse Events**					
ADVERSE	Donor site motor morbidity to include weakness		18		19	BMRC (n=7), BMRC modified(n=2), Dynanometry (n=8), EMG(n=1)<br>Not clear (n=19)
	Donor site sensory morbidity	1	3		4	10-point scale PRO (n=1)<br>Not defined (n=4),2PD (n=2), Monofilaments (n=1)
	Donor site morbidity -pain	3				Not defined PRO (n=3)

For peer review only

Supplementary file 6. Measurement of outcomes and measurement tools used

	General complications				2	Unclear (n=2)
	Respiratory complications	1	5		4	4 point scale PRO (n=1), x-ray (n=2), FEV (n=1), TLC(n=1), MVV (n-1), Not defined (n=4),
	Vascular complications		2		13	Not defined (n=13), Visual assessment (n=1), USS (n=1)
	Musculoskeletal complications		2		19	Not defined CLinRO(n=2), Unclear (n=19)
	Infection complications		1		2	Not defined ClinRo(n=1), Unclear (n=2)
		669	366	46	168	

DASH Disabilities of the arm shoulder and hand, *UEFI* Upper Extremity Functional Index, *MHQ* Michigan Hand Questionnaire, *BMRC* British Medical Research Council, *ULM* Upper Limb Module, *SHAP* Southampton Hand Assessment Procedure, *SST* Simple Shoulder Test, *MPI* Mayo clinic Performance Index for the elbow, *ARAT* Action Research Arm Test, *ClinRO* Clinician Reported Outcome, *PerfO* Performance Outcome, *PRO* Patient Reported Outcome, *ASES* American Shoulder and Elbow Surgeons Index, *TAPES* The Trinity Amputation and Prosthesis Experience Scales, *VAS* Visual Analogue Scale, *NRS* Numerical Rating Scale, *WBFRS* Wong Baker Faces Rating Scale, *UNWNS* University of Washington Neuropathic pain Score, *SF36* Short Form 36 health survey, *NPSI* Neuropathic Pain Symptom Inventory, *BPI* Brief Pain Inventory, *PSFS* Pain Specific Functional Scale, *AMULA* American Measures for Upper Limb Amputees, *UNBPT* University of New Brunswick test of Prosthetics function, *JHFT* Jebsen Hand Function Test, *FEV* Forced Expiratory Volume, *TLC* Tidal Lung Capacity, *MVV* maximal voluntary ventilation, *USS* Ultrasound Scan.

For peer review only

BMJ Open

Developing a core outcome set for traumatic brachial plexus injuries: a systematic review of outcomes.

Journal:	*BMJ Open*
Manuscript ID	bmjopen-2020-044797.R2
Article Type:	Original research
Date Submitted by the Author:	08-Jun-2021
Complete List of Authors:	Miller, Caroline; University of East Anglia, School of Health Sciences, The Queens Building ; Queen Elizabeth Hospital Birmingham, Physiotherapy Department<br>cross, jane; University of East anglia, school AHP<br>O'Sullivan, Joel; Queen Elizabeth Hospital Birmingham, Physiotherapy<br>Power, Dominic; Queen Elizabeth Hospital Birmingham, The Birmingham Peripheral Nerve Injury Service<br>Kyte, Derek; University of Birmingham, Institute of Applied Health Research<br>Jerosch-Herold, Christina; University of East Anglia, Health Sciences
**Primary Subject Heading**:	Neurology
Secondary Subject Heading:	Emergency medicine, Rehabilitation medicine, Surgery
Keywords:	Neurological injury < NEUROLOGY, Limb reconstruction < ORTHOPAEDIC & TRAUMA SURGERY, Plastic & reconstructive surgery < SURGERY, Neurological pain < NEUROLOGY, Elbow & shoulder < ORTHOPAEDIC & TRAUMA SURGERY, Trauma management < ORTHOPAEDIC & TRAUMA SURGERY

SCHOLARONE™
Manuscripts

For peer review only - http://bmjopen.bmj.com/site/about/guidelines.xhtml

I, the Submitting Author has the right to grant and does grant on behalf of all authors of the Work (as defined in the below author licence), an exclusive licence and/or a non-exclusive licence for contributions from authors who are: i) UK Crown employees; ii) where BMJ has agreed a CC-BY licence shall apply, and/or iii) in accordance with the terms applicable for US Federal Government officers or employees acting as part of their official duties; on a worldwide, perpetual, irrevocable, royalty-free basis to BMJ Publishing Group Ltd ("BMJ") its licensees and where the relevant Journal is co-owned by BMJ to the co-owners of the Journal, to publish the Work in this journal and any other BMJ products and to exploit all rights, as set out in our licence.

The Submitting Author accepts and understands that any supply made under these terms is made by BMJ to the Submitting Author unless you are acting as an employee on behalf of your employer or a postgraduate student of an affiliated institution which is paying any applicable article publishing charge ("APC") for Open Access articles. Where the Submitting Author wishes to make the Work available on an Open Access basis (and intends to pay the relevant APC), the terms of reuse of such Open Access shall be governed by a Creative Commons licence – details of these licences and which Creative Commons licence will apply to this Work are set out in our licence referred to above.

Other than as permitted in any relevant BMJ Author's Self Archiving Policies, I confirm this Work has not been accepted for publication elsewhere, is not being considered for publication elsewhere and does not duplicate material already published. I confirm all authors consent to publication of this Work and authorise the granting of this licence.

For peer review only

Title Page

Title of article

Developing a core outcome set for traumatic brachial plexus injuries: a systematic review of outcomes.

Corresponding author

Caroline Miller (corresponding author)

School of Health Sciences,

University of East Anglia,

Norwich,

UK,

NR4 7TJ

Tel no: 00447743501508

Email: caroline.miller@uea.ac.uk

Twitter handle: @c_ahern26

Co-authors

Jane Cross

School of Health Sciences,

University of East Anglia,

Norwich,

UK,

Twitter handle: @JaneCross12

For peer review only

Joel O Sullivan,
Physiotherapy Department,
Queen Elizabeth Hospital Birmingham,
Birmingham
UK,
Twitter handle: @jos2305

Dominic M Power,
The Birmingham Peripheral Nerve Injury Service,
Queen Elizabeth Hospital Birmingham,
Birmingham,
UK,
@dominicpower1

Derek G Kyte,
Centre for Patient-Reported Outcomes Research,
University of Birmingham,
Birmingham,
UK

Christina Jerosch-Herold,
School of Health Sciences,
University of East Anglia,
Norwich,
UK,
Twitter handle: @tinajerosch

Word count, 3,797

For peer review only

ABSTRACT

Objective To identify what outcomes have been assessed in traumatic brachial plexus injury (TBPI) research to inform the development of a Core Outcome Set for TBPI.

Design Systematic review

Method Medline (OVID), EMBASE, CINAHL, and AMED were systematically searched for studies evaluating the clinical effectiveness of interventions in adult traumatic brachial plexus injuries from January 2013 to September 2018 updated in May 2021.Two authors independently screened papers. Outcome reporting bias was assessed. All outcomes were extracted verbatim from studies. Outcomes from patient reported or performance outcome measures were extracted directly from the instrument. Variation in outcome reporting was determined by assessing the number of unique outcomes reported across all included studies. Outcomes were categorized into domains using a prespecified taxonomy.

Results Verbatim outcomes (n= 1491) were extracted from 138 studies including 32 questionnaires. Unique outcomes (n= 157) were structured into four core areas and 11 domains. Outcomes within the musculoskeletal domain were measured in 86% of studies, physical functioning in 25%, emotional functioning in 25% and adverse events in 33%. We identified 63 different methods for measuring muscle strength, 16 for range of movement and 63 studies did not define how they measured movement. Over 2/3rds of outcomes were

incompletely reported in prospective studies.

Conclusion This review of outcome reporting in traumatic brachial plexus injury research demonstrated an impairment focus and heterogeneity. A core outcome set would ensure

For peer review only - http://bmjopen.bmj.com/site/about/guidelines.xhtml

standardized and relevant outcomes are reported to facilitate future systematic review and meta-analysis.

Prospero registration number: CRD42018109843

Strengths and limitations of this study

- This study is a comprehensive and systematic review of all reported clinical outcomes reported in traumatic brachial plexus studies from 2013- 2021 inclusive.
- Unique outcomes were systematically categorized into a clear taxonomy to inform the development of a core outcome set.
- Definition of unique outcomes and categorisation was conducted by researchers and clinicians to account for multidisciplinary perspectives.
- Outcome reporting bias was assessed in included prospective and randomized controlled trials

For peer review only - http://bmjopen.bmj.com/site/about/guidelines.xhtml

INTRODUCTION

A traumatic brachial plexus injury (TBPI) is a major injury to the brachial plexus. It can result in significant functional, social, psychological and economic effects, [1,2] with most occurring in young men as a result of motorbike accidents,[3]. Survival from major trauma is increasing,[4] and with this an increase in the incidence of TBPI,[5] which accounts for 1.2% of polytrauma,[6].The complex and chronic nature of the injury is associated with significant healthcare costs,[7] in addition to indirect costs estimated at up to $2.34 million (in 2017 dollars) over the lifetime of an manual labourer in the USA with a TBPI,[8]. There are multiple strategies for managing a patient with a TBPI with recent advancements in nerve microsurgery,[9] and robotics,[10] resulting in increased treatment options. The choice of treatment should be made using up-to-date, high quality scientific evidence,[11,12].

Ideally, a meta-analysis would identify the most effective treatment for an individual with a TBPI, however, such analysis requires homogenous outcome measurement and reporting across studies to enable optimum synthesis. Indeed, despite increasing numbers of TBPI studies, outcome heterogeneity and poorly defined outcomes has been highlighted as a significant challenge to evidence synthesis in two recent systematic reviews,[13,14]. There is now international agreement that the definition of a core outcome set (COS) for TBPI is a priority,[15,16]. A COS is a minimum agreed set of outcomes to be reported and measured in all studies,[17,18]. Development of a COS has been shown to reduce heterogeneity of outcome reporting in other health conditions, with 81% of trialists in rheumatoid arthritis (RA) now measuring the COS for RA,[19].

For peer review only - http://bmjopen.bmj.com/site/about/guidelines.xhtml

To date a minimum set of outcomes, important to patients and professionals for reporting in TBPI studies, has not been agreed. The choice of what are important outcomes to measure in TBPI is complex due to patient heterogeneity with different mechanisms, locations and severity of injury. COS methodology is continuously being refined and promoted by the Core Outcome Measures in Effectiveness Trials (COMET) initiative [20]. Development of a COS usually begins with identification of a long list of outcomes which is then prioritised through a consensus process. This systematic review sits within the larger global COMBINE project to identify a COS for TBPI. A Delphi study and consensus meeting, informed by data from this systematic review and interviews with people with the injury, will prioritise the final COS for TBPI.

As a first step in the development of an international COS for TBPI we conducted a systematic review to identify outcomes reported and measurement instruments used and their timing in the literature. The final step of the global project will match the COS to existing validated measurement instruments and make recommendations on when they should be collected, therefore it was necessary to identify currently used instruments and their timepoints also.

For peer review only

For peer review only - http://bmjopen.bmj.com/site/about/guidelines.xhtml

The aim of this review was to:

1. Identify what outcome domains are assessed in studies evaluating surgical and non-surgical treatment for TBPI.
2. Compare the definitions of outcomes and time points of outcomes assessed.
3. Assess selective reporting bias in included prospective studies and randomized controlled trials.
4. Identify how the outcomes were measured, that is what validated or non-validated instruments are used.

METHODS

We followed the methods described in the Cochrane Handbook for Systematic Reviews of Interventions,[21] and report in accordance with the Preferred Reporting Items for Systematic Reviews and Meta-Analysis (PRISMA) guidelines,[22]. The systematic review protocol was prospectively registered with PROSPERO (PROSPERO registration number: CRD42018109843). Deviations from the protocol are reported in supplementary file 1.

Identification of studies

We conducted an electronic search of Medline (OVID), EMBASE (OVID), CINAHL and AMED on the 18th September 2018. Studies published between 01 Jan 2013 and 18 September 2018 were included to reflect outcomes employed in current TBPI care. An example of the search strategy for Ovid MEDLINE is presented in supplementary file 2. The thesaurus vocabulary of each database was used to adapt search terms. Boolean operators (AND, OR)

For peer review only

were used to narrow or widen the search and no language restrictions were applied. The search was rerun on the 07 May 2021 to identify any additional outcomes.

Study eligibility

Studies were included if they met the following criteria:

Study type: Any controlled and uncontrolled experimental and observational studies evaluating interventions in traumatic brachial plexus injury including case reports, case series, case studies, prospective and retrospective cohort studies, randomized and non-randomized clinical trials. When the search was rerun in May 2021 only prospective cohort and clinical trials were included. We excluded conference proceedings, abstract only publications and those not involving human subjects.

Participants: Studies reporting outcomes in individuals with traumatic brachial plexus injury aged 16 years or over. Studies of patients with obstetric brachial plexus injuries were excluded.

Interventions: Any surgical or non-surgical intervention for TBPI.

Outcomes: All outcomes reported in the published abstract, methods or results. These included physiological and functional outcomes, adverse events and patient reported outcomes (PROs) either reported in the study or subsequently extrapolated from the PRO instruments.

Language: Non-English language publications were included

For peer review only - http://bmjopen.bmj.com/site/about/guidelines.xhtml

Study selection process

The reference management software Mendeley was used to compile the literature, with duplicates removed. Authors (X and X) independently screened the titles and then the abstracts against the eligibility criteria. Disagreements were discussed and a third reviewer (x) was involved where required. Studies appearing to meet the inclusion criteria based on title and abstract were retrieved as full text articles, and were read to assess for eligibility with decisions on inclusion and exclusion recorded. Disagreements in study selection were resolved by discussion within the research team (x, x, x).

Quality assessment

The aim of this review was to identify outcomes reported in studies rather than synthesise data on intervention effectiveness. However, selective outcome reporting can provide information on what outcomes authors prioritize. We used a modified version of Kirkham et al's matrix [23, 24] to assess outcome reporting bias (ORB) in prospective studies and randomized controlled trials (See ORB instrument in supplementary file 3). Two independent reviewers (XX &XX) performed the assessment of ORB for all outcomes.

Data Extraction

Data were extracted into a piloted data extraction sheet (Microsoft Excel). General data extracted from each study included author, study design, recruiting country, publication year, number of participants, gender, mean age, level of TBPI and intervention tested. The following information was extracted regarding outcomes: each outcome reported

For peer review only - http://bmjopen.bmj.com/site/about/guidelines.xhtml

(verbatim), area of body assessed if relevant (shoulder, elbow, wrist or hand), method of administration, name of measure, timepoints of measure and reported complications. The number of outcomes per study was also documented.

Data extraction was performed independently by X and X for the first 20% of included studies. These were compared, and disagreements discussed and resolved through debate or discussion with a third reviewer (X). Following this a further ten percent of studies had data extracted by both X and X. Due to the high level of agreement between reviewers (91% agreement) on outcomes extracted, at this stage, the remaining studies underwent extraction by a single reviewer (X).

Where a validated PRO or performance outcome measurement was used and composed of multiple items, the following data was extracted by the first author: verbatim name of the instrument, verbatim wording for each individual item. A performance outcome measurement was defined as "A measurement based on a standardized task performed by a patient that is administered and evaluated by an appropriately trained individual or is independently completed" [25]. The frequency of use of instruments was noted and compared between studies. The instruments were categorized as: (i) General Health (generic - for use with any patient); (ii) Upper limb physical function (region-specific); (iii) Symptom or domain specific (to assess a single symptom e.g. pain) and (iv) Condition Specific. Timepoints of measurement of all outcomes were noted. If the outcome was assessed at different timepoints then all timings were recorded.

For peer review only

Classification of outcomes into domains and defining unique outcomes

Identically worded and spelled verbatim outcomes were removed at this stage. Identical outcomes measured over different time points were noted as one outcome. Where outcomes were assessed using an instrument containing several items, each individual item was assigned an outcome name using the International Classification of Functioning and following standard linking rules,[26].

X categorized all outcomes into an outcome taxonomy developed by COMET for categorizing outcomes for core outcome set development,[27]. These included 5 core areas and 38 outcome domains. This is presented in supplementary file 4. A long list of all categorized outcomes was presented to researchers (X and X) at a face to face meeting where the categorization of all outcomes was reviewed using the recommended taxonomy. Subdomains were created within the larger taxonomy to manage the large variation in TBPI clinical outcomes extracted. Disagreements not resolved at this stage were discussed further with subject experts (for example, the Adverse Event domain was discussed with a surgeon).

Due to the diversity in terminology used to report outcomes, we grouped similar outcomes within each subdomain. It is recommended that outcomes with different words, phrasing, or spelling addressing the same concept should be categorized as a unique outcome,[28]. For example, active range of motion of shoulder abduction and active goniometry of shoulder abduction were named as active shoulder abduction range and grasp strength and grip strength were named as grip strength. Independent meetings were held with four subject experts to ratify and define unique outcome names within each domain.

For peer review only - http://bmjopen.bmj.com/site/about/guidelines.xhtml

Patient and public involvement

The need for a COS in TBPI care was conceived following discussions with patients and health professionals. Patients highlighted the diverse effect the injury has on their life and that often these outcomes were overlooked by professionals, such as body image. There is a patient advisory group for the COS and the systematic review was discussed at these meetings. Patients were not actively involved in data collection or analysis of this review. Dissemination will occur at the annual traumatic brachial plexus charity UK meeting where updates from the project are presented yearly and through a six monthly newsletter.

RESULTS

Included studies

The searches retrieved 2819 studies, after removing duplicates 2051 studies remained. Titles and abstract review identified 243 potentially relevant articles. Of these, 105 studies did not meet the inclusion criteria and were excluded (PRISMA flow diagram; figure 1) thus, 138 studies formed the basis of this review. All included studies are presented in supplementary file 5.

Place figure 1 here

Figure 1. Preferred Reporting Items for Systematic Reviews and meta-analysis flow diagram.

For peer review only - http://bmjopen.bmj.com/site/about/guidelines.xhtml

Study characteristics

Thirty-three countries from six continents recruited 3328 participants into the 138 studies (Table 1). Of the 138 studies, 87 (63%) were retrospective case series with most studies published from Asia (n=62, 45%). The most frequently studied surgical intervention was nerve transfers (n=66, 48%).

For peer review only

Table 1. Characteristics and demographics of included studies

	Study number (%)
Number of retrospective studies	87/138(63)
Number of prospective studies	24/138 (17)
Number of case studies	23/138(17)
Randomized controlled trial	4/138 (3)
**World region recruitment**	
Asia	62/138(45)
North America	20/138(14)
South America	23/138(17)
Europe	28/138(20)
Africa	3/138(2.2)
Australasia	2/138(1.5)
**Year published**	
2013	25/138(18)
2014	24/138(17)
2015	15/138(11)
2016	30/138(22)
2017	27/138(20)
2018	11/138(8)
2019 (prospective only)	3/138 (2.2)
2020 (prospective only)	2/138 (1.5)
2021 (prospective only)	1/138 (0.7)
**Gender (total 3328)**	
Male	2737/3328(82)
Female	335/3328(10)
Not stated	256/3328(7.7)
**Site of plexus injury per study (n=138)**	
Upper trunk	27/138(20)

For peer review only

Lower trunk	10/138(7.2)
Pan plexus (all avulsed)	52/138(38)
Infraclavicular	7/138(5)
Mixture	35/138(25)
Unclear	7/138(5)
**Interventions (n=138)**	
Surgical	118/138(86)
Electrotherapy	3/138(2.2)
Pain treatments	11/138 (8)
Rehabilitation	4/138(2.9)
Orthotic	1/138(0.7)
Stem cell	1/138(0.7)
**Types of surgical intervention (n=118)**	
Neurotization	66/118(56)
Tendon transfer	8/118(6.8)
Free flap	17/118(14)
Multiple surgeries	12/118(10)
Contralateral C7	8/118(6.8)
Other	7/118(5.9)

For peer review only

For peer review only - http://bmjopen.bmj.com/site/about/guidelines.xhtml

Outcomes

Extraction of each verbatim outcome domain from each study (e.g range of movement and muscle strength) and those extracted from measures composed of several items identified a total of 1491 verbatim outcomes. After removing duplicates 157 different unique outcomes remained. No single outcome was reported across all 138 studies.

Outcome definition variation. Many outcomes were not clearly defined and different terms were frequently found for the same concept. For example, shoulder abduction strength was described in eleven different ways including 'deltoid strength', 'motor function of axillary nerve', 'motor recovery of shoulder abductors', 'muscle power supraspinatus', 'motor function of deltoid', 'motor function of supraspinatus'.

Outcome timing variation: Forty percent of outcomes were measured between one and three years following intervention. For over 6% of outcomes the timing of the measurement was not stated. See Figure 2.

Place Figure 2 here

Figure 2. Outcome measurement timepoints

For peer review only

For peer review only - http://bmjopen.bmj.com/site/about/guidelines.xhtml

Outcome domains: The 157 different types of outcomes were categorized into four core areas (Physiological and Clinical, Life Impact, Resource Use, Adverse Events/Complications) and 11 domains according to the COMET recommendations,[24]. See supplementary file 6. The core area Physiological/Clinical included three domains: musculoskeletal and connective tissue outcomes, nervous system outcomes and general/symptom outcomes. The core area Life Impact included seven domains: physical functioning, social functioning, role functioning, emotional functioning, global quality of life, perceived health status and delivery of care. The core area Resource Use included one domain: hospital resources. The core area Adverse Events included one domain: adverse events. No outcome could be placed into the core area Death.

Tables 2 to 4 summarise the number of unique outcomes within each domain and the number of studies reporting these outcomes in each core area. The most frequently reported domains were all in the Physiological/ Clinical core area and included musculoskeletal and connective tissue (86%), nervous system (33%) and symptoms (38%). Forty-six studies (33%) reported complications/ adverse events.

For peer review only

1 Table 2. Physiological /Clinical Core Area

Outcome Domains	Number of unique outcomes reported within domain	Examples of unique outcomes	Number of studies reporting outcomes in domain (%)
Musculoskeletal and connective tissue	18	Active range of movement, muscle strength, muscle fatigue	119/138 (86%)
Nervous system	15	Progression of nerve regeneration, ability to feel light touch, ability to feel pain	46/138 (33%)
General/ symptoms	23	Pain intensity/relief, pain duration, pain quality, pain when arm exposed to cold, stiffness, sleep, paresthesia	52/138 (38%)

2

3

4

5

6

7

8

9

10

11

12

13

14

15

16

17

18

19

For peer review only

For peer review only - http://bmjopen.bmj.com/site/about/guidelines.xhtml

Table 3. Life Impact Core Area

Outcome Domains	Number of unique outcomes reported within domain	Examples of unique outcomes	Number of studies reporting outcomes within domain (%)
Physical functioning	19	Reaching, fine hand movement	35/138 (25%)
Role functioning	23	Return to work, Impact on normal hobbies	38/138 (27%)
Social functioning	7	Social activities with family	32/138 (23%)
Emotional functioning	13	Body image, acceptance	34/138 (25%)
Global quality of life	1	Quality of life	2/138 (1.5%)
Perceived health Status	1	Health status rating	9/138 (6%)
Delivery of care	13	Patient satisfaction, quality of care, patient preference, time to surgery	11/138(8%)

For peer review only

For peer review only - http://bmjopen.bmj.com/site/about/guidelines.xhtml

Table 4. Adverse Events and Resource Use Core Areas

Outcome Domains	Number of unique outcomes reported within domain	Examples of unique outcomes	Number of studies reporting outcomes within domain
**Adverse Events Core Area**			
Donor site morbidity	3	Motor weakness, sensory loss	24/138(17%)
Musculoskeletal	7	Co -contraction, Passive movement	12/138 (8.7%)
Respiratory	4	Pneumothorax	6/138 (4.4%)
Vascular	7	Hematoma	7/138 (5.1%)
Infection	1	Infection	3/138 (2.2%)
General non specified complications	1	General complications	3/138 (2.2%)
**Resource Use Core Area**			
Hospital resource use	1	Operation time	1/138 (.7%)

For peer review only - http://bmjopen.bmj.com/site/about/guidelines.xhtml

Outcome Measurement

In addition to extraction of standalone clinician reported and patient reported outcomes such as muscle power, range or movement or return to work, outcomes were also extracted from individual items contained in a total of 32 different instruments; PRO measures (n= 22), combined clinician-reported and patient-reported measures (n= 3) and performance measures (n= 7). See table 5. These measures were reported 98 times in the included publications. Most outcome measures were used once (n= 22/32, 69%). The most frequently reported measures were the Disabilities of the Arm Shoulder and Hand (DASH,[29]) questionnaire (n=28 studies, 29%) and the Visual Analogue Scale (n=20, 20%). The median number of items per instrument was 15 ranging from one (Visual Analogue Scale, Numerical Rating Scale and Wong Baker Faces rating scale),[30] to 54,[31]. These items mapped to 34 different outcome domains.

There was wide variation in the methods used to measure outcomes. This is presented in supplementary file 7

For peer review only

(Measurement instruments mapped to domains). For example; 63 different measurements were used to evaluate muscle function, including the British Medical Research Council,[32] twelve different modifications of the British Medical Council, Isokinetics, Dynanometry and Constant - Murley score,[33]. In addition, it was often not clear which instrument was used for measurement of the outcomes. For example, the instrument used to measure active range of movement was not reported in 34% of total times (63/ 186) the outcome was assessed. Finally with regards to method of measurement 61 studies employed a PRO instrument to evaluate the intervention. Prospective and randomized controlled trials were more likely to evaluate outcomes with a PRO (58%;15/26) compared to 36% (31/87) of retrospective studies.

Table 5: Outcome measures used in included studies

		Number of items	Number of scales	Frequency (n=98)
PRO Measures	**Upper limb physical function measures (n= 17)**			
	Disabilities of Arm Shoulder and Hand	38	3	28
	Quick DASH	19	3	1
	Upper Extremity Functional Index	20	0	2
	American Shoulder and Elbow Score	15	0	1
	Modified American Shoulder and Elbow Score	13	0	1
	Simple Shoulder test	12	0	1
	Michigan Hand Questionnaire	37	0	1
PRO & ClinRO Measure	University of California Los Angelus shoulder score	5	0	1
	Constant- Murley	5	0	1

For peer review only - http://bmjopen.bmj.com/site/about/guidelines.xhtml

	MAYO Performance Index	4	0	1
Performance Measures	Jebsen Taylor	7	0	1
	University of New Brunswick Test of Prosthetic Function for Unilateral Amputees (UNB)	30	3	1
	Upper Limb Module Questionnaire	22	3	1
	Action Reach Arm Test	19	4	2
	Southampton Hand Assessment Procedure	26	0	2
	Purdue Peg test	3	0	1
	Activities Measure for Upper Limb Amputees	24	0	1
PRO Measures	**Generic questionnaires (n=3)**			
	36 item short form survey (SF36)	36	8	8
	Patient Specific Functional Score	4	0	2
	EQ5D-3L	6	0	1
	**Condition specific questionnaires (n=1)**			
	Trinity Amputation and Prosthesis scale	54	5	1
	**Symptom specific questionnaires (n=10)**			
	Visual Analogue Scale	1	0	20
	Numerical Rating Scale	1	0	6
	Wong Baker Faces rating scale	1	0	1
	Brief pain inventory	15	6	4
	Neuropathic pain symptom inventory	10	5	1
	University of Washington Neuropathic score	10	3	1
	McGill Pain Questionnaire	28	3	2
	McGill Pain Questionnaire SF	17	3	1
	McGill Pain Questionnaire (Japanese version)	17	3	1
	Self- rating anxiety scale	20	0	1
	Zung Self rating Depression scale	20	0	1

For peer review only - http://bmjopen.bmj.com/site/about/guidelines.xhtml

Outcome Reporting Bias

Figure 3. illustrates the reporting status of outcomes (n=173) across the included prospective case series, cohort and randomized controlled studies (n=26). None of the studies were prospectively registered. Fewer than one third of the outcomes in the prospective case series and cohort studies and half of outcomes in randomized controlled studies were “completely” reported.

Place Figure 3 here.

Figure 3. Cumulative bar chart showing number of outcomes within each reporting bias category across study types.

For peer review only

DISCUSSION

This systematic review aimed to identify what outcome domains have been reported in studies evaluating interventions for TBPI, examine outcome definitions and timepoints and identify the instruments used to assess outcomes. We found a wide variation in reported outcomes, timing of outcomes and outcome instruments used. Furthermore, a lack of standardized definition for commonly reported outcomes was observed. This heterogeneity in outcome reporting across studies hinders evidence synthesis and results in research waste,[34].

The most commonly reported core area was Physiological/ Clinical including musculoskeletal, nervous system and symptom domains. Eighty-six percent of studies reported musculoskeletal outcomes. However, there were 21 different outcomes reported in this category making comparison between studies difficult. Furthermore, the diversity of measures used to assess the outcomes increases the difficulty with synthesis. For example, muscle function/ strength was assessed using 59 different measures, whilst 10 studies did not report what measure they used. To compound this muscle strength was assessed by both physical examination by a clinician (86%) and also by asking the patient(10%).

Only 44% of studies (61/138) evaluated PROs and within these studies there was significant heterogeneity in the measurement instrument used. Twenty-five different instruments were used with 17 only ever used once. The DASH was the most common instrument employed, in almost half the studies evaluating a PRO. The PRO instruments also varied greatly in terms of content with some as simple as a single item whilst others included up to 54 items. Over 408 individual questionnaire items were evident from the 25 PRO

For peer review only

instruments mapping to 34 different outcomes domains. This highlighted a lack of consistency with no domain being measured by all PRO instruments. None of the included PRO assessments were designed specifically for individuals with a TBPI. Although this may be beneficial in terms of comparison with other conditions, such instruments may not be sensitive to issues of importance to patients with TBPI. Finally, it was evident that prospective studies and randomized controlled trials were more likely to use patient reported outcomes to evaluate interventions. This may correspond with the higher methodological rigour associated with these study designs. However the majority of studies evaluating interventions in TBPI were retrospective (63%). These issues combined pose major questions regarding the clinical interpretation of results from TBPI studies.

It is clear that that individuals with a TBPI suffer significant emotional and psychoscocial issues,[1,35]. However such issues were infrequently and inconsistently measured within this review. Only two studies evaluated Quality of Life [36,37] . Similarly, physical, role and social functioning outcomes were reported in 25%, 27% and 23% of studies respectively. This relates strongly to the use of the DASH within the studies. Indeed, emotional functioning was reported in 34 studies, 28 of these studies used the DASH which has one item on confidence and capability mapping to this domain. If the DASH was excluded, only six studies would assess outcomes within the emotional functioning domain. This is surprising considering the existing literature which evidences the complex emotional and psychological factors, individuals face when adjusting to their injury,[1,38].

For peer review only - http://bmjopen.bmj.com/site/about/guidelines.xhtml

Complications/adverse events were reported in one third of studies. Documentation of complications is crucial to improve patient care and gather data for benchmarking. In 1992, the Clavien-Dindo classification,[39] was introduced to assist with classification of complications to enable comparison between studies,[39]. However, within the adverse events outcomes identified in this review there was heterogeneity. Of the 37 verbatim outcomes reported within the donor morbidity (motor) outcome 19 did not define how this was assessed.

Outcome Reporting Bias

Only four studies included in this review were randomized controlled trials [40,41,42,43]. However despite prospective trial registration on a public registry being a condition of publication [44] none of the randomized trials on TBPI were registered. We also found marked selective outcome reporting in the included prospective and randomized TBPI studies. Most outcomes were only partially reported, frequently lacking specific detail about the outcome result or time of measurement, omitting certain outcome results or lacking detail needed for meta-analysis. This outcome reporting bias identified in current TBPI literature threatens the validity of the evidence based practice in TBPI because it potentially overestimates the effect of treatments or distorts results of studies. This contributes to research waste and critically delays advancement of care for patients.

There are some limitations in this review. We excluded outcomes from older studies to ensure we identified outcomes relevant to contemporary TBPI care. Detailed risk of bias assessment was not undertaken, however the review was designed to identify the breadth of reporting in the literature and not to examine the effectiveness of interventions. The

For peer review only

strengths of this review are that the protocol and the data extraction form were pre-specified, prospectively registered and the literature search systematic. To account for multidisciplinary perspectives, researchers and clinicians where involved in categorizing outcomes into domains. It is the first review to detail the extent of outcome heterogeneity in TBPI research using a systematic method. International and non-English publications were included to reduce the risk of selection bias.

Variation in definitions and measurement of outcomes has been found within other areas of healthcare. Outcome heterogeneity is found in the reporting of outcomes relating to burn care,[45] breast reconstruction,[46] and spinal cord injury,[47] amongst others. A recent review of outcome reporting within burns illustrated wound healing was defined in 166 different ways across 147 studies,[45]. A solution to the variation in outcome reporting across studies in TBPI is the development of a COS,[20]. This has been shown to improve consistency of outcome reporting,[19,48]. Development of a COS in TBPI would not restrict the range of outcomes that can be measured. Researchers and clinicians would still be free to select additional outcomes but the inclusion of such a COS would facilitate synthesis of evidence,[49,50]. Whilst work has begun in obstetric brachial plexus injuries to develop a minimum data set[51], there is no COS for TBPI.

Considerable work has been done by the Core Outcome Measures in Effectiveness Trials (COMET) initiative through dissemination of resources for COS development and support for methodological development. COMET recommends a five step process to develop a COS: define the scope, assess the need, develop the protocol, determine what to measure and determine how to measure,[52]. This systematic review addresses these first two steps for

For peer review only - http://bmjopen.bmj.com/site/about/guidelines.xhtml

For peer review only

the development of the COS in TBPI care. This review has shown the majority of TBPI studies use only clinician reported outcomes to evaluate interventions. However they do not adequately capture patients' health related quality of life,[53] and may underestimate the impact of a condition,[54]. Concurrent qualitative work to identify outcomes which are important to individuals with a TBPI has been completed by this group. The next stage involves integration of all potential outcomes from this review and the qualitative work into a long list of domains. Healthcare professionals and patients will be invited to prioritize these outcomes during a three round international online Delphi process and consensus meeting. This will strengthen the case for uptake of a COS for TBPI as it represents patients' and clinicians' perspectives on what outcomes are important. The final stage will map existing validated measures to the outcome domains in the final COS. A future study will evaluate the psychometric properties of those mapped measurement instruments and identify where new measures need to be developed.

For peer review only

CONCLUSION

This systematic review has shown that outcome reporting in TBPI care is heterogenous and impairment focused with a lack of standardized definitions for commonly reported outcomes. This makes it difficult to compare and combine data from studies to inform decision making in clinical practice. The measurement instruments used in the studies were also often not clear, particularly when range of movement was assessed. In future studies, authors need to be clearer with descriptions of outcomes assessed and how they were measured. Less than half the studies in this review evaluated outcomes using PRO measures. Given that TBPI has a significant impact on health-related quality of life, it is recommended that authors of future studies include PROs in future studies. We have identified a list of potentially relevant outcomes and categorized these into a clear taxonomy. This will inform the next stage of developing a COS for TBPI where patients, surgeons and therapists will be involved in a consensus process to decide the final outcomes included in a COS for TBPI.

Acknowledgements

We would like to thank Colin Shirley for his assistance and guidance categorising neurophysiological outcomes

For peer review only

Competing Interests

Conflicting interests: CM, CJH, JC, DMP and JOS declare no potential conflicts of interest with respect to the research, authorship and publication of this article. DGK reports grants from NIHR, grants from Innovate UK, grants from NIHR Birmingham Biomedical Research Centre, grants from NIHR SRMRC at the University of Birmingham and University Hospitals Birmingham NHS Foundation Trust, personal fees from Merck, personal fees from GSK, grants from Macmillan Cancer Support, grants from Kidney research UK, outside the submitted work; .

Funding

The work was supported by the National Institute of Health Research [grant number ICA-CDRF-2017-03-039]

Ethical approval

Ethical approval was not sought for the present study because it was a systematic review and did not involve human or animal subjects.

Informed consent

Informed consent was not sought for the present study because it was a systematic review and did not involve human participation

For peer review only

Data availability

All data relevant to the study are included in the article or uploaded as supplementary information.

Contributorship

CM, CJH and JC conceived and designed the review. CM and JOS reviewed the titles, abstracts and full text papers for eligibility. Authors resolved disagreements by discussion or where necessary CJH and DMP offered their view. CM and JOS were responsible for extracting data and data extraction was verified by CJH. CM and JOS independently reviewed outcome reporting bias. CM, CJH and JC categorised outcomes. Categorisation was reviewed and edited by DMP and DK. CM prepared the manuscript. CJH,JC, DMP, DK and JOS reviewed and edited the manuscript.

For peer review only - http://bmjopen.bmj.com/site/about/guidelines.xhtml

References

[1] Brito S, White J, Thomacos N, et al. The lived experience following free functioning muscle transfer for management of pan-brachial plexus injury: reflections from a long-term follow-up study. *Disabil Rehabil* 2019;1:1–9.

[2] Morris M, Daluiski A, Dy C. A thematic analysis of online discussion boards for brachial plexus injury. *J Hand Surg AM* 2016;41(8):813-8.

[3] Kaiser R, Waldauf P, Ullas G, et al. Epidemiology, etiology, and types of severe adult brachial plexus injuries requiring surgical repair: systematic review and meta-analysis. Neurosurg Rev 2020;43(2):443-452.

[4] Moran CG, Lecky F, Bouamra O, et al. Changing the system - major trauma patients and their outcomes in the NHS (England) 2008-17. *EClinicalMedicine* 2018;13–21.

[5] Dy CJ, Peacock K, Olsen MA, et al. Incidence of surgically treated brachial plexus injury in privately insured adults under 65 years of age in the USA. *Hosp Spec Surg J* 2020;online.

[6] Midha R. Epidemiology of brachial plexus injuries in a multitrauma population. *Neurosurgery* 1997;40:1182–8

[7] Felici N, Zaami S, Ciancolini G, et al. Cost analysis of brachial plexus injuries: Variability of compensation by insurance companies before and after surgery. *Handchir Mikrochir Plast Chir* 2014;46:85–9.

[8] Hong TS, Tian A, Sachar R, et al. Indirect cost of traumatic brachial plexus injuries in the United States. *J Bone Joint Surg Am* 2019;101:e80.

[9] Bhandari PS, Maurya S. Recent advances in the management of brachial plexus injuries. *Indian J Plast Surg* 2014;47:191–8.

For peer review only - http://bmjopen.bmj.com/site/about/guidelines.xhtml

[10] Kubota S, Hara Y, Shimizu Y, et al. A newly developed upper limb single-joint HAL in a patient with elbow flexion reconstruction after traumatic brachial plexus injury: A case report. *Interdiscip Neurosurg Adv Tech Case Manag* 2017;10:66–8.

[11] Dickinson HD. Evidence-based decision-making: an argumentative approach. *Int J Med Inform* 1998;51:71–81.

[12] Sackett DL, Rosenberg WM, Gray JA, et al . Evidence based medicine: what it is and what it isn't. *BMJ* 1996;312:71–2.

[13] Ayhan E, Soldado F, Fontecha CG, et al. Elbow flexion reconstruction with nerve transfer or grafting in patients with brachial plexus injuries: A systematic review and comparison study. *Microsurgery* 2020;40:79–86.

[14] Donnelly MR, Rezzadeh KT, Vieira D et al. Is one nerve transfer enough? A systematic review and pooled analysis comparing ulnar fascicular nerve transfer and double ulnar and median fascicular nerve transfer for restoration of elbow flexion after traumatic brachial plexus injury. *Microsurgery* 2020;40:361–9.

[15] Dy CJ, Garg R, Lee SK, et al. A systematic review of outcomes reporting for brachial plexus reconstruction. *J Hand Surg Am* 2015;40:308–13.

[16] Hill B, Williams G, Olver J, et al. Letter regarding outcome reporting for brachial plexus reconstruction. *J Hand Surg Am* 2015;40:1504.

[17] Gargon E, Gurung B, Medley N, et al. Choosing important health outcomes for comparative effectiveness research: a systematic review. *PLoS One* 2014;17(7):A435

[18] Williamson P, Altman D, Blazeby J et al. Driving up the quality and relevance of research through the use of agreed core outcomes. *J Health Serv Res Policy* 2012;17:1–2.

For peer review only

[19] Kirkham JJ, Bracken M, Hind L, et al. Industry funding was associated with increased use of core outcome sets. *J Clin Epidemiol* 2019;115:90–7.

[20] Williamson PR, Altman DG, Blazeby JM et al. The COMET (Core Outcome Measures in Effectiveness Trials) Initiative. *Trials* 2011;12:A70.

[21] Higgins J, Green S. Cochrane Handbook for Systematic Reviews of Interventions Version (updated March 2011). version 5. 2011.

[22] Shamseer L, Moher D, Clarke M, et al. Preferred reporting items for systematic review and meta-analysis protocols (PRISMA-P) 2015: elaboration and explanation. *BMJ* 2015;349:g7647.

[23] Kirkham JJ, MDwan K, Altman DG et al. The impact of outcome reporting bias in randomised controlled trials on a cohort of systematic reviews. *BMJ* 2010;340:637-40.

[24] Deshmukh SR, Mousoulis C, Marson BA et al. Developing a core outcome set for hand fractures and joint injuries in adults: a systematic review. *Journal of Hand Surgery(Eur)* 2021; 46(5):488-495.

[25] Richardson E, Burnell J, Adams HR, et al. Developing and implementing performance outcome assessments: evidentiary, methodologic, and operational considerations. *Ther Innov Regul Sci.*2019;53(1):146-153.

[26] Cieza A, Geyh S, Chatterji S, et al. ICF linking rules: an update based on lessons learned. *J Rehabil Med* 2005;37:212–8.

[27] Dodd S, Clarke M, Becker L, et al. A taxonomy has been developed for outcomes in medical research to help improve knowledge discovery. *J Clin Epidemiol* 2018;96:84–92.

[28] Young AE, Brookes ST, Avery KNL, et al. A systematic review of core outcome set

For peer review only

For peer review only - http://bmjopen.bmj.com/site/about/guidelines.xhtml

development studies demonstrates difficulties in defining unique outcomes. *J Clin Epidemiol* 2019;115:14–24.

[29] Hudak PL, Amadio PC, Bombardier C, et al. Development of an upper extremity outcome measure: The DASH (disabilities of the arm, shoulder, and head). *Am J Ind Med* 1996;29:602–8.

[30] Baker C, Wong D. Q.U.E.S.T: a process of pain assessment in children (continuing eductation credit). *Orthop Nurs* 1987;6:11–21.

[31] Gallagher P, MacLachlan M. Development and psychometric evaluation of the trinity amputation and prosthesis experience scales (TAPES). *Rehabil Psychol* 2000;45:130–54.

[32] Medical Research Council. Aids to the investigation of the peripheral nervous system. London: Her Majesty's stationary office; 1943.

[33] Constant CR, Murley AH (1987) A clinical method of functional assessment of the shoulder. *Clinical Orthopaedics & Related Research* 1987;214:160-164

[34] Chalmers I, Glasziou P. Avoidable waste in the production and reporting of research evidence. *Lancet* 2009;374:86–9.

[35] McDonald J, Pettigrew J. Traumatic brachial plexus injury: the lived experience. *British Journal of Occupational Therapy* 2014;77(3):147-154.

[36] Kim JH, Shin SH, Lee YR, et al. Ultrasound-guided peripheral nerve stimulation for neuropathic pain after brachial plexus injury: two case reports. *J Anesth* 2017;31:453–7.

[37] Cole T, Nicks R, Ferris S et al. Outcomes after occupational therapy intervention for traumatic brachial plexus injury: A prospective longitudinal cohort study. *Journal of Hand Therapy* 2020;33:528-539

For peer review only - http://bmjopen.bmj.com/site/about/guidelines.xhtml

[38] Miller C, Peek AL, Power D, et al. Psychological consequences of traumatic upper limb peripheral nerve injury: A systematic review. *Hand Ther* 2017;22:35–45.

[39] Clavien P, Sanabria J, Strasberg S. Proposed classification of complications of surgery with examples of utility in cholecystectomy. *Surgery* 1992;111(5):518-26

[40] Razak I, Chung TY, Ahmad S et al. A comparative study of two modalities in pain management of patients presenting with chronic brachial neuralgia. *Journal of Alternative and Complementary Medicine* 2019; 1-7

[41] Ferreira CM, de Carvalho CD, Gomes R et al. Transcranial direct current stimulation and mirrir therapy for neuropathic pain after brachial plexus avulsion: A randomised double-blind, controlled pilot study. *Frontiers in Neurology* 2020; 11:1-10

[42] Martins RS, Siqueira MG, Heise CO et al. A prospective study comparing single and double fascicular transfer to restore elbow flexion after brachial plexus injury. *Neurosurgery 2013;*72:709-715

[43] Tu YK. Tsai YJ, Chang CH et al. Surgical treatment for total root avulsion type brachial plexus injuries by neurotization: a prospective comparison study between total and hemicontralateral C7 nerve root transfer. *Microsurgery* 2014; 34:91-101

[44] Angelis CD, Drazen JM, Frizelle FA et al. Clinical trial registration: a statement from the international committee of medical journal editors. The New England Journal of Medicine 2004; 351:1250-1251

[45] Young AE, Davies A, Bland S et al. Systematic review of clinical outcome reporting in randomised controlled trials of burn care. *BMJ Open* 2019;15;9(2):e025135

[46] Potter S, Brigic A, Whiting PF, et al. Reporting clinical outcomes of breast reconstruction: a systematic review. *J Natl Cancer Inst* 2011;103:31–46.

For peer review only - http://bmjopen.bmj.com/site/about/guidelines.xhtml

[47] Watzlawick R, Antonic A, Sena ES, et al. Outcome heterogeneity and bias in acute experimental spinal cord injury A meta-analysis. *Neurology* 2019;2;93(1):e40-e51

[48] Bautista-Molano W, Navarro-Compán V, Landewé RBM, et al. How well are the ASAS/OMERACT core outcome sets for ankylosing spondylitis implemented in randomized clinical trials? A systematic literature review. *Clin Rheumatol* 2014;33:1313–22.

[49] Clarke M, Williamson PR. Core outcome sets and systematic reviews. *Syst Rev* 2016;5:11.

[50] Clarke M. Standardising outcomes for clinical trials and systematic reviews. *Trials* 2007;8:39.

[51] Pondaag W, Malessy MJA. Outcome assessment for Brachial Plexus birth injury. Results from the iPluto world-wide consensus survey. *J Orthop Res* 2018;36:2533–41.

[52] Williamson PR, Altman DG, Bagley H, et al. The COMET Handbook: version 1.0. *Trials* 2017;18:280.

[53] Pakhomov S, Jacobsen SJ, Chute CG, et al. Agreement between patient-reported symptoms and their documentation in the medical record. *Am J Manag Care* 2008;14:530–9.

[54] Turner GM, Slade A, Retzer A, et al. An Introduction to Patient Reported Outcome Measures (PROMs) in Trauma. *J Trauma Acute Care Surg* 2019;86(2):314-320

Figure 2 Legends

mths, months; NS, not stated; yrs, years.

For peer review only - http://bmjopen.bmj.com/site/about/guidelines.xhtml

For peer review only

For peer review only - http://bmjopen.bmj.com/site/about/guidelines.xhtml

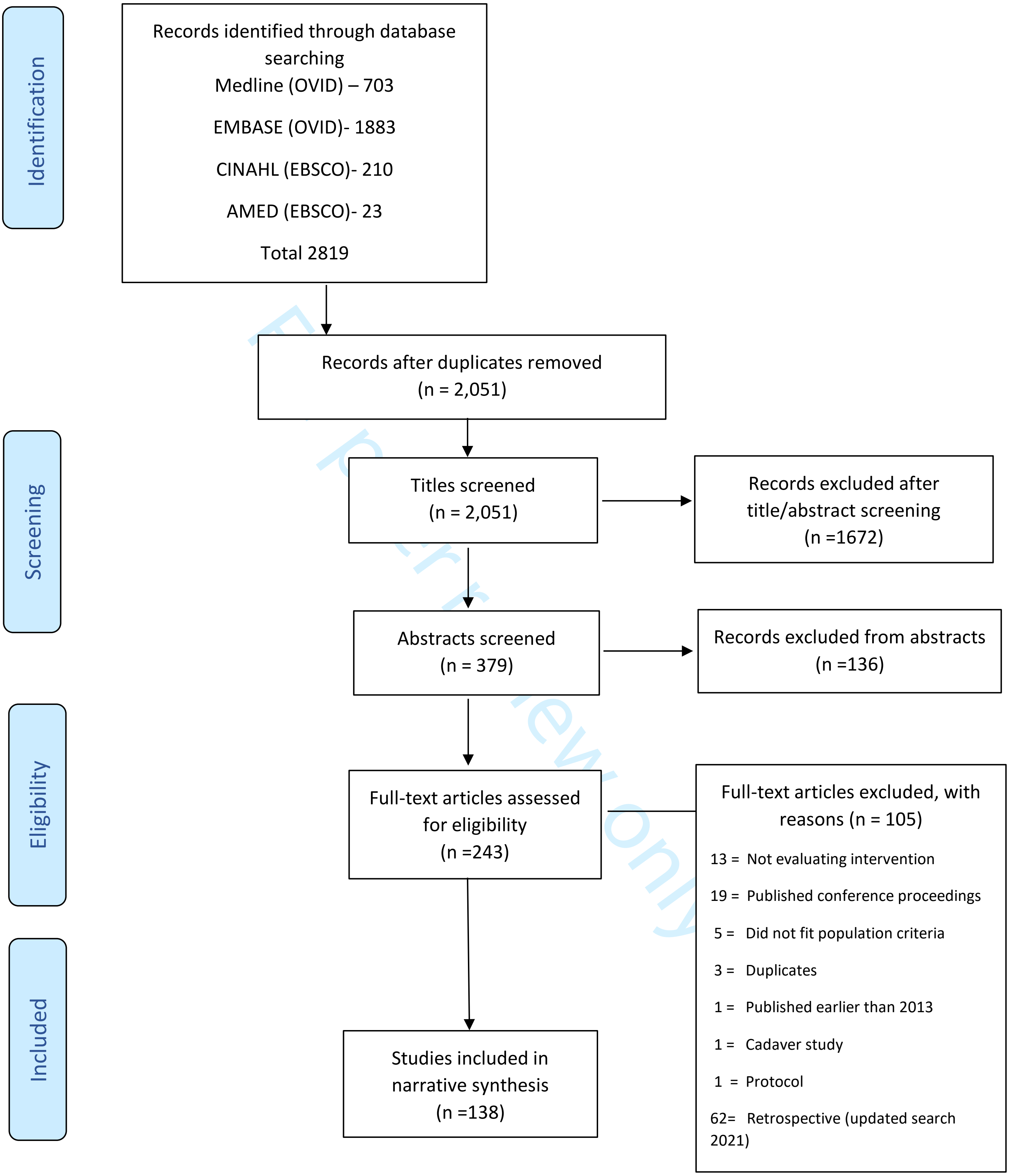
Identification
Records identified through database searching
Medline (OVID) – 703
EMBASE (OVID)- 1883
CINAHL (EBSCO)- 210
AMED (EBSCO)- 23
Total 2819
Records after duplicates removed
(n = 2,051)
Screening
Titles screened
(n = 2,051)
Records excluded after title/abstract screening
(n =1672)
Abstracts screened
(n = 379)
Records excluded from abstracts
(n =136)
Eligibility
Full-text articles assessed for eligibility
(n =243)
Full-text articles excluded, with reasons (n = 105)
13 = Not evaluating intervention
19 = Published conference proceedings
5 = Did not fit population criteria
3 = Duplicates
1 = Published earlier than 2013
1 = Cadaver study
1 = Protocol
62= Retrospective (updated search 2021)
Included
Studies included in narrative synthesis
(n =138)

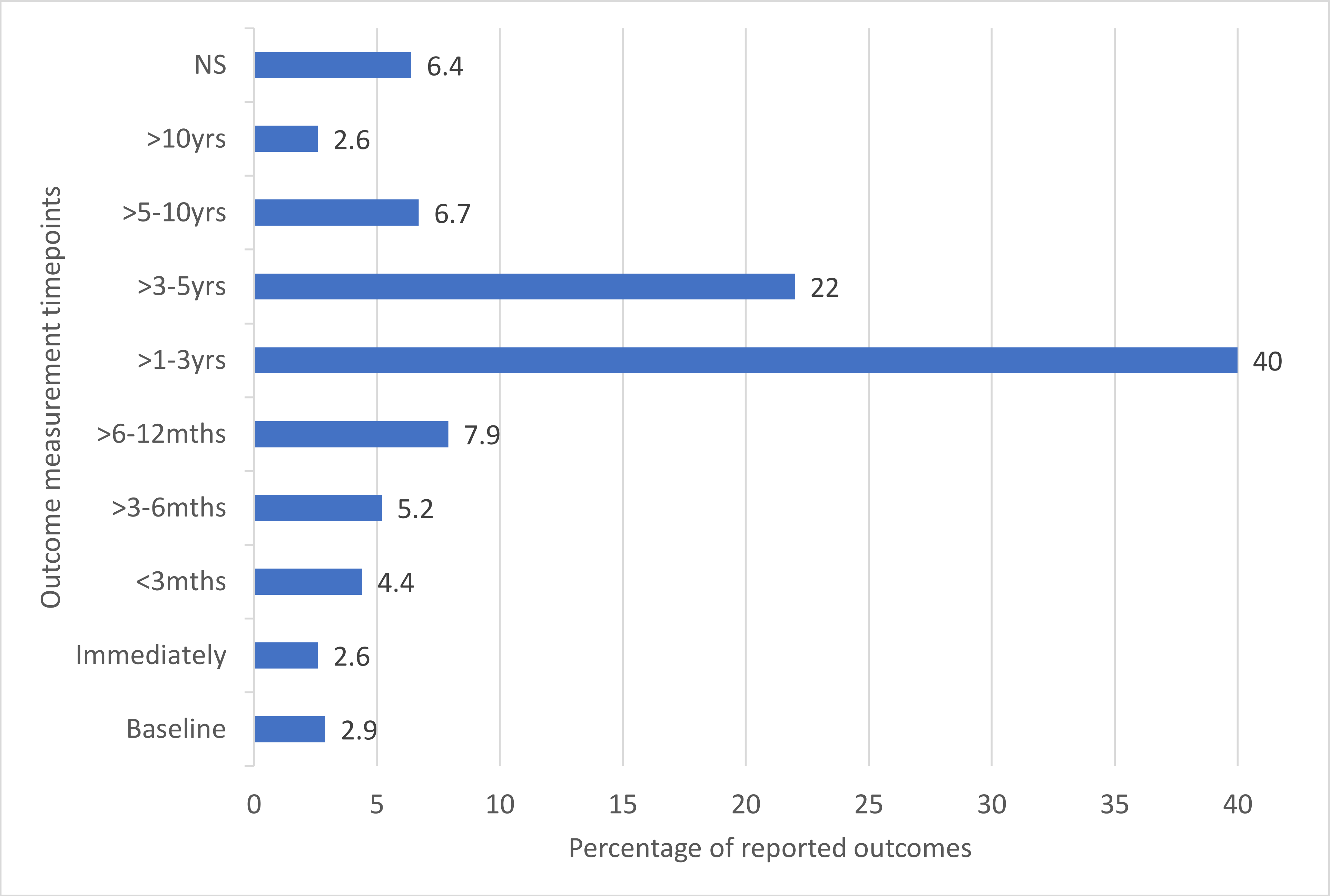
Outcome measurement timepoints
NS
>10yrs
>5-10yrs
>3-5yrs
>1-3yrs
>6-12mths
>3-6mths
<3mths
Immediately
Baseline
6.4
2.6
6.7
22
40
7.9
5.2
4.4
2.6
2.9
0
5
10
15
20
25
30
35
40
Percentage of reported outcomes

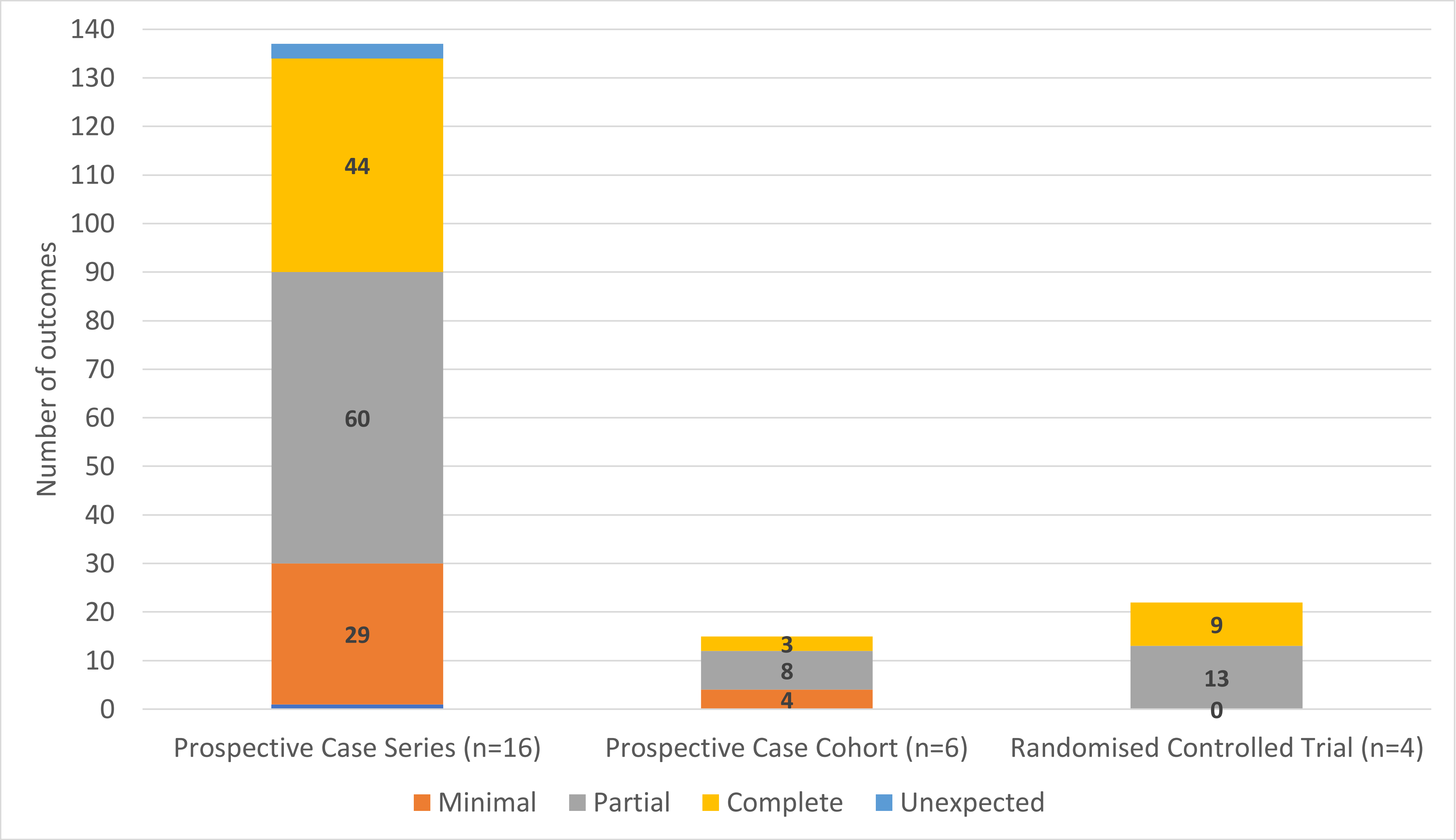
140
130
120
110
100
90
80
70
60
50
40
30
20
10
0
Number of outcomes
44
60
29
3
8
4
9
13
0
Prospective Case Series (n=16)
Prospective Case Cohort (n=6)
Randomised Controlled Trial (n=4)
Minimal
Partial
Complete
Unexpected

Developing a core outcome set for traumatic brachial plexus injuries: a systematic review of outcomes

Supplementary file 1. Deviations from study protocol

Protocol method	Deviation from protocol method with justification
We planned to hand search Journal of Hand Surgery (Eur) and The Journal of Hand Surgery (American).	We did not hand search these Journals as they were all indexed for MEDLINE.
We planned to include studies with participants aged 18 and over within the review.	We reduced the age of include participants to 16 or over as many studies included older teenagers with adults in their studies. On discussion with the research team we concluded that there was no difference between treatment of those aged 16 and over versus aged 18. If we excluded these studies many outcomes used across these age ranges would have been lost.
One search date was originally proposed in the study protocol	We updated the search in May 2021, including prospective and randomized controlled trials to ensure that the outcomes identified and reported in the publication reflected current outcomes in the literature and to ensure that no outcomes were omitted.
No quality assessment was proposed in the original study protocol	Outcome reporting bias was assessed in the included prospective and randomized controlled trials. This was included as it was thought this could improve understanding on what outcomes authors prioritise.

For peer review only

Supplementary file 2. Search strategy systematic review outcome reporting traumatic brachial plexus injuries

Search strategy 10/09/2018 COMBINE systematic review (reran 07 May 2021)

MEDLINE (OVID)

1.(brachial plexus adj3 injur*).mp. [mp=title, abstract, original title, name of substance word, subject heading word, floating sub-heading word, keyword heading word, protocol supplementary concept word, rare disease supplementary concept word, unique identifier, synonyms]

2 (brachial plexus adj3 pals*).mp. [mp=title, abstract, original title, name of substance word, subject heading word, floating sub-heading word, keyword heading word, protocol supplementary concept word, rare disease supplementary concept word, unique identifier, synonyms]

3 (brachial plexus adj3 lesion*).mp. [mp=title, abstract, original title, name of substance word, subject heading word, floating sub-heading word, keyword heading word, protocol supplementary concept word, rare disease supplementary concept word, unique identifier, synonyms]

4 brachial plexopath*.mp. [mp=title, abstract, original title, name of substance word, subject heading word, floating sub-heading word, keyword heading word, protocol supplementary concept word, rare disease supplementary concept word, unique identifier, synonyms]

5 (brachial plexus adj3 traction*).mp. [mp=title, abstract, original title, name of substance word, subject heading word, floating sub-heading word, keyword heading word, protocol supplementary concept word, rare disease supplementary concept word, unique identifier, synonyms]

6 (brachial plexus adj3 avulsion*).mp. [mp=title, abstract, original title, name of substance word, subject heading word, floating sub-heading word, keyword heading word, protocol supplementary concept word, rare disease supplementary concept word, unique identifier, synonyms]

7 Brachial Plexus/in, su, tr [Injuries, Surgery, Transplantation]

8 1 or 2 or 3 or 4 or 5 or 6 or 7

9 limit 8 to (humans and "all adult (19 plus years)")

10. limit 9 to yr="2013 -Current

For peer review only

Supplementary file 2. Search strategy systematic review outcome reporting traumatic brachial plexus injuries

For peer review only

Supplementary file 3. Outcome reporting bias assessment instrument

Outcome Reporting Bias assessment instrument (adapted from Deshmukh et al 2021)

**Study ID**	**Author**	**Registered**	**No clear reporting of outcome through description/table/figure**	**Outcome only by summary comment (e.g. there was no significant difference), no numerical values provided, lack of information so that reporting not meaningful (outcomes but no timepoints)**	**Outcome reported but not at all timepoints; lacks detail to be included in review**	**Outcome reported at all time points (methods, Results)**	**Outcome not specified in registration or prior to results**
			**NOT DONE**	**MIMIMAL**	**PARTIAL**	**COMPLETE**	**UNEXPECTED**

Deshmukh SR, Mousoulis C, Marson BA et al. Developing a core outcome set for hand fractures and joint injuries in adults: a systematic review. Journal of Hand Surgery (Eur) 2021;46(5):488-495

For peer review only

Supplementary File 4 COMET outcome taxonomy

Title: Supplemental File 4 COMET outcome taxonomy - adapted from Dodd et al (2018)

**Core Area**	Outcome Domain
**Death**	1. Mortality/ survival
**Physiological/clinical**	2. Blood and lymphatic system outcomes
	3. Cardiac outcomes
	4. Congenital, familial and genetic outcomes
	5. Endocrine outcomes
	6. Ear and labyrinth outcomes
	7. Eye outcomes
	8. Gastrointestinal outcomes
	9. General outcomes
	10. Hepatobiliary outcomes
	11. Immune system outcomes
	12. Infection and infestation outcomes
	13. Injury and poisoning outcomes
	14. Metabolism and nutrition outcomes
	15. Musculoskeletal and connective tissue outcomes
	16. Outcomes, relating to neoplasms: benign, malignant and unspecified (including cysts and polyps)
	17. Nervous system outcomes
	18. Pregnancy, puerperium and perinatal outcomes
	19. Renal and urinary outcomes
	20. Reproductive system and breast outcomes
	21. Psychiatric outcomes
	22. Respiratory, thoracic and mediastinal outcomes
	23. Skin and subcutaneous tissue outcomes
	24. Vascular outcomes
**Life Impact**	**Functioning**
	25. Physical functioning
	26. Social functioning
	27. Role functioning
	28. Emotional functioning/ well being
	29. Cognitive functioning
	30. Global quality of life
	31. Perceived health status
	32. Delivery of care
	33. Personal circumstances
**Resource use**	**Resource Use**
	34. Economic
	35. Hospital
	36. Need for further intervention
	37. Societal/ carer burden
**Adverse Events**	38. Adverse Events / effects

Dodd S, Clarke M, Becker L et al. A taxonomy has been developed for outcomes in medical research to help improve knowledge discovery. *J Clin Epidemiol*. 2018;96:84-92.

For peer review only

Supplementary file 5. Included Studies

	Study title	First author	Year of publication
**1**	Effectiveness and safety of home-based muscle electrical stimulator in brachial plexus Injury patient	Limthongthang	2014
**2**	Elbow proprioception sense in total arm -type brachial plexus injured patients after neurotisation: a preliminary study	Homreprasert	2014
**3**	Comparison between the anterior and posterior approach for transfer of the spinal accessory nerve to the suprascapular nerve in late traumatic brachial plexus injuries	Souza	2014
**4**	Ultrasound-guided peripheral nerve stimulation for neuropathic pain after brachial plexus injury: two case reports	Kim	2017
**5**	Contralateral lower trapezius transfer for restoration of shoulder external rotation in traumatic brachial plexus palsy: preliminary report and literature review	Satbhai	2014
**6**	Restoration of shoulder abduction in brachial plexus avulsion injuries with double neurotization from the spinal accessory nerve: a report of 13 cases	Huan	2017
**7**	Transfer of the musculocutaneous nerve branch to the brachialis muscle to the triceps for elbow extension: anatomical study and report of five cases	Bertelli	2017
**8**	Posterior approach for accessory to suprascapular nerve transfer: an electrophysiological outcomes study	Rui	2013
**9**	Reliability of functioning free muscle transfer and vascularized ulnar nerve grafting for elbow flexion in complete brachial plexus palsy	Potter	2017
**10**	Management of infraclavicular (Chuang Level IV) brachial plexus injuries: A single surgeon experience with 75 cases	Lam	2015
**11**	Functioning free muscle transfer for the restoration of elbow flexion in brachial plexus injury patients	Estrella	2016
**12**	Radial to axillary nerve transfers: A combined case series	Desai	2016
**13**	Thalamic deep brain stimulation for neuropathic pain after amputation or brachial plexus avulsion	Pereira	2013
**14**	Nerve transfers for shoulder function for traumatic brachial plexus injuries	Estrella	2014
**15**	Results of operative treatment of brachial plexus injury resulting from shoulder dislocation: A study with a long-term follow-up	Gutkowska	2017
**16**	Surgical treatment of brachial plexus posterior cord lesion: A combination of nerve and tendon transfers, about nine patients	Oberlin	2013
**17**	The medial cord to musculocutaneous (MCMc) nerve transfer: a new method to reanimate elbow flexion after C5-C6-C7-(C8) avulsive injuries of the brachial plexus—technique and results	Ferraresi	2014
**18**	Transfer of a terminal motor branch nerve to the flexor carpi ulnaris for triceps reinnervation: anatomical study and clinical cases	Bertelli	2015
**19**	Free functioning gracilis muscle transfer with and without simultaneous intercostal nerve transfer to musculocutaneous nerve for restoration of elbow flexion after traumatic adult brachial pan-plexus injury	Maldonado	2017(a)

Article title: Developing a core outcome set for Traumatic Brachial Plexus Injuries: a systematic review of outcomes

For peer review only - http://bmjopen.bmj.com/site/about/guidelines.xhtml

Supplementary file 5. Included Studies

**20**	Isolated latissimus dorsi transfer to restore shoulder external rotation in adults with brachial plexus injury	Ghosh	2013
**21**	Functional outcome and quality of life after traumatic total brachial plexus injury treated by nerve transfer or single/double free muscle transfers	Satbhai	2016
**22**	Successful graded mirror therapy in a patient with chronic deafferentation pain in whom traditional mirror therapy was ineffective: A case report	Mibu	2016
**23**	Bipolar Transfer of Latissimus Dorsi Myocutaneous Flap for Restoration of Elbow Flexion in Late Traumatic Brachial Plexus Injury: Evaluation of 13 Cases	Azab	2017
**24**	Comparison of objective muscle strength in C5-C6 and C5-C7 brachial plexus injury patients after double nerve transfer	Tsai	2014
**25**	Phantom remodeling effect of dorsal root entry zone lesioning in phantom limb pain caused by brachial plexus avulsion	Son	2015
**26**	Comparison of surgical strategies between proximal nerve graft and/or nerve transfer and distal nerve transfer based on functional restoration of elbow flexion: A retrospective review of 147 patients	Hu	2018
**27**	Reconstruction of shoulder abduction by multiple nerve fascicle transfer through posterior approach	Ren	2013
**28**	Intercostal nerve transfer to neurotize the musculocutaneous nerve after traumatic brachial plexus avulsion: A comparison of two, three, and four nerve transfers	Xiao	2014
**29**	Use of the DEKA Arm for amputees with brachial plexus injury: A case series	Resnik	2017
**30**	Polyester tape scapulopexy for chronic upper extremity brachial plexus injury	Leechavengvongs	2015
**31**	Contralateral C7 nerve transfer with direct coaptation to restore lower trunk function after traumatic brachial plexus avulsion	Wang	2013
**32**	Outcome of surgical reconstruction after traumatic total brachial plexus palsy	Dodakundi	2013
**33**	Bionic reconstruction to restore hand function after brachial plexus injury: a case series of three patients	Aszmann	2015
**34**	Surgical treatment of the plexus brachialis injury using long-lasting electrostimulation	Tsymbalyuk	2013
**35**	Phrenic nerve transfer for reconstruction of elbow extension in severe brachial plexus injuries	Flores	2016
**36**	Direct coaptation of the phrenic nerve with the posterior division of the lower trunk to restore finger and elbow extension function in patients with total brachial plexus injuries	Wang	2016
**37**	A prospective study comparing single and double fascicular transfer to restore elbow flexion after brachial plexus injury	Martins	2013
**38**	Chronic post-traumatic neuropathic pain of brachial plexus and upper limb: a new technique of peripheral nerve stimulation	Stevanato	2014

Article title: Developing a core outcome set for Traumatic Brachial Plexus Injuries: a systematic review of outcomes

For peer review only - http://bmjopen.bmj.com/site/about/guidelines.xhtml

Supplementary file 5. Included Studies

**39**	Effectiveness of contralateral C7 nerve root and multiple nerve transfer for treatment of brachial plexus root avulsion	Wei	2014
**40**	Combined proximal nerve graft and distal nerve transfer for a posterior cord brachial plexus injury	Plate	2013
**41**	The role of elective amputation in patients with traumatic brachial plexus injury	Maldonado	2016
**42**	Early microsurgical management of clavicular fracture combined with brachial plexus injury	Liu	2014(a)
**43**	Contralateral trapezius transfer to restore shoulder external rotation following adult brachial plexus injury	Elhassan	2016
**44**	Comparative study of phrenic nerve transfers with and without nerve graft for elbow flexion after global brachial plexus injury	Liu	2014
**45**	Shoulder and elbow recovery at 2 and 11 years following brachial plexus reconstruction	Wang	2016
**46**	Functional outcomes after treatment of traumatic brachial plexus injuries: clinical study	Aras	2013
**47**	Free gracilis transfer reinnervated by the nerve to the supinator for the reconstruction of finger and thumb extension in longstanding C7-T1 brachial plexus root avulsion	Soldado	2013
**48**	Restoration of hand function in C7–T1 brachial plexus palsies using a staged approach with nerve and tendon transfer	Zhang	2014
**49**	Neurotization to innervate the deltoid and biceps: 3 cases	Dy	2013
**50**	Arthroscopic arthrodesis of the shoulder in brachial plexus palsy	Lenoir	2017
**51**	Outcome of contralateral C7 nerve transferring to median nerve	Gao	2013
**52**	Intercostal nerve transfer to the biceps motor branch in complete traumatic brachial plexus injuries	Cho	2015
**53**	Tactile feedback for relief of deafferentation pain using virtual reality system: a pilot study	Sano	2016
**54**	Functioning free gracilis transfer to reconstruct elbow flexion and quality of life in global brachial plexus injured patients	Yang	2016
**55**	Evaluation of infraspinatus reinnervation and function following spinal accessory nerve to suprascapular nerve transfer in adult traumatic brachial plexus injuries	Baltzer	2017
**56**	Anatomic study of the intercostal nerve transfer to the suprascapular nerve and a case report	Hu	2014
**57**	Shoulder abduction and external rotation restoration with nerve transfer	Kostas-Agnantis	2013
**58**	Contralateral C-7 transfer: is direct repair really superior to grafting?	Bhatia	2017
**59**	Impact of phrenic nerve paralysis on the surgical outcome of intercostal nerve transfer	Kita	2015
**60**	Flow-through anastomosis using a T-shaped vascular pedicle for gracilis functioning free muscle transplantation in brachial plexus injury	Hou	2015
**61**	Free functional muscle transfer tendon insertion secondary advancement procedure to improve elbow flexion	Sechachalam	2017

Article title: Developing a core outcome set for Traumatic Brachial Plexus Injuries: a systematic review of outcomes

For peer review only - http://bmjopen.bmj.com/site/about/guidelines.xhtml

Supplementary file 5. Included Studies

**62**	Dual nerve transfers for restoration of shoulder function after brachial plexus avulsion injury	Chu	2016
**63**	Cortical plasticity after brachial plexus injury and repair: a resting-state functional MRI study	Bhat	2017
**64**	Results of spinal accessory to suprascapular nerve transfer in 110 patients with complete palsy of the brachial plexus	Bertelli	2016
**65**	Magnetic resonance neurographic and clinical long-term results after oberlins transfer for adult brachial plexus injuries	Frueh	2017
**66**	Free functioning gracilis muscle transfer versus intercostal nerve transfer to musculocutaneous nerve for restoration of elbow flexion after traumatic adult brachial pan-plexus injury	Maldonado	2016
**67**	Results of wrist extension reconstruction in C5–8 brachial plexus palsy by transferring the pronator quadratus motor branch to the extensor carpi radialis brevis muscle	Bertelli	2016
**68**	Donor nerve sources in free functional gracilis muscle transfer for elbow flexion in adult brachial plexus injury	Nicoson	2017
**69**	Use of contralateral spinal accessory nerve for ipsilateral suprascapular neurotization in global brachial plexus injury: a new technique	Bhandari	2016
**70**	Objective evaluation of elbow flexion strength and fatigability after nerve transfer in adult traumatic brachial plexus injuries	Marciq	2014
**71**	Outcomes of muscle brachialis transfer to restore finger flexion in brachial plexus palsy	DeGeorge	2017
**72**	Functional outcome of nerve transfers for traumatic global brachial plexus avulsion	Liu	2013
**73**	Transfer of a flexor digitorum superficialis motor branch for wrist extension reconstruction in C5-C8 root injuries of the brachial plexus: a case series	Bertelli	2013
**74**	Outcome after transfer of intercostal nerves to the nerve of triceps long head in 25 adult patients with total plexus root avulsion injury	Gao	2013
**75**	Good sensory recovery of the hand in brachial plexus surgery using the intercostobrachial nerve as the donor	Foroni	2017
**76**	The phrenic nerve as a donor for brachial plexus injuries: is it safe and effective? Case series and literature analysis	Socolovsky	2015
**77**	Complete avulsion of brachial plexus with associated vascular trauma: Feasibility of reconstruction using the double free muscle technique	Hattori	2013
**78**	Long-term outcome of brachial plexus re-implantation after complete brachial plexus avulsion injury	Kachramanoglo u	2017
**79**	Force recovery assessment of functioning free muscle transfers using ultrasonography	Kodama	2014
**80**	Rhomboid nerve transfer to the suprascapular nerve for shoulder reanimation in brachial plexus palsy: A clinical report	Goubier	2016
**81**	Outcome of contralateral C7 transfer to two recipient nerves in 22 patients with the total brachial plexus avulsion injury	Gao	2013
**82**	Comparative study of phrenic and intercostal nerve transfers for elbow flexion after global brachial plexus injury	Liu	2015

For peer review only - http://bmjopen.bmj.com/site/about/guidelines.xhtml

Supplementary file 5. Included Studies

**83**	Donor-side morbidity after contralateral C-7 nerve transfer: results at a minimum of 6 months after surgery	Li	2016
**84**	Outcome after brachial plexus injury surgery and impact on quality of life	Rasulić	2017
**85**	Pronator teres branch transfer to the anterior interosseous nerve for treating C8T1 brachial plexus avulsion: An anatomic study and case report	Yang	2014
**86**	Operative treatment with nerve repair can restore function in patients with traction injuries in the brachial plexus	Stiasny	2015
**87**	Thoracodorsal nerve transfer for triceps reinnervation in partial brachial plexus injuries	Soldado	2016
**88**	Co-infusion of autologous adipose tissue derived neuronal differentiated mesenchymal stem cells and bone marrow derived hematopoietic stem cells, a viable therapy for post-traumatic brachial plexus injury: a case report	Thakkar	2014
**89**	Long-term clinical outcomes of spinal accessory nerve transfer to the suprascapular nerve in patients with brachial plexus palsy	Emamhadi	2016
**90**	Surgical treatment for total root avulsion type brachial plexus injuries by neurotisation: a prospective comparison study between total and hemicontralateral C7 nerve root transfer	Tu	2014
**91**	Deactivation of distant pain-related regions induced by 20-day rTMS: a case study of one-week pain relief for long-term intractable deafferentation pain	Qiu	2014
**92**	End-to-side neurorrhaphy in brachial plexus reconstruction	Haninec	2013
**93**	Reanimation of elbow extension with medial pectoral nerve transfer in partial injuries to the brachial plexus	Flores	2013
**94**	Early post-operative results after repair of traumatic brachial plexus palsy	Mohammad-Reda	2013
**95**	Satisfied patients after shoulder arthrodesis for brachial plexus lesions even after 20 years of follow-up	van der Lingen	2018
**96**	Posterior branch of the axillary nerve transfer to the lateral triceps branch for restoration of elbow extension: case report	Kilka	2013
**97**	Clinical analysis of repairing the whole brachial plexus nerve root avulsion by transferring C7 nerve root from the uninjured side	Liu	2014
**98**	Bipolar transfer of the pectoralis major muscle for restoration of elbow flexion in 29 cases	Cambon-Binder	2018
**99**	Thoracodorsal nerve transfer for elbow flexion reconstruction in infraclavicular brachial plexus injuries	Soldado	2014
**100**	Median nerve fascicle transfer versus ulnar nerve fascicle transfer to the biceps motor branch in C5-C6 and C5-C7 brachial plexus injuries: nonrandomised prospective study of 23 consecutive patients	Cho	2014
**101**	Free functional muscle transplantation of an anomalous femoral adductor with a very large muscle belly: a case report	Kaizawa	2013
**102**	Selective neurotisation of the radial nerve in the axilla using the intercostal nerve to treat complete brachial plexus palsy	Tuohuti	2016

Article title: Developing a core outcome set for Traumatic Brachial Plexus Injuries: a systematic review of outcomes

For peer review only - http://bmjopen.bmj.com/site/about/guidelines.xhtml

Supplementary file 5. Included Studies

**103**	Objective predictors of functional recovery associated with intercostal nerves transfer for triceps reinnervation in global brachial plexus palsy	Flores	2016
**104**	Nerve transfer to relieve pain in upper brachial plexus injuries: does it work?	Emamhadi	2017
**105**	Phrenic nerve transfer versus intercostal nerve transfer for the repair of brachial plexus root avulsion injuries	Abdixbir	2016
**106**	End-to-side neurorrhaphy to restore elbow flexion in brachial plexus injury	Limthongthang	2016
**107**	Chordata method combined with electrotherapy in functional recovery after brachial plexus injury:report of three clinical cases	De Oliveira	2016
**108**	Clinical outcome following transfer of the supinator motor branch to the posterior interosseous nerve in patients with C7-T1 brachial plexus palsy	Xu	2015
**109**	Transposition of branches of radial nerve innervating supinator to posterior interosseous nerve for functional reconstruction of finger and thumb extension in 4 patients with middle and lower trunk root avulsion injuries of brachial plexus	Wu	2017
**110**	Electromyographic findings in gracilis muscle grafts used to augment elbow flexion in traumatic brachial plexopathy	Kazamel	2016
**111**	Double distal intraneural fascicular nerve transfers for lower brachial plexus injuries	Li	2016
**112**	Restoration of elbow and hand function in total brachial plexus palsy with intercostal nerves and C5 root neurotisation. Results in 21 patients	Amal	2016
**113**	The phrenic nerve transfer in the treatment of a septuagenarian with brachial plexus avulsion injury: a case study	Jiang	2018
**114**	Outcomes of transferring a healthy motor fascicle from the radial nerve to a branch for the triceps to recover elbow extension in partial brachial plexus palsy	Flores	2017
**115**	Successful nerve transfers for traumatic brachial plexus palsy in a septuagenarian	Johnsen	2016
**116**	Free functioning gracilis muscle transfer for elbow flexion reconstruction after traumatic brachial pan-plexus injury: Where is the optimal distal tendon attachment for elbow flexion?	Maldonado	2017(b)
**117**	Results of distal nerve transfers in restoration of shoulder function in C5 and C6 root avulsion injury to the brachial plexus	Bhandari	2017
**118**	Bipolar dual-lead spinal cord stimulation between two electrodes on the ventral and dorsal sides of the spinal cord: consideration of putative mechanisms	Watanabe	2018
**119**	Triceps nerve to deltoid nerve transfer after an unsatisfactory intra-plexus neurotisation of the posterior division of the upper trunk	Al-Qattan	2017
**120**	Trapezius muscle transfer for restoration of elbow extension in a traumatic brachial plexus injury	Alrabai	2018
**121**	Transfer of the radial nerve branch to the extensor carpi radialis brevis to the anterior interosseous nerve to reconstruct thumb and finger flexion	Bertelli	2015

Article title: Developing a core outcome set for Traumatic Brachial Plexus Injuries: a systematic review of outcomes

For peer review only - http://bmjopen.bmj.com/site/about/guidelines.xhtml

Supplementary file 5. Included Studies

**122**	Ultrasound-guided pulse-dose radiofrequency: treatment of neuropathic pain after brachial plexus lesion and arm vascularisation	Magistroni	2014
**123**	Phrenic nerve transfer to the musculocutaneous nerve for the repair of brachial plexus injury: electrophysiological characteristics	Liu	2015
**124**	Postoperative motor deficits following elbow flexion reanimation by nerve transfer	Hanneur	2018
**125**	Comparative study of phrenic and partial ulnar nerve transfers for elbow flexion after upper brachial plexus avulsion-a retrospective clinical analysis	Liu	2018
**126**	Contralateral medial pectoral nerve transfer with free gracilis muscle transfer in old brachial plexus injury	Yavari	2018
**127**	MEG-BMI to control phantom limb pain	Yanagisawa	2018
**128**	Complete brachial plexus injury- an amputation dilemma, A case report	Choong	2015
**129**	Reversal of phantom pain and hand-to-face remapping after brachial plexus avulsion	Tsao	2016
**130**	A newly developed upper limb single-joint HAL in a patient with elbow flexion reconstruction after traumatic brachial plexus injury: A case report	Kubota	2017
**131**	Free reverse gracilis muscle combined with steindler flexorplasty for elbow flexion reconstruction after failed primary repair of extended upper-type paralysis of the brachial plexus	Bertelli	2018
**132**	Multiple nerve and tendon transfers – a new strategy for restoring hand function in a patient with C7-T1 brachial plexus avulsions	Xu	2017
**Studies included following updated review May 2021**			
**133**	Outcomes after occupational therapy intervention for traumatic brachial plexus injury: A prospective longitudinal cohort study	Cole	2020
**134**	Lower trapezius transfer for patients with brachial plexus injury	Crepaldi	2019
**135**	Bionic upper limb reconstruction: A valuable alternative in global brachial plexus avulsion injuries—a case series	Hruby	2019
**136**	Transcranial Direct Current Stimulation and mirror therapy for neuropathic pain after brachial plexus avulsion: A randomised double-blind, controlled pilot study	Ferreira	2020
**137**	A comparative study of two modalities in pain management of patients presenting with chronic brachial neuralgia	Razak	2019
**138**	Do technical components of microanastomoses influence the functional outcome of free gracilis muscle transfer for elbow flexion in traumatic brachial plexus injury	Martins-Filho	2021

Article title: Developing a core outcome set for Traumatic Brachial Plexus Injuries: a systematic review of outcomes

For peer review only - http://bmjopen.bmj.com/site/about/guidelines.xhtml

Supplementary file 5. Included Studies

References

[1] Limthongthang R, Muennoi P, Phoojaroenchanachai R et al. Effectiveness and safety of home-based muscle electrical stimulator in brachial plexus injury patients. *J Med Assoc Thail* 2014;97:S56–61.

[2] Homsreprasert T, Limthongthang R, Vathana T et al. Elbow joint proprioceptive sense in total arm-type brachial plexus injured patients after neurotization: a preliminary study. *J Med Assoc Thail* 2014;97:S103–7.

[3] Souza F, Bernardino S, Filho H et al. Comparison between the anterior and posterior approach for transfer of the spinal accessory nerve to the suprascapular nerve in late traumatic brachial plexus injuries. *Acta Neurochir (Wien)* 2014;156: 2345–9.

[4] Kim JH, Shin SH, Lee YR et al. Ultrasound-guided peripheral nerve stimulation for neuropathic pain after brachial plexus injury: two case reports. *J Anesth* 2017; 31:453–7.

[5] Satbhai NG, Doi K, Hattori Y, Sakamoto S et al. Contralateral lower trapezius transfer for restoration of shoulder external rotation in traumatic brachial plexus palsy: a preliminary report and literature review. *J Hand Surg Eur* 2014;39:861–7.

[6] Huan KWSJ, Tan JSW, Tan SH et al. Restoration of shoulder abduction in brachial plexus avulsion injuries with double neurotization from the spinal accessory nerve: a report of 13 cases. *J Hand Surg Eur* 2017;42:700–5.

[7] Bertelli JA, Soldado F, Ghizoni MF et al. Transfer of the musculocutaneous nerve branch to the brachialis muscle to the triceps for elbow extension: anatomical study and report of five cases. *J Hand Surg Eur* 2017;42:710–4.

[8] Rui J, Zhao X, Zhu Y et al. Posterior approach for accessory-suprascapular nerve transfer: an electrophysiological outcomes study. *J Hand Surg Eur* 2013;38:242–7.

[9] Potter SM, Ferris SI. Reliability of functioning free muscle transfer and vascularized ulnar nerve grafting for elbow flexion in complete brachial plexus palsy. *J Hand Surg Eur* 2017; 42: 693–9.

[10] Lam WL, Fufa D, Chang N-J et al. Management of infraclavicular (Chuang Level IV) brachial plexus injuries: A single surgeon experience with 75 cases. *J Hand Surg Eur* 2015; 40:573–82.

[11] Estrella EP, Montales TD. Functioning free muscle transfer for the restoration of elbow flexion in brachial plexus injury patients. *Injury* 2016;47:2525–33.

[12] Desai MJ, Daly CA, Seiler JG et al. Radial to Axillary Nerve Transfers: A Combined Case Series. *J Hand Surg Am* 2016; 41:1128–34.

[13] Pereira EAC, Boccard SG, Linhares P et al. Thalamic deep brain stimulation for neuropathic pain after amputation or brachial plexus avulsion. *Neurosurg Focus* 2013;35:E7.

[14] Estrella EP, Favila AS . Nerve transfers for shoulder function for traumatic brachial plexus injuries. *J Reconstr Microsurg* 2014;30:59-64.

[15] Gutkowska O, Martynkiewicz J, Mizia S et al. Results of Operative Treatment of Brachial Plexus Injury Resulting from Shoulder Dislocation: A Study with A Long-Term Follow-Up. *World Neurosurg* 2017;105:623–31.

[16] Oberlin C, Chino J, Belkheyar Z. Surgical treatment of brachial plexus posterior cord lesion: A combination of nerve and tendon transfers, about nine patients. *Chir Main* 2013;32:141–6.

Article title: Developing a core outcome set for Traumatic Brachial Plexus Injuries: a systematic review of outcomes

For peer review only - http://bmjopen.bmj.com/site/about/guidelines.xhtml

Supplementary file 5. Included Studies

[17] Ferraresi S, Garozzo D, Basso E et al. The medial cord to musculocutaneous (MCMc) nerve transfer: A new method to reanimate elbow flexion after C5-C6-C7-(C8) avulsive injuries of the brachial plexus - Technique and results. *Neurosurg Rev* 2014;37:321–9.

[18] Bertelli J, Soldado F, Ghizoni MF et al. Transfer of a terminal motor branch nerve to the flexor carpi ulnaris for triceps reinnervation: Anatomical study and clinical cases. *J Hand Surg Am* 2015;40:2229-2235.

[19] Maldonado AA, Kircher MF, Spinner RJ et al. Free Functioning Gracilis Muscle Transfer With and Without Simultaneous Intercostal Nerve Transfer to Musculocutaneous Nerve for Restoration of Elbow Flexion After Traumatic Adult Brachial Pan-Plexus Injury. *J Hand Surg Am* 2017;42:293.

[20] Ghosh S, Singh VK, Jeyaseelan L, et al. Isolated latissimus dorsi transfer to restore shoulder external rotation in adults with brachial plexus injury. *Bone Jt J* 2013;95:660–3.

[21] Satbhai NG, Doi K, Hattori Y et al. Functional outcome and quality of life after traumatic total brachial plexus injury treated by nerve transfer or single/double free muscle transfers: A comparative study. *Bone Jt J* 2016;98:209–17.

[22] Mibu A, Nishigami T, Tanaka K et al. Successful Graded Mirror Therapy in a Patient with Chronic Deafferentation Pain in Whom Traditional Mirror Therapy was Ineffective: A Case Report. *Pain Pract* 2016;16:E62–9.

[23] Azab AA-H, Alsabbahi MS. Bipolar Transfer of Latissimus Dorsi Myocutaneous Flap for Restoration of Elbow Flexion in Late Traumatic Brachial Plexus Injury: Evaluation of 13 Cases. *Ann Plast Surg* 2017;78:98–201.

[24] Tsai YJ, Su FC, Hsiao CK et al. Comparison of objective muscle strength in C5-C6 and C5-C7 brachial plexus injury patients after double nerve transfer. *Microsurgery* 2015;35:107–14.

[25] Son BC, Ha SW. Phantom Remodeling Effect of Dorsal Root Entry Zone Lesioning in Phantom Limb Pain Caused by Brachial Plexus Avulsion. *Stereotact Funct Neurosurg* 2015;93:240–4.

[26] Hu CH, Chang TN, Lu JC et al. Comparison of Surgical Strategies between Proximal Nerve Graft and/or Nerve Transfer and Distal Nerve Transfer Based on Functional Restoration of Elbow Flexion: A Retrospective Review of 147 Patients. *Plast Reconstr Surg* 2018;141:68e-79e.

[27] Ren G, Li R, Xiang D, Yu B. Reconstruction of shoulder abduction by multiple nerve fascicle transfer through posterior approach. *Injury* 2013;44:492–7.

[28] Xiao C, Lao J, Wang T et al. Intercostal nerve transfer to neurotize the musculocutaneous nerve after traumatic brachial plexus avulsion: a comparison of two, three, and four nerve transfers. *J Reconstr Microsurg* 2014;30:297–304.

[29] Resnik L, Fantini C, Latlief G et al. Use of the DEKA Arm for amputees with brachial plexus injury: A case series. *PLoS One* 2017; 12: e0178642.

[30] Leechavengvongs S, Jiamton C, Uerpairojkit C et al. Polyester tape scapulopexy for chronic upper extremity brachial plexus injury. *J Hand Surg Am* 2015;40:1184

[31] Wang S, Li P, Xue Y et al. Contralateral C7 nerve transfer with direct coaptation to restore lower trunk function after traumatic brachial plexus avulsion. *J Bone Joint Surg Am* 2013;95: 821–2.

[32] Dodakundi C, Doi K, Hattori Y et al. Outcome of surgical reconstruction after traumatic total brachial plexus palsy. *J Bone Joint Surg Am* 2013;95:1505–12.

For peer review only - http://bmjopen.bmj.com/site/about/guidelines.xhtml

Supplementary file 5. Included Studies

[33] Aszmann OC, Roche AD, Salminger S et al. Bionic reconstruction to restore hand function after brachial plexus injury: a case series of three patients. *Lancet* 2015; 385:2183–9.

[34] Tsymbaliuk VI, Tretiak IB. [Surgical treatment of the plexus brachialis injury using long-lasting electrostimulation]. *Klin Khirurhiia* 2013;59–61.

[35] Flores LP, Socolovsky M. Phrenic Nerve Transfer for Reconstruction of Elbow Extension in Severe Brachial Plexus Injuries. *J Reconstr Microsurg* 2016;32:546–50.

[36] Wang S, Li P, Xue Y et al. Direct Coaptation of the Phrenic Nerve With the Posterior Division of the Lower Trunk to Restore Finger and Elbow Extension Function in Patients With Total Brachial Plexus Injuries. *Neurosurgery* 2016;78:208–14.

[37] Martins RS, Siqueira MG, Heise CO et al. A prospective study comparing single and double fascicular transfer to restore elbow flexion after brachial plexus injury. *Neurosurgery* 2013; 72:709–15.

[38] Stevanato G, Devigili G, Eleopra R et al. Chronic post-traumatic neuropathic pain of brachial plexus and upper limb: a new technique of peripheral nerve stimulation. *Neurosurg Rev* 2014;37:473–9.

[39] Wei W, Meihua S, Yafei L et al. [EFFECTIVENESS OF CONTRALATERAL C7 NERVE ROOT AND MULTIPLE NERVES TRANSFER FOR TREATMENT OF BRACHIAL PLEXUS ROOT AVULSION]. *Zhongguo Xiu Fu Chong Jian Wai Ke Za Zhi* 2014;28:737–40.

[40] Plate JF, Ely LK, Pulley BR et al. Combined proximal nerve graft and distal nerve transfer for a posterior cord brachial plexus injury. *J Neurosurg* 2013;118: 155–9.

[41] Maldonado AA, Kircher MF, Spinner RJ et al. The role of elective amputation in patients with traumatic brachial plexus injury. *J Plast Reconstr Aesthetic Surg* 2016;69:311–7.

[42] Liu Y, Wang W, Regmi AM et al. Early microsurgical management of clavicular fracture combined with brachial plexus injury. *Zhongguo Xiu Fu Chong Jian Wai Ke Za Zhi* 2014;28:1329–32.

[43] Elhassan BT, Wagner ER, Spinner RJ et al. Contralateral trapezius transfer to restore shoulder external rotation following adult brachial plexus injury. *J Hand Surg Am* 2016;41:e45-51.

[44] Liu Y, Lao J, Gao K et al. Comparative study of phrenic nerve transfers with and without nerve graft for elbow flexion after global brachial plexus injury. *Injury* 2014;45:227–31.

[45] Wang JP, Rancy SK, Lee SK et al. Shoulder and Elbow Recovery at 2 and 11 Years Following Brachial Plexus Reconstruction. *J Hand Surg Am* 2016;41:173–9.

[46] Aras Y, Aydoseli A, Sabanci PA et al. Functional outcomes after treatment of traumatic brachial plexus injuries: clinical study. *Ulus Travma Acil Cerrahi Derg* 2013;19:521–8.

[47] Soldado F, Bertelli J. Free gracilis transfer reinnervated by the nerve to the supinator for the reconstruction of finger and thumb extension in longstanding C7-T1 brachial plexus root avulsion. *J Hand Surg Am* 2013;38: 941–6.

[48] Zhang CG, Dong Z, Gu YD. Restoration of hand function in C7-T1 brachial plexus palsies using a staged approach with nerve and tendon transfer. *J Neurosurg* 2014;121:1264–70.

[49] Dy CJ, Kitay A, Garg R et al. Neurotization to innervate the deltoid and biceps: 3 Cases. *J Hand Surg Am* 2013;38:237–40.

[50] Lenoir H, Williams T, Griffart A et al. Arthroscopic arthrodesis of the shoulder in brachial

For peer review only - http://bmjopen.bmj.com/site/about/guidelines.xhtml

Supplementary file 5. Included Studies

plexus palsy. *J Shoulder Elb Surg* 2017; 26: e115–21.

[51] Gao K, Lao J, Zhao X et al. Outcome of contralateral C7 nerve transferring to median nerve. *Chin Med J (Engl)* 2013;126:3865–8.

[52] Cho AB, Iamaguchi RB, Silva GB et al. Intercostal nerve transfer to the biceps motor branch in complete traumatic brachial plexus injuries. *Microsurgery* 2015;35:428–31.

[53] Sano Y, Wake N, Ichinose A et al. Tactile feedback for relief of deafferentation pain using virtual reality system: a pilot study. *J Neuroeng Rehabil* 2016;13:61.

[54] Yang Y, Yang JT, Fu G et al. Functioning free gracilis transfer to reconstruct elbow flexion and quality of life in global brachial plexus injured patients. *Sci Rep* 2016;6:22479.

[55] Baltzer HL, Wagner ER, Kircher MF, et al. Evaluation of infraspinatus reinnervation and function following spinal accessory nerve to suprascapular nerve transfer in adult traumatic brachial plexus injuries. *Microsurgery* 2017;37:365–70.

[56] Hu S, Chu B, Song J, et al. Anatomic study of the intercostal nerve transfer to the suprascapular nerve and a case report. *J Hand Surg Eur* 2014;39:194–8.

[57] Kostas-Agnantis I, Korompilias A, Vekris M et al. Shoulder abduction and external rotation restoration with nerve transfer. *Injury* 2013;44:299–304.

[58] Bhatia A, Doshi P, Koul A, et al. Contralateral C-7 transfer: is direct repair really superior to grafting? *Neurosurg Focus* 2017;43:E3.

[59] Kita Y, Tajiri Y, Hoshikawa S et al. Impact of phrenic nerve paralysis on the surgical outcome of intercostal nerve transfer. *Hand Surg* 2015; 20:47–52.

[60] Hou Y, Yang J, Yang Y, et al. Flow-through anastomosis using a T-shaped vascular pedicle for gracilis functioning free muscle transplantation in brachial plexus injury. *Clinics (Sao Paulo)* 2015;70:544–9.

[61] Sechachalam S, O'Byrne A, MacQuillan A. Free Functional Muscle Transfer Tendon Insertion Secondary Advancement Procedure to Improve Elbow Flexion. *Tech Hand Up Extrem Surg* 2017;21: 8–12.

[62] Chu B, Wang H, Chen L, et al. Dual Nerve Transfers for Restoration of Shoulder Function After Brachial Plexus Avulsion Injury. *Ann Plast Surg* 2016;76: 668–73.

[63] Bhat DI, Indira Devi B, Bharti K, et al. Cortical plasticity after brachial plexus injury and repair: a resting-state functional MRI study. *Neurosurg Focus* 2017;42: E14.

[64] Bertelli JA, Ghizoni MF. Results of spinal accessory to suprascapular nerve transfer in 110 patients with complete palsy of the brachial plexus. *J Neurosurg Spine* 2016;24:990–5.

[65] Frueh FS, Ho M, Schiller A, et al. Magnetic Resonance Neurographic and Clinical Long-Term Results After Oberlin's Transfer for Adult Brachial Plexus Injuries. *Ann Plast Surg* 2017;78:67–72.

[66] Maldonado AA, Kircher MF, Spinner RJ,et al. Free Functioning Gracilis Muscle Transfer versus Intercostal Nerve Transfer to Musculocutaneous Nerve for Restoration of Elbow Flexion after Traumatic Adult Brachial Pan-Plexus Injury. *Plast Reconstr Surg* 2016;138:483e-488e.

[67] Bertelli JA, Ghizoni MF, Tacca CP. Results of wrist extension reconstruction in C5-8 brachial plexus palsy by transferring the pronator quadratus motor branch to the extensor carpi radialis brevis muscle. *J Neurosurg* 2016;124:1442–9.

Article title: Developing a core outcome set for Traumatic Brachial Plexus Injuries: a systematic review of outcomes

For peer review only - http://bmjopen.bmj.com/site/about/guidelines.xhtml

Supplementary file 5. Included Studies

[68] Nicoson MC, Franco MJ, Tung TH. Donor nerve sources in free functional gracilis muscle transfer for elbow flexion in adult brachial plexus injury. *Microsurgery* 2017;37:377–82.

[69] Bhandari PS, Deb P. Use of contralateral spinal accessory nerve for ipsilateral suprascapular neurotization in global brachial plexus injury: A new technique. *J Neurosurg Spine* 2016;24: 186–8.

[70] Maricq C, Jeunehomme M, Mouraux D, et al. Objective evaluation of elbow flexion strength and fatigability after nerve transfer in adult traumatic upper brachial plexus injuries. *Hand Surg* 2014;19:335–41.

[71] DeGeorge B, Becker H, Faryna J,et al. Outcomes of brachialis muscle transfer to restore finger flexion in traumatic lower trunk brachial plexus palsy. *J Hand Surg Am* 2017;42:S33–4.

[72] Liu Y, Lao J, Gao K, et al. Functional outcome of nerve transfers for traumatic global brachial plexus avulsion. *Injury* 2013;44:655–60.

[73] Bertelli JA, Ghizoni MF. Transfer of a flexor digitorum superficialis motor branch for wrist extension reconstruction in C5-C8 root injuries of the brachial plexus: a case series. *Microsurgery* 2013;33:39–42.

[74] Gao K, Lao J, Zhao X,et al. Outcome after transfer of intercostal nerves to the nerve of triceps long head in 25 adult patients with total brachial plexus root avulsion injury. *J Neurosurg* 2013;118:606–10.

[75] Foroni L, Siqueira MG, Martins RS, et al. Good sensory recovery of the hand in brachial plexus surgery using the intercostobrachial nerve as the donor. *Arq Neuropsiquiatr* 2017;75:796–800.

[76] Socolovsky M, di Masi G, Bonilla G, et al. The phrenic nerve as a donor for brachial plexus injuries: is it safe and effective? Case series and literature analysis. *Acta Neurochir (Wien)* 2015;157:1077–86.

[77] Hattori Y, Doi K, Sakamoto S,et al. Complete avulsion of brachial plexus with associated vascular trauma: feasibility of reconstruction using the double free muscle technique. *Plast Reconstr Surg* 2013;132:1504–12.

[78] Kachramanoglou C, Carlstedt T, Koltzenburg M, et al. Long-Term Outcome of Brachial Plexus Reimplantation After Complete Brachial Plexus Avulsion Injury. *World Neurosurg* 2017;103:28–36.

[79] Kodama N, Doi K, Hattori Y. Force recovery assessment of functioning free muscle transfers using ultrasonography. *J Hand Surg Am* 2014;39:2269–76.

[80] Goubier J, Teboul F. Rhomboid nerve transfer to the suprascapular nerve for shoulder reanimation in brachial plexus palsy: A clinical report. *Hand Surg Rehabil* 2016;35:363–6.

[81] Gao K, Lao J, Zhao X,et al. Outcome of contralateral C7 transfer to two recipient nerves in 22 patients with the total brachial plexus avulsion injury. *Microsurgery* 2013;33:605–11.

[82] Liu Y, Lao J, Zhao X. Comparative study of phrenic and intercostal nerve transfers for elbow flexion after global brachial plexus injury. *Injury* 2015;46:671–5.

[83] Li XM, Yang JT, Hou Y, et al. Donor-side morbidity after contralateral C-7 nerve transfer: results at a minimum of 6 months after surgery. *J Neurosurg* 2016;124:1434–41

[84] Rasulic L, Savic A, Zivkovic B, et al. Outcome after brachial plexus injury surgery and impact on

Article title: Developing a core outcome set for Traumatic Brachial Plexus Injuries: a systematic review of outcomes

For peer review only - http://bmjopen.bmj.com/site/about/guidelines.xhtml

Supplementary file 5. Included Studies

quality of life. *Acta Neurochir (Wien)* 2017;159:1257–64

[85] Yang J, Jia X, Yu C, et al. Pronator teres branch transfer to the anterior interosseous nerve for treating C8T1 brachial plexus avulsion: An anatomic study and case report. *Neurosurgery* 2014;75:375–9.

[86] Stiasny J, Birkeland P, J. S. Operative treatment with nerve repair can restore function in patients with traction injuries in the brachial plexus. *Dan Med J* 2015;62.

[87] Soldado F, Ghizoni MF, Bertelli J, et al. Thoracodorsal nerve transfer for triceps reinnervation in partial brachial plexus injuries. *Microsurgery* 2016;36:191–7.

[88] Thakkar UG, Vanikar A V, Trivedi HL. Co-infusion of autologous adipose tissue derived neuronal differentiated mesenchymal stem cells and bone marrow derived hematopoietic stem cells, a viable therapy for post-traumatic brachial plexus injury: a case report. *Biomed J* 2014;37:237–40.

[89] Emamhadi M, Alijani B, Andalib S,et al. Long-term clinical outcomes of spinal accessory nerve transfer to the suprascapular nerve in patients with brachial plexus palsy. *Acta Neurochir (Wien)* 2016;158:1801–6.

[90] Tu YK, Tsai YJ, Chang CH et al. Surgical treatment for total root avulsion type brachial plexus injuries by neurotization: a prospective comparison study between total and hemicontralateral C7 nerve root transfer. *Microsurgery* 2014;34:91–101.

[91] Qiu YQ, Hua XY, Zuo CT et al. Deactivation of distant pain-related regions induced by 20-day rTMS: a case study of one-week pain relief for long-term intractable deafferentation pain. *Pain Physician* 2014;17:E99–105.

[92] Haninec P, Mencl L, Kaiser R. End-to-side neurorrhaphy in brachial plexus reconstruction. *J Neurosurg* 2013;119:689–94.

[93] Flores LP. Reanimation of elbow extension with medial pectoral nerve transfer in partial injuries to the brachial plexus. *J Neurosurg* 2013;118:588–93.

[94] Mohammad-Reda A. Early post-operative results after repair of traumatic brachial plexus palsy. *Turk Neurosurg* 2013;23:1–9.

[95] van der Lingen MAJ, de Joode SGCJ, Schotanus MGM, et al. Satisfied patients after shoulder arthrodesis for brachial plexus lesions even after 20 years of follow-up. *Eur J Orthop Surg Traumatol* 2018;28:1089–94

[96] Klika BJ, Spinner RJ, Bishop AT et al. Posterior branch of the axillary nerve transfer to the lateral triceps branch for restoration of elbow extension: case report. *J Hand Surg Am* 2013;38:1145–9.

[97] Liu J, Wang X, Zhang S, et al. A clinical analysis of repairing the whole brachial plexus nerve root avulsion by transferring C7 nerve root from the uninjured side. *J Neurol Sci* 2014;31:521–31.

[98] Cambon-Binder A, Walch A, Marchei P et al. Bipolar transfer of the pectoralis major muscle for restoration of elbow flexion in 29 cases. *J Shoulder Elb Surg* 2018;27:e330-e336

[99] Soldado F, Ghizoni MF, Bertelli J. Thoracodorsal nerve transfer for elbow flexion reconstruction in infraclavicular brachial plexus injuries. *J Hand Surg Am* 2014;39:1766–70.

[100] Cho A, Paulos R, De Resende M, et al. Median nerve fascicle transfer versus ulnar nerve

Article title: Developing a core outcome set for Traumatic Brachial Plexus Injuries: a systematic review of outcomes

For peer review only - http://bmjopen.bmj.com/site/about/guidelines.xhtml

Supplementary file 5. Included Studies

fascicle transfer to the biceps motor branch in C5-C6 and C5-C7 brachial plexus injuries: nonrandomized prospective study of 23 consecutive patients. *Microsurgery* 2014;34:511–5.

[101] Kaizawa Y, Kakinoki R, Ohta S,et al. Free functional muscle transplantation of an anomalous femoral adductor with a very large muscle belly: A case report. *J Brachial Plex Peripher Nerve* Inj 2013;8:11.

[102] Tuohuti T, Yu Q, Yang J,et al. Selective neurotization of the radial nerve in the axilla using intercostal nerve to treat complete brachial plexus palsy. *Int J Clin Exp Med* 2016;9:22880–5.

[103] Flores LP. Objective Predictors of Functional Recovery Associated with Intercostal Nerves Transfer for Triceps Reinnervation in Global Brachial Plexus Palsy. *Brazilian Neurosurg* 2016;35:271–8.

[104] Emamhadi M. Nerve transfer to relieve pain in upper brachial plexus injuries: Does it work? *Clin Neurol Neurosurg* 2017;163:67–70.

[105] Abdixbir A, Li P, Ilhamjan U, et al. Phrenic nerve transfer versus intercostal nerve transfer for the repair of brachial plexus root avulsion injuries. *Chinese J Tissue Eng Res* 2016;20:7660–5.

[106] Limthongthang R, Vathana T, Wongtrakul S,et al. End-to-Side Neurorrhaphy to Restore Elbow Flexion in Brachial Plexus Injury. *J Med Assoc Thai* 2016;99:1203–8.

[107] De Oliveira C, Da Silva Melo D, et al. Chordata method combined with electrotherapy in functional recovery after brachial plexus injury: Report of three clinical cases. *Sci Med (Porto Alegre)* 2016;26:22425.

[108] Xu B, Dong Z, Zhang CG,et al. Clinical outcome following transfer of the supinator motor branch to the posterior interosseous nerve in patients with C7-T1 brachial plexus palsy. *J Reconstr Microsurg* 2015;31:102–6.

[109] Wu X, Cong XB, Huang QS, et al. Transposition of branches of radial nerve innervating supinator to posterior interosseous nerve for functional reconstruction of finger and thumb extension in 4 patients with middle and lower trunk root avulsion injuries of brachial plexus. *J Huazhong Univ Sci Technolog Med Sci* 2017;37:933–7.

[110] Kazamel M, Sorenson EJ. Electromyographic Findings in Gracilis Muscle Grafts Used to Augment Elbow Flexion in Traumatic Brachial Plexopathy. *J Clin Neurophysiol* 2016;33:549–53.

[111] Li Z, Reynolds M, Satteson E,et al. Double Distal Intraneural Fascicular Nerve Transfers for Lower Brachial Plexus Injuries. *J Hand Surg Am* 2016;41:e15-9.

[112] Arnal M, Cambon A, Marcheix P. Restoration of elbow and hand function in total brachial plexus palsy with intercostal nerves and C5 root neurotization. Results in 21 patients. *Hand Surg Rehabil* 2016;35:283–7.

[113] Jiang Y, Lao J. The phrenic nerve transfer in the treatment of a septuagenarian with brachial plexus avulsion injury: a case report. *Int J Neurosci* 2018;128:467–71.

[114] Flores LP. Outcomes of Transferring a Healthy Motor Fascicle From the Radial Nerve to a Branch for the Triceps to Recover Elbow Extension in Partial Brachial Plexus Palsy. *Neurosurgery* 2017;80:448–53.

[115] Johnsen PH, Wolfe SW. Successful Nerve Transfers for Traumatic Brachial Plexus Palsy in a Septuagenarian: A Case Report. *Hand (N Y)* 2016;11:NP30–3.

For peer review only - http://bmjopen.bmj.com/site/about/guidelines.xhtml

Supplementary file 5. Included Studies

[116] Maldonado AA, Romero-Brufau S, Kircher MF, et al. Free Functioning Gracilis Muscle Transfer for Elbow Flexion Reconstruction after Traumatic Adult Brachial Pan-Plexus Injury: Where Is the Optimal Distal Tendon Attachment for Elbow Flexion? *Plast Reconstr Surg* 2017;139: 128–36.

[117] Bhandari PS. Results of Distal Nerve Transfers in Restoration of Shoulder Function in C5 and C6 Root Avulsion Injury to the Brachial Plexus. *Indian J Neurotrauma* 2017;14:21–5.

[118] Watanabe M, Yamamoto T, Fukaya C, et al. Bipolar dual-lead spinal cord stimulation between two electrodes on the ventral and dorsal sides of the spinal cord: consideration of putative mechanisms. *Acta Neurochir (Wien)* 2018;160:639–43.

[119] Al-Qattan M, Kattan A, Al-Qahtany B et al. Triceps nerve to deltoid nerve transfer after an unsatisfactory intra-plexus neurotization of the posterior division of the upper trunk. *Int J Surg Case Rep* 2017;37:124–6.

[120] Alrabai H, Gesheff G, Hammouda A,et al. Trapezius Muscle Transfer for Restoration of Elbow Extension in a Traumatic Brachial Plexus Injury. *J Hand Surg Am* 2018;43:872.

[121] Bertelli JA. Transfer of the radial nerve branch to the extensor carpi radialis brevis to the anterior interosseous nerve to reconstruct thumb and finger flexion. *J Hand Surg Am* 2015;40:323-328.

[122] Magistroni E, Ciclamini D, Panero B. Ultrasound-guided pulse-dose radiofrequency: Treatment of neuropathic pain after brachial plexus lesion and arm revascularization. *Case Rep Med* 2014;201:429618.

[123] Liu Y, Xu X, Zou Y, et al. Phrenic nerve transfer to the musculocutaneous nerve for the repair of brachial plexus injury: Electrophysiological characteristics. *Neural Regen Res* 2015;10:328–33.

[124] Le Hanneur M, Walch A, Gerosa T et al. Postoperative motor deficits following elbow flexion reanimation by nerve transfer. *Hand Surg Rehabil* 2018;37:289-294

[125] Liu Y, Zhuang Y, Yu H,et al. Comparative study of phrenic and partial ulnar nerve transfers for elbow flexion after upper brachial plexus avulsion: A retrospective clinical analysis. *J Plast Reconstr Aesthetic Surg* 2018;71:1245–51.

[126] Yavari M, Mahmoudvand H, Nadri S. Contralateral medial pectoral nerve transfer with free gracilis muscle transfer in old brachial plexus palsy. *J Surg Res* 2018;231:94–8.

[127] Yanagisawa T, Fukuma R, Seymour B, et al. MEG-BMI to control phantom limb pain. *Neurol Med Chir (Tokyo)* 2018;58:327–33.

[128] Choong C, Shalimar A. Complete brachial plexus injury - An amputation dilemma. A case report. *Malaysian Orthop J* 2015;9:52–4.

[129] Tsao J, Finn S. Reversal of phantom pain and hand-to-face remapping after brachial plexus avulsion. *Ann Clin Transl Neurol* 2016;3:463–4..

[130] Kubota S, Hara Y, Shimizu Y, et al. A newly developed upper limb single-joint HAL in a patient with elbow flexion reconstruction after traumatic brachial plexus injury: A case report. *Interdiscip Neurosurg Adv Tech Case Manag* 2017;10:66–8.

[131] Bertelli JA. Free Reverse Gracilis Muscle Combined With Steindler Flexorplasty for Elbow Flexion Reconstruction After Failed Primary Repair of Extended Upper-Type Paralysis of the Brachial Plexus. *J Hand Surg Am* 2019;44:112-120

Article title: Developing a core outcome set for Traumatic Brachial Plexus Injuries: a systematic review of outcomes

For peer review only - http://bmjopen.bmj.com/site/about/guidelines.xhtml

Supplementary file 5. Included Studies

[132] Xu B, Dong Z, Zhang C. Multiple nerve and tendon transfers: A new strategy for restoring hand function in a patient with C7-T1 brachial plexus avulsions. *J Neurosurg* 2017;127:837–42.

[133] Cole T, Nicks R, Ferris S et al. Outcomes after occupational therapy intervention for traumatic brachial plexus injury: A prospective longitudinal cohort study. *Journal of Hand Therapy*. 2020;33:528-539

[134] Crepaldi BE, Neto JQL, Rezende MR et al. *Hand*.2019;14(2):179-186

[135] Hruby LA, Gstoettner C, Sturma A et al. *Journal of Clinical Medicine*. 2020;9(23):1-14

[136] Ferreira CM, de Carvalho CD, Gomes R et al. Transcranial direct current stimulation and mirror therapy for neuropathic pain after brachial plexus avulsion: A randomised double-blind, controlled pilot study. Frontiers in *Neurology*. 2020;11:1-10

[137] Razak I, Chung TY, Ahmad S et al. A comparative study of two modalities in pain managementof patients presenting with chronic brachial neuralgia*. Journal of Alternative and Complementary Medicine.* 2019;1-7

[138] Martins-Filho FVF, de Carmo Iwase F, Silva GB et al. Do technical components of microanastomosis influence the functional outcome of free gracilis muscle transfer for elbow flexion in traumatic brachial plexus injury? *Orthopaedics & Traumatology: Surgery & Research.*2021;107:1-5

Article title: Developing a core outcome set for Traumatic Brachial Plexus Injuries: a systematic review of outcomes

For peer review only - http://bmjopen.bmj.com/site/about/guidelines.xhtml

Supplementary file 6: Unique outcomes mapped to potential domains and core areas according to COMET

Online Supplementary file 4. Table: Unique outcomes mapped to potential domains and core areas according to COMET(Dodd et al., 2018)

**Outcomes (n=157)**	**Subdomains**	**Domains**	**Core Areas**
Isometric muscle strength	Muscle strength/ function	Musculoskeletal and connective tissue domain	Physiological/Clinical
Concentric strength			
Eccentric strength			
Muscle flicker/contraction			
Anti-gravity muscle activity			
Muscle endurance			
Muscle fatigue			
Muscle torque			
Active range of movement	Active movement		
Perception of movement			
Antigravity movement			
Independent movement without donor			
Passive range of movement	Passive movement		
Movement control/stability	Control of movement/stability		
Muscle mass	Muscle mass		
Bony union	Bone structure/position		
Joint position			
Joint stability			
General sensory recovery	General sensory recovery	Nervous system	
Feeling of numbness			
Proprioception			
Light touch	Discriminative touch		
2 PD			

For peer review only

Article title: Developing a core outcome set for Traumatic Brachial Plexus Injuries: a systematic review of outcomes

For peer review only - http://bmjopen.bmj.com/site/about/guidelines.xhtml

Supplementary file 6: Unique outcomes mapped to potential domains and core areas according to COMET

Vibration			
Object recognition			
Pain	Protective touch		
Temperature			
Deep pressure			
Brachial plexus structure	Peripheral nervous system structure		
Level of reinnervation	Reinnervation		
Time to reinnervation			
Progression of regeneration	Progression of regeneration		
Speed of motor sensory conduction	Speed of motor and sensory conduction		
Pain intensity	Pain intensity/relief	General outcomes/symptoms	
Pain relief / reduction			
Pain duration	Pain duration/frequency		
Pain frequency			
Pain quality	Pain quality and interference with life		
Pain interference with walking			
Pain interference in mood			
Pain interference with work			
Pain interference in activities of daily living			
Pain interference with relationships			
Pain interference with enjoyment of life			
Pain interference with sleep			
Sensitivity to cold	Pain when arm exposed to cold		
Paraesthesia	Paraesthesia and itchiness		
Itchiness			

For peer review only

For peer review only - http://bmjopen.bmj.com/site/about/guidelines.xhtml

Supplementary file 6: Unique outcomes mapped to potential domains and core areas according to COMET

Sensitivity to pressure	Sensitivity to touch, pressure etc		
Sensitivity to touch			
Pain location	Location of pain		
Pain relief from medication	Pain medication use		
Stiffness	Stiffness		
Impact on general sleep	Impact on sleep		
Impact on sleep on affected side			
Frequency sleep disturbed by injury			
General physical function	Physical function non-specific	Physical functioning	Life Impact
Patient led functional outcome			
Walking short distance	Lower limb and non -upper limb function		
Balance			
Running			
Climbing stairs			
Bending			
Kneeling			
Reaching	Reaching, pulling, pushing, carrying etc		
Pulling			
Pushing			
Carrying			
Throwing			
Lifting			
General function of arm			
Turning and twisting arm	Turning twisting, gripping and releasing with the arm		
Grip and release			
Pinching	Fine hand movement including writing		
Fine hand movement (writing/buttons)			
Returning to work	Impact on paid or unpaid work or role in education	Role functioning	
Ability to do work			
Usual time at work			
Type of work			

For peer review only

For peer review only - http://bmjopen.bmj.com/site/about/guidelines.xhtml

Supplementary file 6: Unique outcomes mapped to potential domains and core areas according to COMET

Usual school activities			
General rating to perform a patient specific activity	Role function - patient specific		
Impact on ADL (general)	Carrying out daily routine, (including food preparation, housework, garden, plants)		
Return to ADL (general)			
Impact on food preparation and feeding			
Housework (washing, cleaning, ironing, folding, vacuuming)			
Gardening (Includes indoor plants)			
Using a phone			
Maintaining personal hygiene			
Maintaining personal appearance (grooming hair)	Maintaining personal hygiene		
Dressing	Maintaining personal appearance		
Transport needs (e.g driving)	Dressing		
Impact on normal hobbies	Transport needs		
Time doing normal hobbies	Impact on recreational activities and sport		
Playing instrument in usual way			
Ability to play instrument			
Impact on time spent playing instrument			
Impact on time spent doing sport			
Impact on participation in sport			
Social activities with friends	Effect on relationship with	Social functioning	

For peer review only

For peer review only - http://bmjopen.bmj.com/site/about/guidelines.xhtml

Supplementary file 6: Unique outcomes mapped to potential domains and core areas according to COMET

Social activities with neighbours	family, friends, neighbours and groups		
Social activities with family			
Social activities with groups			
Dependence on family and friends			
Appearance interferes with social activities			
Intimate relationships	Effect on intimate relationships		
Emotional impact on work	Emotional distress/mood	Emotional functioning	
Energy levels			
Emotional impact on ADL			
Happiness			
Impact on life enjoyment / satisfaction			
Emotional impact on relationships			
Anxiety			
Depression			
Acceptance/ Adjustment	Thoughts and beliefs (acceptance, coping)		
Coping with trauma			
Confidence	Self esteem and confidence		
Self esteem			
Body image	Body image		
Quality of life	Quality of Life	Global Quality of Life	Quality of Life
Rating of health	Perceived Health status	Health status	Health status
General patient satisfaction	Patient satisfaction	Delivery of Care	Delivery of Care
Satisfaction with appearance of arm			
Satisfaction with function			
Satisfaction with movement			
Satisfaction with strength			

For peer review only

For peer review only - http://bmjopen.bmj.com/site/about/guidelines.xhtml

Supplementary file 6: Unique outcomes mapped to potential domains and core areas according to COMET

Satisfaction with pain			
Satisfaction with colour			
Satisfaction with shape			
Satisfaction with feeling			
Satisfaction with procedure			
Patient preference	Patient preference		
Quality of intervention	Accessibility, quality and adequacy of intervention		
Time to surgery	Time to surgery		
Operation time	Operation time	Resource Use	Resource Use
Motor morbidity	Donor site morbidity	Adverse Events	Adverse Events
Sensory morbidity			
Pain			
General complications	General complications		
Pneumothorax	Respiratory complications		
Respiratory function			
Respiratory symptoms			
Pneumonia			
Arterial thrombosis	Vascular complications		
Venous thrombosis			
Haematoma			
Venous spasm			
Iatrogenic vascular injury			
Vascularity of flap			
Swelling			
Fracture	Musculoskeletal complications		
Passive range of motion loss			
Co-contraction			
Bowstringing			
Failure of tendon attachment			
Joint Instability			
Scapula crepitus			

For peer review only - http://bmjopen.bmj.com/site/about/guidelines.xhtml

Supplementary file 6: Unique outcomes mapped to potential domains and core areas according to COMET

Infection complications	Infection complications		

Dodd, S. et al. A taxonomy has been developed for outcomes in medical research to help improve knowledge discovery. Journal of clinical epidemiology. 2018, 96: 84–92.

For peer review only

For peer review only - http://bmjopen.bmj.com/site/about/guidelines.xhtml

Supplementary file 7. Measurement instruments mapped to domains

Supplementary file 7. Measurement instruments mapped to domains

56 outcome subdomains in 4 core areas (Physiological/clinical, Life Impact, Resource Use and Adverse events) and within the following COMET domains

Musculoskeletal/connective tissue, Nervous system outcome domain, General outcome and symptom domain, Physical functioning, Role functioning, Emotional functioning, Global quality of life, Perceived health status, Delivery of care, Hospital resources and Adverse Events

**Core Area**	Outcome subdomains	**Measurement type used (N)**				**Measurement instruments used (number of studies)**
PHYSIOLOGICAL /CLINICAL		**Patient reported Outcome**	**Clinician reported Outcome**	**Performance Outcome**	**Not Clear**	
	**Musculoskeletal/connective tissue**					
	Muscle strength	31	131	20	3	DASH (n= 28), UEFI (n=2), MHQ (n=1)<br><br>**Manual Muscle Testing**<br>**Manual muscle testing undefined (n=5)**<br>**MRC muscle grading (n=62, including UCLA)**<br>**MRC muscle grading modified (n= 24)**<br>*MRC modified*, unclear how (n= 6)<br>*MRC modified,* grade 3 active must equal passive (n=2)<br>*MRC modified* , grade 2 active must equal passive movement (n=2)<br>*MRC modified,* M3+ contraction with resistance against a finger for less than 30 seconds, M4 contraction of resistance against a finger against a finger for more than 30 seconds (n=1)<br>*MRC modified*: M0, M1+, M1, M1+, M2-, M2, M2+,M3-, M3, M3+, M4-, M4, M4+, M5-, M5 (n=6)<br>*MRC modified,* Finger flexion tested with wrist extended 20-30 degrees (n=1)

Supplementary file 7. Measurement instruments mapped to domains

						MRC modified, Addition of M4.5 (n=1)<br>*MRC modified,* graded two muscles together (n=1)<br>*MRC modified,* finger extension tested with wrist extension at 20-30 degrees (n=1)<br>*MRC modified,* summated muscle score (n=1)<br>*MRC modified,* FDS tested by stabilising LF and IF to table and testing MF and RF IP flexion (n=1)<br>Lovett & Sunderlands standardisation (n=1)<br>**Other manual muscle tests (n=3)**<br>Kendall and McCreary testing procedure (n=1)<br>Oxford muscle testing (n=1)<br>Modification of the Louisiana State University Medical Centre grading system (n=1)<br>**Time to (n= 12)**<br>contraction (n=7); M2 (n=1); strength greater than or equal to M3 (n=1); M 3 (n=1); greater than or equal to modified M3 (n=1); Time to improvement in MRC scale (n= 1)<br>**Dynanometry (n=23)**<br>Dynanometry – isokinetic machine, undefined method (n =1)<br>Grip strength JAMAR , undefined method (n=4); Hook grip – isokinetic machine, undefined method (n=1); Grip strength JAMAR, mean of 3 trials n=2); Grip strength , PABLO system, undefined (n=1); Pinch grip, JAMAR, undefined (n= 3), Pinch grip JAMAR, mean 3 trials (n= 1); Peak isometric, hand held dynamometer (n=2); Isometric strength , hand held dynamometer, best of 3 trials (n=1); Isometric strength , Kendall & Kendall positions, 3 trials mean value (n=1); Measurement on digital scales after 5 seconds (n=1)<br>Concentric strength through range, Isokinetics (n=1)<br>Eccentric strength through range, isokinetics (n=1)<br>Combined action of using elbow and hand on digital hanging scale (n=1)

For peer review only

Supplementary file 7. Measurement instruments mapped to domains

						Constant-Murley score: dynanometry 90 degrees abduction(n=2)<br><br>**Narakas score modified (one study)**<br>Thoaraco brachial grasp (n=1)<br>Elbow flexion with weight (n=1)<br>Elbow extension with weight (n=1)<br>Wrist flexion with weight (n=1)<br>Wrist extension with weight(n=1)<br>Fist power with weight (n=1)<br>Pinch power (n=1)<br><br>**ULM (one study)**<br>Shoulder flexion to shoulder height with 500g (n=1)<br>Shoulder flexion above shoulder height with 500g (n=1)<br>Shoulder flexion above shoulder with 1kg (n=1)<br>Move weight on table (100g) (n=1)<br>Move weight on table (500g) (n=1)<br>Move weight on table (1KG) (n=1)<br><br>**SHAP (two studies)**<br>Grip strength (n=2)<br>Pinch strength (n=2)<br>Pinch grip (lateral) (n=2)<br>Pinch grip (tip) (n=2)<br>Grip strength (power) (n=2)<br>Heavy extension (n=2)<br><br><br>Ability to lift weight, undefined (n=1)<br>Number of repetitions movement can be performed in 10 seconds (n=1)<br>Maximum weight sustained when flexing elbow (n=1)

For peer review only

Supplementary file 7. Measurement instruments mapped to domains

						**Unclear (n= 3)**<br><br>Force recovery: Cross sectional area of the muscle under isometric contraction divided by cross sectional area at rest (n=1)
	Active movement	5	105	4	63	SST(n=1), MHQ (n=1), UCLA shoulder rating scale (n=1), MPI(n=2), CONSTANT- MURLEY(n=2) (2xPRO, 8x ClinRO), ARAT (PerfO, n=2), ULM (PerfO, n=2), Goniometry(n=50), MALLET (n=1), Visual assessment (n=32), First web space in cm (n=3), Total active movement(n=2), Pulp to palm distance (n=2)<br>Months to full active movement (n=1)<br>Months to antigravity movement (n=3)<br>Months to initial movement (n= 1)<br>Months to independent movement without donor (n=1)<br>Not clear (n=63)
	Passive range of movement		6		7	Not defined (n=7), Goniometry(n=6)
	Movement control and stability		1	1	2	MPI (ClinRo, n=1), ULM (PerfO, n=1), Not clear (n=2)
	Bone structure/position/healing				4	Not clear (n=4)
	Muscle mass				4	Not clear(n=4)
	**Nervous system outcome subdomains**					
	General sensory recovery including proprioception		**9**		**8**	Sensory BMRC (n=5), Modified Sensory BMRC (n= 2), Highet classification(n=2), Not clear (n=8)
	Discriminative touch (light touch, two point discrimination, vibration, object recognition)	**1**	**14**			MHQ (n=1), Cotton wool (n=3), Semmes Weinstein Monofilaments (n=4), Two point discrimination(n=2), Tuning fork (n=4), Not defined (ClinRo, n=1)
	Protective touch (pain, temperature, deep pressure)		**3**		**7**	Blunt pin (n=3), Not clear (n=7)
	Structure of peripheral nervous system		1			MRI (n=1)
	Reinnervation (level of reinnervation, time to innervation)		54			Two point scale on EMG(n=1) Four point scale on EMG (n=4), Not clear EMG (n= 49)
	Progression of regeneration		5			Tinel sign (n=5)

For peer review only

For peer review only - http://bmjopen.bmj.com/site/about/guidelines.xhtml

Supplementary file 7. Measurement instruments mapped to domains

	Speed of motor and sensory conduction		9			EMG (n=9)
	**General outcomes / symptoms**					
	Pain intensity/ relief	81			3	DASH (n=27), ASES (n=1), TAPES (n=1), VAS(n=20), NRS(n=12),MHQ (n=1) WBFRS(n=1), BPI (n= 4), UNWNS (n=1), McGill Pain Questionnaire SF (n=2), McGill pain questionnaire (n= 2), MPI (n=1), CONSTANT-MURLEY (n=2), 4 point scale (n=3) Author developed questionnaire(n=1), Not Clear (n=3), QuickDash (n=1), EQ5D 3L (n=1)
	Pain duration or frequency	18	0	0	0	SST (n=1), SF36 (n=8), MHQ (n=1),TAPES(n=1), NPSI (n=1), BPI (n= 4), UCLA shoulder rating score (n=1), Not described PRO (n=1)
	Pain quality	8				TAPES (n= 1), NPSI(n=1), UWNS(n= 1), McGill SF(n=2), McGill (n=2), Non described PRO (n =1)
	Pain when arm exposed to cold	1				NPSI (n=1)
	Paraesthesia	28				DASH (n=27), QuickDash(n=1)
	Sensitivity to touch, pressure, vibration etc	3				NPSI (n=1) UWNS (n= 1), NRS (n=1)
	Location of pain	4				BPI (n=4)
	Pain medication use	4				BPI (n=4)
	Stiffness	27				DASH (n=27)
**LIFE IMPACT**	**Physical functioning**					
	Physical function non-specific	3				PSFS (n=2), TAPES (n=1)
	Lower limb and non-upper limb function (walking, running, climbing stairs etc)	14			1	SF36 (n=8), TAPES (n= 1), BPI (n=4) Non described PRO (n=1), EQ5D-3L (n=1)
	Reaching, pulling, pushing, carrying, throwing , lifting	41		4		DASH (n=28), UEFI (n=2), MHQ(n=1), ASES(n=1), SST (n=1), SF36(n=8), ARAT(n=2), AMULA (n=1) UNBtP (n=1)
	Turning twisting, gripping and releasing with the arm	33		6	1	DASH (n=28), UEFI (n=2), MHQ (n=1),ARAT(n=2),SHAP(n= 2), JHFT (n=1), AMULA (n=1), UNBtP (n=1), Not clear (n=1), QuickDash (n=1)
	Fine hand movement include writing	32		7		DASH (n=28), UEFI (n=2), MHQ (n=1),ARAT(n=2), SHAP(n=2), JHFT (n=1) Purdue Peg test (n=1),AMULA (n=1), UNBtP (n=1)
	**Role Functioning**					

For peer review only

Supplementary file 7. Measurement instruments mapped to domains

	Impact on return to work	46				DASH (n =28), UEFI (n=2),MHQ (n=1), ASES (n=1), SST (n=1), SF36 (n=8), TAPES (n=1), MPI (n=1) No description PRO (n=1), Questionnaire no data (n=1), QuickDash(n=1)
	Role function patient specific	2				PSFS(n=2)
	Carrying out daily routine, (including food preparation, housework, garden, plants)	43	1	5		DASH (n=28), UEFI (n=2), MHQ (n=1),TAPES(n=1) , BPI (n=4), UCLA (n=1), SHAP (n=2), Jebsen (n=1), ULM (n=1) Questionnaire not defined (n=2),No description PRO (n=1) Unclear CLinRO(n=1), AMULA (n=1), UNBtP (n=1), QuickDash(n=1), EQ5D 3L (n=1)
	Maintaining personal hygiene	41		2		DASH (n=28), ASES (n=1), SST(n=1), SF36(n=8), MHQ(n=1) AMULA (n=1), UNBtP (n=1), QuickDash(n=1), EQ5D 3L (n=1)
	Maintaining personal appearance	3		1		UEFI (n=2), ASES (n= 1), AMULA (n=1)
	Dressing	33		2		DASH (n =28), UEFI (n=2), MHQ (n=1),ASES (n= 1), SST (n=1), AMULA (n=1) SHAP(n=2)
	Transport needs	29				DASH (n =27), UEFI (n=2),
	Impact on recreational activities and sport	36				DASH (n =28), UEFI (n=2), ASES (n= 1), TAPES(n=1), CONSTANT-MURLEY (n=2),Not described PRO (n=1), QuickDash(n=1)
	**Social functioning**					
	Effect on relationship with family, friends, neighbours and groups	43				DASH (n =28), SF36 (n=8), TAPES (n=1), MHQ (n=1), QuickDash(n=1), BPI (n=4)
	Effect on intimate relationships	28				DASH (n =28)
	**Emotional Functioning**					
	Emotional distress/ mood	18				SF36 (n=8), TAPES (n= 1),BPI(n=4), UWNS(n=1), Self-rated anxiety scale (n=1), Self-rated depression scale (n=1), MHQ (n=1), EQ5D 3L (n=1)
	Thoughts and beliefs (acceptance and adjustment)	1				TAPES (n=1)
	Self-esteem and self confidence	29				DASH (n=28), TAPES(n= 1)
	Body image	3				MHQ (n= 2), Not described (n=1)
	**Sleep and overall health**					

For peer review only

Supplementary file 7. Measurement instruments mapped to domains

	Impact on sleep	41				DASH (n=28), UEFI (n=3), ASES(n= 1), MHQ (n=1), SST (n=1), BPI(n=4), CONSTANT- MURLEY(n=2),Not described PRO (n=1)
	**General Quality of life**	1				Not described PRO (n=1)
	**Perceived Health Status**	10				SF36 (n=8), TAPES (n=1), Eq5D 3L (n=1)
	**Delivery of Care**					
	Patient satisfaction	10				TAPES (n=1), UCLA (n=1), MHQ (n=1),10-point scale (n=1) 4 point scale (n=2), 3 point likert scale (n=1), Questionnaire not described (n=1),Not defined PRO(n=2)
	Patient preference for treatment	1				Not described (n=1)
	Accessibility, quality and adequacy of intervention				1	4 point scale (n=1)
**RESOURCE**	**Hospital**					
	Operation time				1	Not described (n=1)
	**Adverse Events**					
**ADVERSE EVENTS**	Donor site motor morbidity to include weakness		18		19	BMRC (n=7), BMRC modified(n=2), Dynanometry (n=8), EMG(n=1),Not clear (n=19)
	Donor site sensory morbidity	1	3		4	10-point scale PRO (n=1),Not defined (n=4),2PD (n=2), Monofilaments (n=1)
	Donor site morbidity -pain	3				Not defined PRO (n=3)
	General complications				3	Unclear (n=3)
	Respiratory complications	1	5		4	4 point scale PRO (n=1), x-ray (n=2), FEV (n=1), TLC(n=1), MVV (n-1), Not defined (n=4),
	Vascular complications		2		13	Not defined (n=13), Visual assessment (n=1), USS (n=1)
	Musculoskeletal complications		2		19	Not defined CLinRO(n=2), Unclear (n=19)
	Infection complications		1		2	Not defined ClinRo(n=1), Unclear (n=2)
		757	370	52	169	

For peer review only

Supplementary file 7. Measurement instruments mapped to domains

DASH Disabilities of the arm shoulder and hand, *UEFI* Upper Extremity Functional Index, *MHQ* Michigan Hand Questionnaire, *BMRC* British Medical Research Council, *ULM* Upper Limb Module, *SHAP* Southampton Hand Assessment Procedure, *SST* Simple Shoulder Test, *MPI* Mayo clinic Performance Index for the elbow, *ARAT* Action Research Arm Test, *ClinRO* Clinician Reported Outcome, *PerfO* Performance Outcome, *PRO* Patient Reported Outcome, *ASES* American Shoulder and Elbow Surgeons Index, *TAPES* The Trinity Amputation and Prosthesis Experience Scales, *VAS* Visual Analogue Scale, *NRS* Numerical Rating Scale, *WBFRS* Wong Baker Faces Rating Scale, *UNWNS* University of Washington Neuropathic pain Score, *SF36* Short Form 36 health survey, *NPSI* Neuropathic Pain Symptom Inventory, *BPI* Brief Pain Inventory, *PSFS* Pain Specific Functional Scale, *AMULA* American Measures for Upper Limb Amputees, *UNBPT* University of New Brunswick test of Prosthetics function, *JHFT* Jebsen Hand Function Test, *FEV* Forced Expiratory Volume, *TLC* Tidal Lung Capacity, *MVV* maximal voluntary ventilation, *USS* Ultrasound Scan.

For peer review only

PRISMA 2009 Checklist

Section/topic	#	Checklist item	Reported on page #
**TITLE**			
Title	1	Identify the report as a systematic review, meta-analysis, or both.	1
**ABSTRACT**			
Structured summary	2	Provide a structured summary including, as applicable: background; objectives; data sources; study eligibility criteria, participants, and interventions; study appraisal and synthesis methods; results; limitations; conclusions and implications of key findings; systematic review registration number.	3
**INTRODUCTION**			
Rationale	3	Describe the rationale for the review in the context of what is already known.	5-6
Objectives	4	Provide an explicit statement of questions being addressed with reference to participants, interventions, comparisons, outcomes, and study design (PICOS).	7
**METHODS**			
Protocol and registration	5	Indicate if a review protocol exists, if and where it can be accessed (e.g., Web address), and, if available, provide registration information including registration number.	7
Eligibility criteria	6	Specify study characteristics (e.g., PICOS, length of follow-up) and report characteristics (e.g., years considered, language, publication status) used as criteria for eligibility, giving rationale.	8
Information sources	7	Describe all information sources (e.g., databases with dates of coverage, contact with study authors to identify additional studies) in the search and date last searched.	8
Search	8	Present full electronic search strategy for at least one database, including any limits used, such that it could be repeated.	Supplementary 2
Study selection	9	State the process for selecting studies (i.e., screening, eligibility, included in systematic review, and, if applicable, included in the meta-analysis).	9
Data collection process	10	Describe method of data extraction from reports (e.g., piloted forms, independently, in duplicate) and any processes for obtaining and confirming data from investigators.	9-10
Data items	11	List and define all variables for which data were sought (e.g., PICOS, funding sources) and any assumptions and simplifications made.	9-10
Risk of bias in individual studies	12	Describe methods used for assessing risk of bias of individual studies (including specification of whether this was done at the study or outcome level), and how this information is to be used in any data synthesis.	9
Summary measures	13	State the principal summary measures (e.g., risk ratio, difference in means).	N/A
Synthesis of results	14	Describe the methods of handling data and combining results of studies, if done, including measures of consistency (e.g., I^2) for each meta-analysis.	11

For peer review only - http://bmjopen.bmj.com/site/about/guidelines.xhtml

PRISMA 2009 Checklist

Section/topic	#	Checklist item	Reported on page #
Risk of bias across studies	15	Specify any assessment of risk of bias that may affect the cumulative evidence (e.g., publication bias, selective reporting within studies).	9
Additional analyses	16	Describe methods of additional analyses (e.g., sensitivity or subgroup analyses, meta-regression), if done, indicating which were pre-specified.	N/A
**RESULTS**			
Study selection	17	Give numbers of studies screened, assessed for eligibility, and included in the review, with reasons for exclusions at each stage, ideally with a flow diagram.	13 (Fig 1)
Study characteristics	18	For each study, present characteristics for which data were extracted (e.g., study size, PICOS, follow-up period) and provide the citations.	14-16
Risk of bias within studies	19	Present data on risk of bias of each study and, if available, any outcome level assessment (see item 12).	n/a
Results of individual studies	20	For all outcomes considered (benefits or harms), present, for each study: (a) simple summary data for each intervention group (b) effect estimates and confidence intervals, ideally with a forest plot.	n/a
Synthesis of results	21	Present results of each meta-analysis done, including confidence intervals and measures of consistency.	n/a
Risk of bias across studies	22	Present results of any assessment of risk of bias across studies (see Item 15).	23-24
Additional analysis	23	Give results of additional analyses, if done (e.g., sensitivity or subgroup analyses, meta-regression [see Item 16]).	n/a
**DISCUSSION**			
Summary of evidence	24	Summarize the main findings including the strength of evidence for each main outcome; consider their relevance to key groups (e.g., healthcare providers, users, and policy makers).	24-25
Limitations	25	Discuss limitations at study and outcome level (e.g., risk of bias), and at review-level (e.g., incomplete retrieval of identified research, reporting bias).	26
Conclusions	26	Provide a general interpretation of the results in the context of other evidence, and implications for future research.	27
**FUNDING**			
Funding	27	Describe sources of funding for the systematic review and other support (e.g., supply of data); role of funders for the systematic review.	30

From: Moher D, Liberati A, Tetzlaff J, Altman DG, The PRISMA Group (2009). Preferred Reporting Items for Systematic Reviews and Meta-Analyses: The PRISMA Statement. PLoS Med 6(7): e1000097. doi:10.1371/journal.pmed1000097

For more information, visit: **www.prisma-statement.org**.

For peer review only - http://bmjopen.bmj.com/site/about/guidelines.xhtml
